# The comparative energetics of the ray-finned fish in an evolutionary context

**DOI:** 10.1093/conphys/coac039

**Published:** 2022-07-05

**Authors:** Konstadia Lika, Starrlight Augustine, Sebastiaan A L M Kooijman

**Affiliations:** Department of Biology, University of Crete, Voutes University Campus, 70013, Heraklion, Greece; Akvaplan-niva, Fram High North Research Centre for Climate and the Environment, Postboks 6606, 9296 Tromsø, Norway; Department of Theoretical Biology, VU University Amsterdam, de Boelelaan 1087, 1081 HV Amsterdam, The Netherlands

**Keywords:** Add-my-Pet collection, Dynamic Energy Budgets, metabolism, waste-to-hurry, life history, population growth rate

## Abstract

To address challenges in management and conservation of fishes and fisheries it is essential to understand their life histories and energetics. The Add-my-Pet (AmP) collection of data on energetics and Dynamic Energy Budget (DEB) parameters currently contains 1150 of the 40000 extant species of fish. It gives 250–280 traits per species, depending on the model type that was applied, such as maximum reserve capacity, lifespan, specific respiration and precociality index, based on which the ray-finned fish (Actinopterygii) was compared with the four other fish classes (Cyclostomata, Chondrichthyes, Actinistia, Dipnoi) and the Tetrapoda. The Actinopterygii are the only vertebrate class that shows metabolic acceleration, and clearly so in only three sub-clades. Different from chondrichthyans, quite a few species follow the waste-to-hurry strategy, especially small bodied freshwater fish such as tropical annual killifish, but also in small minnows and darters in continental climates. We briefly discuss links between waste-to-hurry, which is associated with a large specific somatic maintenance, and sensitivity for pesticides. We discuss why this interferes with the physical co-variation between maximum reserve capacity and ultimate structural length or weight and explains why maximum reserve capacity increases with body length in chondrichthyans, but not in actinopterygians. Reserve capacity has relevance, e.g. mass-specific maintenance, starvation and the kinetics of lipophyllic compounds (such as pesticides), since reserve is relatively rich in lipids in fish. Also, unlike chondrichthyans, the size at birth is very small and not linked to ultimate size; we discuss the implications. Actinopterygians allocate more to soma, compared with chondrichthyans; the latter allocate more to maturity or reproduction. Actinopterygians, Actinistia and Dipnoi are near the supply-end of the supply–demand spectrum, while chondrichthyans clearly show demand properties.

## Teaser

Life history and energetics of Actinopterygii are analysed using parameters and traits from the Add-my-Pet collection. Actinopterygii are the only vertebrate class that shows metabolic acceleration a short period after birth, and they are near the supply-end of the supply–demand spectrum. Many small freshwater species sport the waste-to-hurry strategy.

## 1 Introduction

The conservation relevance of eco-physiology knowledge about fishes is self-evident (McKenzie1 *et al.*, 2016, Ren *et al.*, 2020). Almost all commercial marine species experience severe reductions of standing crop due to intense pressure by fisheries (Hilborn *et al.*, 2020), while many non-commercial freshwater species suffer from environmental pollution and degradation, blocking of migration routes and rapid climate change (Hughes, 2021). Half of the fish species live in freshwater and a third of the freshwater species run the risk of extinction. The fact that many species live in a very much restricted geographical area, such as a single pool or stretch of creek, makes them extra vulnerable. Sustainable management of fisheries is difficult due to the high demand for fish, illegal uncontrolled fishing and also because population dynamical models can hardly be called reliable (see below). Better knowledge of fish biology could not only help resolve this deep and complex problem, but also optimize aquaculture of fish (Chary *et al.*, 2020, Sarà *et al.*, 2018, 2014, Stavrakidis-Zachou *et al.*, 2021). A proper understanding of eco-physiological properties is key to understanding population dynamics (Kooijman *et al.*, 2020).

Dynamic Energy Budget (DEB) theory (Kooijman, 2010) is suitable for comparative analysis of energetics and life histories in dynamic environments, since it is based on first principles, such as energy and mass conservation, surface area–volume relationships and various forms of homeostasis, while being simple enough to be applicable to many species with limited data in the literature. It seems to be unique in this respect (Kooijman, 2020b). The median relative error of predictions for some 45000 data sets, e.g. growth, reproduction and respiration for over 3000 animal species is only 5%; for over 1000 species of actinopterygians even 4% (AmP, 2022). The Add-my-Pet (AmP) database (AmP, 2022) is an open access, online, collection of data on energetics, DEB parameters and implied traits for animals (AmP, 2022, Marques *et al.*, 2018). It is run as a journal, so everyone can contribute and some 175 authors did, so far. This paper is part of a series of case studies on selected taxa from AmP whereby DEB parameters and associated traits are represented in eco-evolutionary context. Previous studies were on five classes of fish (Kooijman & Lika, 2014a), petrels and penguins (Kooijman, 2020a); ones on cephalopods (Kooijman & Augustine, 2022b), chondrichthyans (Augustine *et al.*, 2022), turtles and crocodiles (Marn & Kooijman, 2022) and carnivores and pangolins (Kooijman & Augustine, 2022a) are underway. This study focusses on ray-finned fish (Actinopterygii) and compares them with the four other fish classes (Cyclostomata, Chondrichthyes, Actinistia, Dipnoi) and the tetrapods, which form the clade Sarcopterygii with the latter two taxa.

Our 2014 review (Kooijman & Lika, 2014a) on the five fish classes was based on 64 species. Now, the AmP collection has 1149 fish species and our first research question is if our earlier assessments need updating while we also wanted to discover patterns in changes in parameter values during evolution. Our second research question is to find new patterns in the co-variation of DEB parameters. In this context, we made a special study of the 790 North American freshwater fish species and found data for 450 of them. We have the impression that this group of fish is now complete, meaning that it will not be that easy to find more species-with-data in the open literature. The number of species in the AmP collection and the percent of each of the five fish classes and the tetrapods covered in AmP are provided in Table 1. The number of species of fish and tetrapods are about equal, both in terms of extant species and AmP species.

**Table 1:** The number of vertebrate species in the AmP collection at 16 October 2021, the number of extant species (estimates from Wikipedia) and the coverage

Taxon	AmP	Extant	Coverage
Actinopterygii	934	30000	3.1 %
Cyclostomata	12	120	10.0 %
Chondrichthyes	200	1120	17.9 %
Actinistia	1	2	50.0 %
Dipnoi	2	6	33.3 %
Tetrapoda	1286	33278	3.9 %

The method section first gives some background and concepts of DEB models, then it introduces implied traits and recognized patterns in the co-variation of parameter values. The AmP data base is introduced, as well as the ray-finned fish. The results section reports patterns that we found in parameter values in the context of known co-variation patterns and presents the results of multidimensional scaling. The waste-to-hurry pattern is discussed in more detail since it underlies unexpected parameter patterns for ray-finned fish. A general discussion section puts the findings in a wider evolutionary context.

## 2 Materials and methods

### 2.1 DEB models

DEB theory captures the metabolic dynamics of an individual organism through its entire life cycle, covering the start of embryo development to death by aging through a range of life stages. Differential equations, derived from a small set of assumptions, describe how state variables of an individual (reserve energy, structural length, cumulated energy investment into maturation and reproduction) change in time through all life stages. Supplementary Section A.1 summarizes, respectively, the metabolic processes, the dynamics of the state variables and the DEB parameters. For an accessible summary of the principles of DEB theory we refer the reader to Kooijman (2012) or, for a more physical setting, to Jusup *et al.* (2017).

DEB theory aims to capture many aspects of the energetics mechanistically, such as food searching, feeding, defecation, digestion, storing, development, aging, growth, reproduction and the fluxes of CO$_2$, H$_2$O, O$_2$, NH$_3$ and heat, explicitly respecting energy and mass conservation and stoichiometric constraints. This phrasing applies to animals, but DEB theory is meant to apply to all organisms.

DEB theory includes embryo development, where the egg initially consists of reserve only, and fixed fraction $\kappa $ of mobilized reserve fuels somatic maintenance and the growth of structure; the rest is allocated to maturity maintenance and maturation ($\kappa $-rule). The theory can be used to produce a maturity-based staging atlas for embryo development (Augustine *et al.*, 2011), which has the advantage above age- or length-based atlases in being less sensitive for environmental factors. Foetal development is a variation on egg development, where the mother replenishes embryo reserve across the placenta. Birth is defined as the moment at which feeding starts, but otherwise development just continues smoothly; food-derived metabolites replenish reserve in a process called assimilation. The onset of feeding occurs when maturity hits a threshold value $E_H^b$. Reserve density, i.e. the ratio of the amounts of reserve and structure, at birth equals that of the mother at egg laying: the maternal effect. Hatching can be quite a bit earlier than birth, where the larva lives of the yolk sack (= reserve); sometimes the mouth is not yet open (Shirota, 1970, Srithongthum *et al.*, 2020).

Puberty, i.e. the ceasing of maturation and the onset of allocation to reproduction, occurs when maturity hits a threshold value $E_H^p$. A reproduction buffer is then filled, but transformation of the contents to eggs is species-specific, frequently in interaction with environmental factors.

DEB theory takes the hazard rate for death by aging proportional to the density of damage compounds (e.g. modified proteins), which are generated by damage inducing compounds (e.g. modified mitochondrial DNA), which are formed by reactive oxygen species, so coupled to respiration. Respiration has links with assimilation, maintenance, growth and reproduction. Under constant environmental conditions this leads to a two-parameter aging module that has both the Weibull and the Gompertz models as special cases. For actinopterygians the Gompertz stress coefficient is taken almost zero, except for species that sport programmed death, where this coefficient is set equal to 1 and the survival probability decays suddenly after reaching the mean age at death, rather than more gradually.

DEB theory assumes that the main component of somatic maintenance is proportional to structural volume (e.g. maintaining concentration gradients across membranes, turnover of structural body proteins, a mean level of movement and production of scales) and a minor component is proportional to surface area (osmosis, thermal heating). We have not yet met cases that allowed us to estimate surface area-linked maintenance costs as function of the ion-strength of the water. Scatter in data can easily make this impossible, since it is just one parameter from a list that needs to be estimated. Moreover, we expect that this parameter will not be constant, even for constant environments, due to time-dependent adaptation. A surface-area linked maintenance would reduce ultimate length or weight in a DEB context and we did not meet cases where ultimate body size in different populations could be linked to ion-strength. Differences in food availability or quality can easily obscure this expected effect. We will come back to this issue in the Discussion.

Endothermy of particular body parts occurs in few big-bodied actinopterygians (Dickson & Graham, 2004), such as tunas (Jusup *et al.*, 2014, 2011), swordfish and billfish, which represent another surface area-linked maintenance drain. The estimation of its costs suffers from the same problems as osmosis. The costs might be small, however, since tunas tend to swim to warmer surface waters, transport blood to their skin to calibrate rapidly, before diving again to cooler deeper waters.

The standard DEB model (std) is the most simple one in the family of DEB models. It includes three life stages (embryos, juveniles and adults) and assumes isomorphic growth over all life stages. That is, organisms do not change shape during growth, which implies that surface area is proportional to structural length squared. All DEB models are simple extensions of the std-DEB model (Marques *et al.*, 2018). The std model as well as the abj model that accounts for metabolic acceleration (see Section 2.2) was used for the actinopterygians. During metabolic acceleration the surface area grows proportionally to structural volume (i.e. structural length cubed; see Supplementary Section A).

### 2.2 DEB traits and patterns

All DEB parameters have a physical interpretation, and, therefore, simple dimensions. Traits are here defined as DEB parameters and any conceivable function of these parameters. Table 2 gives the list of traits discussed in the present study, and the Supplementary Section A details the formulae and DEBtool functions to compute them.

**Table 2:** A short list of traits defined as DEB primary or compound parameters and functions of these parameters. The process associated with each primary parameter is given in parentheses. Notation: square brackets, [ ], indicate parameters expressed per unit of structural volume, and curly brackets, { }, per unit of structural surface area.

Symbol	Units	Interpretation
$\{\dot {p}_{Am}\}$	J d$^{-1}$ cm$^{-2}$	Maximum specific assimilation rate (assimilation)
$\dot {v}$	cm d$^{-1}$	Energy conductance (mobilization)
$\kappa $	–	Allocation fraction to soma (allocation)
$[\dot {p}_M]$	J d$^{-1}$ cm$^{-3}$	Somatic (volume specific) maintenance rate (turnover, activity)
$[E_m] = \frac {\{\dot {p}_{Am}\}}{ \dot {v}}$	J cm$^{-3}$	Maximum energy density
$L_m = \frac {\kappa \{\dot {p}_{Am}\}}{[\dot {p}_M]}$	cm	Maximum volumetric length
$s_{\mathcal {M}} = \frac {L_j}{L_b}$	-	Acceleration factor
$s_H^{bp} = \frac {E_H^b}{E_H^p}$	-	Precociality coefficient
$s_s = \frac {\dot {k}_J E_H^p [p_M]^2}{f^3 s_{\mathcal {M}}^3 \{\dot {p}_{Am}\}^3}$	-	Supply stress ($f \in [0,1]$: scaled functional response)
$a_b, a_p, a_m$	d	Age at birth, puberty, death
$W_w^b, W_w^p, W_w^\infty $	g	Wet weight at birth, puberty, ultimate
$L_\infty =s_{\mathcal {M}} L_m$	cm	Ultimate volumetric length
$\dot {r}_N$	d$^{-1}$	Specific population growth rate
$\dot {r}_m$	d$^{-1}$	Specific body growth rate at maximum growth
$j_O^\infty $	mol d$^{-1}$ g$^{-1}$	Weight-specific O$_2$ consumption rate at ultimate size
$\dot {R}_\infty $	# d$^{-1}$	Maximum reproduction rate at ultimate size
$N_\infty W_w^b$	g	Life time neonate mass: product of the life time reproductive output $N_\infty $ and the neonate wet weight $W_w^b$
$j_{W_w^b}^\infty $	mol d$^{-1}$ g$^{-1}$	Weight-specific neonate mass production rate at ultimate size

The maximum surface area-specific assimilation rate, $\{ \dot {p}_{Am} \}$, is linked to the assimilation process (increases reserve) and the conductance rate, $\dot {v}$, to the reserve mobilization process; the ratio of the two control the maximum reserve capacity: $[E_m]=\frac {\{ \dot {p}_{Am} \}}{\dot {v}}$; an implied trait that is very critical, e.g. during starvation periods.

Five patterns in DEB parameters have so far been identified: physical co-variation rules, metabolic acceleration, waste-to-hurry, supply–demand spectra and altricial–precocial spectra. We briefly introduce them.

The physical co-variation rules (Kooijman, 2010) state that (i) parameters are either intensive or extensive (i.e. the physical interpretation of intensive parameters does depend on the absolute size of the individual), (ii) appropriate ratios of extensive parameters are intensive and (iii) intensive parameters have the same values among all species. These simple rules couple all DEB parameters since maximum structural length, $L_m=\frac {\kappa \{ \dot {p}_{Am} \}} {[\dot {p}_M]}$, depends on a single extensive parameter, i.e. maximum specific assimilation rate, $\{\dot {p}_{Am}\}$, while allocation fraction $\kappa $ and specific somatic maintenance, $[\dot {p}_M],$ are intensive. Since energy conductance $\dot {v}$ is an intensive parameter, which follows from the mechanism behind reserve dynamics (Kooijman, 2010), we should expect that the maximum reserve capacity $[E_m] = \{\dot {p}_{Am}\}/ \dot {v}$ is extensive and co-varies with $\{\dot {p}_{Am}\}$ and, thus, with maximum structural length $L_m$. Co-variation rules also imply that maturity levels at birth and puberty are proportional to cubed $L_m$, respiration proportional to $L_m$ to some power between 0.66 and 1 and length at birth proportional to $L_m$. Other traits that scale with maximum structural length are discussed in detail in Kooijman (2010).

Metabolic acceleration occurs a short period after birth during which the specific assimilation $\{\dot {p}_{Am}\}$ and the energy conductance $\dot {v}$ increase with structural length till maturity hits a threshold value $E_H^j$. This occurs well before the maturity threshold for puberty $E_H^p$ is reached and might or might not coincide with metamorphosis, i.e. sudden morphological changes. Acceleration is quantified by the acceleration factor $s_{\mathcal {M}} = \min (L,L_j)/ L_b$, where $L_b$ and $L_j$ are the structural lengths at birth and metamorphosis, respectively. After the acceleration period, the acceleration factor is constant and equals $s_{\mathcal {M}}=L_j/L_b$, but depends on food availability during the acceleration period. No acceleration is a special case for which $E_H^j = E_H^b$ and $s_{\mathcal {M}} = 1$. Metabolic acceleration is discussed in detail in Kooijman *et al.* (2011) and Lika *et al.* (2014b).

The $\kappa $-rule of DEB theory has the implication that a simultaneous increase in somatic maintenance and specific assimilation enhances growth and reproduction. The simultaneous increase of both somatic maintenance and specific assimilation is termed waste-to-hurry strategy. It is typically found in small-bodied species that experience seasonal short periods of food abundance and combine it with strategies to cope with food scarcity between these periods. Waste-to-hurry is discussed in detail in (Kooijman, 2013).

The structure of the DEB models is a mix of components with supply (growth, maturation/reproduction) and demand (somatic and maturity maintenance) organization. The supply stress $s_s$ is defined as the ratio of the maturity maintenance times the squared somatic one and the cubed assimilation rate (2). This dimensionless quantity can take values between 0 and 4/27, is independent of structural length and quantifies the distance to the supply-end in the ranking of species in the supply–demand spectrum. The supply–demand spectra is discussed in detail in Lika *et al.* (2014a).

The altricial–precocial spectrum, where altricial species are born in an early state of development and precocial species in a late one, is quantified, within the framework of DEB theory, by the precociality coefficient, $s_H^{bp}=\frac {E_H^b}{E_H^p}$, the ratio of maturity levels at birth and puberty (Table 2). This index takes values between 0 (extreme altricial) and 1 (extreme precocial). The altricial–precocial spectra is discussed in detail in Augustine *et al.* (2019a).

### 2.3 AmP database

Measured data are used for parameter estimation and parameter values are used for the evaluations of traits. The availability of data varies considerably among species, but this two-step procedure allows us to compare many traits for all species. The AmP website (AmP, 2022) gives 250–280 traits for each of its over 3000 species, depending on the model type that was applied. A list of species, with the data types that are used and references, is given in Supplementary Table B.1. The collection has measured data with references, the DEB parameters and the code that has been used to estimate them from the data and a large number of implied traits, being functions of the parameters, including some at population level. All can be found on the AmP website (AmP, 2022). It is supported by two large software packages, DEBtool and AmPtool (AmPtool, 2022, DEBtool, 2021), which can be used for visualization and for computing traits at different (and varying) environmental.

For parameters that depend on temperature, we use the reference temperature of 20 $^\circ $C. The AmP site gives the temperature correction factor that has been used for the values based on the body temperature, which includes arctic and deep water species.

The AmP collection has many examples where temperature is not constant and is typically also coupled to variations in food availability. These types of variations affect growth and reproduction trajectories considerably. Although the size-trajectory of DEB models reduces to the von Bertalanffy (or better Pütter, see Kearney, 2020) growth model after metabolic acceleration in constant environments, these models differ substantially in dynamic environments. Asympototic size and the von Bertalanffy growth rate are not DEB parameters, and the von Bertalanffy growth model does not account for interactions with the environment, while such interactions are key to DEB models.

### 2.4 Ray-finned fish

Although AmP has a dozen extinct species, the focus is on extant ones. For comparative practical reasons we here use the label CL-group (= coelacanth + lungfish) for the classes Actinistia plus Dipnoi because the number of species is that small. The coelacanth–lungfish–tetrapod group (Sarcopterygii) is widely accepted as a monophyletic sister group of the ray-finned fish (Actinopterygii), which is the largest of the five fish classes. These five classes are combined in the paraphyletic taxon Pisces. The Actinopterygii–Sarcopterygii group, the Osteichthyes, is a sister group of the cartilaginous fish, Chondrichthyes. The Myxini (= cyclostomes) are poorly represented in AmP because it is very difficult to age these deep-water species; only indirect evidence can be used to arrive at some rate or time estimates (Meer & Kooijman, 2014). Although the tetrapods look very different from fish, the external gills of lungfish larvae strongly resemble those of the caudata (Amphibia), illustrating the evolutionary continuum in morphology. We include tetrapods and the other fish classes here are outgroup for the actinopterygian, together forming the clade Vertebrata.

The actinopterygians span an impressive body size range as fully grown adults, from the tiny 6 mg dwarf minnow *Paedocypris progenetica* (Malmstrmø *et al.*, 2018), to the colossal 2.3 mg bump-head sunfish *Mola alexandrini* (Main, 2018); a factor of 9 orders of magnitude.

The huge number of tiny offspring of actinopterygians poses a formidable challenge for modelling their population dynamics (Leggett & Deblois, 1994). This is intrinsic to the strategy of especially ray-finned fish to feed their prey species with their offspring. The use of individual-based population modelling seems unavoidable (Hayes *et al.*, 2009), but such models require detailed information and have problems with a large number of individuals. Moreover, the trophic interactions within the plankton are complex (Woodward *et al.*, 2019), and plankton dynamics depends on many factors (Morabito *et al.*, 2018). What exactly happens in the plankton stage is mostly treated as a black box and often bypassed by refraining from linking recruitment to standing crop. Food availability is key to population dynamics. The large change in size during ontogeny in actinopterygians (the ratio of the median ultimate and birth weights in the AmP collection is $5.6 \times 10^5$) is associated with changes in diet during ontogeny, which further complicates models for population dynamics since the dynamics of all food types needs to be included.

About 2% of actinopterygian species is hermaphroditic (Avise & Mank, 2009), mostly sequential, but the sea basses, Serranidae, are synchronous (Petersen, 2006). Some 90% is oviparous, but several sub-taxa evolved ovovivipary (e.g. the halfbeaks Zenarchopteridae and the eelpouts Zoarcidae) and in 2% placental vivipary (Uribe & Grier, 2005), as in the surfperches Embiotocidae, with complex and intriguing transitions, such as in *Poeciliopsis* (Reznick *et al.*, 2002).

If the definition of parental care is restricted to parental behaviour, chondrichthyans do not show it, but many actinopterygians do (Goldberg *et al.*, 2020), in the form of nest building (the stickleback *Gasterosteus* is famous for this), brood pouches (e.g. pipe fish *Syngnathus* and seahorse *Hippocampus*), mouth breeding (e.g. tilapia *Oreochromis*, betta *Betta*, cardinalfish Apogonidae), patches of eggs on the skin (e.g. humphead *Kurtus* and banjo catfish *Platystacus*) and defending eggs, young and territories (with direct links to food availability; too many to give examples). These nests sometimes also function to attract females, like the spectacular 2-m-diameter nests of the small marine puffer *Torquigener albomaculosus* (Matsuura, 2015). Males of many actinopterygians change colour during breeding, or develop keratin warts on the snout, like in many Cypriniformes, or hooked beaks like in the semelparous Coho salmon, illustrating the importance of season-coupled hormonal cycles in this group, which has links with the handling rules for the reproduction buffer in DEB theory; the moment at which (part of) the buffer is converted to eggs or sperm and leaves the buffer is frequently triggered by temperature in species-specific ways. The modelling of multiple spawning requires the delineation of the part of reproduction buffer that will be used per spawn. This involves a single (Pecquerie *et al.*, 2009) or a few (Muller *et al.*, 2019) extra parameters and a temperature threshold to start the spawning cycle; it ends when the reproduction buffer is empty.

**Fig. 1:**
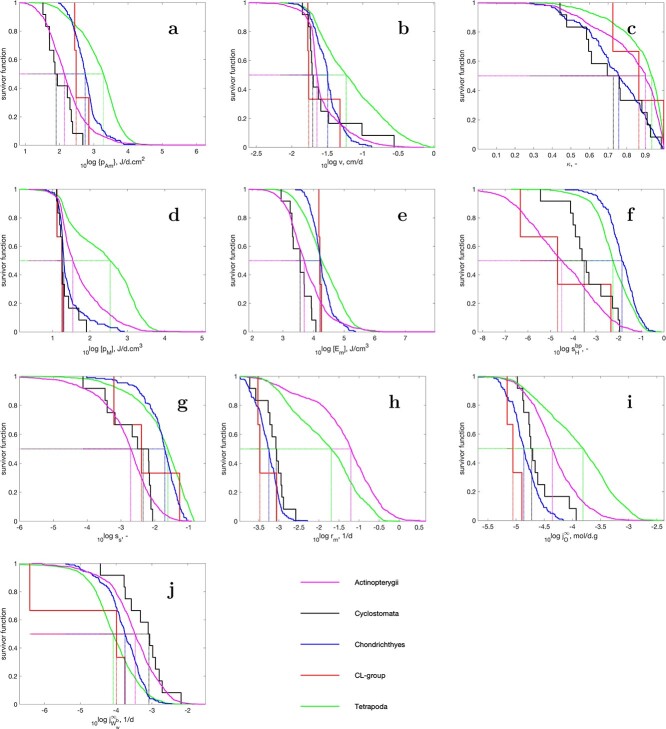
Survivor curves for parameters and other traits (i.e. the fraction of species with a parameter/trait-value that exceeds the value on the abscissa) for Actinopterygii taxa in the AmP collection. All traits are presented for a body temperature of 20 $^\circ $C

## 3 Results

### 3.1 Energetics and life history

The Actinopterygii is the only vertebrate class in which acceleration occurs. The acceleration factor reaches peak values in three clades only: Otomorpha/Protacanthopterygii, Paracanthomorphacea and three subclades of the Percomorphaceae: Scombrimopharia, Carangimopharia and Eupercaria. See https://www.bio.vu.nl/thb/deb/deblab/add_my_pet/phyla.html. But even within these accelerating taxa, some 25% of the member species do not accelerate. We see acceleration in the Actinopterygii as an extension of the planktonic stage to enhance dispersal (Lika *et al.*, 2014b), by starting the life cycle extra slowly. It comes with constraints on the absolute size to realize a low Reynolds number (Li *et al.*, 2016), which explains why neonate size hardly depends on the size of the fully grown adult: only if sufficiently small can fry float effortlessly in the water.

#### 3.1.1 Distribution of selected traits

**Fig. 2:**
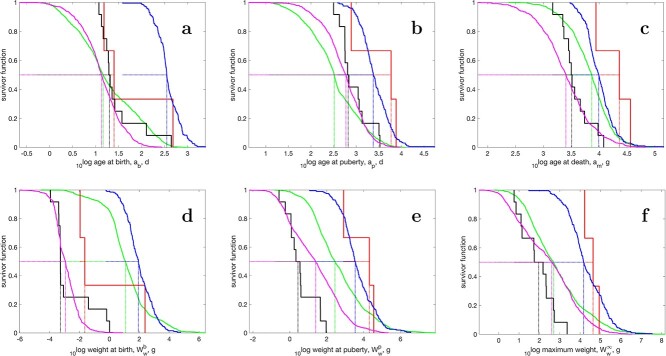
Survivor curves for (top) age at birth, puberty and death and (bottom) wet weight at birth, puberty and ultimate (i.e. the fraction of species with a parameter/trait-value that exceeds the value on the abscissa) for Actinopterygii taxa in the AmP collection: Actinopterygii (magenta), Cyclostomata (black), Chondrichthyes (blue), CL-group (Actinistia and Dipnoi) (red) and Tetrapoda (green). Ages are presented for a body temperature of 20 $^\circ $C.

Figures 1 and 2 present survivor curves of a selection of traits (Table 2), for vertebrate taxa at the reference temperature of 20 $^\circ $C. The value of the survivor function at a specified trait-value (on the abscissa) gives the fraction of species with a trait-value that exceeds the specified value. The distributions of the selected traits for Actinopterygii are compared with those for the four other fish classes (Cyclostomata, Chondrichthyes, Actinistia, Dipnoi) and tetrapods. The vertical lines indicate the median values of the trait for each taxa. Steep survivor curves indicate small variance within the taxa for that trait. The less smooth curves for Cyclostoma and the CL-group (Actinistia and Dipnoi) are due to the small number of species in these taxa. Although this type of comparison is rather crude, it already reveals some salient features with quite a bit of coherence.

Figure 1h shows that actinopterygians grow very fast, even faster than the tetrapods, while Chondrichthyans, cyclostomata and the CL-group grow equally slow. Chondrichthyans are known to grow slowly (Compagno, 1990). We come back to this in the discussion of Fig. 7.

Although actinopterygians have a high reproduction rate, and chondrichthyans a low one, expressed in terms of weight-specific neonate mass production rate, the differences are not that big (Fig. 1j); the cyclostomes are shown to have a high weight-specific neonate mass production rate.

That the differences in relative size at birth are large is shown in Fig. 2d, with direct consequences for age at birth (Fig. 2a). The median weight at birth is 0.5 mg for cyclostomes, 1.1 mg for actinopterygians, 22.4 mg for the CL-group, 11.8 g for tetrapoda and 88.8 g for chondrichthyans. The small weight for actinopterygians has little scatter and seems to be an adaptation to a planktonic larval stage, a property that dominates their life history. The median of the ratios of age at puberty (Fig. 2b) over life span (Fig. 2c) is 0.26 for the cyclostomes, 0.21 for the actinopterygians, 0.24 for the CL-group and 0.22 for the chondrichthyans; similar relative age at puberty. The median of the ratios of weight at puberty, $W_w^p$, (Fig. 2e) over ultimate weight, $W_w^\infty $, (Fig. 2f) is 0.06 for the cyclostomes, 0.08 for the actinopterygians, 0.41 for the CL-group and 0.24 for the chondrichthyans; a clear trend towards larger relative puberty size from cyclostomes to CL-group, while the side branch of chondrichthyans is close to the CL-group. That tetrapods have high ratio, $W_w^p/W_w^\infty = 0.87$, is mainly due to the birds, which hardly grow after puberty.

The investment into development and reproduction is relatively low (= large $\kappa $) in actinopterygians and tetrapods and high in chondrichthyans and cyclostomes (Fig. 1c). The remarkable shape of the survivor curve for $\kappa $ is explained in Lika *et al.* (2019). Weight at birth in actinopterygians has a low variance, since egg size of small- and large-bodied ray-finned fish species are very similar, and, thus, hardly depends on the weight of the fully grown adult, unlike the expectations of the physical co-variation rules. We see this as an adaptation to the planktonic life style of fish fry and part of the altricial–precocial spectrum (Augustine *et al.*, 2019a). This also explains the low score for the precociality coefficient (Fig. 1f), i.e. the ratio of the maturities at birth and puberty (Table 2), for actinopterygians and the CL-group, while chondrichthyans score even somewhat higher than the tetrapods; the maturity ratio does not deviate that much from the corresponding weight ratio in constant environments. The explanation is probably the relatively large size at birth for chondrichthyans (Fig. 2d).

Actinopterygians have high weight-specific respiration compared with the other fish taxa, but less than tetrapods (Fig. 1i), mainly due to the large contribution of birds and mammals, which are known to have a high metabolic rate; note that all times and rates are expressed at the common reference temperature of 20$^\circ $C. For the same reason, tetrapods have a high supply stress, making them demand species (Fig. 1g; see Lika *et al.*, 2014a), but the chondrichthyans also score remarkably high, very different from the actinopterygians, the CL group and the cyclostomes.

The median maximum reserve capacity for the chondrichthyans, the CL group and the tetrapods is equal and larger than that of the cyclostomes and actinopterygians (Fig. 1e). The maximum reserve capacity is the ratio of the maximum specific assimilation rate (Fig. 1a) and the energy conductance (Fig. 1b): $[E_m] = \{\dot {p}_{Am}\}/ \dot {v}$. The latter has a small variance (i.e. steep survivor curves) for the four fish taxa, but a larger variance for the tetrapods.

#### 3.1.2 Reserve capacity

**Fig. 3:**
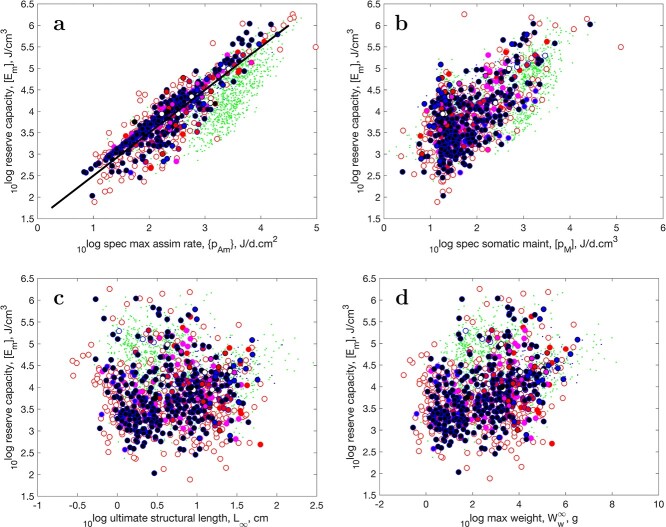
The maximum reserve capacity as function of specific assimilation (a), specific somatic maintenance (b), ultimate structural length (c) and maximum wet weight (d). Markers as in Fig. 4. The slope of the line equals one; for explanation, see text.

Figure 3 shows how the maximum reserve capacity co-varies with other parameters. Based on the physical co-variation rules, $[E_m] = \{\dot {p}_{Am}\}/ \dot {v}$ is extensive and we should expect that co-varies with $\{\dot {p}_{Am}\}$, which is confirmed (Fig. 3a) and not surprising in view that the scatter in $\dot {v}$ is small among fish (Fig. 1b).

For the same reason, we should expect that maximum structural length $L_m = \kappa \{\dot {p}_{Am}\}/ [\dot {p}_M]$ should co-vary with $\{\dot {p}_{Am}\}$, and so with $[E_m]$, since $\kappa $ shows little scatter. We found this co-variation for chondrichthyans (Augustine *et al.*, 2022), but not for actinopterygians (Fig. 3c). (The detailed argument is slightly more complex in view of metabolic acceleration, but acceleration does not affect reserve capacity since both specific assimilation and energy conductions are increased, so their ratio is not).

The reason is that the waste-to-hurry co-variation pattern interferes (Augustine *et al.*, 2019b, Kooijman, 2013), where both $\{\dot {p}_{Am}\}$ and $[\dot {p}_M]$ are increased. These two parameters are coupled in a natural way in the DEB model (Lika & Kooijman, 2011), since (demand-organized) somatic maintenance is paid from (supply-organized) assimilation, which excludes the combination of a large somatic maintenance with a small assimilation.

This mechanistic coupling causes that the extensive parameter $[E_m]$, which is proportional to $\{\dot {p}_{Am}\}$, also co-varies with the intensive parameter $[\dot {p}_M]$ (Fig. 3b). A large value for $[\dot {p}_M]$ causes a small value for $L_m$, all other things being equal, which obscures the relationship that was expected between $[E_m]$ and $L_m$. This coupling has a logical ecological functionality: if somatic maintenance is high, it is helpful to have substantial reserve during temporary starvation. That maximum reserve capacity has a slight tendency to co-vary with maximum ultimate weight (Fig. 3d) is due to the contribution of reserve to weight. Quite a few parameters contribute to the relationship, however, so the relationship is not really clear due to the huge scatter. We come back to this issue in the discussion.

#### 3.1.3 Respiraton, life span and reproduction

**Fig. 4:**
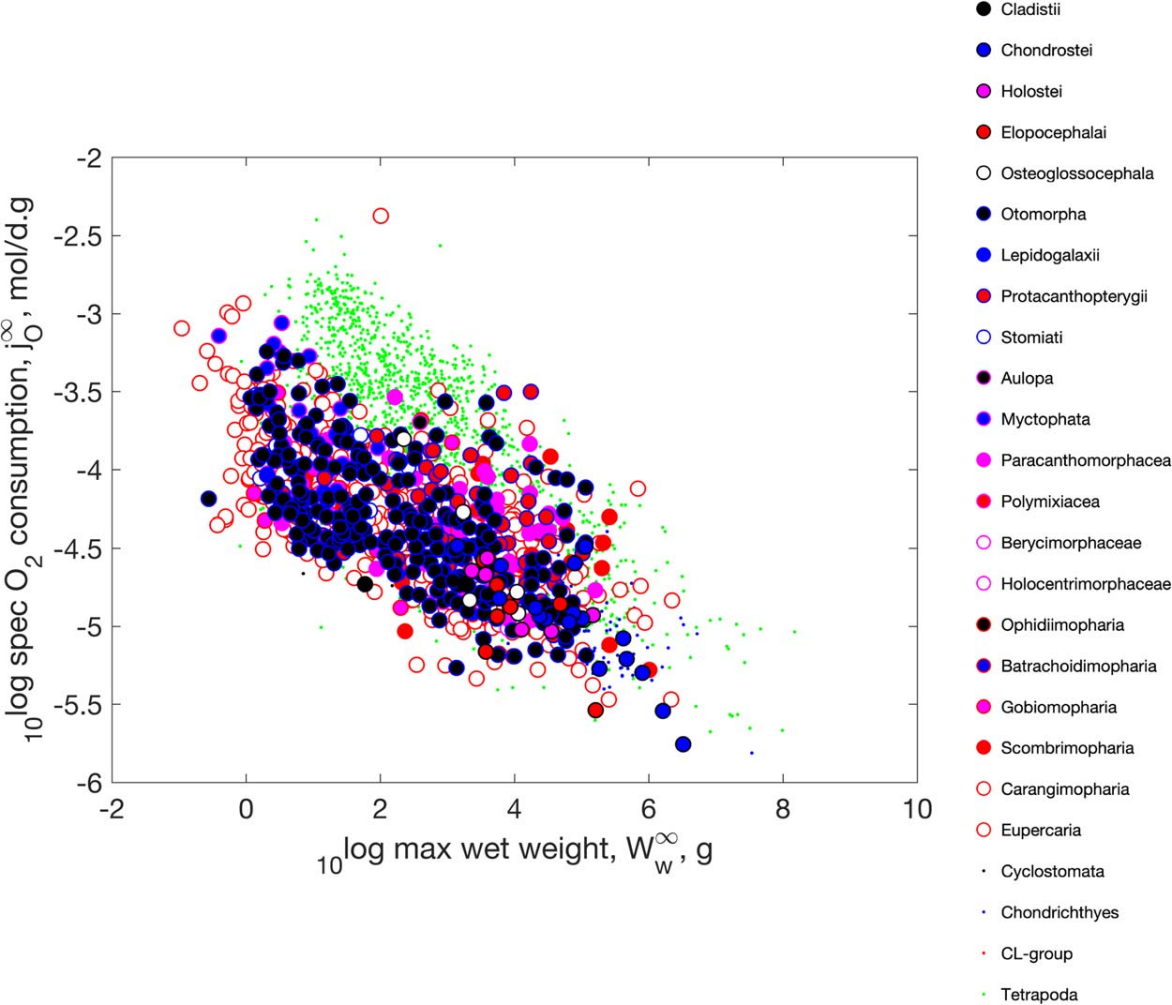
The weight-specific respiration as function of maximum wet weight. Actinopterygii is separated in sub-groups and have large symbols.

Figure 4 shows that Kleiber’s law also applies to Actinopterygii and is explained by the physical co-variation rules: The weight-specific respiration decreases for increasing weight; DEB theory does not make use of allometric functions and dioxygen consumption is, for constant food and temperature, a cubic polynomial in structural length, while weights have contributions from structure and reserve (see Supplementary Section A). So, the detailed value of ‘the’ slope in a log-log plot has little meaning in DEB context, but, if curvature is ignored, it is not far from around -1/4. The plots further shows that tetrapods have a steeper slope than ray-finned fish. That said, tetrapods are not a homogeneous group: it comprises birds and mammals that differ in intercept and not in slope (not shown here, as our focus is on ray finned fish). Despite the fact that all data is corrected for a common body temperature of 20 $^\circ $C, birds have a large intercept with a rather small size range, mammals have a somewhat smaller intercept with a larger size range and the rest an intercept that is comparable with the fishes, while their intra-taxon slopes are not very different. A frequently occurring phenomenon that devaluates many statistical analyses of body size scaling relationships in the literature, which typically have a strong focus on the slope, but hardly on intercepts, which have associated dimensional problems.

**Fig. 5:**
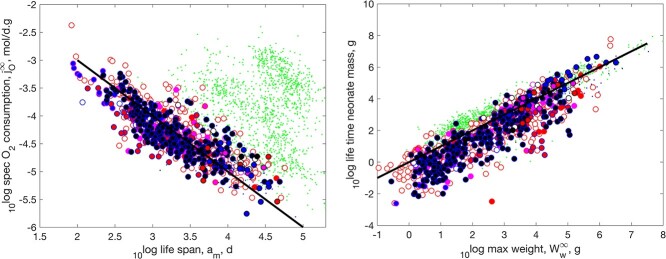
Left: The specific respiration rate of fully grown adults as function of the life span. The line has slope -1. Right: The life-time cumulated neonate mass as function of maximum weight. The line indicates equality. Markers like in Fig. 4.

Like we found for chondrichthyans (Augustine *et al.*, 2022), life span is inversely proportional to specific respiration in actinopterygians (Fig. 5 left). Also, the life-time cumulated neonate mass almost equals the ultimate adult mass (Fig. 5 right), but the relationships have quite some scatter.

If all parameters are kept fixed, except allocation fraction $\kappa $, reproduction is at maximum for values around $\kappa = 0.45$, depending on the values of other parameters. For values close to $\kappa = 1$, hardly anything is allocated to reproduction, and close to 0 investment in reproduction is also small, because such investments have to come from food and food intake is taken proportional to squared length, while hardly anything is allocated to growth (Kooijman & Lika, 2014b). How reproduction relates to $\kappa $ between species, where all parameters are estimated from data, can deviate substantially from this due to the variations in other parameters.

**Fig. 6:**
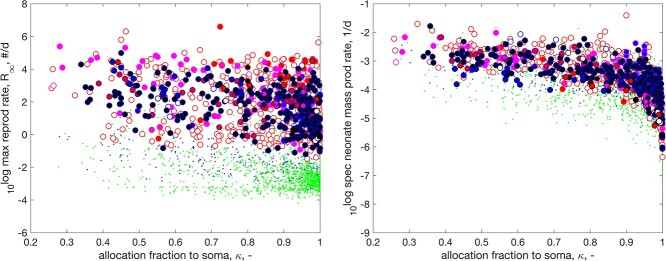
The maximum reproduction rate (left) and the weight-specific neonate mass production rate for a fully grown adult (right) as functions of the allocation fraction to soma for Actinopterygii in the AmP collection. Markers like in Fig. 4.

Figure 6 shows that reproductive investment tends to decrease for increasing $\kappa $, and that most species differ in the size of neonates, rather than in reproductive investment. Where fish reproduce much faster than tetrapods in number of offspring per time, this difference is mostly gone when expressed in mass per time.

**Fig. 7:**
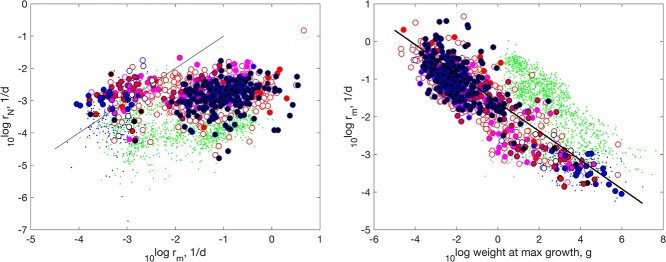
Left: specific population growth rate with thinning as function of specific body growth at maximum growth. The line indicates equal rates. Right: specific body growth at maximum growth as function of weight at maximum growth. The line has slope -0.38. Markers like in Fig. 4.

#### 3.1.4 Population and body growth

Thinning is defined as an age-dependent hazard rate such that the feeding rate of a cohort of neonates does not change under constant environmental conditions: the increase of the feeding rate of individuals due to growth, is exactly balanced by a reduction in numbers of individuals in the cohort (Kooijman *et al.*, 2020). The specific population growth rate under thinning was found to be more or less equal to the specific body growth rate at maximum growth for many animal species, suggesting an evolutionary relict from times when organisms were dividing unicellulars and a direct relationship between these quantities existed (Kooijman *et al.*, 2020). Figure 7 shows, however, that this holds for the early branching Cladistii, Chondrostei, Protacanthopterygii and Stomiati, but the specific body growth rate at maximum growth exceeds the specific population growth rate for other later branching actinopterygians. It is remarkable that none of these early branching taxa accelerate their metabolism.

Figure 1j shows that the median specific body growth at maximum growth of actinopterygians exceeds that of tetrapods. Figure 7b shows that the main reason is that actinopterygians reach that maximum at a smaller body weight, but for the same body weight tetrapods have a higher growth rate.

#### 3.1.5 Multi-dimensional scaling

**Fig. 8:**
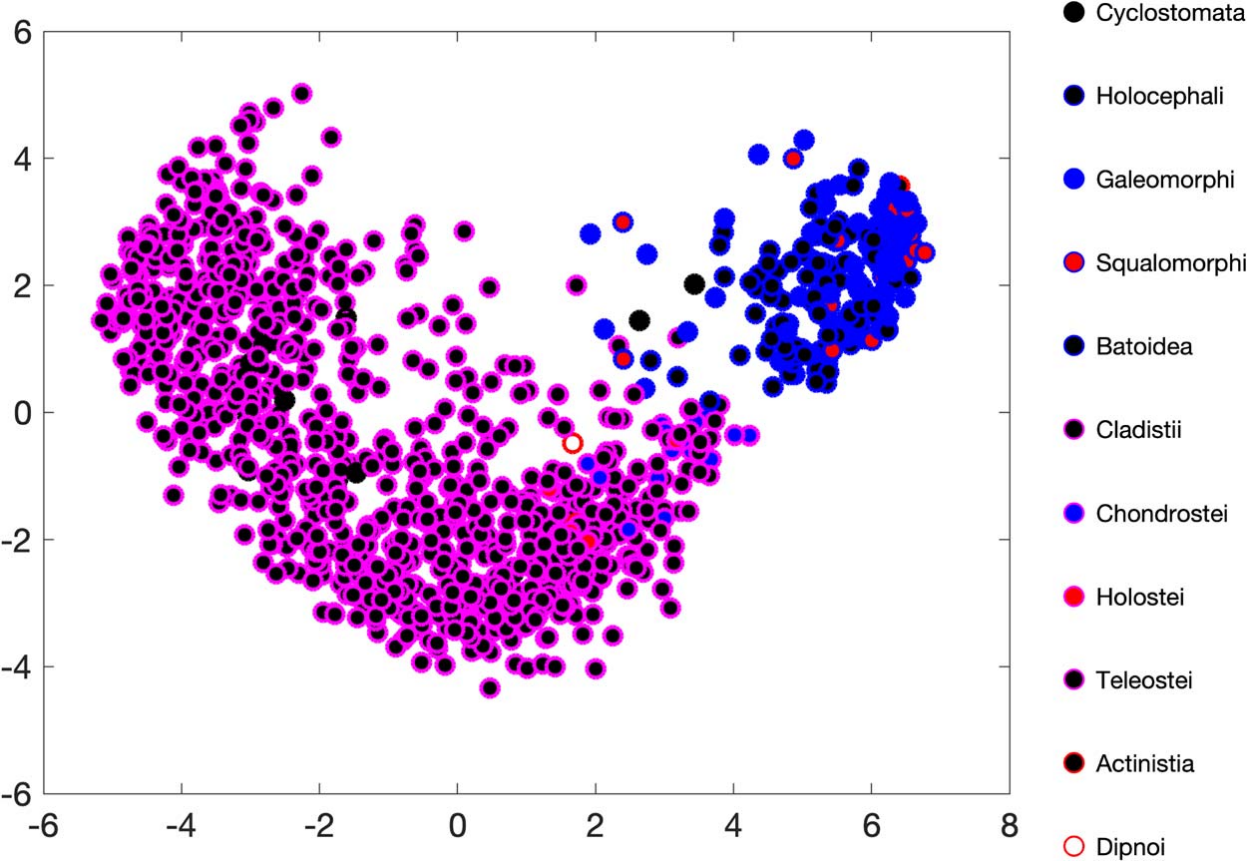
Classic multidimensional scaling for Pisces, using 15 traits: life span $a_m$, age at puberty $a_p$, age at birth $a_b$, ultimate structural length $L_\infty $, structural length at puberty $L_p$, structural length at birth $L_b$, max reserve capacity $[E_m]$, ultimate reproduction rate $R_\infty $, supply stress $s_s$, precociality index $s_H^{bp}$, specific somatic maintenance $[p_M]$, energy conductance $\dot {v}$, allocation fraction to soma $\kappa $, maturity at birth $E_H^b$ and maturity at puberty $E_H^p$.

The classical multi-dimensional scaling result for the fish (Pisces), using 15 traits, clearly separates the Chondrichthyes from the Actinopterygii; see Fig. 8. The coelacanth combines with chondrichthyes, the longfishes and the cyclostomates with the Actinopterygii. The chondrostei are, within the Actinopterygii, close to the Chondrichthyes. Correlations between first eigenvector and traits showed that the length at birth, ultimate length, life span and supply-stress play the most important roles in this result.

### 3.2 Extreme forms of waste-to-hurry

Waste-to-hurry is the co-variation pattern in parameter values across species where specific assimilation and somatic maintenance are increased simultaneously, which has the effect that growth and reproduction are boosted. Typical forms of waste-to-hurry couple this to a short life span as response to strong seasonal forcing of food availability. Key to the argument is the direct link between somatic maintenance and ultimate body size, which is why we first make some general comments. DEB theory takes parameter values to be individual-specific, but variations between individuals are typically small relative to the inter-specific ones, so that it still makes sense to talk about typical parameter values for a species as a kind of mean among individuals. Different populations can sport larger differences. Parameter values have a genetic as well as phenotypic component and external factors, such as temperature and toxic compounds, can affect particular parameter values. The effect of hybridization on parameter values is studied in Lika *et al.* (2020) for the catfishes *Clarias gariepinus* and *Heterobranchus longifilis*. The response of the Atlantic molly *Poecilia mexicana* to hydrogen sulphide, by reducing body size in combination with respiration (Passow *et al.*, 2017), points, in a DEB context, to a reducing effect of hydrogen sulphide on specific assimilation. Assimilation overheads contribute to respiration. A reduction in respiration here combines with a reduction in ultimate body size, while in waste-to-hurry the reverse happens. The reason why an increase in somatic maintenance reduces body size in DEB theory, is because it directly competes with growth as implication of the $\kappa $-rule.

**Fig. 9:**
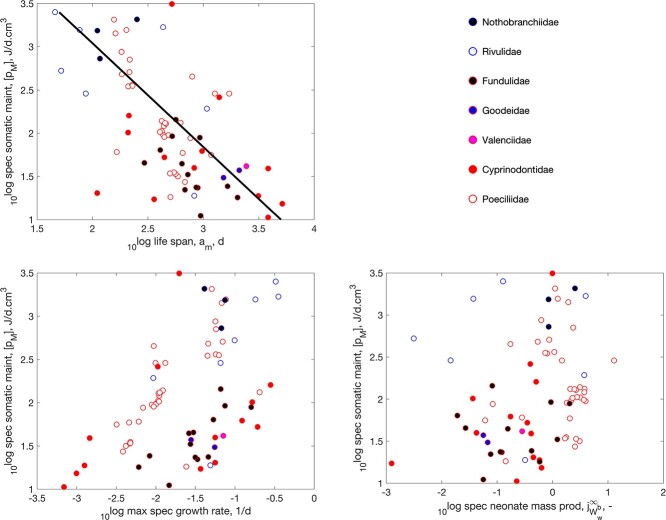
The waste-to-hurry strategy illustrated for the toothcarps Cyprinodontiformes in the AmP collection. The annual killifish belong to this taxon and are most extreme: a very short life span, coupled to the very high specific somatic maintenance. The line has slope -1.2. The specific neonate mass production rate is the life-time cumulated egg production times the neonate mass divided by the ultimate weight. Data corrected for a body temperature of 20 $^\circ $C.

**Fig. 10:**
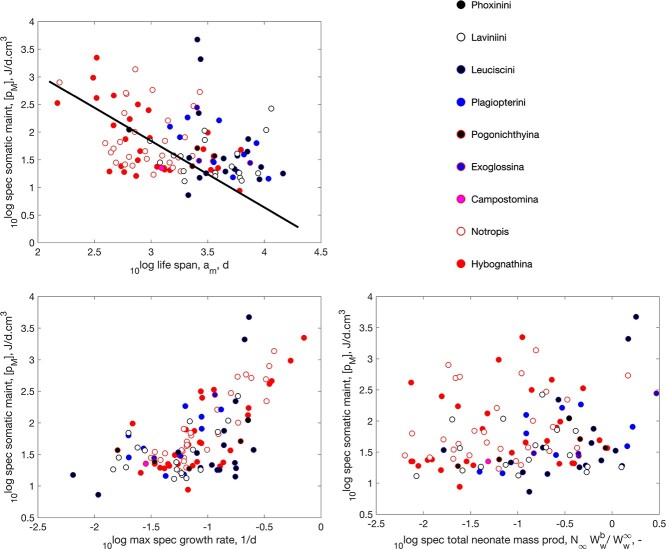
Similar to Fig. 9, but now for the minnows Leuciscinae in the AmP collection.

**Fig. 11:**
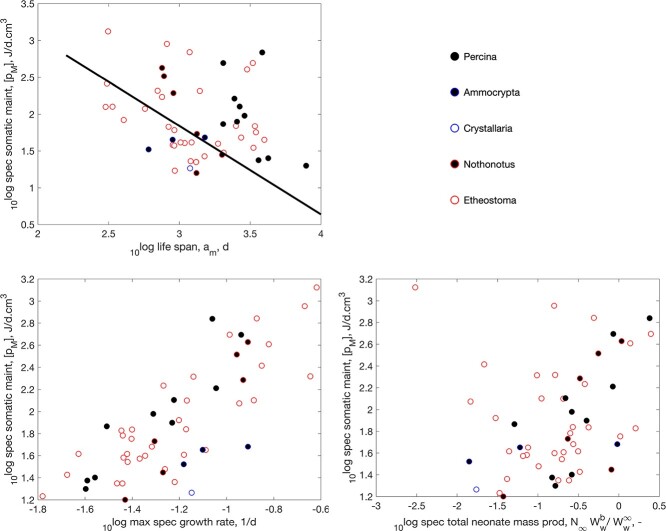
Similar to Fig. 9, but now for the darters Etheostomatinae in the AmP collection.

Having to deal with a short favorable season in terms of temperature and food availability, quite a few ray-finned fish sport the waste-to-hurry strategy. They typically avoid the absence of food by timing their embryo-phase in this period. Annual killifish (Cyprinodontiformes) from tropical savannas even have to deal with the additional problem of lack of water during the dry season, where eggs survive in moist mud. This is why they sport this strategy into the extreme and mature in a few weeks (Cui *et al.*, 2019), as illustrated in Fig. 9. They have the name of having the shortest adult life span among vertebrates (Valenzano *et al.*, 2015, Vrtílek *et al.*, 2018), but the Madagascan Labords chameleon (*Furcifer labordi*) also rivals this position (Cui *et al.*, 2019). A little less extreme, waste-to-hurry clearly occurs in the small minnows (Leuciscinae) and darters (Etheostomatinae) that mainly live in continental climates of North America; see Figs 10 and 11.

The boosting effect of waste-to-hurry on growth is clearly visible in the Figs 9–11. That the increase in both specific assimilation and somatic maintenance boosts growth is no surprise, since the von Bertalanffy growth rate, which is a statistic in the DEB context that also depends on food availability, is proportional to specific somatic maintenance, although the effect is partly counteracted by the increase in reserve capacity, which slows down growth (the reserve capacity is the ratio of specific assimilation and the energy conductance). We expected that the reducing effect of an increase reserve capacity was avoided by an increase of the energy conductance together with assimilation, since it controls reserve mobilization, but we could not find such a co-variation.

That waste-to-hurry generally also boosts reproduction in a DEB context is via the increase in specific assimilation, since a fixed fraction of assimilation is, via the reserve pool, allocated to maturity maintenance plus reproduction. However, assimilation is taken proportional to squared length, and the increase in specific somatic maintenance reduces size that counteracts the increase in assimilation to the extend that this can obscure the boosting effect on reproduction. Figures 9–11 show that the overall effect on reproduction is not that clear for these three taxa (Cyprinodontiformes, Leuciscinae and Etheostomatinae). For many other taxa, we did find clear boosting effects (see pattern-page of the AmP website).

Figures 9–11 clearly show the reducing effect of an increase in specific maintenance on life span. The lines in the figures not only have the same slope -1.2, but also the same intercept, so, in combination, they represent the same single line. We could not find a relationship between specific somatic maintenance and the Weibull aging acceleration. So the effect of specific somatic maintenance on life span does non involve the ageing process as such, but works via an increase of respiration.

## 4 Discussion

When the AmP collection had much less species in 2014, we reported a covariation of maximum reserve capacity $[E_m]$ and maximum structural length $L_m$ (Kooijman & Lika, 2014b) for non-accelerating actinopterygians, but not for accelerating ones. Now, with many more species, this relationship is no longer clear. The difference is mainly due to the many small short-lived American freshwater species that have been added since then, which follow the waste-to-hurry strategy.

The reserve capacity is not only of ecological importance via the ability to survive starvation periods and the mass-specific maintenance (since reserve does not need maintenance, but contributes to weight), but also for the kinetics of chemical compounds: both reserve and structure consist of a mixture of carbohydrates, proteins and lipids, while reserve is relatively rich in lipids in fish; pesticides are lipophyllic, for example.

The reason we found co-variation of $[E_m]$ and $L_m$ in the chondrichthyans, but not in the actinopterygians, is that the chondrichthyans hardly show the waste-to-hurry strategy and have a low somatic maintenance close to the one that AmP uses for the ‘generalized animal’; see Fig. 1d and Augustine *et al.* (2022).

The lack of covariation between $[E_m]$ and $L_m$, in combination with the large variation in $[\dot {p}_M]$, was reason for Maury *et al.* (2019) to propose modifications of the standard DEB model and to introduce an extra maintenance parameter. We do not think that deviations from expected co-variation of parameter values among species are sound arguments to modify ideas on the metabolic organization within an individual which are based on mechanisms in DEB theory; the lack of co-variation also does not apply to all taxa (e.g. chondrichthyans). Moreover, the extra maintenance parameter makes it impossible to estimate the other parameters in practice, especially $\kappa $, since reproductive investment relative to growth no longer provides information about this parameter with their modification. The fraction $\kappa $ equals the ratio between somatic maintenance and assimilation in fully grown individuals (Lika *et al.*, 2019), which can both be measured directly. But with the changes proposed by Maury *et al.* (2019), however, this is no longer the case. Indeed, the parameters of the modified model were never estimated from data. Last, but not least, their complaint that $[\dot {p}_M]$ varies that much is not solved by the introduction of a new parameter. Variation of parameters among species is key to biodiversity and adaptation.

In this paper, we explained why the lack of co-variation between $[E_m]$ and $L_m$ for the actinopterygians is due to interference with the waste-to-hurry pattern. The physical co-variation rules were meant to explain basic patterns of co-variation, but never to deny the importance of eco-evolutionary adaptations or biodiversity; the condition of the physical co-variation rules that all intensive parameters are the same for all species is meant as a ‘what if’ condition, not as a realistic assumption that would exclude all adaptation. On the contrary, by describing all species with the same model and accounting for the obvious physical constraints, the remaining differences between species point more clearly to eco-evolutionary adaptations.

We see the link between the specific somatic maintenance and the sensitivity for pesticides (Baas & Kooijman, 2015), i.e. human-made chemicals developed to kill crop grazers, as strong and independent support for the waste-to-hurry pattern, since crop technology creates exactly the conditions that select for the waste-to-hurry strategy (Augustine *et al.*, 2019b, Kooijman, 2013). We found, apart from waste-to-hurry, three more adaptation-based patterns that cause deviations from the condition that all species would have the same values for intensive parameters: acceleration (Kooijman, 2014), supply–demand spectra (Lika *et al.*, 2014a) and altricial-precocial spectra (Augustine *et al.*, 2019a).

In 2014 we reported that the allocation to development and reproduction was larger (= small $\kappa $) in chondrichthyans and actinopterygians, compared with other species. This pattern is again confirmed, but we now also found that chondrichthyans and cyclostomes allocate more to reproduction than actinopterygians and the CL-group; actinopterygians allocate only slightly more than tetrapods. As a consequence, the specific growth rate at maximum growth of actinopterygians and tetrapods is much higher than of the other three taxa, and the actinopterygians even higher than the tetrapods, since they reach this maximum at a smaller body size.

The pattern in the precociality coefficients can also be confirmed: the actinopterygians and the CL-group have a low one, the cyclostomes an intermediate one and the chondrichthyans a very large one, even larger than the tetrapods.

While the cyclostomes, the CL-group and the actinopterygians show a low supply stress, the chondrichthyans and the tetrapods have a much higher one, making them demand-species, with the birds and mammals as the most extreme taxa in this respect.

Our overall conclusion is that the many species that are presently included in the AmP collection facilitates recognition of adaptation patterns. To what extend measured data determines parameters is always an issue, and a large scatter in a few points can easily be misleading for pattern detection. A large number of species reduces that problem somewhat. This notion motivated us to develop methods for parameter estimation in context and the present findings provide further support for the taxonomic signal in parameter values.

## Supplementary Material

suppl_data_coac039
