# Supplementary material for: The comparative energetics of the ray-finned fish in an evolutionary context
Source: Conserv Physiol. 2022 Jul 5;10(1):coac039. doi: 10.1093/conphys/coac039 (PMC9258789; doi:10.1093/conphys/coac039)
Supplement: suppl_data_coac039 [file suppl_data_coac039.zip › LikaAugu2022_SM_A.pdf]

Supporting material for "The comparative energetics of the ray-finned fish in an evolutionary context" *

Konstadia Lika[1*], Starrlight Augustine[2] and Sebastiaan A.L.M. Kooijman[3]

[1]Department of Biology, University of Crete, 70013, Heraklion, Greece

[2]Akvaplan-niva, Fram High North Research Centre for Climate and the Environment, 9296 Tromsø, Norway

[3]Department of Theoretical Biology, VU University Amsterdam, The Netherlands

* Corresponding author: lika@uoc.gr

[1]Department of Biology, University of Crete, 70013, Heraklion, Greece

[2]Akvaplan-niva, Fram High North Research Centre for Climate and the Environment, 9296 Tromsø, Norway

[3]Department of Theoretical Biology, VU University Amsterdam, The Netherlands

* Corresponding author: lika@uoc.gr

In this Appendix we present the standard DEB model and then link the state variables and parameters with certain traits. For the traits that analytical formulae is either complex or not available we give the DEBtool functions used to compute them. DEBtool (DEBtool, 2022) is a free software package written in Matlab, the Mathworks©, and it was used to estimate parameters as well as compute all of the implied traits (Table 2, main text). The DEBtool package is freely downloadable from `https://github.com/add-my-pet/DEBtool_M`. DEBtool functions are marked in magenta throughout this appendix.

*Submitted to Conservation Physiology

A.1 The DEB model

The Dynamic Energy Budget (DEB) theory is about the metabolic dynamics of an individual organism through its entire life cycle. The state of an individual is described by four state variables: reserve energy, E, structural length, L, cumulated energy investment into maturation, E_H, and cumulated energy investment into reproduction, E_R. The assumptions that state how the state variable change are described in (Kooijman, 2012) or for a more physical setting to (Jusup et al., 2017). Briefly, an organism converts food to reserves via the process called assimilation. Subsequently, it allocates mobilized reserve to somatic and maturity maintenance, growth (i.e., increase in structural body mass) and maturation or reproduction (for adults). A fixed fraction κ of the mobilized energy is allocated to somatic functions (somatic maintenance and growth) and the remaining fraction to maturity maintenance and maturation or reproduction. Table A.1 summarizes the metabolic processes and the dynamics of the state variables and Table A.2 lists the DEB parameters needed for this study.

The standard (std) DEB model includes three life stages (embryos, juveniles and adults) and assumes isomorphic growth over all life stages. Isomorphy implies that surface area is proportional to structural length squared. Many fish species show metabolic acceleration during the early developmental stages. When acceleration occurs between birth and metamorphosis the model is named "abj". In terms of modeling, during metabolic acceleration the surface area grows proportionally to structural volume (L^3), with the consequence that the two parameters $\{\dot{p}_{Am}\}$ and $\dot{v}$ (Table A.2) with surface area in the dimensions are no longer constant but increase proportional to structural length. Acceleration is quantified by the acceleration factor $s_{\mathcal{M}} = \max\left(L_b, \min(L, L_j)\right)/L_b$, where L_b and L_j are the structural lengths at birth and metamorphosis respectively. After acceleration these parameters are multiplied by the acceleration factor $s_{\mathcal{M}} = \frac{L_j}{L_b}$.

The effects of temperature on any rate parameter $\dot{k}$ is computed using the Arrhenius equation

$$\dot{k}(T) = \dot{k}(T_{ref}) \exp\left(\frac{T_A}{T_{ref}} - \frac{T_A}{T}\right) \tag{A.1}$$

A.1.1 Computing traits

In the following subsections and in Table A.3 we present the analytical formulae for the traits and the DEBtool functions used to compute them.

Table A.1: Energy fluxes linked to metabolic processes, state variables, and dynamics of the standard DEB model. Square brackets [ ] indicate quantities expressed per unit of structural volume and curly brackets { } per unit of structural surface area. The logical (boolean) operation enclosed in parentheses, e.g. $(x \geq y)$, have value 1 if true and value 0 if false.

**Metabolic process**	**Energy fluxes**
Assimilation	$\dot{p}_A = \{\dot{p}_{Am}\} f L^2 (E_H \geq E_H^b)$
Mobilization	$\dot{p}_C = E \frac{\dot{v}[E_G]L^2 + \dot{p}_S}{[E_G]L^3 + \kappa E}$
Somatic maintenance	$\dot{p}_S = [\dot{p}_M]L^3 + \{\dot{p}_T\}L^2$
Maturity maintenance	$\dot{p}_J = \dot{k}_J \max(E_H, E_H^p)$
Growth	$\dot{p}_G = \kappa \dot{p}_C - \dot{p}_S$
Maturation/reproduction	$\dot{p}_R = (1-\kappa)\dot{p}_C - \dot{p}_J$
**State variables**	
E	Reserve energy
L	Structural length
E_H	Cumulated energy investment into maturation
E_R	Cumulated energy investment to reproduction
**Dynamics**	
$\frac{d}{dt}L^3 = \frac{\dot{p}_G}{[E_G]}$	
$\frac{d}{dt}E = \dot{p}_A - \dot{p}_C$	
$\frac{d}{dt}E_H = \dot{p}_R(E_H < E_H^p)$	
$\frac{d}{dt}E_R = \kappa_R \dot{p}_R (E_H \geq E_H^p)$	

Table A.2: DEB parameters. Notation: square brackets, [ ], indicate parameters expressed per unit of structural volume, and curly brackets, { }, per unit of structural surface area.

Symbol	Units	Interpretation
$\{\dot{p}_{Am}\}$	$\mathrm{J\,d^{-1}\,cm^{-2}}$	Maximum specific assimilation rate (assimilation)
$\dot{v}$	$\mathrm{cm\,d^{-1}}$	Energy conductance (mobilization)
κ	–	Allocation fraction to soma (allocation)
$[\dot{p}_M]$	$\mathrm{J\,d^{-1}\,cm^{-3}}$	Somatic (volume specific) maintenance rate (turnover, activity)
$\{\dot{p}_T\}$	$\mathrm{J\,d^{-1}\,cm^{-3}}$	Somatic (surface specific) maintenance rate (heating, osmosis)
$\dot{k}_J$	$\mathrm{d^{-1}}$	Maturity maintenance rate coefficient (development)
$[E_G]$	$\mathrm{J\,cm^{-3}}$	Specific cost for structure (growth)
E_H^b	J	Maturity at birth (life cycle)
E_H^j	J	Maturity at metamorphosis (life cycle)
E_H^p	J	Maturity at puberty (life cycle)
$\ddot{h}_a$	$\mathrm{d^{-2}}$	Weibull aging acceleration (aging)
s_G	–	Gompertz stress coefficient (aging)
$[E_m] = \frac{\{\dot{p}_{Am}\}}{\dot{v}}$	$\mathrm{J\,cm^{-3}}$	Maximum energy density
$g = \frac{[E_G]}{\kappa [E_m]}$	–	Energy investment ratio
$L_m = \frac{\kappa \{\dot{p}_{Am}\}}{[\dot{p}_M]}$	cm	maximum volumetric length
$\overline{\mu}_E$	$\mathrm{J\ mol^{-1}}$	chemical potential of reserve
d_V	$\mathrm{g\ cm^{-3}}$	specific density of structure
$w = \frac{[E_m]\, w_E}{\overline{\mu}_E\, d_V}$	–	contribution of reserve to ash free dry mass
$\dot{k}_M = \frac{[\dot{p}_M]}{[E_G]}$	$\mathrm{d^{-1}}$	somatic maintenance rate coefficient
$k = \frac{\dot{k}_J}{\dot{k}_M}$	–	maintenance ratio
f	–	functional response
T_A	K	Arrhenius temperature
T_{ref}	K	Reference temperature

Respiration

The metabolism of the individual is conceptualized as a single macro-chemical reaction equation:

- food + O_2 → reserve + structure + faeces + H_2O + CO_2 + NH_3 + heat

It is possible to split this single macro-chemical reaction equation into three "micro-chemical reaction equations":

1. assimilation: food + O_2 → reserve + faeces + H_2O + CO_2 + NH_3 + heat
2. dissipation: reserve + O_2 → H_2O + CO_2 + NH_3 + heat
3. growth: reserve + O_2 → structure + H_2O + CO_2 + NH_3 + heat

These equations have to obey conservation rules for energy and the four chemical elements C, H, O and N.

It turns out that the mineral fluxes $\dot{J}_C$ (mol CO_2 day^{-1}), $\dot{J}_H$ (mol H_2O day^{-1}), $\dot{J}_O$ (mol O_2 day^{-1}), and $\dot{J}_N$ (mol NH_3 day^{-1}) are weighted sums of the three basic powers, assimilation, $\dot{p}_A$, dissipation, $\dot{p}_D = \dot{p}_S + \dot{p}_J + (1 - \kappa_R)\dot{p}_R$ (where $\kappa_R = 0$ for embryos and juveniles) and growth, $\dot{p}_G$, defined in Table A.1. Thus, the dioxygen consumption rate $\dot{J}_O$ (mol/d) can be written as

$$\dot{J}_O = \eta_{OA}\,\dot{p}_A + \eta_{OD}\,\dot{p}_D + \eta_{OG}\,\dot{p}_G \tag{A.2}$$

The three basic powers are cubic polynomials in structural length (Kooijman, 2010, Chapter 2, Table 2.5). The coefficients $\eta_{O\star}$ specify the mass flux of dioxygen per unit of power $\star$ (i.e., the coupling between the mass and energy fluxes) and are combinations of DEB parameters (Kooijman, 2010, Chapter 4.3).

The ultimate dioxygen consumption rate $\dot{J}_O^\infty$ (i.e., the oxygen consumption of a fully grown individual) is computed with the DEBtool function scaled_power (std-DEB model) or scaled_power_j (abj-DEB model) at ultimate structural length $L_\infty = s_\mathcal{M} f L_m$ and abundant food $f = 1$.

The **ultimate specific dioxygen consumption rate** $\dot{j}_O^\infty$, which is used as a trait in the main text, is the ratio of the dioxygen consumption rate and wet weight at ultimate size:

$$\dot{j}_O^\infty = \frac{\dot{J}_O^\infty}{W_w^\infty} \tag{A.3}$$

where $W_w^\infty = L_\infty^3(1 + f\omega)$ at abundant food $f = 1$.

Aging

In addition to the four state variables discussed above, the aging module of DEB theory requires two additional state variable: the damage inducing compounds and the damage compounds, the dynamics of which are linked to the energy mobilization rate $\dot{p}_C$ and the specific growth rate $\dot{r} = \frac{1}{V}\frac{dV}{dt}$. The aging module has two aging parameters, the Weibull aging acceleration $\ddot{h}_a$ and the Gompertz stress coefficient s_G. The mean life span is computed with the DEBtool function get_tm_mod.

Reproduction

When maturity, E_H, reaches the threshold value E_H^p, maturation ceases and energy is allocated to reproduction at a rate

$$\dot{p}_R = (1 - \kappa)\dot{p}_C - \dot{k}_J E_H^p \tag{A.4}$$

The mean reproduction rate in terms of number of eggs per time equals $\dot{R} = \kappa_R \dot{p}_C / E_0$, where E_0 is the initial energy in egg (Table A.3).

At constant food density, where reserve density $[E] = E/V$ equals $f[E_m]$, the maximum (mean) reproduction rate for an individual of maximum size, i.e., $L = L_\infty$ and W_w^∞, equals

$$\dot{R}_\infty = \kappa_R \dot{k}_M \frac{1 - k v_H^p}{v_E^0} \tag{A.5}$$

with $v_E^0 = \frac{E_0}{(1-\kappa)g[E_m]L_m^3}$ and $v_E^p = \frac{E_H^p}{(1-\kappa)g[E_m]L_m^3}$.

The maximum (mean) reproduction rate is computed with the DEBtool functions reprod_rate (std-DEB model) or reprod_rate_j (abj-DEB model) at ultimate structural length L_∞ and abundant food $f = 1$.

The ultimate neonate mass production rate is obtained by multiplying the maximum reproduction rate $\dot{R}_\infty$ (eq. A.5) and the wet weight of a neonate W_w^b (Table A.3), then dividing by the ultimate weight of the mother we obtain the **ultimate weight-specific neonate mass production rate**:

$$\dot{j}_{W_w^b}^\infty = \frac{\dot{R}_\infty W_w^b}{W_w^\infty} \tag{A.6}$$

The **life time neonate mass** is the product of the life time reproductive output N_∞ and the neonate wet weight W_w^b (Table A.3).

Population and body growth

In a constant environment with only aging as cause of death, any population would grow theoretically exponentially. To avoid enormous population growth rates, we specify a hazard rate called "thinning": the hazard rate is chosen such that the feeding rate of a cohort of neonates does not change in time. When individuals grow, they eat more, but this effect is exactly balanced by a reduction in numbers. The **specific population growth rate** $\dot{r}_N$ can be computed by solving the characteristic equation (see for details Kooijman et al. (2020)). The computation of the population growth rate has been done for all species of the AmP collection with the function AmPtool/curation/prt_my_pet_pop of AmPtool (AmPtool, 2022).

The text presents results in terms of weight-specific body (structure + reserve) growth rate at maximum growth and maximum specific growth rate of structure. We here work out their relationship after birth.

The change in structural volume, for the standard DEB models, at abundant food ($f = 1$) is given by

$$\frac{d}{dt}L^3 = \frac{\dot{p}_G}{[E_G]} = \dot{r}L^3 \tag{A.7}$$

with

$$\dot{r} = \dot{v}\frac{1/L - 1/L_m}{1+g} = \frac{\dot{k}_M(L_m/L - 1)}{1 + 1/g} \quad \text{the specific growth rate of structure} \tag{A.8}$$

Alternatively, the change in structural length is

$$\frac{d}{dt}L = \dot{r}_B(L_m - L) \tag{A.9}$$

with

$$\dot{r}_B = \frac{\dot{k}_M/3}{1 + 1/g} = \frac{\dot{r}/3}{L_m/L - 1} \quad \text{the von Bertalanffy growth rate} \tag{A.10}$$

Growth of structure is at maximum when $\frac{d}{dt}(\dot{r}L^3) = 0$ and occurs at structural length $L = \frac{2}{3}L_m$ (Kooijman et al., 2020) and the specific growth rate at this length is $\dot{r}_m = \frac{\dot{k}_M/2}{1+1/g}$. The implication is that the specific growth at maximum growth, $\dot{r}_m$, relates to the von Bertalanffy growth rate, $\dot{r}_B$: $\dot{r}_m = \frac{3}{2}\dot{r}_B$.

The **specific body growth rate at maximum growth** $\dot{r}_m$ is computed for all models with the DEBtool functions statistics_st.

Table A.3: DEB traits and other quantities.

Symbol	Units	Interpretation	
E_0	J	initial energy in egg	initial_scaled_reserve
a_b, a_p	d	age at birth, puberty	get_tj
a_m	d	age at death	get_tm_mod
L_b, L_p	cm	structural length at birth, puberty	get_tj
L_∞	cm	ultimate volumetric length	$s_\mathcal{M} f L_m$
W_w^b	g	wet weight at birth	$L_b^3(1 + f\omega)$
W_w^p	g	wet weight puberty	$L_p^3(1 + f\omega)$
W_w^∞	g	ultimate wet weight	$L_\infty^3(1 + f\omega)$
N_∞	#	life time reproductive output	cum_reprod (std-DEB) or
			cum_reprod_j (abj-DEB)

References

AmPtool (2022). Software package AmPtool. https://github.com/add-my-pet/AmPtool.

DEBtool (2022). Software package debtool. https://github.com/add-my-pet/DEBtool$_M$.

Jusup, M., Sousa, T., Domingos, T., Velimir, L., Marn, N., Wand, Z., and Klanjšček, T. (2017). Physics of metabolic organization. Journal of Sea Research, 94:144–155.

Kooijman, S. (2012). Energy budgets. In Hastings, A., Gross, L., and eds., editors, Encyclopedia of Theoretical Ecology. University of California Press.

Kooijman, S. A. L. M. (2010). Dynamic Energy Budget theory for metabolic organisation. Cambridge University Press.

Kooijman, S. A. L. M., Lika, K., Augustine, S., and Kooi, B. W. (2020). The energetic basis of population growth in animal kingdom. Ecol. Mod., 428:109055.
