# Supplementary material for: The comparative energetics of the ray-finned fish in an evolutionary context
Source: Conserv Physiol. 2022 Jul 5;10(1):coac039. doi: 10.1093/conphys/coac039 (PMC9258789; doi:10.1093/conphys/coac039)
Supplement: suppl_data_coac039 [file suppl_data_coac039.zip › LikaAugu2022_SM_B.pdf]

Supporting material for
The comparative energetics of the ray-finned fish in an evolutionary context *

Konstadia Lika[1*], Starrlight Augustine[2] and Sebastiaan A.L.M. Kooijman[3]

[1]Department of Biology, University of Crete, 70013, Heraklion, Greece

[2]Akvaplan-niva, Fram High North Research Centre for Climate and the Environment, 9296 Tromsø, Norway

[3]Department of Theoretical Biology, VU University Amsterdam, The Netherlands

* Corresponding author: lika@uoc.gr

B.1 List of species and data types

In this Appendix we give the list of species (Table B.1) with the data types (Table B.2) that are used in the analysis and references.

*Submitted to Cons. Physiol.

Table B.1: Ray-finned fish species that are included in the AmP collection at 2022/04/11, the data types . as extracted from the literature and selected references. Besides these references, websites for have used to get data, which are presented on the AmP website. The codes of the data types are presented in Table B.3

species	data	references
Erpetoichthys calabaricus	ah, ab, ap, am, Lb, Lp, Li, L_t, Wwp, Wwi, Ri	Babatunde (2011)
Polypterus senegalus	ah_T, am, Lp, Li, Wwb, Wwi, Ri, t-L	Bartsch et al. (1997)
Polyodon spathula	ap, am, Li, Wwb, Wwp, Wwi, Ri, t-L, t-Ww, L-Ww	Rosen (1976)
Acipenser brevirostrum	am, Lp, Li, Wwb, Wwi, Ri, t-L, t-Ww, T-ab	Hardy and Litvak (2004), Woodland (2005)
Acipenser fulvescens	ab, ap, am, Lp, Li, Wwb, Ri, t-L, L-Ww	Bruch et al. (2006), Haugen (1969)
Acipenser gueldenstaedtii	ah, ab, ap, am, Lb, Lp, Li, Wwh, Wwi, Ri, t-Ww	Chebanov and Galich (2013), Coad (2018), J (1993), Memis et al. (2009), Nazari et al. (2013), Siddique et al. (2014)
Acipenser medirostris	ab, am, Lp, Li, Wwb, Wwi, Ri, t-Ww	Moser et al. (2016)
Acipenser nudiventris	ah, ab, ap, am, Lh, Lb, Li, Wwi, Ri, t-Ww	Chebanov and Galich (2013), Coad (2018), fisheries organization (2017), Kottelat and Freyhof (2007), Mousavi and Ghafor (2014), Rochard et al. (1991), Siddique et al. (2014)
Acipenser oxyrinchus	ab, ap, am, Li, Wwb, Wwi, Ri, t-L, t-Ww, L-Ww	Mason et al. (1992), Mims et al. (2002)
Acipenser persicus	ah, ab, ap, am, ah, Lb, Lp, Li, Wwp, Wwi, Ri, t-L	Alavi et al. (2005), Coad (2018), Imani and Falahatkar (2017), Kottelat and Freyhof (2007), Nazari et al. (2013), Siddique et al. (2014), Vecsei and Artyukhin (2001)
Acipenser ruthenus	ab, ap, am, Lb, Lp, Li, Wwb, Wwp, Wwi, Ri	Peterson et al. (2006), Pikitch et al. (2005), Reinartz (2002), Rochard et al. (1991)
Acipenser stellatus	ah, ab, ap, am, Lb, Lp, Li, Wwh, Wwi, Ri, t-Ww	Chebanov and Galich (2013), Coad (2018), Frimodt (1995), Ghasemi et al. (2018), J (1993), Kottelat and Freyhof (2007), Mousavi and Ghafor (2014), Siddique et al. (2014)
Acipenser sturio	ah, ab, aj, ap, am, Lh, Lb, Lj, Lp, Li, Wwi, Ri	Bruch (2008), Dettlaff et al. (1993), Ehrenbaum (1894), Kinzelbach (1987), Williot et al. (2009)
Acipenser transmontanus	ah, ab, ap, am, Lb, Lp, Li, Wwb, Wwi, Ri, t-dWw	Cherr and Clark (1982), Lee et al. (2017), Parsley and Kofoot (2013)
Huso huso	ab, ap, am, Lb, Lp, Li, Wwb, Wwp, Wwi, Ri, t-L	Chebanov and Galich (2013), Coad (2018), Falahatkar et al. (2013), J (1993), Kottelat and Freyhof (2007), Motlagh (2001), Siddique et al. (2014)

Continuation of Table B.1		
**species**	**data**	**references**
Huso dauricus	am, Lp, Li, Wwb, Ri, t-L, L-Ww	Birstein et al. (2002), Koshelev et al. (2014)
Scaphirhynchus albus	ab, ap, am, Li, Wwb, Wwi, Ri, LWw, t-L, t-Ww	George et al. (2012), Keenlyne and Jenkins (1993), Keenlyne and Maxwell (1993)
Scaphirhynchus suttkusi	ab, ap, am, Lp, Li, Wwb, Wwi, Ri	George et al. (2012), Kuhajda and Rider (2016)
Scaphirhynchus platorynchus	ab, ap, am, Lp, Li, Wwb, Wwi, Ri, t-L	Helms (1974)
Amia calva	ah, ab, ap, am, Lh, Lb, L_t, Lp, Li, Wwi, Ri, t-L, L-Ww	Etnier and Starnes (1993)
Atractosteus spatula	ah, ab, ap, am, Lh, Lb, Li, Wwi, Ri, t-Ww	Clay (2004)
Lepisosteus oculatus	ab, ap, am, Lb, Li, Wwb, Ri, t-L, L-Ww	Love (2004), Wallus et al. (1990)
Lepisosteus osseus	ap, am, Lp, Li, Wwb, Ri_L, t-L, L-Ww	Kelley (2012), Wallus et al. (1990)
Lepisosteus platostomus	ab, ap, am, Lp, Li, Wwb, Wwi, R_Ww, t-L	Sutton et al. (2009), Wallus et al. (1990)
Lepisosteus platyrhincus	ap, am, Lp, Li, Wwb, Wwi, R_L, t-L	Murie et al. (2009), Wallus et al. (1990)
Elops saurus	ab, am, Ls, Lp, Li, Wwb, Wwp, Wwi, Ri, dLb, L-Ww	Levesque (2014), Santos-Martínex and Ardboleda (1993)
Megalops atlanticus	ab, ap, am, Lb, Ls, Lj, L_t, Lp, Li, Wwi, Ri, t-L	Crabtree et al. (1995)
Albula vulpes	ab, as, aj, ap, am, Lb, Ls, Lj, Lp, Li, Wwi, Ri, L-Ww	Bruger (1974)
Notacanthus chemnitzii	aj, ap, am, Lj, Lp, Li, Wwb, Ri, t-L, L-Ww	Vedishcheva et al. (2016)
Anguilla anguilla	ab, ap, am, Lb, Lp, Li, Wwb, Wwp, Wwi, Ri, t-L, t-Ww, L-Ww	Angelidis et al. (2005), Penaz and Tesch (1970), Tesch (2003)
Anguilla japonica	am, Lp, Li, Wwb, Wwi, Ri, t-L, t-Ww	Castellani and Edwards (2017), Guan et al. (1994), H. Tanaka) (2001)
Conger conger	am, Lp, Li, Wwb, Wwp, Wwi, Ri, t-L, t-Ww	Cunningham (1891), O'Sullivan et al. (2003)
Hiodon tergisus	ap, am, Lb, L_t, Li, Wwi, Ri	
Hiodon alosoides	ap, am, Lp, Li, Wwb, Wwi, Ri, t-L_f, t-Ww_f	Donald and Kooyman (2011), Wallus et al. (1990)
Osteoglossum bicirrhosum	ah, ab, ap, am, Lh, Lb, Li, Wwi, Ri	Yanwirsal (2013)
Notopterus notopterus	ah, ab, ap, am, Lh, Lb, Lp, Li, Ww_L, Ri	Yanwirsal (2013)
Chitala chitala	ah, ab, am, Lb, Lp, Li, Wwb, t-L, L-Ww, Ww-N	Hussain et al. (2015), Sarkar et al. (2006, 2008)
Arapaima gigas	ab, ap, am, Lb, Lp, Li, Wwi, Ri, t-L, L-Ww	Arantes et al. (2010), Queiroz (2000)
Clupea harengus	ab, aj, ap, am, Lb, Lp, Li, Wwi, Ri, t-L, t-Ww_f, L-Ww	Bigelow et al. (1963), Blaxter (1968), G.H. and M. (2004), J. (2002), Koli (1990), Lea (1930), Teal (2003)
Clupea bentincki	ab, am, Lp, Li, Wwb, Wwi, Ri, t-L	Castillo-Jordán et al. (2010)
Clupea pallasii	ab, am, Lp, Li, Wwb, Wwi, Ri, t-L	Chernoivanova (2017)

Continuation of Table B.1		
**species**	**data**	**references**
Sprattus sprattus	am, Lb, Lp, Li, Ri, t-L, L-Ww, L-N	Dulcic (1998), Prodanov (1997), Torstensen (1992)
Clupeonella cultriventris	am, Lp, Li, Wwb, Ri, t-L_f, t-Ww_f	Kiyashko et al. (2006)
Alosa aestivalis	ap, am, Lp, Li, Wwb, Ri, t-L, L-Ww	Messieh (1977), Wallus et al. (1990)
Alosa alabamae	am, Lp, Li, Wwb, t-L, L-Ww, L-N	Ingram (2007)
Alosa alosa	ap, am, Li, Wwb, Wwi, Ri, t-L, t-Ww	de Laak (2009)
Alosa chrysochloris	ap, am, Lh, Lb, Lp, Li, Wwb, Wwi, Ri	Wallus et al. (1990)
Alosa mediocris	ah, am, Lp, Li, Wwb, Wwi, Ri, t-L	Mansueti (1962)
Alosa sapidissima	ah_T, ab, ap, am, Lh, Lb, Lp, Li, Wwi, Ri, t-L	Maltais et al. (2010), Savoy and Crecco (2004)
Alosa saposchnikowii	am, Lp, Li, Wwb, Wwi, Ri, t-L, t-Ww	Malkin and Andrianova (2008)
Alosa pseudoharengus	ab, ap, am, Lp, Li, Wwb, Wwi, Ri, t-L, L-Ww	Messieh (1977), Wallus et al. (1990)
Brevoortia patronus	ab, am, Lp, Li, Wwb, Wwp, Wwi, Ri, t-L	Hettler (1984), Warlen (1988)
Sardina pilchardus	ab, ap, am, Lb, Lp, Li, Wwb, Wwp, Wwi, Ri, t-L, L-Ww, t-Ww, t-GSI	Dessier (2015), ICES (2012), Nunes et al. (2011), Q. Queiros (2017), Ré and Meneses (2008), Silva et al. (2008, 2006)
Sardinops sagax	ab, ap, am, Lp, Li, Wwb, Wwi, Ri, t-L	nonez Velázquez et al. (2000)
Limnothrissa miodon	am, Lp, Li, Wwb, Wwp, Wwi, t-L, Ww-N	Kimura (1995), Peter (1999)
Stolothrissa tanganicae	am, Li, Wwb, Wwp, Wwi, t-L, Ww-N	Kimura (1995), Peter (1999)
Tenualosa ilisha	am, Lp, Li, Wwb, Wwp, Wwi, Ri, t-L	Almukhtar et al. (2016), Amin et al. (2005), Marammazi et al. (2016), Rahman and Cowx (2006)
Amblygaster sirm	ab, ap, am, Lp, Li, Wwb, Wwi, Ri, t-L	Dayaratne and Gjøsaeter (1986)
Dorosoma cepedianum	ab, am, Lp, Li, Wwb, Ri, t-L, t-Ww, L-Ww	Bodola (1964), Wallus et al. (1990)
Dorosoma petenense	am, Lp, Li, Wwb, Wwi, t-L, L-N	Johnson (1970, 1971)
Ethmalosa fimbriata	am, Lp, Li, Wwb, Wwp, Wwi, Ri, t-L	Moses (1988)
Herklotsichthys quadrimaculatus	am, Lp, Li, Wwb, Wwi, Ri, t-L_f	Milton et al. (1993), Shirafuji et al. (2007)
Hilsa kelee	am, Lp, Li, Wwb, Wwi, Ri, t-L	Panhwar et al. (2013)
Opisthonema oglinum	am, Lp, Li, Wwb, Wwi, Ri, t-L	Vega-Cendejas et al. (1997)
Opisthonema libertate	am, Lp, Li, Wwb, Wwi, Ri, t-L	Ruiz-Domínguez and Quinonez-Velázquez (2018)
Sardinella aurita	ab, ap, am, Lb, Lp, Li, Wwb, Wwi, Ri, t-L, L-Ww, Ww-N	Houria (2015), Tsikliras and Antonopoulou (2006)
Sardinella albella	ab, ap, am, Lp, Li, Wwb, Wwi, Ri, t-L	Dayaratne and Gjøsaeter (1986)
Sardinella gibbosa	ab, ap, am, Lp, Li, Wwb, Wwi, Ri, t-L	Dayaratne and Gjøsaeter (1986)
Sardinella longiceps	ab, ap, am, Lp, Li, Wwb, Wwi, Ri, t-L	Dayaratne and Gjøsaeter (1986)
Engraulis encrasicolus	ab, aj, ap, am, Lb, Lj, Lp, Li, Wdb, Wdj, Wwp, Wwi, Ri, t-L, T-ah	Catalan et al. (2012), Garrido et al. (2012), Pecquerie (2008), Pecquerie et al. (2009), Ré (1996), Regner (1996)

Continuation of Table B.1		
**species**	**data**	**references**
Engraulis anchoita	ab, am, Lp, Li, Wwb, Wwi, Ri, t-L_f	Carvalho and Castello (2013), Castello and Catello (2003), de Ciechomski (2013)
Engraulis japonicus	ab, am, Lp, Li, Wwb, Wwi, Ri, t-L, L-Ww	Fukuhara (1983), Fukuhara and Takao (1988)
Anchoa mitchilli	ab, am, Lp, Li, Wwb, Wwp, Wwi, Ri, t-L	Acosta (2000)
Spratelloides delicatulus	am, Lp, Li, Wwb, Wwi, Ri, t-L	Milton et al. (1991), Shirafuji et al. (2007)
Spratelloides gracilis	am, Lp, Li, Wwb, Wwi, Ri, t-L_f	Milton et al. (1991), Shirafuji et al. (2007)
Spratelloides lewisi	am, Lp, Li, Wwb, Wwi, Ri, t-L	Milton et al. (1991), Shirafuji et al. (2007)
Chirocentrus dorab	ab, ap, am, Lb, Lp, Li, Wwi, Ri, t-L	Abdussamad et al. (2011), Delsman (1930), Luther (1985), Richards (2008)
Alepocephalus bairdii	am, Lp, Li, Wwb, Wwi, Ri, t-L	Allain and Lorance (2000)
Bajacalifornia burragei	am, Lp, Li, Wwb, Wwp, Wwi, Ri, t-L	Childress et al. (1980)
Chanos chanos	ah, ab, aj, ap, am, Lh, Lb, Lp, Li, Wwp, Wwi, Ri, t-L, t-Ww, L-N, Ww-N	Bagarinao (1991), Sumagaysay and Borlongan (1995)
Enteromius liberiensis	ab, ap, am, Lp, Li, Wwb, Wwp, Wwi, Ri, t-L	Payne (1976), Weimans (2007)
Cyprinus carpio	ah, ab, ap, am, Lh, Lb, Lp, Li, Wwb, Wwp, Wwi, GSI, t-L_f, t-Ww, L-Ww, L-N, Ww-N	Crivelli (1981), de Wilt and van Emmerik (2008), Donkers (2011), I. Balık et al. (2006), Karatas and Seze (2005), Njouokou et al. (2013)
Carassius auratus	ab, am, Li, Wwb, Wwp, Wwi, Ri, t-L	Lorenzoni et al. (2007), Ortega-Salas1 and Reyes-Bustamante (2006)
Carassius carassius	ap, am, Lh, Lb, Li, Wwb, Ri, t-L, L-Ww, T-ah	Wijmans (2009)
Carassius gibelio	am, Lp, Li, Wwb, Ri, t-L_f, L-Ww_f	Marinović et al. (2016)
Barbus barbus	ab, ap, am, Lp, Li, Wwb, Wwp, Wwi, t-L, t-Ww, L-N	Weimans (2007)
Capoeta capoeta	am, Lp, Li, Wwb, Ri, t-L, L-Ww	Türkmen et al. (2002)
Luciobarbus sclateri	ap, am, Lp, Li, Wwb, GSI, t-L, t-Ww	Herrera et al. (1988)
Schizothorax richardsonii	am, Lp, Li, Wwb, Wwi, Ri, t-L, t-Ww	Joshi et al. (2016), Singh and Sharrna (1995)
Danio rerio	ab, aj, ap, am, L0, Lb, Lj, Lp, Li, Wd0, Wwi, Ri, GSI, t-Le, t-Wwe, t-Wde, t-JCe, t-JNe, t-L_fT, t-Ww_f, t-N, t-S, L-Ww	Augustine (2009), Bagatto et al. (2001), Bang et al. (2004), Bayer (2005), Beaudouin et al. (2015), Best et al. (2010), Drew et al. (2008), Forbes et al. (2010), Geffroy and Simon (2013), Gerhard et al. (2002), Lawrence et al. (2008), Schilling (2002), Spence et al. (2008)
Hypophthalmichthys molitrix	am, Lp, Li, Wwb, Wwp, Wwi, t-L, t-Ww, L-Ww	Beelen (2008), Kamilov (2014), Lenaerts et al. (2015)
Hypophthalmichthys nobilis	am, Lp, Li, Wwb, Wwp, Wwi, Ri, t-L	Schranka and Guy (2002)

Continuation of Table B.1		
**species**	**data**	**references**
Ctenopharyngodon idella	ap, am, Lp, Li, Wwb, Wwp, Wwi, Ri, t-L, t-Ww	Venkatesh and Shetty (1978)
Tinca tinca	ah, ab, ap, am, Lh, Lb, Lp, Li, Wwb, Wwp, Wwi, t-L, L-N, Ww-N	Beelen (2008), Pompei et al. (2012)
Rhodeus amarus	ab, ap, am, Lb, Lp, Li, Wwb, Wwp, Wwi, Ri, t-L	Battes and Stoica (2005), Konecna (2012), Smith and Reichard (2013)
Gobio gobio	ah, ab, ap, am, Lb, Lp, Li, Wwb, Wwp, Wwi, Ri, t-Ww	Beers (2005)
Romanogobio albipinnatus	am, Lp, Li, Wwb, Ri, t-Ww_T, T-ab	Wanzenböck and Wanzenböck (1993)
Phoxinus phoxinus	ab_T, aj, ap, am, L0, Lb, Lp, Li, JO_W, Ww0, Wwb, Wwi, Ri, t-L, t-Ww, L-Ww, L-N, Ww-JO	Bengtsson (1974), Frost (1943), Heese (1984), Jenssen et al. (2010), Killen (2014), Kottelat and Freyhof (2007), Mills (1987), Mills and A: (1985), Museth et al. (2002), Muus and Dahlstrøm (1968), Plath et al. (2013), Schönweger et al. (2000), Stalmans and Kestemont (1991)
Chrosomus erythrogaster	am, Lp, Li, Wwb, Wwp, Wwi, t-L, L-N	Settles (1974)
Chrosomus neogaeus	ab, ap, am, Lp, Li, Wwb, Wwp, Wwi, Ri, dLp	Stasiak (1978)
Chrosomus oreas	am, Lp, Li, Wwb, Wwp, Wwi, Ri, t-L	Stasiak (1978), Thompson et al. (2017)
Chrosomus saylori	ap, am, Lp, Li, Wwb, Wwi, Ri, t-L, L-Ww	White (2012)
Chrosomus tennesseensis	am, Lp, Li, Wwb, Wwp, Wwi, t-L, L-N	Hamed et al. (2009)
Gila atraria	am, Lp, Li, Wwb, Wwp, Wwi, t-L, L-N	Neuhold (1954), Varley and Livesay (1976)
Gila coerulea	ab, am, Lp, Li, Wwb, Wwp, Wwi, t-L, L-N	Bird (1975)
Gila cypha	ah, ap, am, Lb, Li, Wwb, Wwi, R_L, t-L, L-Ww	Hamman (1982)
Gila elegans	ab, ap, am, Li, Wwb, Wwi, RL, t-L, t-Ww	
Gila intermedia	ab, ap, am, Lb, Lp, Li, Wwb, Wwi, Ri, t-L	Kucera (1978)
Gila pandora	am, Lp, Li, Wwb, Wwp, Wwi, Ri, t-L	
Gila robusta	am, Lp, Li, Wwb, Wwi, Ri, t-L, L-Ww	Vanicek and Kramer (1969)
Ptychocheilus lucius	ap, am, Li, Wwb, Wwi, t-L, L-Ww, Ww-N	Inslee (1983), Valdez et al. (2002), Vanicek and Kramer (1969)
Ptychocheilus grandis	ap, am, Li, Wwb, Wwi, Ri, t-L	Brown (1990), Inslee (1983)
Ptychocheilus oregonensis	ab, ap, am, Lp, Li, Wwb, Wwi, Ri, t-L	Beamesderfer (1992), Garcia (2014)
Siphateles bicolor	ah, ab, ap, am, Lp, Li, Wwb, Wwi, Ri, t-L, L-N, Ww-N	Archdeacon and Bonar (2009), Bird (1975), Kucera (1978)
Acrocheilus alutaceus	ab_T, ap, am, Lp, Li, Wwb, Wwp, Wwi, Ri, t-L	Moodie and Lindsey (1972)
Lavinia exilicauda	am, Lp, Li, Wwb, Wwp, Wwi, R_L, t-L	Geary and Moyle (1980)
Alburnoides bipunctatus	ab, ap, am, Li, Wwb, Wwi, Ri, t-L_f, L-Ww	Beekman and van Emmerik (2005)
Alburnoides eichwaldii Kaboudval	ah, ab, ap, am, Lb, Lp, Li, Wwb, Ri, t-L, t-Ww, L-Ww	Abbasi and Ghorbani (2010), Beekman and van Emmerik (2007)

Continuation of Table B.1		
**species**	**data**	**references**
Alburnoides eichwaldii ShirAbad	ah, ab, ap, am, Lb, Lp, Li, Wwb, Ri, t-L, t-Ww, L-Ww	Abbasi and Ghorbani (2010), Beekman and van Emmerik (2007)
Alburnoides eichwaldii Tajan	ah, ab, ap, am, Lb, Lp, Li, Wwb, Ri, t-L, t-Ww, L-Ww	Beekman and van Emmerik (2007), Keivani et al. (2016), **?**
Alburnoides eichwaldii ZarrinGol	ah, ab, ap, am, Lb, Lp, Li, Wwb, Ri, t-L, t-Ww, L-Ww	Beekman and van Emmerik (2007), Esmaeilpour Poodeh and H (2010)
Notemigonus crysoleucas	ap, am, Lp, Li, Wwb, Wwp, Wwi, t-L	Johannes et al. (1989)
Abramis brama	ab, ap, am, Lp, Li, Wwb, Wwp, Wwi, Ri, t-L	van Emmerik (2008), Zhang et al. (2016)
Mirogrex terraesanctae	am, Lp, Li, Wwb, Wwi, Ri, L-L	Ostrovsky and Walline (1999)
Vimba vimba	am, Lp, Li, Wwb, Wwi, Ri, t-L, L-Ww	Okgerman et al. (2011), Wieser et al. (1992)
Chondrostoma nasus	ap, am, Lp, Li, Wwb, Ri, t-L_f, L-Ww, T-ab	Beekman (2007)
Iberochondrostoma lemmingii	am, Lp, Li, Wwb, Wwp, Wwi, GSI, t-L	Velasco et al. (1990)
Pseudochondrostoma polylepis	am, Lp, Li, Wwb, Wwp, Wwi, Ri, t-L_f	Herrera and Fernández-Delgado (1994)
Rutilus rutilus	ab, ap, am, Lp, Li, Wwb, Wwp, Wwi, R_L, t-L_f	de Laak (2010), Raczynski et al. (2008)
Scardinius erythrophthalmus	am, Lp, Li, Wwb, Wwp, Wwi, Ri, t-L	Kennedy and Fitzmaurice (1974), Patimar et al. (2010)
Squalius cephalus	ah_T, ab_T, ap, am, Lh, Lb, Lp, Li, Wwh, Wwb, Wwi, Ri, t-L, t-Ww, L-Ww, L-N, Ww-N	Calta (2000), H. (2004), Kilic and Becer (2016), Koc et al. (2007), Kupren et al. (2011)
Squalius cii	am, Lp, Li, Wwb, GSI, t-L, L-Ww	Economou et al. (1991), Koc et al. (2007)
Squalius pyrenaicus	am, Lp, Li, Wwb, Wwp, Wwi, Ri, t-L	Fernández-Delgado and Herrera (1995)
Hemitremia flammea	ab, am, Lp, Li, Wwb, Wwp, Wwi, t-L, L-N	Muller (2012), Sossamon (1990)
Meda fulgida	am, Lp, Li, Wwb, Wwp, Wwi, Ri, t-L	Barber et al. (1970)
Lepidomeda albivallis	am, Lp, Li, Wwb, Wwp, Wwi, Ri, t-L	Billman et al. (2011), Scoppettone et al. (2004)
Lepidomeda aliciae	am, Lp, Li, Wwb, Wwp, Wwi, Ri, t-L	Billman et al. (2011)
Lepidomeda copei	am, Lp, Li, Wwb, Wwp, Wwi, Ri, t-L	Johnson et al. (1995)
Semotilus atromaculatus	am, Lp, Li, Wwb, Ri, t-L, L-Ww	Henshell et al. (2006)
Semotilus corporalis	am, Lp, Li, Wwb, Wwi, Ri, t-L	Reed (1971)
Margariscus margarita	am, Lp, Li, Wwb, Wwi, Ri, t-L	Fava and fa Tsai (1974)
Rhynchocypris oxycephalus	am, Lp, Li, Wwb, Wwi, R_L, t-L	Ling et al. (2015)
Iotichthys phlegethontis	am, Lp, Li, Wwb, Wwp, Wwi, Ri, t-L	Hanks and Belk (2004), Mills et al. (2004)
Mylocheilus caurinus	am, Lp, Li, Wwb, Wwp, Wwi, t-L, L-N	Cartwright (1959), Moyle et al. (2004), Scott (1952)
Pogonichthys macrolepidotus	ap, am, Lp, Li, Wwb, Wwp, Wwi, Ri, t-L	Moyle et al. (2004)

Continuation of Table B.1		
**species**	**data**	**references**
Richardsonius egregius	am, Lp, Li, Wwb, Wwp, Wwi, t-L, L-N, L-Ww	Bird (1975), Evens (1969)
Exoglossum maxillingua	am, Lp, Li, Wwb, Wwp, Wwi, Ri, t-L	Cowley et al. (2013), Maurakis and Green (2001), Pappantoniou et al. (1964)
Oregonichthys crameri	ab, am, Lp, Li, Wwb, Wwi, Ri, t-L	Anonymous (1998), Scheerer and McDonald (2003)
Rhinichthys atratulus	am, Lp, Li, Wwb, Wwp, Wwi, Ri, t-L_f	Fraker et al. (2002), Reed and Moulton (1973)
Rhinichthys cataractae	am, Lp, Li, Wwb, Wwp, Wwi, Ri, t-L_f	Fraker et al. (2002), Reed and Moulton (1973)
Campostoma anomalum	am, Lp, Li, Wwb, Wwp, Wwi, Ri, t-L	
Agosia chrysogaster	am, Lp, Li, Wwb, Wwp, Wwi, t-L, L-N	Kepner (1982), Winther (1962)
Blicca bjoerkna	ah, ab, ap, am, Lb, Lp, Li, Wwb, Ri, t-L, t-Ww, L-Ww	Schoone and van Breugel (2006), Yilmaz et al. (2015)
Cyprinella analostana	am, Lp, Li, Wwb, Wwp, Wwi, Ri, t-L, L-Ww	Denoncourt and Messersmith (1982)
Cyprinella lutrensis	am, Lp, Li, Wwb, Wwp, Wwi, Ri, t-L	Coburn (1986)
Cyprinella trichroistia	am, Lp, Li, Wwb, Wwp, Wwi, Ri, t-L	Scott and Mayden (2008)
Cyprinella venusta	am, Lp, Li, Wwb, Wwp, Wwi, Ri, t-L	
Dionda argentosa	am, Lp, Li, Wwb, Wwi, Ri, t-L	Gibson and Fries (2005), Robertson et al. (2016)
Dionda diaboli	ah, ab, am, Lb, Lp, Li, Wwb, Wwi, Ri, t-L	Gibson and Fries (2005), Hulbert et al. (2007)
Hybognathus amarus	am, Lp, Li, Wwb, Wwp, Wwi, t-L_f, L-N	Caldwell et al. (2019), Cowley et al. (2006)
Hybognathus nuchalis	am, Lp, Li, Wwb, Wwp, Wwi, t-L_f, L-N	Ramírez-García and Piller (2018)
Hybognathus placitus	am, Lp, Li, Wwb, Wwp, Wwi, t-L_f, L-N	Taylor and Miller (1990)
Hybopsis amnis	am, Lp, Li, Wwb, Wwp, Wwi, Ri	Coburn (1986)
Luxilus chrysocephalus	ap, am, Lp, Li, Wwb, GSI, t-L, t_Ww	Coburn (1986), Simmons and Beckman (2012)
Luxilus cornutus	ap, am, Lp, Li, Wwb, Wwp, Wwi, t-L_f, Ww-N	Coburn (1986), Fee (1965)
Luxilus pilsbryi	am, Lp, Li, Wwb, GSI, t-L, t_Ww	Coburn (1986), Simmons and Beckman (2012)
Lythrurus roseipinnis	am, Lp, Li, Wwb, Wwi, t-L, L-N	Heins and Bresnick (1975)
Lythrurus umbratilis	am, Lp, Li, Wwb, Wwp, Wwi, Ri	Coburn (1986)
Macrhybopsis gelida	ap, am, Lp, Li, Wwb, Wwp, Wwi, Ri, t-L	Dieterman et al. (2006), Starks et al. (2016)
Macrhybopsis hyostoma	ap, am, Lp, Li, Wwb, Wwp, Wwi, Ri, t-L	Dieterman et al. (2006), Scoppettone et al. (1992), Starks et al. (2016)
Macrhybopsis meeki	ap, am, Lp, Li, Wwb, Wwp, Wwi, t-L, L-N	Dieterman et al. (2006), Starks et al. (2016)
Macrhybopsis storeriana	tp, am, Lp, Li, Wwb, Wwp, Wwi, Ri, tL	Dieterman et al. (2006)

Continuation of Table B.1		
**species**	**data**	**references**
Moapa coriacea	ap, am, Lp, Li, Wwb, Wwp, Wwi, L-dL, L-N	Hereford (2014), Scoppettone et al. (1992)
Notropis amabilis	am, Lp, Li, Wwb, Wwp, Wwi, Ri, t-L	Coburn (1986)
Notropis atherinoides	am, Lp, Li, Wwb, Wwp, Wwi, Ri, t-L	Coburn (1986), Fuchs (1967)
Notropis atrocaudalis	am, Lp, Li, Wwb, Wwp, Wwi, Ri, t-L	Coburn (1986)
Notropis bifrenatus	am, Lp, Li, Wwb, Wwi, t-L, L-N	Harrington (1948)
Notropis blennius	am, Lp, Li, Wwb, Wwp, Wwi, Ri, t-L	Coburn (1986)
Notropis boops	am, Lp, Li, Wwb, Wwi, GSI, t-L	Lehtinen and Echelle (1979)
Notropis buccula	am, Lp, Li, Wwb, Wwp, Wwi, Ri, t-L	Marks (1999)
Notropis buccatus	am, Lp, Li, Wwb, Ri, t-L, t-Ww, L-Ww	Hoyt (1971)
Notropis buchanani	am, Lp, Li, Wwb, Wwp, Wwi, Ri, t-L	Coburn (1986)
Notropis chalybaeus	am, Lp, Li, Wwb, Wwp, Wwi, t-L, L-N	Perkin et al. (2012)
Notropis chrosomus	am, Lp, Li, Wwb, Wwp, Wwi, Ri, t-L_f, t-Ww	Holder and Powers (2010)
Notropis girardi	am, Lp, Li, Wwb, Wwp, Wwi, Ri, t-L	Coburn (1986)
Notropis heterolepis	am, Lp, Li, Wwb, Wwi, t-L, L-N	Emery and Wallace (1974), Roberts et al. (2006)
Notropis hudsonius	am, Lp, Li, Wwb, t-L, L-Ww, L-N	Coburn (1986), Wells and House (1974)
Notropis jemezanus	am, Lp, Li, Wwb, Wwp, Wwi, Ri, t-L	
Notropis longirostris	am, Lp, Li, Wwb, Wwi, t-L, L-N	Heins and Clemmer (1976)
Notropis maculatus	ab, am, Lp, Li, Wwb, Wwp, Wwi, Ri, t-L	
Notropis oxyrhynchus	am, Lp, Li, Wwb, Wwp, Wwi, Ri, t-L	Coburn (1986), Marks (1999)
Notropis petersoni	am, Lp, Li, Wwb, Wwi, t-L, L-N	Davis and Louder (1971)
Notropis potteri	am, Lp, Li, Wwb, Wwp, Wwi, Ri, t-L	Coburn (1986)
Notropis rafinesquei	am, Lp, Li, Wwb, Ri, t-L, L-Ww	Haag et al. (2007)
Notropis rubellus	am, Lp, Li, Wwb, Wwi, Ri, t-L	Reed (1957)
Notropis sabinae	am, Lp, Li, Wwb, Wwp, Wwi, t-L_f, L-N	Heins (1981)
Notropis spectrunculus	am, Lp, Li, Wwb, Wwp, Wwi, Ri, t-L	Olson and Martin (2016)
Notropis stramineus	ap, am, Lp, Li, Wwb, Wwp, Wwi, Ri, t-L	Summerfelt and Minckley (1969)
Notropis texanus	am, Lp, Li, Wwb, Wwp, Wwi, Ri, t-L	
Notropis topeka	ab, am, Lp, Li, Wwb, Wwp, Wwi, t-L, L-N	Dahle (2001)
Notropis volucellus	am, Lp, Li, Wwb, Wwp, Wwi, Ri, t-L	
Notropis xaenocephalus	am, Lp, Li, Wwb, Wwi, t-L, L-N	Jolly and Powers (2008)
Opsopoeodus emiliae	am, Lp, Li, Wwb, Wwp, Wwi, Ri, t-L	
Phenacobius mirabilis	am, Lp, Li, Wwb, Wwp, Wwi, t-L_T	Bestgen and Compton (2007)
Pimephales notatus	am, Lp, Li, Wwb, Ri, t-L, t-Ww, L-Ww	Coburn (1986), Gale (1983), Gill and Weatherley (1984)
Pimephales promelas	ab, ap, am, Lb, Lp, Li, Wd0, Ww0, Wwb, Wwp, Wwi, Ri, t-L, t-Ww, L-Ww, t-N, t-dC, t-WwR	Boehler (2012), Boros et al. (2015), Braunbeck et al. (1998), Grant and Tonn (2002), Panter et al. (2012), Parrott and Balakrishnan (2017), Sohoni et al. (2001), Van Aerle et al. (2004), Wang (1986), Watanabe et al. (2007), Weber et al. (2003)

Continuation of Table B.1		
**species**	**data**	**references**
Pimephales vigilax	ab, am, Lp, Li, Wwb, Wwp, Wwi, Ri, t-L	Parker (1964)
Platygobio gracilis	am, Lp, Li, Wwb, Wwp, Wwi, t-L, L-N_f	Gould (1985), Martyn and Schmulbach (1978)
Pteronotropis welaka	am, Lp, Li, Wwb, Wwp, Wwi, Ri, t-L	Johnston and Knight (1999)
Catostomus catostomus	am, Lp, Li, Wwb, Wwi, Ri, t-L	Bailey (1969)
Catostomus columbianus	am, Lp, Li, Wwb, Wwp, Wwi, Ri, t-L	Dauble (1980)
Catostomus commersonii	am, Lp, Li, Wwb, Wwp, Wwi, Ri, t-L	Beamish (1973), Beckman and Hutson (2012)
Catostomus latipinnis	am, Lp, Li, Wwb, Wwi, Ri, t-L	McAda and Wydoski (1985)
Catostomus macrocheilus	ap, am, Lp, Li, Wwb, Wwp, Wwi, Ri, dLj	Dauble (1980, 1986)
Catostomus microps	am, Lp, Li, Wwb, Wwp, Wwi, Ri, t-L	Moyle and Marciochi (1975)
Catostomus platyrhynchus	am, Lp, Li, Wwb, Wwp, Wwi, R_L, t-L	Hauser (1968)
Catostomus rimiculus	am, Lp, Li, Wwb, Wwp, Wwi, Ri, t-L	Hauser (1968), Hohler (1981)
Catostomus santaanae	ap, am, Lh, Lb, Lp, Li, Wwb, Wwp, Wwi, Ri	Bailey (1969), Greenfield et al. (1970), Warren and Brooks (2014)
Catostomus tahoensis	am, Lp, Li, Wwb, Wwp, Wwi, t-L, t-Ww, L-N	Kennedy et al. (1978)
Catostomus warnerensis	am, Lp, Li, Wwb, Wwp, Wwi, L-dL, L-Ww	Dauble (1980), Scheerer et al. (2011)
Chasmistes brevirostris	ab, ap, am, Lp, Li, Wwb, Wwi, Ri,	Warren and Burr (2014)
Chasmistes cujus	ab, ap, am, Lp, Li, Wwb, Wwi, Ri, t-L	Scoppettone (1988), Warren and Burr (2014)
Chasmistes liorus	ab, ap, am, Lp, Li, Wwb, Wwi, Ri, t-L	Belk (1998), Warren and Burr (2014)
Deltistes luxatus	am, Lp, Li, Wwb, Wwp, Wwi, Ri, t-L	Terwilliger et al. (2010)
Xyrauchen texanus	ap, am, Lh, Lp, Li, Wwb, Wwi, Ri	Bailey (1969)
Erimyzon oblongus	am, Lp, Li, Wwb, Wwp, Wwi, Ri, t-L	
Erimyzon sucetta	am, Lp, Li, Wwb, Wwp, Wwi, Ri, t-L	
Minytrema melanops	am, Lp, Li, Wwb, Wwi, Ri, t-L	Grabowski et al. (2012)
Hypentelium etowanum	am, Lp, Li, Wwb, Ri, t-L, t-tWw	O'Kelley and Powers (2007)
Hypentelium nigricans	am, Lp, Li, Wwb, Wwp, Wwi, Ri, t-L	Raney and Lachner (1946)
Thoburnia atripinnis	am, Lp, Li, Wwb, Wwp, Wwi, Ri, t-L	Timmons et al. (1983)
Thoburnia rhothoeca	am, Lp, Li, Wwb, Wwp, Wwi, t-L, L-N	Raney and Lachner (1946)
Moxostoma anisurum	am, Lp, Li, Wwb, Wwp, Wwi, Wwi, Ri, t-L	Mongeau et al. (1992), Thompson et al. (2015)
Moxostoma carinatum	am, Lp, Li, Wwb, Wwp, Wwi, Ri, t-L	Beckman and Hutson (2012)
Moxostoma cervinum	am, Lp, Li, Wwb, Wwp, Wwi, Wwi, Ri, t-L	Thompson et al. (2015)
Moxostoma congestum	am, Lp, Li, Wwb, Wwp, Wwi, Ri, t-L_f	Thompson et al. (2015), Zymonas and Propst (2007)
Moxostoma duquesnii	am, Lp, Li, Wwb, Wwp, Wwi, Ri, t-L	Reid (2009)
Moxostoma erythrurum	am, Lp, Li, Wwb, Wwp, Wwi, Ri, t-L	Keeton (1963), Mitton and Lewis (1989)
Moxostoma hubbsi	am, Lp, Li, Wwb, Wwp, Wwi, Ri, t-L	Mongeau et al. (1992), Thompson et al. (2015)

Continuation of Table B.1		
**species**	**data**	**references**
Moxostoma macrolepidotum	am, Lp, Li, Wwb, Wwi, Wwi, Ri, t-L, L-Ww	Sule and Skelly (1985)
Moxostoma poecilurum	am, Lp, Li, Wwb, Wwi, Ri, t-L	Grabowski et al. (2012)
Moxostoma valenciennesi	am, Lp, Li, Wwb, Wwi, Wwi, Ri, t-L	Mongeau et al. (1992), Thompson et al. (2015)
Cycleptus elongatus	am, Lp, Li, Wwb, Wwp, Wwi, GSI, t-L	
Cycleptus meridionalis	am, Lp, Li, Wwb, Wwi, GSI, t-L, t-Ww	Peterson et al. (1999)
Carpiodes carpio	am, Lp, Li, Wwb, Wwp, Wwi, Ri, t-L, L-Ww	Morris (1965)
Carpiodes cyprinus	am, Lp, Li, Wwb, Wwi, Ri, t-L	Grabowski et al. (2012)
Carpiodes velifer	am, Lp, Li, Wwb, t-L, t-Ww, L-N	Woodward and Wissing (1976)
Ictiobus cyprinellus	am, Lp, Li, Wwb, Wwi, Ri, t-L, L-Ww	Johnson (1959)
Ictiobus bubalus	am, Lp, Li, Wwb, Wwi, t-L, L-Ww, L-N	Jester (1973)
Ictiobus niger	am, Lp, Li, Wwb, Wwp, Wwi, Ri, t-L	
Cobitis taenia	ap, am, Lb, Lp, Li, Wwb, Wwp, Wwi, Ri, t-L, t-Ww	Robotham (1981)
Cobitis paludica	am, Lp, Li, Wwb, Wwp, Wwi, Ri, t-L	Oliva-Paterna et al. (2002)
Hydrocynus vittatus	ah, ab, aj, ap, am, Lh, Lb, L_t, Lp, Li, Wwi, Ri	Steyn et al. (1996)
Hoplias malabaricus	ab, am, Lp, Li, Wwb, Wwi, Ri, t-W	Daniel et al. (2019)
Colossoma macropomum	am, Lp, Li, Wwb, Wwi, Ri, t-L	Costa et al. (2013)
Piaractus brachypomus	ab, am, Lp, Li, Wwb, Wwi, Ri, t-L	Guerreiro et al. (2018)
Prochilodus nigricans	ab, am, Lp, Li, Wwb, Wwi, Ri, t-L	Guerreiro et al. (2018)
Semaprochilodus taeniurus	ab, am, Lp, Li, Wwb, Wwi, Ri, t-L	Guerreiro et al. (2018)
Semaprochilodus insignis	ab, am, Lp, Li, Wwb, Wwi, Ri, t-L	Guerreiro et al. (2018)
Leporinus friderici	am, Lp, Li, Wwb, Wwp, Wwi, Ri, t-L	Boujard et al. (1991), Gosmann and de Oliveira Nuner (2015)
Astyanax mexicanus	ab, am, Lp, Li, Wwb, Wwi, Ri, t-L	Simon et al. (2017)
Hyphessobrycon pulchripinnis	ab, ap, am, Li, Wwb, Wwi, Ww_L, Ri, t-L	Cole et al. (1999)
Moenkhausia dichroura	ab, am, Lp, Li, Wwb, Wwi, Ri, t-L	Cunha et al. (2007)
Brycon moorei	ab, am, Lp, Li, Wwb, Wwi, Ri, t-L	Baras and Lucas (2005, 2010), Mancera-Rodriguez (2017)
Brycon opalinus	ab, am, Lp, Li, Wwb, Wwi, Ri, t-L	Gomiero et al. (2007)
Brycon amazonicus	ab, am, Lp, Li, Wwb, Wwi, Ri, t-L	Guerreiro et al. (2018)
Salminus brasiliensis	am, Lp, Li, Wwb, Wwi, Ri, t-L	Zuliani et al. (2016)
Electrophorus electricus	ah, ap, am, Lh, Lb, Lp, L_t, Li, Wwp, Wwi, Ri, t-L	Assuncao and Schwassmann (1995), de Santana et al. (2013)
Apteronotus leptorhynchus	ab, am, Lp, Li, Wwb, GSI, t-L, L-Ww	Ilieş et al. (2014), Kirschbaum and Schugardt (2002)
Corydoras aeneus	ab, ap, am, Lb, Lp, Li, Wwb, Wwp, Wwi, Ri, t-L	Huysentruyt et al. (2009), **?**

Continuation of Table B.1		
**species**	**data**	**references**
Silurus glanis	ab, ap, am, Lb, Lp, Li, Wwb, Wwp, Wwi, Ri, t-L, L-Ww	Alp et al. (2011)
Clarias gariepinus	ab_T, am, Li, Wwb, Wwp, Wwi, GSI, t-Ww	Legendre et al. (1992)
Clarias gariepinus x Heterobranchus longifilis	ab_T, am, Li, Wwb, Wwp, Wwi, GSI, t-Ww	Legendre et al. (1992)
Heterobranchus longifilis	ab_T, am, Li, Wwb, Wwp, Wwi, GSI, t-Ww	Legendre et al. (1992)
Heterobranchus longifilis x Clarius gariepinus	ab_T, am, Li, Wwb, Wwp, Wwi, GSI, t-Ww	Legendre et al. (1992)
Ariopsis felis	am, Lp, Li, Wwb, Wwp, Wwi, Ri, t-L, L-Ww	Flinn et al. (2019)
Bagre marinus	am, Lp, Li, Wwb, Wwp, Wwi, Ri, t-L, L-Ww	Flinn et al. (2019)
Pangasianodon hypophthalmus	ab, ap, am, Lb, Li, Wwb, Wwp, Wwi, Ri, t-Ww	Chowdhury et al. (2020), Legendre et al. (1998), Mukai et al. (2010)
Ictalurus furcatus	am, Lp, Li, Wwb, Wwp, Wwi, Ri, t-L	
Ictalurus punctatus	ab, ap, am, Lb, Lp, Li, Wwb, Wwp, Wwi, Ri, t-L, T-JO, T-dWw_f, Ww-JO, Ww-JO, t-N, L-Ww	Andrews and Matsuda (1975), Andrews and Stickney (1972), Graham et al. (1999), Jearald and B.E. (1971), Moody-Carpentera et al. (2015), Pawiroredjo (2004), Raibley and Jahn (1991), Sakaris and Irwin (2008), Tyus and Nikirk (1990)
Pylodictis olivaris	ab, am, Lp, Li, Wwb, Wwi, Ri, t-L, L-Ww	Grabowski et al. (2004)
Ameiurus melas	ab, am, Lp, Li, Wwb, Wwi, Ri, t-L	Copp et al. (2016)
Ameiurus natalis	am, Lp, Li, Wwb, Wwp, Wwi, Ri, t-L	
Ameiurus nebulosus	ab, am, Lp, Li, Wwb, Wwi, Ri, t-L	Gash and Bass (1973)
Noturus albater	am, Lp, Li, Wwb, Wwi, Ri, t-L, L-N	Mayden et al. (1980)
Noturus baileyi	ab, am, Lp, Li, Wwb, Wwi, Ri, t-L	Dinkins and Shute (1996)
Noturus eleutherus	am, Lp, Li, Wwb, Wwi, R_L, t-L	Starnes and Starnes (1985)
Noturus exilis	ab, am, Lp, Li, Wwb, Wwp, Wwi, t-L, Ww-N	Vives (1987)
Noturus flavipinnis	am, Lp, Li, Wwb, Wwi, Ri, t-L	Dinkins and Shute (1996)
Noturus flavus	am, Lp, Li, Wwb, Wwi, Ri, t-L	Puchala et al. (2018)
Noturus funebris	am, Lp, Li, Wwb, Wwi, Ri, t-L	Bennett and Kuhajda (2008)
Noturus gyrinus	am, Lp, Li, Wwb, Wwp, Wwi, Ri, t-L	
Noturus hildebrandi	am, Lp, Li, Wwb, t-L, t-Ww, L-N	Baker and Heins (1994), Mayden and Walsh (1984)
Noturus insignis	ab, am, Lp, Li, Wwb, Ri, t-L, L-Ww	Clugston and Cooper (1982)
Noturus lachneri	am, Lp, Li, Wwb, Wwp, Wwi, Ri, t-L	Tumilson and Hardage (2014)
Noturus miurus	am, Lp, Li, Wwb, t-L, L-Ww, L-N	Burr and Mayden (1982)
Noturus munitus	am, Lp, Li, Wwb, Wwi, Ri, t-L	Bennett et al. (2010), Starnes and Starnes (1985)

Continuation of Table B.1		
**species**	**data**	**references**
Noturus nocturnus	am, Lp, Li, Wwb, Wwp, Wwi, Ri, t-L	
Noturus phaeus	ab, am, Lp, Li, Wwb, Wwp, Wwi, Ri, t-L	Chan and Parsons (2000), Simon and Wallus (2003)
Noturus stigmosus	am, Lp, Li, Wwb, t-L, L-Ww, L-N	Scheibly (2003)
Lepidogalaxias salamandroides	am, Lp, Li, Wwb, t-L, L-Ww, L-N	Morgan et al. (2000)
Argentina silus	am, Lb, Lp, Li, Wwb, Wwp, Wwi, Ri, t-L, L-Ww	Magnussen (2007), Munk and Nielsen (2005)
Pseudobathylagus milleri	am, Lp, Li, Wwb, Wwp, Wwi, Ri, t-L	Childress et al. (1980)
Leuroglossus stilbius	am, Lp, Li, Wwb, Wwp, Wwi, Ri, t-L	Childress et al. (1980)
Galaxias paucispondylus	am, Lp, Li, Wwb, Ri, t-L, L-Ww, L-N	Bonnett (1990, 1992), Boy et al. (2007)
Galaxias prognathus	am, Lp, Li, Wwb, Ri, t-L, L-Ww	Bonnett (1990, 1992), Boy et al. (2007)
Galaxias maculatus	am, Lb, Lp, Li, Wwb, Ri, t-L_f, L-Ww	Barriga et al. (2012), Bonnett (1990), Boy et al. (2007)
Coregonus albula	ap, am, Lp, Li, Wwb, Wwi, Ri, t-L, L-Ww	Kozowski et al. (2010)
Coregonus artedi	am, Lp, Li, Wwb, Wwp, Wwi, Ri, t-L	Stockwell et al. (2009)
Coregonus autumnalis	am, Lp, Li, Wwb, Wwp, Wwi, Ri, t-L_T, t-Ww_T	Fechhelm et al. (1993)
Coregonus clupeaformis	am, Lp, Li, Wwb, Wwp, Wwi, Ri, t-L	Oosten and Hile (1949)
Coregonus hoyi	am, Lp, Li, Wwb, Wwp, Wwi, Ri, t-L	Schaeffer (2004)
Coregonus kiyi	ab, am, Lp, Li, Wwb, Wwp, Wwi, Ri, t-L, t-Ww	Deason and Hile (1947)
Coregonus laurettae	am, Lp, Li, Wwb, Wwp, Wwi, Ri, t-L	Alt (1973)
Coregonus lavaretus	am, Lp, Li, Wwb, Wwi, GSI, t-L, L-Ww	Szczepkowski et al. (2010), Tolonen (1997)
Coregonus nasus	am, Lp, Li, Wwb, Wwp, Wwi, Ri, t-L	Griffiths et al. (1992)
Coregonus pidschian	am, Lp, Li, Wwb, Wwp, Wwi, Ri, t-L	Bochkarev et al. (2018)
Coregonus reighardi	am, Lp, Li, Wwb, Wwp, Wwi, Ri, t-L	Jobes (1943)
Coregonus sardinella	am, Lp, Li, Wwb, Wwp, Wwi, Ri, t-L	Muir et al. (2014)
Coregonus zenithicus	am, Lp, Li, Wwb, Wwp, Wwi, Ri, t-L	Muir et al. (2014)
Prosopium abyssicola	am, Lp, Li, Wwb, Wwp, Wwi, t-L, L-N, L-Ww	Shestakov (2017), Thompson (2003)
Prosopium coulterii	ap, am, Li, Wwb, Wwi, Ri, t-L	Stewart et al. (2016)
Prosopium cylindraceum	am, Lp, Li, Wwb, t-L, t-Ww, L-N	Shestakov (2017)
Prosopium gemmifer	am, Lp, Li, Wwb, Wwp, Wwi, Ri, t-L_f	Frantz and Cordone (1965), Shestakov (2017)
Prosopium spilonotus	am, Lp, Li, Wwb, Wwp, Wwi, t-L, L-N, L-Ww	Shestakov (2017), Thompson (2003)
Prosopium williamsoni	am, Lp, Li, Wwb, Wwp, Wwi, Ri, t-L	Benjamin et al. (2014), Shestakov (2017)
Stenodus leucichthys	am, Lp, Li, Wwb, Wwp, Wwi, Ri, t-L	Alt (1973)
Thymallus arcticus	am, Lp, Li, Wwb, Wwp, Wwi, R_i, t-L	Craig and Poulin (1975)
Thymallus thymallus	ah_T, ap, am, Lh, Lp, Li, R_L, GSI, t-L_f, t-Ww, L-Ww, T-ah	Hellawell (1969), Ingram et al. (20aa), Jungwirth and Winkler (1984), Laak (2008)

Continuation of Table B.1		
**species**	**data**	**references**
Thymallus tugarinae	am, Lp, Li, Wwb, t-L_f, t-Ww_f, L-N	Mikheeva et al. (2012, 2013)
Oncorhynchus clarkii stomias	ah, ab, ap, am, Lb, Lp, Li, Wwb, Wwp, Wwi, Ri, t-L_T, t-Ww_T	Cartwright et al. (1998), Coleman and Fausch (2007), Scarnecchia and Bergersen (1986), Young (2009)
Oncorhynchus gilae	ab, am, Lp, Li, Wwb, Wwp, Wwi, L-dL	Rinne (1982)
Oncorhynchus gorbuscha	ab, am, Lp, Li, Wwb, Wwp, Wwi, t-L	LeBrasseur and Parker (1964)
Oncorhynchus keta	ab, am, Lb, Lp, Li, Wwb, Wwi, t-dL, L-N	Beacham (1982), Urbach et al. (2012)
Oncorhynchus kisutch	ab, ap, am, Lp, Li, Wwb, Wwp, Wwi, Ni, t-L	Beacham et al. (1985), Raymond (1986)
Oncorhynchus mykiss	ah_T, ab_T, ap, am, Lp, Li, Wd0, Wdh, Wdb, Wwp, Wwi, Ri, t-Ww_f, t-L_f, t-Wde, t-WdYe, T-ah, Ww-JO	Davidson et al. (2014), From and Rasmussen (1991), Kieffer et al. (1998), Lauff and Wood (1996), Ninness et al. (2006), Sumpter et al. (1991), Tyler et al. (1996), Velsen (1987), Weatherley and Gill (1981), Wieser (1985), Yanik et al. (2002)
Oncorhynchus nerka	ab, ap, am, Lp, Li, Wwb, Wwp, Wwi, Ni, t-Ww	Beacham et al. (1985)
Oncorhynchus tshawytscha	ab, ap, am, Lb, Lp, Li, Wd0, Wwb, Wwp, Wwi, Ni, E0, t-Le_T, L-Wde_T, T-ab, t-L_fT, t-Ww_fT, L-Ww, L-N	Allen and Hassler. (1985), Beacham and Murray (1990), Beacham and Murray. (1993), Berg et al. (2001a), Einum et al. (2003), Heming (1982), Jasper and Evenson (2006), Orsi and Jaenicke (1996), Shelbourn et al. (1995), Zabel and Achord (2004)
Salmo trutta	ab_T, ap, am, Lh, Lb, Lp, Li, Ri, L-Ww, T-ah	Elliot (1975, 1984, 1994), Klemetrsen et al. (2003), Ojanguren and Brana (2003)
Salmo salar	ab, aj, ap, am, Lb, Lj, Li, Wwb, Wwj, Ri, L-Ww, t-L	Berg et al. (2001b), Einum and Fleming (2000), Fleming (1996), Flower (1935), Hutchings and Jones (1998), Jutila et al. (2006), Thorpe et al. (1984a,b), Van Leeuwen et al. (2016)
Salvelinus alpinus	ah, ab, ap, am, Lb, Lp, Li, Wwh, Wwi, Ri, t-Ww	Gruber and Wieser (1983), Yanik et al. (2002)
Salvelinus curilus	am, Lp, Li, Wwb, Ri, t-L_f, L-Ww	Kolpakov et al. (2014)
Salvelinus fontinalis	am, Lp, Li, Wwb, Wwp, Wwi, Ri, t-L	Öhlund et al. (2008)
Salvelinus leucomaenis	am, Lp, Li, Wwb, Wwi, Ri, t-L	Morita (2001)
Salvelinus malma	am, Lp, Li, Wwb, Wwi, Ri, t-L, t-Ww	Esin (2015)
Salvelinus namaycush	am, Lp, Li, Wwb, Wwi, Ri, t-L_f	Morbey et al. (2010)
Salvelinus willoughbii	ab, am, Lp, Li, Wwb, Wwi, Ri, t-L, L-Ww	Frost and Kipling (1980)
Esox americanus	am, Lp, Li, Wwb, Wwp, Wwi, Ri, t-L	
Esox lucius	ab, ap, am, Lb, Lp, Li, Wwb, Wwp, Wwi, Ri, t-L, t-Ww	Anderson and Neumann (1996), Bregazzi and Kennedy (1980), Žiliukien and Žiliukas (2010)

Continuation of Table B.1		
**species**	**data**	**references**
Esox masquinongy	ab, ap, am, Lp, Li, Wwb, Wwp, Wwi, Ri, t-L	Crane et al. (2020), Wallus et al. (1990)
Esox niger	ab, ap, am, Lp, Li, Wwb, Wwp, Wwi, Ri, t-L	Crane et al. (2020), Wallus et al. (1990)
Dallia pectoralis	ap, am, Lp, Li, Wwb, Wwp, Wwi, Ri, t-L, L-Ww, Ww-Ww_R	Blackett (1962)
Umbra limi	ab, ap, am, Lp, Li, Wwb, Wwi, Ri, t-L_f, t-Ww_f, L-Ww	Robinson et al. (2010), Wallus et al. (1990)
Umbra pygmaea	ap, am, Lp, Li, Wwb, Wwi, t-L, L-N	Panek and Weis (2012)
Umbra krameri	ab, ap, am, Lp, Li, Wwb, Wwi, Ri, t-L	Kováč (1995)
Osmerus mordax	ab, ap, am, Lb, Lp, Li, Wwb, Ri, t-L, L-Ww, T-ab	Jones and McCarthy (2013), Maitland and Lyle (2010), Quigley et al. (2004)
Osmerus eperlanus	ab, am, Lp, Li, Wwb, Ri, t-L, t-Ww	Korlyakov and Mukhachev (2009), Quigley et al. (2004)
Mallotus villosus	ab, ap, am, Lb, Lp, Li, Ri, L-Ww, L-N, Ww-N	Jóhannsdóttir and Vilhjálmsson (1999)
Hypomesus nipponensis	am, Lb, Lp, Li, Wwb, Ri, t-L_Tf	Kudo and Mizuguchi (2000)
Spirinchus thaleichthys	am, Lb, Lp, Li, Wwb, Wwp, Wwi, t-L_f, t-Ww_f, L-N	Chigbu and Sibly (1994)
Thaleichthys pacificus	am, Lb, Lp, Li, Wwb, Wwp, Wwi, Ri, t-L	Chigbu and Sibly (1994), Clarke et al. (2007)
Retropinna semoni	am, Lp, Li, Wwb, Wwp, Wwi, Ri, t-L	Milton and Arthington (1985)
Maurolicus muelleri	am, Lp, Li, Wwb, Wwp, Wwi, t-L, t-Ww, Ww-N	Goodson et al. (1995), Munk and Nielsen (2005)
Maurolicus imperatorius	am, Lp, Li, Wwb, Wwp, Wwi, t-L	Savinykh and Baytalyuk (2010)
Vinciguerria nimbaria	ap, am, Lp, Li, Wwb, Wwi, t-L, L-N	Javier Tomás (2000), Stequert et al. (2003)
Borostomias panamensis	am, Lp, Li, Wwb, Wwp, Wwi, Ri, t-L	Childress et al. (1980)
Harpadon nehereus	am, Lp, Li, Wwb, Wwi, Ri, L-dL	Firdaus et al. (2017), Ghosh (2014)
Saurida undosquamis	am, Lp, Li, Wwb, Wwi, R_W, t-L	El-Halfawy et al. (2007), Kadharsha et al. (2013), Yoneda et al. (2002)
Bathypterois dubius	am, Lp, Li, Wwb, Wwi, GSI, t-L	Morales-Nine et al. (1996), Porcu et al. (2010)
Chlorophthalmus agassizi	am, Lp, Li, Wwb, Wwi, GSI, t-L	Anastasopoulou and Papaconstantinou (2006), Cabiddu et al. (2010)
Benthosema suborbitale	aj, am, Lp, Li, Wwb, Wwp, Wwi, t-L, L-N	Gartner (1991, 1993)
Benthosema pterotum	ab, am, Lp, Li, Wwb, Ri, t-L, L-Ww	Gjøsæter and Tilseth (1988), Hosseini-Shekarabi et al. (2015)
Benthosema glaciale	am, Lp, Li, Wwb, Wwp, Wwi, Ri, t-L	Gjösæter (1973)
Electrona antarctica	aj, am, Lp, Li, Wwb, Wwi, Ri, t-L	Greely et al. (1999)
Myctophum asperum	am, Lp, Li, Wwb, Wwp, Wwi, Ri, t-L	Hayashi et al. (2001)
Symbolophorus californiensis	am, Lp, Li, Wwb, Wwp, Wwi, Ri, t-L	Takagi et al. (2006)
Tarletonbeania crenularis	am, Lj, Lp, Li, Wwb, Wwp, Wwi, Ri, t-L	Bystydzieńska et al. (2010)

Continuation of Table B.1		
**species**	**data**	**references**
Ceratoscopelus warmingii	am, Lp, Li, Wwb, Wwp, Wwi, Ri, t-L	Takagi et al. (2006)
Diaphus dumerilii	aj, am, Lp, Li, Wwb, Wwp, Wwi, Ri, t-L	Gartner (1991, 1993)
Diaphus theta	aj, am, Lp, Li, Wwb, Wwp, Wwi, Ri, t-L	Moku et al. (2001)
Nannobrachium ritteri	am, Lp, Li, Wwb, Wwp, Wwi, Ri, t-L	Childress et al. (1980)
Nannobrachium regale	am, Lp, Li, Wwb, Wwp, Wwi, Ri, t-L	Childress et al. (1980)
Stenobrachius leucopsarus	am, Lp, Li, Wwb, Wwp, Wwi, Ri, t-L	Childress et al. (1980)
Triphoturus mexicanus	am, Lp, Li, Wwb, Wwp, Wwi, Ri, t-L	Childress et al. (1980)
Lampanyctodes hectoris	am, Lp, Li, Wwb, Wwp, Wwi, Ri, t-L	Prosch (1991), Robertson (1977), Young et al. (1988)
Notoscopelus resplendens	ap, am, Li, Wwb, Ri, t-Ww, L-Ww	Sarmiento-Lezcano et al. (2018)
Notoscopelus elongatus	am, Lp, Li, Wwb, Wwi, Ri, t-L	Gjøsæter (1981)
Lepidophanes guentheri	am, Lp, Li, Wwb, Wwp, Wwi, t-L, L-N	Gartner (1991, 1993)
Lampris guttatus	ap, am, Lp, Li, Wwb, Wwi, Ri, t-L	Cole (2010), Francis et al. (2004)
Percopsis omiscomaycus	ah, ab, ap, am, Lh, Lp, Li, L_W, Wwb, Wwp, Wwi, R_L, t-L_f	House and Wells (1973), Spafford (1999), **?**
Percopsis transmontana	ap, am, Lp, Li, Wwb, Wwi, R_L, t-L	Gray and Dauble (1979)
Chologaster cornuta	ab, ap, am, Lp, Li, Wwb, Wwp, Wwi, Ri, t-L	Poulson (1963)
Forbesichthys agassizii	ab, ap, am, Lp, Li, Wwb, Wwp, Wwi, Ri, t-L	Poulson (1963)
Typhlichthys subterraneus	ab, ap, am, Lp, Li, Wwb, Wwp, Wwi, Ri, t-L	Poulson (1963)
Amblyopsis spelaea	ab, ap, am, Lp, Li, Wwb, Wwp, Wwi, Ri, t-L	Poulson (1963)
Amblyopsis rosae	ab, ap, am, Lp, Li, Wwb, Wwp, Wwi, Ri, t-L	Poulson (1963), White and Culver (2012)
Aphredoderus sayanus	ap, am, Lp, Li, Wwb, Wwi, Ri, t-L	McCallum (2012), Shepherd and Huish (1978)
Zeus faber	ah, ap, am, Lh, Lp, Li, Wwi, GSI, t-L, L-Ww	Ismen et al. (2013)
Boreogadus saida	ab, aj, ap, am, L0, Lj, Lp, Li, Ww0, Wwj, Wwp, Wwi, Ni, t-L_T, t-Le, t-Ww_T, t-Wwe, t-JX_T, L-JO_T, L-Ww, L-N, L0-Lt, Ww0-Wwt, L-X, T-JO	Bouchard et al. (2016), Craig et al. (1982), Dahlke et al. (2017), Fey and Węsławski (2017), Hop et al. (1997), Kent et al. (2016), Kunz et al. (2016), Nahrgang et al. (2014), Walkusz et al. (2011)
Eleginus gracilis	am, Lp, Li, Wwb, Wwp, Wwi, Ri, t-L, t-Ww	Datsky (2016)
Gadiculus argenteus	am, Lp, Li, Wwb, Wwi, Ri, t-L	Magnussen (2007), Munk and Nielsen (2005)

Continuation of Table B.1		
**species**	**data**	**references**
Gadus morhua	ah, ab, aj, ap, am, Lh, Lj, Lp, Li, Wwh, Wwj, Wwi, Ri, GSI, E0, t-L_T, t-Wd_T, t-Ww_f, L-Ww, t-p+, t-JOe, t-JNe, t-VY, t-E, t-Wwe, t-Wde, T-ab, L-N	Björnsson et al. (2007), Buslov et al. (2010), Finn et al. (1995a,b), Kjesbu et al. (1991, 1998), McCollum et al. (2006), Otterlei et al. (1999), Paulsen et al. (2009), Thorsen et al. (2010)
Gadus chalcogrammus	ab, am, Lp, Li, Wwb, Wwp, Wwi, R_L, t-L	Lai and Gunderson (1987)
Microgadus tomcod	am, Lp, Li, Wwb, t-L, t-Ww, L-N	Couillard et al. (2011), Schaner and Sherman (1960)
Micromesistius poutassou	am, Lp, Li, Wwb, Wwp, Wwi, Ri, t-L	Magnussen (2007)
Micromesistius australis	ab, ap, am, Lp, Li, Wwb, Wwp, Wwi, GSI, t-L	Contreras-Reyes et al. (2014)
Melanogrammus aeglefinus	ab_T, ap, am, Lp, Li, Wwb, Ww_L, Wwp, Wwi, Ri, t-L_f	Bolle et al. (2004)
Merlangius merlangus	ab, aj, ap, am, Lb, Lj, Lp, Li, Wwb, Wwp, Wwi, Ri, t-L, L-N, L-Ww	Hislop and Hall (1974), Jones and Hislop (1972), Last (1978), Povoa et al. (2011), Shaw et al. (2008)
Pollachius virens	ab, ap, am, Lp, Li, Wwb, Wwp, Wwi, R_L, t-L_f	Bolle et al. (2004)
Trisopterus luscus	ah, ab, am, Lh, Lp, Li, L-N, L-Ww	Alonso-fernandez et al. (2010), Metin et al. (2008)
Trisopterus minutus	am, Lb, Lp, Li, Wwb, Wwp, Wwi, Ri, t-L	Magnussen (2007), Munk and Nielsen (2005)
Merluccius merluccius	ab, aj, ap, am, Lp, Li, Wwb, Wwi, R_L, t-L, L-Ww	Pineiro and Sainza (2003)
Merluccius hubbsi	ab, ap, am, Lp, Li, Wwb, Wwp, Wwi, GSI, t-L	Betti et al. (2014), dos Santos and Rossi-Wongtschowski (2007), Rodrigues et al. (2018)
Merluccius polli	am, Lp, Li, Wwb, Wwp, Wwi, GSI, t-L	Rey et al. (2016)
Merluccius senegalensis	am, Lp, Li, Wwb, Wwp, Wwi, GSI, t-L	Rey et al. (2016)
Merluccius capensis	am, Lp, Li, Wwb, Wwp, Wwi, GSI, t-L	Wilhelm et al. (2017)
Macruronus novaezelandiae	ab, am, Lp, Li, Wwb, Wwi, Ri, t-L	Sweetman et al. (2018)
Coryphaenoides rupestris	am, Lp, Li, Wwb, Wwi, Ri, t-L	Allain and Lorance (2000)
Coryphaenoides acrolepis	am, Lp, Li, Wwb, Wwi, Ri, t-L	Andrews (1997)
Coelorinchus caelorhincus	am, Lp, Li, Wwb, Ri, t-L, t-Ww	Labropoulou and Papaconstantinou (2000)
Nezumia sclerorhynchus	am, Lp, Li, Wwb, Ri, t-L, t-Ww	Labropoulou and Papaconstantinou (2000)
Hymenocephalus italicus	am, Lp, Li, Wwb, Wwp, Wwi, Ri, t-L	D'Onchia et al. (2000)
Macrourus berglax	am, Lp, Li, Wwb, Wwi, Ri, t-L, t-Ww	Murua (2003), Orlova et al. (2018)
Macrourus carinatus	am, Lp, Li, Wwb, Wwi, Ri, t-L	Lee et al. (2019)
Trachyrincus scabrus	am, Lp, Li, Wwb, Wwi, Ri, t-L	Sion et al. (2012)
Raniceps raninus	am, Lb, Lp, Li, Wwb, Wwi, Ri, t-L, L-Ww	Deniel (1985), Munk and Nielsen (2005)

Continuation of Table B.1		
**species**	**data**	**references**
Phycis phycis	ap, am, Lp, Li, Wwb, Wwp, Wwi, Ri, t-L_f	Matić-Skoko et al. (2011), Vieira et al. (2014)
Phycis blennoides	am, Lp, Li, Wwb, Wwi, Ri, t-L_f	Casas and Pineiro (2000)
Brosme brosme	am, Lp, Li, Wwb, Wwp, Wwi, Ri, t-L	Magnussen (2007)
Ciliata mustela	ab, ap, am, Lp, Li, Ri, L-Ww, t-L	Badsha and Sainsbury (1978), Cohen et al. (1990), Smietana, P (1992)
Gaidropsarus guttatus	ap, am, Lp, Li, Wwb, Wwp, Wwi, GSI, t-L	Morato et al. (2003)
Lota lota	ah, ab, ap, am, Lh, Lb, Lp, Li, Wwb, Wwp, Wwi, Ri, t-L, L-Ww	Kupren et al. (2014), Smederevac-Lalić et al. (2015)
Molva molva	am, Lp, Li, Wwb, Wwp, Wwi, Ri, t-L	Magnussen (2007), Munk and Nielsen (2005)
Molva dypterygia	ap, am, Lp, Li, Wwb, Wwp, Wwi, Ri, t-L	Magnussen (2007), Munk and Nielsen (2005)
Pseudophycis bachus	am, Lp, Li, Wwb, Wwp, Wwi, Ri, t-L_f	Kemp et al. (2013)
Antimora rostrata	am, Lp, Li, Wwb, Ri, t-L, t-Ww	Orlova et al. (2018)
Polymixia nobilis	am, Lp, Li, Wwb, GSI, t-L, L-Ww	García-Mederos et al. (2010)
Hoplostethus atlanticus	am, Lp, Li, Wwb, Wwi, Ri, t-L	Allain and Lorance (2000)
Beryx splendens	am, Lp, Li, Wwb, Wwi, Ri, t-L_f	Leis and Carson-Ewart (2000), Rico et al. (2001)
Poromitra crassiceps	am, Lp, Li, Wwb, Wwp, Wwi, Ri, t-L	Childress et al. (1980)
Holocentrus adscensionis	ab, am, Lb, Lp, Li, Wwi, Ri, rB	Shinozaki-Mends et al. (2007)
Carapus bermudensis	ab, am, Lb, Lp, Li, Wwi, Ri, t-L	Glynn et al. (2008), Smith et al. (1981)
Genypterus blacodes	am, Lb, Lp, Li, Ww_L, Wwi, t-L, L-N, Ww-N	Baker et al. (2014), Crechriou et al. (2015), Paredes and Bravo (2005), Wiff et al. (2007)
Opsanus tau	ah, ap, am, Lb, Lp, Li, Wwi, R_L, t-L_T, t-Ww_T, L-Ww	Dovel (1960), Mensinger et al. (2001), Palazón-Fernaández et al. (2001), Swartz and Engel (1968)
Opsanus beta	am, Lp, Li, Wwb, Ri, t-L_T, L-Ww	Malca et al. (2009)
Halobatrachus didactylus	ab, ap, am, Lb, Lp, Li, L-Ww, L-N, Ww-N	Palazón-Fernaández et al. (2001)
Kurtus gulliveri	am, Lb, Lj, Lp, Li, Wwb, Wwi, Ri, t-L	BERRA and ADAY (2004), BERRA and Neira (2003)
Ostorhinchus doederleini	ab, aj, ap, am, Lj, Lp, Li, Wwb, Wwj, Wwp, Wwi, Ri, t-L	Kingsford et al. (2014)
Siphamia tubifer	aj, ap, am, Lj, Lp, Li, Wwb, t-L, L-Ww, L-N	Gould et al. (2016)
Perccottus glenii	am, Lp, Li, Wwb, Wwp, Wwi, Ri, t-L	Joanna et al. (2011)
Dormitator latifrons	am, Lp, Li, Wwb, Wwi, dWw, GSI, t-L	Basto-Rosales et al. (2019), Chang and Navas (1984)
Gobiomorus dormitor	am, Lp, Li, Wwb, Wwi, GSI, L-dL, L-Ww	Bacheler (2002), Bacheler et al. (2004)
Boleophthalmus pectinirostris	ab, am, Lp, Li, Wwb, Wwp, Wwi, Ri, t-L	Atsushi Nanamia (2005), Takegaki (2008), Wanshu and Qiyong (2004)

Continuation of Table B.1		
**species**	**data**	**references**
Gymnogobius urotaenia	ab, am, Lp, Li, Wwb, t-L, t-Ww, LN	Kolpakov and Deminab (2011)
Tridentiger bifasciatus	am, Lp, Li, Wwb, Wwi, t-L, L-N	Qin et al. (2020), Slater (2005)
Pomatoschistus minutus	ab, ap, am, Lb, Lp, Li, Wwb, Wwp, Wwi, Ri, t-L, L-Ww_f, L-N, T-JO_f, T-ab	Bouchereau et al. (1990, 1989), Fonds (1973), Freitas (2010), Gaas (1977), Penning de Vries (1969)
Rhinogobius brunneus	ab, ap, am, Lb, Lp, Li, Wwb, Wwp, Wwi, Ri, t-L	Heimowitz and Fuller (2007), Kondo et al. (2013)
Gobius niger	ap, am, Lp, Li, Wwb, Wwp, Wwi, Ri, t-L	Silva and Gordo (1997)
Gobius paganellus	ap, am, Lp, Li, Wwb, Wwp, Wwi, Ri, t-L	Azevedo and Simas (2000)
Gobiosoma bosc	ab, am, Lp, Li, Wwb, Wwp, Wwi, Ri, t-L	
Ponticola iranicus	am, Lp, Li, Wwb, Ri, t-L, t-Ww	Mohammadi-Darestani et al. (2016)
Neogobius melanostomus	am, Lp, Li, Wwb, Wwp, Ri, t-L, t-Ww	Shemonaev and Kirilenko (2009)
Knipowitschia caucasica	am, Lp, Li, Wwb, t-L, L-Ww, L-N	Güçü and Ömer Erdoğan (2017), Kevrekidis et al. (1990)
Hippocampus whitei	ab, ap, am, Lb, Li, Wwb, Wwi, Ri, t-L	Pitargue (2007), Vincent and Giles (2003), Wong and Benzie (2003)
Hippocampus kuda	ab, ap, am, Lp, Li, Wwb, Wwp, Wwi, Ri, t-L, t-Ww	Choo and Liew (2006), Saavedra et al. (2014)
Hippocampus guttulatus	ab, tp, am, Lb, Lp, Li, Wwb, Wwp, Wwi, Ri, L-L, t-Ww	Curtis and Vincent (2006), Planas et al. (2010)
Phyllopteryx taeniolatus	ab, am, Lp, Li, Wwb, Wwp, Wwi, Ri, t-L_f	Forsgren and Lowe (2006), Sanchez-Camara et al. (2005)
Macroramphosus scolopax	ab, ap, am, Lp, Li, Wwb, Wwp, Wwi, Ri, t-L	Borges (2000)
Macroramphosus gracilis	ab, ap, am, Lp, Li, Wwb, Wwp, Wwi, Ri, t-L	Borges (2000)
Upeneus moluccensis	am, Lp, Li, Wwb, Ri, t-L, L-Ww	Kaya et al. (1999)
Upeneus tragula	am, Lp, Li, Wwb, Ww_L, Ri, t-L	Pavlov et al. (2015)
Parupeneus multifasciatus	am, Lp, Li, Wwb, Ri, t-L, L-Ww	Pavlov et al. (2013)
Thunnus orientalis	ap, am, Lb, Lp, Li, t-L, L-Ww, L-N, T-ab	Jusup et al. (2011)
Thunnus thynnus	ap, am, Lb, Lp, Li, t-Le, t-L_f, t-Ww, L-Ww, t-N, T-ab	Aguado-Gimenez and Garcia-Garcia (2005), Jusup et al. (2011), Miyashita et al. (2001, 2000), Nisbet et al. (2012)
Thunnus maccoyii	am, Lp, Li, Wwb, Wwi, t-L	Gunna et al. (2008)
Thunnus obesus	ab, am, Lp, Li, Wwb, Wwi, Ri, t-L	da Silva et al. (2015)
Thunnus albacares	ab, am, Lp, Li, Wwb, Wwi, Ri, t-L	Eveson et al. (2015)
Thunnus alalunga	ab, am, Lp, Li, Wwb, Wwi, Ri, t-L	de Zárate and Babcock (2016)
Katsuwonus pelamis	ab, am, Lp, Li, Wwb, Wwi, Ri, t-L	Eveson et al. (2015)
Scomber scombrus	ah, ab, ap, am, Lh, Lb, Lp, Li, Wwb, Wwp, Wwi, Ri, t-L_T, t-Ww, Ww-N, L-Ww, L-Wd, T-ah	Damme (2007), Lockwood (1988), Mendiola et al. (2007, 2006)
Scomber japonicus	ab, am, Lp, Li, Wwb, Wwp, Wwi, Ri, t-L	Takahashi et al. (2005)

Continuation of Table B.1		
**species**	**data**	**references**
Scomberomorus commerson	ap, am, Lp, Li, Wwb, Wwp, Wwi, t-L_T, L-N	McIlwain et al. (2005), Noegroho et al. (2018)
Scomberomorus plurilineatus	ap, am, Lp, Li, Wwb, Wwp, Wwi, Ri, t-L	Chale-Matsau et al. (1999)
Scomberomorus sierra	ap, am, Lp, Li, Wwb, GSI, t-L, L-Ww	Chale-Matsau et al. (1999), Lucano-Ramírez et al. (2011), Nava-Ortega et al. (2012)
Trichiurus nanhaiensis	ab, am, Lp, Li, Wwb, Wwi, GSI, t-L	Kwok and Ni (1999, 2000)
Trichiurus lepturus	ab, am, Lp, Li, Wwb, Wwi, Ri, t-L	Narasimham (1976)
Trichiurus lepturus japonicus	ab, ap, am, Lb, Lp, Li, Wwb, Wwp, Wwi, Ri, GSI, t-L, t-Ww, L-Ww, Ww-N	Chai et al. (2015), Hong (1980), Kwok and Ni (1999), Li (1983), Luo et al. (1983), Shi et al. (2020), Wan and Meng (2003), Wu et al. (1985)
Aphanopus carbo	ab, ap, am, Lp, Li, Wwb, Wwi, Ri, t-L	Morales-Nin and Sena-Carvalho (1996)
Brama brama	am, Lp, Li, Wwb, Ri, t-L, L-Ww	Lobo and Erzini (2001), Richards (2003)
Pomatomus saltatrix	ab, am, Lp, Li, Wwb, Wwi, Ri, t-L	Robillard et al. (2009)
Promethichthys prometheus	am, Lp, Li, Wwb, Wwp, Wwi, Ri, t-L	Lorenzo and Pajuelo (1995), Robillard et al. (2009)
Mastacembelus erythrotaenia	ah, ab, ap, am, Lh, Lp, Li, Wwi, Ri	
Monopterus albus	ap, am, Lp, Li, Wwb, Wwp, Wwi, Ri, t-L, L-Ww	Susatyo et al. (2018), Yang and Xiong (2010)
Channa argus	ab, am, Lp, Li, Wwb, Wwp, Wwi, Ri, Ww-JX_T, Ww-r_T	Liu et al. (1998), Resh et al. (2018)
Channa marulius	am, Lp, Li, Wwb, Ri, t-L, t-Ww, L-Ww	Dua and Kumar (2006), Kilambi (1986)
Channa punctata	ab, ap, am, Lb, Lp, Li, L_t, Wwb, Wwp, Wwi, Ri	Saika et al. (2013)
Channa striata	am, Lp, Li, Wwb, Wwp, Wwi, Ri, t-L	Kilambi (1986)
Leptomelanosoma indicum	am, Lp, Li, Wwb, Wwi, Ri, t-L	Kadwade (1971), Kam and Leung (2002)
Filimanus heptadactyla	am, Lp, Li, Wwb, Wwp, Wwi, Ri, t-L	Prasad et al. (2005)
Eleutheronema tetradactylum	am, Lp, Li, Wwb, Wwi, Ri, t-L, L-Ww	Ballagh et al. (2012)
Polydactylus macrochir	am, Lp, Li, Wwb, Wwi, Ri, t-L_f, L-Ww	Moore et al. (2012)
Coryphaena hippurus	ab, aj, ap, am, Lb, L_t, Lj, Lp, Li, Wwi, Ri, t-L, L-Ww	Furukawa et al. (2012)
Trachurus trachurus	ah, ab, ap, am, Lh, Lb, Lp, Li, Wwb, Wwp, Wwi, Ri, t-L, L-Ww, T-ah	Abaunza et al. (2003), King et al. (1977), Pipe and Walker (1987)
Trachurus mediterraneus	ab, ap, am, Lp, Li, Wwb, Wwp, Wwi, Ri, t-L, L-Ww, T-ah	Karlou-Riga (2000)
Seriola dumerili	ah, ab, ap, am, Lh, Lb, Lp, Li, Wwi, GSI, t-L	Papandroulakis (2013), Papandroulakis et al. (2005b)
Echeneis naucrates	am, Lp, Li, Wwb, Wwi, Ri, t-L	Bachman et al. (2018)

Continuation of Table B.1		
**species**	**data**	**references**
Sphyraena barracuda	ap, am, Lp, Li, Wwb, Wwp, Wwi, GSI, t-L, L-Ww	Kadison et al. (2010)
Xiphias gladius	ab, ap, am, Lb, Lp, Li, Wwb, Wwp, Wwi, Ri, t-L_f, L-Ww	S.-P. Wang and Chiang (2010), S.-P. Wang and Yeh (2003)
Makaira nigricans	ab, ap, am, Lb, Lp, Li, Wwi, R_L, t-L, L-Ww	Martins et al. (2007), Sponaugle et al. (2005), Sun et al. (2013)
Istiompax indica	am, Lp, Li, Wwb, Wwi, Ri, t-L	Sun et al. (2015a,b)
Istiophorus platypterus	am, Lj, Lp, Li, Wwb, Wwi, Ri, t-L, L-Ww	Hoolihan (2006)
Centropomus undecimalis	ab, am, Lh, Lb, Lp, Li, Wwb, Wwi, Ri, t-L_f	Perera-Garcia et al. (2013)
Trinectes maculatus	am, Lp, Li, Wwb, Wwp, Wwi, Ri, t-L	
Solea senegalensis	ah, ab, aj, ap, am, L0, Lh, Lb, Lj, Lp, Li, Wd0, Wdh, Wdb, Wdj, Wwp, Wwi, Ri, E0, T-ah, T-aj, t-L_T, t-Wd_f, t-Ww, L-Ww, L-Wd	Canavate and Fernandez-Diaz (1999), Carballo et al. (2018), Dinis et al. (1999), Maulvault et al. (2019), Ortiz-Delgado et al. (2019), Parra and Yufera (2001), Ribeiro et al. (2017, 1999), Teixeira and Cabral (2010), Vinagre (2007), Yufera et al. (1999)
Solea solea	ah, ab, aj, ap_T, am, Lh, Lb, Lj, Lp, Li, Wd0, Wdh, Wdb, Wdj, Wwp, Wwi, Ri_L, E0, pAi, T-ah, T-ab, T-aj, t-L_T, t-Wd, L-Ww, L-N, L-JX_T	Day et al. (1996), Deniel (1981,?), Devauchelle et al. (1987), Dorel (1986), Fonds (1979), Fonds et al. (1989), Fonds and Saksena (1977), Galois et al. (1990), Lund et al. (2008), Wheeler (1978), Witthames and Greer Walker (1995), **?**), **?**), **?**), **?**
Dicologlossa cuneata	ab, ap, am, Lb, Lp, Li, Wwb, Wwp, Wwi, Ri, t-L, L-W	Boufersaoui and Saidani (2015), Boufersaoui and Zeroual (2018), Marinaro et al. (2007), Ouabadi (2015)
Microchirus azevia	ap, am, Lb, Lp, Li, Wwb, Wwp, Wwi, Ri, t-L, L-W	Boufersaoui and Zeroual (2018), Marinaro (1991)
Microchirus variegatus	am, Lp, Li, Wwb, Wwp, Wwi, Ri, t-L	Deniel (1990), Gibson and Ezzi (1980)
Pegusa lascaris	ab, am, Lp, Li, Wwb, Wwp, Wwi, Ri, t-L, L-Ww	Ahlstrom et al. (1984), Boufersaoui et al. (2021), Deniel (1990), Marinaro (1991)
Pegusa impar	am, Lp, Li, Wwb, Wwp, Wwi, Ri, t-L	Deniel (1990)
Buglossidium luteum	am, Lp, Li, Wwb, Wwp, Wwi, Ri, t-L	Castellani and Edwards (2017), Deniel (1990)
Lepidorhombus whiffiagonis	ab, am, Lp, Li, Wwb, Wwp, Wwi, Ri, t-L_f	Landa et al. (1996)
Lepidorhombus boscii	ab, am, Lp, Li, Wwb, Wwp, Wwi, Ri, t-L_f	Landa et al. (2002), Santos (1995)
Scophthalmus maximus	ah, ab, aj, ap, am, Lb, Lj, Lp, Li, Wwb, Wwp, Wwi, Ri, t-L_f, L-Ww, Ww-N	Jomes (1974), Jones (1972, 1973), van der Hammen (2011)
Bothus podas	ab, ap, am, Lj, Lp, Li, Wwb, Wwi, GSI, t-L	Morato et al. (2007)

Continuation of Table B.1		
**species**	**data**	**references**
Arnoglossus laterna	ab, am, Lj, Lp, Li, Wwb, Ri, t-L, t-Ww, L-Ww	Castellani and Edwards (2017), Gibson and Ezzi (1980), Ticina and Matic-Skoko (2012)
Arnoglossus thori	am, Lp, Li, Wwb, Wwp, Wwi, Ri, t-L	Deniel (1990)
Paralichthys californicus	aj, ap, am, Lb, Lj, Lp, Li, Wwb, Wwi, Ri, t-L_f	MacNair et al. (2001)
Paralichthys lethostigma	am, Lp, Li, Wwb, Wwp, Wwi, Ri, t-L	
Syacium gunteri	am, Lb, Lp, Li, Wwi, Ri, t-L	Sánchez-Iturbe et al. (2006)
Hippoglossus hippoglossus	ah, ab, ap, am, Lh, Lb, Lj, Lp, Li, Wwb, Wwi, R_L, t-L, t-Ww	Andriyashev (1964), Blaxter et al. (1983), Haug and Gulliksen (1988), Jakupsstovu and Haug (1988), Lein et al. (1997), Lønning et al. (1982), Munk and Nielsen (2005), Wienerroither et al. (2011)
Hippoglossus stenolepis	ab, aj, ap, am, Li, Wwb, Wwi, Ri, t-L, t-Ww	McFarlane et al. (1991), Orlov et al. (2011)
Reinhardtius hippoglossoides	am, Lb, Lj, Lp, Li, Wwb, Wwi, Ri, t-L	Dwyer et al. (2016)
Hippoglossoides elassodon	am, Lp, Li, Wwb, Wwp, Wwi, GSI, t-L	Forrester (1964), Stark (2004)
Glyptocephalus cynoglossus	ab, am, Lp, Li, Wwb, Wwp, Wwi, R_L, t-L	Bagenal (1963), Magnussen (2007)
Microstomus kitt	ab, am, Lp, Li, Wwb, Wwp, Wwi, Ri, t-L	Magnussen (2007)
Hypsopsetta guttulata	ap, am, Li, Wwb, Wwi, Ri, t-L, t-Ww	
Lepidopsetta polyxystra	am, Lp, Li, Wwb, Wwp, Wwi, GSI, t-L	Forrester (1964), Stark and Somerton (2002)
Lepidopsetta bilineata	am, Lp, Li, Wwb, Wwp, Wwi, GSI, t-L	Forrester (1964), Stark and Somerton (2002)
Pleuronectes platessa	ab, ap, am, Lb, Lp, Li, Wwb, Wwp, Wwi, R_L, t-L, t-N, L-Ww, L-N, Ww-N, Ww-pX_T, Ww-JO_T, T-ah, T-dL	Bolle et al. (2009), Deniel (1981), Fonds et al. (1992), Glazenburg (1983), Kennedy et al. (2007), Rijnsdorp and Ibelings (1989), Skjærven et al. (2003), Wheeler (1978)
Limanda limanda	am, Lp, Li, Wwb, Wwp, Wwi, Ri, t-L	Magnussen (2007)
Limanda ferruginea	ap, am, Lp, Li, Wwb, Wwi, Ri, t-L	Dwyer et al. (2003)
Platichthys stellatus	ah_T, ab, ap, am, Lh, Lb, Lj, Lp, Li, Wwb, Wwi, Ri, t-L, L-Ww	
Liopsetta pinnifasciata	am, Lp, Li, Wwb, Ri, t-L, t-Ww, L-Ww	Vdovin et al. (2017)
Eopsetta jordani	ab, am, Lp, Li, Wwb, Wwp, Wwi, Ri, t-L	Alderdice and Forrester (1971), Krüger (1969)
Ambassis agassizii	am, Lp, Li, Wwb, Wwp, Wwi, Ri, t-L	Milton and Arthington (1985)
Amatitlania nigrofasciata	am, Lp, Li, Wwb, Wwp, Wwi, t-L, Ww-N	Ishikawa and Tachihara (2010)
Amphilophus citrinellus	am, Lp, Li, Wwb, Wwi, Ri, t-Ww	Oldfield (2009)

Continuation of Table B.1		
**species**	**data**	**references**
Coptodon rendalli	ah, ab, ap, am, Lh, Lb, Lp, Li, Ww0, Wwi, Ri, L-Ww, t-L, Ww-N, T-JO, T-JX, t-dWw_T	Caulton (1977, 1982), Chandrasoma and De Silva (1981), Hlophe (2011), Kangombe (2004), Morioka and Matsumoto (2007), Philippart and Ruwet (1982), Weyl and Hecht (1998)
Coptodon zillii	am, Lp, Li, Wwb, Wwp, Wwi, Ri, t-Ww	Dikel et al. (2002)
Mayaheros beani	am, Lp, Li, Wwb, Wwp, Wwi, Ri, t-Ww	García-Lizárraga et al. (2011), Martinez-Cardenas et al. (2014)
Mayaheros urophthalmus	am, Lp, Li, Wwb, Wwp, Wwi, Ri, t-L	Faunce et al. (2002)
Oreochromis aureus	am, Lp, Li, Wwb, Wwp, Wwi, Ri, t-L	
Oreochromis mossambicus	am, Lp, Li, Wwb, Wwp, Wwi, Ri, t-L	
Oreochromis niloticus	am, Lb, Lp, Li, Wwb, Wwp, Wwi, Ri, t-Ww_f	Bhujel et al. (2007)
Pterophyllum scalare	ab, am, Wwb, Wwp, Wwi, t-Ww, Ww-N	Espitia-Manrique et al. (2017), Korzelecka-Orkisz et al. (2012), Ortega-Salas et al. (2009)
Oryzias latipes	ab, aj, ap, am, Lb, Lj, Lp, Li, Wd0, Wwb, Wdb, Wwp, Wwi, Ri, t-L, t-Ww_f, t-N_f, t-S	Ankley and Johnson (2004), Egami and Etoh (1969), Faber (2013), Ghoneum and Egami (1982), Gonzales-Doncel et al. (2016), Hasebe et al. (2016), Iwamatsu and Egami (2004), Kang et al. (2002), Marty et al. (1995), Spencer et al. (2006), Stanley (1972), Teh and Hinton (1998)
Belone belone	ah, am, Lh, L_t, Lp, Li, Wwi, t-L, L-Ww, L-N, Ww-N	Dulcic et al. (2009), Zorica and Kec (2013), Zorica et al. (2011)
Scomberesox saurus	ab, am, Lb, Lp, Li, Wwi, Ri, t-L	Agüera and Brophy (2012), Brownell (1983)
Hirundichthys affinis	ab, ap, am, Lb, Lp, Li, Wwi, Ri, t-L, L-N	Casazza et al. (2005), Oxenford et al. (1994)
Hyporhamphus picarti	am, Lp, Li, Wwb, Wwi, Ri, t-L_T	Döring et al. (2018), McBride and Thurman (2003)
Hyporhamphus australis	am, Lp, Li, Wwb, Wwi, Ri, t-L	Stewart and Hughes (2007)
Hyporhamphus regularis	am, Lp, Li, Wwb, Wwi, Ri, t-L	Stewart and Hughes (2007)
Arrhamphus sclerolepis	am, Lp, Li, Wwb, Wwi, Ri, t-L	Stewart and Hughes (2007)
Nothobranchius furzeri	ab, ap, am, Lb, Lp, Li, Wwb, Wwi, t-L_f, t-N_f	Berois et al. (2016), Blažek et al. (2013)
Nothobranchius kadleci	ab, ap, am, Lb, Lp, Li, Wwb, Wwi, t-L_f, t-N_f	Berois et al. (2016), Blažek et al. (2013)
Fundulopanchax gardneri	ab, ap, am, Lb, Li, L_t, Wwb, Wwi, Ww_t, Ri	Arimoro and Ofojekwu (2004)
Anablepsoides hartii	ap, am, Lb, Lp, Li, Wwi, Ni, t-L, t-Ww	Furness and Reznick (2014)
Kryptolebias marmoratus	ab, ap, am, Lb, Lp, Li, Ww_L, GSI, t-L_f	Gargeda et al. (2004), Parka et al. (2010), Sakakura and Noakes (2000)

Continuation of Table B.1		
**species**	**data**	**references**
Millerichthys robustus	ap, am, Lb, Lp, Li, Wwi, Ni, t-L, t-Ww	Dominguez-Castanedo et al. (2017)
Cynopoecilus melanotaenia	am, Lb, Lp, Li, Wwb, Wwp, Wwi, Ri, t-L	Arenzon et al. (1999, 2001)
Austrolebias adloffi	ap, Lb, Li, Ni, t-L_Tf, t-Ww_Tf, t-S	Liu and Walford (1966), Walford and Liu (1965)
Austrolebias viarius	ap, am, Lb, Li, Ni, t-L_Tf	Erreaa and Danulatb (2001)
Austrolebias wolterstorffi	ap, am, Lb, Li, Wwi, Ni, t-L_T, t-Ww_T	da Fonseca et al. (2013)
Fundulus catenatus	ap, am, Lp, Li, Wwb, Wwi, Ri, t-L	Wallus and Simon (2026)
Fundulus chrysotus	ap, am, Lt, Li, Wwb, Wwi, Ri,	
Fundulus diaphanus	am, Lp, Li, Wwb, Wwi, Ri, t-L	Edberg and Powers (2010), Phillips et al. (2007)
Fundulus dispar	ap, am, Lb, Lp, Li, Wwb, Wwi, Ri, t-L	Wallus and Simon (2026)
Fundulus julisia	ab, am, Lp, Li, Wwb, Wwi, Ri, t-L	Rakes (1989)
Fundulus grandis	am, Li, Wwb, Wwp, Wwi, Ri, t-L	Patterson et al. (2013), Vastano et al. (2017)
Fundulus heteroclitus	ab, am, Lp, Li, Wwb, Wwi, Ri, t-L	Kneib and Stiven (1978)
Fundulus kansae	am, Lp, Li, Wwb, Wwi, t-L, L-N	Minckley and Klaassen (1969)
Fundulus luciae	ab, ap, am, Lp, Li, Wwb, Wwi, Ri	Byrne (1976)
Fundulus notatus	am, Lp, Li, Wwb, Wwi, Ri, t-L	Nieman and Wallace (1974)
Fundulus parvipinnis	am, Lp, Li, Wwb, Wwi, Ri, t-Ww	H. Pérez-Espana and Abitia-Cárdenas (1998)
Fundulus seminolis	am, Lp, Li, Wwb, Wwi, GSI, t-L	Durant et al. (1979)
Fundulus stellifer	am, Lp, Li, Wwb, Wwi, Ri, t-L	Edberg and Powers (2010)
Empetrichthys latos	ap, am, Lp, Li, Wwb, L-Ww, L-N	Deacon and Williams (2010), Goodchild (2016), Uribe et al. (2012)
Crenichthys baileyi	ab, am, Lp, Li, Wwb, Wwi, dLb	Kopec (1949)
Valencia hispanica	am, Lp, Li, Wwb, Wwp, Wwi, Ri, dLb, t-L	Caiola et al. (2001)
Aphanius anatoliae	am, Lp, Li, Wwb, Wwi, Ri, t-L, L-Ww	Güçlü et al. (2007)
Aphanius danfordii	ap, am, Lp, Li, Wwb, Wwp, Wwi, Ri, t-L, t-Ww, L-Ww	Yoğurtçuoğlu and Ekmekçi (2012)
Aphanius dispar	ap, am, Lp, Li, Wwb, Wwp, Wwi, Ri, t-Ww	Frenkel and Goren (2000)
Aphanius fasciatus	ab, ap, am, Lb, Lp, Li, Wwp, Wwi, Ri, t-L, t-Ww, L-Ww, X-JX	Leonardos and Sinis (1998, 1999), Sezen and Olmez (2010)
Aphanius iberus	ab, am, Lp, Li, Wwb, Wwi, Ri, t-L	Fernandez-Delgado et al. (1988)
Aphanius mento	am, Lp, Li, Wwb, Wwi, Ri, t-L, L-Ww	Güçlü and Küçük (2008, 2011), S.A. (2020)
Cyprinodon bovinus	ab, ap, am, Lp, Li, Wwb, Wwi	Cripe et al. (2008a), EPA (2002), Jordan and Evermann (1900), Kennedy (1977)
Cyprinodon diabolis	ab, ap, am, Li, Wwb, Wwi, Ri, dLb	Deacon et al. (1995)
Cyprinodon elegans	ab, ap, am, Lp, Li, Wwb, Wwi, Ri	Leiser and Itzkowitz (2003)
Cyprinodon macularius	ab, ap, am, Lp, Li, Wwb, Wwi, Ri, dLb	Schoenherr (1988)
Cyprinodon nevadensis	ab, ap, am, Li, Wwb, Wwi, t-L_f	Lema and Nevitt (2006)

Continuation of Table B.1		
**species**	**data**	**references**
Cyprinodon variegatus	ab, aj, ap, am, Lb, Lj, Lp, Li, Wwb, Ri, t-L_f, t-Ww_f, L-Ww, t-N_f	Cripe et al. (2008b), EPA (2002), Foster (1967), Jordan and Evermann (1900), Page and Burr (1991), Parrish et al. (1978), Rosenfield and Sandoval-Green (2004), Schnitzler et al. (2017)
Jordanella floridae	am, Lp, Li, Wwb, Wwp, Wwi, Ri, t-L, t-Ww, T-ab	Beyger (2009), Foster et al. (1969), Mary et al. (2004)
Belonesox belizanus	ap, am, Lb, Lp, Li, Wwb, Wwp, Wwi, L-N	Turner and Snelson (1984)
Gambusia affinis	ab, aR_T, am, Lb, Lp, Li, Wwb, WwR, Wwi, Ri_T, t-Ww_Tf	Beadouin et al. (2008), Vondracek et al. (1988)
Gambusia puncticulata	ab, ap, am, Lb, Lp, Li, Wwb, Wwi, Ri, t-L	de León et al. (2013)
Gambusia punctata	ab, ap, am, Lb, Lp, Li, Wwb, Wwi, Ri, t-L	de León et al. (2013)
Gambusia holbrooki	ab, am, Lb, Lp, Li, Wwb, Wwi, Ri, t-L	Beadouin et al. (2008)
Heterandria formosa	ab, ap, am, Lb, Lp, Li, Wwb, Wwp, Wwi, Ri	Brown (2016), Henrich (1988)
Pseudoxiphophorus bimaculatus	am, Lp, Li, Wwb, Wwi, L-N, t-L	Olinger et al. (2016)
Girardinus metallicus	ab, ap, am, Lb, Lp, Li, Wwb, Wwi, Ri, t-L	de León et al. (2013)
Girardinus uninotatus	ab, ap, am, Lb, Lp, Li, Wwb, Wwi, Ri, t-L	de León et al. (2013)
Girardinus creolus	ab, ap, am, Lb, Lp, Li, Wwb, Wwi, Ri, t-L	de León et al. (2013)
Girardinus denticulatus	ab, ap, am, Lb, Lp, Li, Wwb, Wwi, Ri, t-L	de León et al. (2013)
Girardinus microdactylus	ab, ap, am, Lb, Lp, Li, Wwb, Wwi, Ri, t-L	de León et al. (2013)
Girardinus falcatus	ab, ap, am, Lb, Lp, Li, Wwb, Wwi, Ri, t-L	de León et al. (2013)
Quintana atrizona	ab, ap, am, Lb, Lp, Li, Wwb, Wwi, Ri, t-L	de León et al. (2013)
Limia vittata	ab, ap, am, Lb, Lp, Li, Wwb, Wwi, Ri, t-L	de León et al. (2013)
Poecilia latipinna	ab, ap, am, Lb, Lp, Li, Wwb, Wwp, Wwi, Ri, t-L	Moshayedi et al. (2015), Travis et al. (1989)
Poecilia reticulata	ab, ap, am, Lb, Li, Wwb, Wwi, Ri, t-L_f, t-S_f	Comfort (1983), Garc'ia et al. (2008), Ursin (1967)
Poecilia reticulata HPO	ab_f, Li, Wdb_f, JO, t-Ww_f, t-L_f, t-N_f, L-Ww_f, t-S_f	Auer et al. (2018), Reznick et al. (2004)
Poecilia reticulata HP Cur96	ab_f, L_i, t-Ww_f, t-L_f, t-N_f, L_Ww_f	Reznick (1996)
Poecilia reticulata HP Mad91	ab_f, Wdb_f, Li, t-Ww_f, t-L_f, t-N_f, L_Ww_f	Reznick and Bryant (1996)
Poecilia reticulata HP Mrn91	ab_f, Wdb_f, t-Ww_f, t-L_f, t-N_f, L_Ww_f	Reznick and Bryant (1996)
Poecilia reticulata HP Mrn96	ab_f, Li, t-Ww_f, t-L_f, t-N_f, L_Ww_f	Reznick (1996)
Poecilia reticulata HPY	ab_f, Li, Wdb_f, JO, t-Ww_f, t-L_f, t-N_f, L-Ww_f, t-S_f	Auer et al. (2018), Reznick et al. (2004)
Poecilia reticulata HP Yar91	ab_f, Wdb_f, Li, t-Ww_f, t-L_f, t-N_f, L_Ww_f	Reznick and Bryant (1996)

Continuation of Table B.1		
**species**	**data**	**references**
Poecilia reticulata HP Yar96	ab_f, Li, t-Ww_f, t-L_f, t-N_f, L_Ww_f	Reznick (1996)
Poecilia reticulata LPO	ab_f, Li, Wdb_f, JO, t-Ww_f, t-L_f, t-N_f, L-Ww_f, t-S_f	Auer et al. (2018), Reznick et al. (2004)
Poecilia reticulata LP Mad91	ab_f, Wdb_f, Li, t-Ww_f, t-L_f, t-N_f, L_Ww_f	Reznick and Bryant (1996)
Poecilia reticulata LP Mrn91	ab_f, Wdb_f, Li, t-Ww_f, t-L_f, t-N_f, L_Ww_f	Reznick and Bryant (1996)
Poecilia reticulata LP Mrn96	ab_f, Li, t-Ww_f, t-L_f, t-N_f, L_Ww_f	Reznick (1996)
Poecilia reticulata LP Par96	ab_f, Li, t-Ww_f, t-L_f, t-N_f, L_Ww_f	Reznick (1996)
Poecilia reticulata LP PaT96	ab_f, Li, t-Ww_f, t-L_f, t-N_f, L_Ww_f	Reznick (1996)
Poecilia reticulata LPY	ab_f, Li, Wdb_f, JO, t-Ww_f, t-L_f, t-N_f, L-Ww_f, t-S_f	Auer et al. (2018), Reznick et al. (2004)
Poecilia reticulata LP Yar91	ab_f, Wdb_f, Li, t-Ww_f, t-L_f, t-N_f, L_Ww_f	Reznick and Bryant (1996)
Xiphophorus helleri	ap, am, Lb, Lp, Li, Wwb, Wwp, Wwi, Ri, t-Ww	James and Sampath (2003), Moshayedi et al. (2015)
Xiphophorus maculatus	ab, am, Lb, Lp, Li, Wwb, Wwp, Wwi, Ri, t-L	Felin (1951), Moshayedi et al. (2015)
Xiphophorus montezumae	am, Lb, Lp, Li, Wwb, Wwp, Wwi, Ri, t-L	Kruesi et al. (2011), Moshayedi et al. (2015)
Atherina presbyter	ab, ap, am, Lb, Lp, Li, Wwb, Wwp, Wwi, Ri, t-L	Moreno and Morales-Nin (2003), Pajuelo and Lorenzo (2000), Palmer and Culley (2006), Turnpenny et al. (1981)
Atherina boyeri	am, Lp, Li, Wwb, Wwp, Wwi, Ri, t-L	Dulčić et al. (2008), Leonardos and Sinis (2000), Pajuelo and Lorenzo (2000)
Labidesthes sicculus	ap, am, Lp, Li, Wwb, Wwi, Ri, t-L	Powles and Sandeman (2008)
Leuresthes tenuis	ab, am, Lp, Li, Wwb, Wwp, Wwi, t-L, L-Ww, L-N	Clark (1925)
Menidia beryllina	ab, ap, am, Lp, Li, Wwb, Ri, t-L, t-Ww	Conover and Ross (1982), EPA (2002), Huber and Bengtson (1999), Letcher and Bengtson (1993)
Menidia extensa	ab, ap, am, Lb, Lp, Li, Wwb, Wwp, Wwi, Ri	Anonymous (2001), EPA (2002)
Menidia menidia	ab_T, am, Lp, Li, Wwb, Ri, t-L, t-Ww	Bengsten et al. (1987), Conover and Ross (1982)
Membras martinica	ab, am, Lj, Lp, Li, Wwb, Wwp, Wwi, Ri, dLj, t-L	Martin and Drewry (1978)
Odontesthes argentinensis	am, Lp, Li, Wwb, Wwp, Wwi, Ri, t-L	EPA (2002), Llompart et al. (2013)

Continuation of Table B.1		
**species**	**data**	**references**
Chromis chromis	ab, aj, ap, am, Lb, Lj, Lp, Li, Wwp, Wwi, Ri, t-L, L-Ww, Ww-JO	Alessandro (2014), Dulcic and Kraljevic (1995), Dulcic et al. (1994), Dulčić et al. (2005), Giacoletti (2016), Kingsford (1985), Macpherson and Raventos (2005), Valle et al. (2003)
Pomacentrus amboinensis	ab, ap, am, Lb, Lj, Lp, Li, Wwb, Wwj, Wwp, Wwi, Ri, t-L	Jones and McCormick (2002), Kingsford et al. (2017), Murphy et al. (2007)
Pomacentrus coelestis	ab, aj, ap, am, Lb, Lj, Lp, Li, Wwb, Wwj, Wwp, Wwi, Ri, t-L_f	Kingsford et al. (2017)
Chelon labrosus	ah, ab, ap, am, Lh, Lb, Lp, Li, Wwi, R_L, t-L, t-Ww	Leijzer (2006), Zouiten et al. (2008)
Chelon ramada	am, Lp, Li, Wwb, Wwp, Wwi, Ri, t-L	Koutrakis and Sinis (1994)
Chelon saliens	am, Lp, Li, Wwb, Wwp, Wwi, R_i, t-L	Koutrakis and Sinis (1994)
Mugil cephalus	am, Lp, Li, Wwb, Wwp, Wwi, R_i, t-L, L-Ww	nez Aguirre et al. (1999)
Mugil curema	am, Lp, Li, Wwb, Wwp, Wwi, Ri, t-L, L-Ww	nez Aguirre et al. (1999)
Mugil liza	ab, am, Lp, Li, Wwb, Wwi, R_i, t-L, t-Ww	Garbina et al. (2014), Monteiro-Ribas and Bonecker (2001)
Hyperprosopon argenteum	am, Lb, Lp, Li, Wwi, t-L, Ww-N	DeMartini et al. (1983)
Hysterocarpus traskii	am, Lp, Li, t-L, Ww-N	Bundy (1970)
Pseudochromis flavivertex	ab, am, Lb, Lp, Li, Wwb, Wwp, Wwi, Ri, t-L	Mies et al. (2004), Olivotto et al. (2006)
Lepadogaster lepadogaster	ab, ap, am, Lb, L_t, Lp, Li, Wwi, Ri	Tojeira et al. (2012)
Parablennius ruber	ab, am, Lb, Lj, Lp, Li, Wwb, Wwi, GSI, t-L	Azevedo and Homem (2002), Faria et al. (2010)
Lipophrys pholis	am, Lb, Lp, Li, Wwb, Wwi, GSI, t-L	Carvalho et al. (2012), Faria et al. (1996)
Enneapterygius atriceps	am, Lp, Li, Wwb, t-L, L-Ww, L-N	Longeneckera and Langston (2005)
Forsterygion lapillum	ah, ab, aj, ap, am, Lh, Lj, Lp, Li, GSI, rB, t-L, L-W, T-JO	Foch (2018), Francis (2001), Fricke (1994), J.R and N.A (2012), McArley et al. (2017), McDermott and Shima (2006), Mensink (2014), Nagelkerken (2017), Shima and Swearer (2009), Taylor and Willis (1998)
Gerres equulus	am, Lp, Li, Wwb, Wwp, Wwi, t-L, L-N	Iqbal et al. (2006, 2008)
Gerres oyena	am, Lp, Li, Wwb, Wwp, Wwi, t-L, L-N	H.A. Lamtane and and Ndaro (2007), Kanak and Tachihara (2006)
Ammodytes marinus	ab, am, Lp, Li, Wwb, Ri, t-L_f, t-Ww_f	Bergstad et al. (2002), Boulcott et al. (2017), Régnier et al. (2018)
Pseudopercis semifasciata	am, Lp, Li, Wwb, Wwp, Wwi, Ri, t-L_f	Gonzáalez (2006)
Uranoscopus scaber	am, Lp, Li, Wwb, Wwp, Wwi, t-L, t-Ww, L-N, Ww-N	Çoker et al. (2008), Demirhan et al. (2007)
Achoerodus viridis	ab, ap, am, Lb, Lp, L_t, Li, Wwp, Wwi, Ri	Gillanders (1995), Jeffrey (2007)

Continuation of Table B.1		
**species**	**data**	**references**
Semicossyphus pulcher	ab, ap, am, Lb, Lp, Li, Wwp, Wwi, Ri, t-L	Alonzo et al. (2004), Jirsa et al. (2007)
Symphodus tinca	am, Lp, Li, Wwb, Wwi, Ri, t-L	Boughamou et al. (2015), Gordoa et al. (2000)
Symphodus roissali	am, Lp, Li, Wwb, Wwp, Wwi, Ri, t-L	Gordoa et al. (2000), Jirsa et al. (2007), Raventos and Planes (2009)
Symphodus melops	am, Lp, Li, Wwb, GSI, t-L, t-Ww	Sayer et al. (1996)
Coris julis	am, Lp, Li, Wwb, Wwp, Wwi, t-L, L-N	Alonso-Fernádez and Palmer (2014), Gordoa et al. (2000)
Labrus merula	am, Lp, Li, Wwb, Wwp, Wwi, Ri, t-L	Gordoa et al. (2000), Jakov et al. (1999)
Bodianus frenchii	am, Lp, Li, Wwb, Wwp, Wwi, t-L_f, L-N_f	Cossington et al. (2010)
Centrolabrus exoletus	am, Lp, Li, Wwb, GSI, t-L, t-Ww	Sayer et al. (1996)
Ctenolabrus rupestris	ab, am, Lp, Li, Wwb, GSI, t-L, t-Ww	Sayer et al. (1995)
Tautogolabrus adspersus	am, Lb, Lp, Li, Wwb, t-L, L-N, L-Ww	Nitschke and Mather (2001), Serchuk and Cole (1974)
Scarus rivulatus	am, Lp, Li, Wwb, Wwp, Wwi, Ri, t-L, t-Ww	Lou (1993)
Sillago robusta	am, Lp, Li, Wwb, Wwp, Wwi, Ri, t-L	Hyndes and Potter (1996)
Sillago bassensis	am, Lp, Li, Wwb, Wwp, Wwi, Ri, t-L	Hyndes and Potter (1996)
Dicentrarchus labrax	ah_T, ab_T, aj, am, Lh, Lb, Lj, Li, Wwb, Wwp, Wwi, t-L, t-Ww_T, Ww-R, Ww-JX_T, T-JN_W	Anonimous (2008), Lika et al. (2014, 2015), MAYER et al. (1990), Papandroulakis (2012), Papandroulakis et al. (2014), Ruyet et al. (2004), Zanuy and Carrillo (1985)
Morone americana	am, Lp, Li, Wwb, Ri, t-L, t-Ww	Krüger (1965)
Morone chrysops	ab, am, Lp, Li, Wwb, Wwi, Ri, t-L	
Morone mississippiensis	ab, am, Lp, Li, Wwb, Wwi, Ri, t-L	Monteleone and Houde (1990), Zervas (2010)
Morone saxatilis	ab, am, Lp, Li, Wwb, Wwi, Ri, t-L	
Platax orbicularis	ab, aj, am, Lb, Lj, Lp, Li, Wwb, Wwj, Wwp, Wwi, Ri, t-Ww	Remoissenet et al. (2012)
Chaetodon larvatus	ab, am, Lp, Li, Wwb, Wwp, Wwi, GSI, t-L	Leis (1989), Zekeria (2003), Zekeria et al. (2006)
Aplodinotus grunniens	ap, am, Lp, Li, Wwb, Wwp, Wwi, Ri, t-L	
Argyrosomus regius	ah, ab, aj, ap, am, L0, Lh, Lb, Lj, Lp, Li, Ww0, Wwb, Wwp, Wwi, GSI, t-L_fT, t-Le, t-Ww_fT, L-Ww, Ww-R, Ww-JO_T	Kir et al. (2017), Klimogianni et al. (2013), Lika et al. (2014), Mylonas et al. (2013), Papadakis et al. (2013), Papandroulakis (2013), Vargas-Chacoff et al. (2014)
Argyrosomus japonicus	ab, am, Lp, Li, Wwb, Wwi, Ri, t-L	Lika et al. (2014), Silberschneider et al. (2009)
Atractoscion nobilis	am, Lp, Li, Wwb, Wwp, Wwi, Ri, t-L	Anonymous (2001), Romo-Curiel et al. (2015)
Cynoscion nebulosus	am, Lp, Li, Wwb, Wwi, Ri, t-L_f	Bortone (2003), Nieland et al. (2002), Smith et al. (2008)

Continuation of Table B.1		
**species**	**data**	**references**
Cynoscion othonopterus	am, Lp, Li, Wwb, Wwi, Ri, t-L_f	Aragón-Noriega (2014), Bortone (2003)
Larimichthys polyactis	ab, ap, am, Lb, Lp, Li, Wwb, Wwp, Wwi, Ri, GSI, t-L, t-Ww, L-Ww, Ww-N	Jiang et al. (2018), Li et al. (2011), Lim et al. (2010), Ma et al. (2017), Masuda et al. (1984), Shui (2000), Yan et al. (2006, 2014), Zhan et al. (2016), Zhang et al. (2010)
Macrodon atricauda	ap, am, Li, Wwb, Ri, t-L, L-Ww	Cardoso and Haimovici (2011)
Micropogonias undulatus	am, Lp, Li, Wwb, Wwp, Wwi, GSI, t-L	Barbieri et al. (1994), Hales and Reitzh (1992)
Pogonias cromis	am, Lj, Lp, Li, Wwb, Wwi, Ri, t-L	Olsen et al. (2018), Richards (2005)
Sciaena umbra	ah, ab, aj, ap, am, Lh, Lb, Lp, Li, Wwi, GSI, t-L	Lika et al. (2014), Papandroulakis (2013)
Stellifer lanceolatus	ap, am, Lp, Li, Wwb, Ri, t-L, L-Ww	Ramos-Miranda et al. (2009)
Totoaba macdonaldi	ap, am, Lp, Li, Wwb, Wwp, Wwi, Ri, t-L	Cisneros-Mata et al. (1995)
Umbrina canariensis	am, Lp, Li, Wwb, Wwp, Wwi, Ri, t-L	Hutchings et al. (2006)
Acanthurus lineatus	am, Lp, Li, Wwb, Wwp, Wwi, Ri, t-L	Choat and Axe (1996), Tucker (1998)
Acanthurus olivaceus	am, Lp, Li, Wwb, Wwp, Wwi, Ri, t-L	Choat and Axe (1996), Tucker (1998)
Ctenochaetus striatus	am, Lp, Li, Wwb, Wwp, Wwi, Ri, t-L	Choat and Axe (1996), Leis (1989)
Ctenochaetus binotatus	am, Lp, Li, Wwb, Wwp, Wwi, Ri, t-L, t-Ww	Lou (1993)
Zebrasoma scopas	am, Lp, Li, Wwb, Wwp, Wwi, Ri, t-L	Choat and Axe (1996), Leis (1989)
Naso brevirostris	am, Lp, Li, Wwb, Wwp, Wwi, Ri, t-L	Choat and Axe (1996), Leis (1989)
Naso hexacanthus	am, Lp, Li, Wwb, Wwp, Wwi, Ri, t-L	Choat and Axe (1996), Leis (1989)
Naso tuberosus	am, Lp, Li, Wwb, Wwp, Wwi, Ri, t-L	Choat and Axe (1996), Leis (1989)
Naso unicornis	am, Lp, Li, Wwb, Wwp, Wwi, Ri, t-L	Choat and Axe (1996), Leis (1989)
Naso vlamingii	am, Lp, Li, Wwb, Wwp, Wwi, Ri, t-L	Choat and Axe (1996), Leis (1989)
Prionurus maculatus	am, Lp, Li, Wwb, Wwp, Wwi, Ri, t-L	Choat and Axe (1996), Leis (1989)
Pomacanthus imperator	aj, am, Lp, Li, Wwb, Wwi, Ri, t-L, L-Ww, L-Ww	Arellano-Martínez et al. (2007), Chung and Woo (1999), Thresher and Brothers (1985)
Pomadasys kaakan	ab, am, Lp, Li, Wwb, Wwp, Wwi, Ri, t-L	Al-Husaini et al. (2002)
Conodon nobilis	am, Lp, Li, Wwb, Wwp, Wwi, Ri, t-L	Pombo et al. (2014)
Rhomboplites aurorubens	ab, am, Lp, Li, Wwb, Wwp, Wwi, t-L, Ww-N	Allman (2007), Cuellar et al. (1996)
Lutjanus analis	ab, am, Lp, Li, Wwb, Ri, t-L, L-Ww	Burton (2002)
Lutjanus fulviflamma	am, Lp, Li, Wwb, Wwp, Wwi, GSI, t-L	Grandcourt et al. (2006), Hamamoto et al. (1992)
Lutjanus guttatus	am, Lp, Li, Wwb, Wwp, Wwi, Ri, t-L	Amezcuaa et al. (2006), Hamamoto et al. (1992)
Lutjanus erythropterus	am, Lp, Li, Wwb, Wwi, GSI, t-L	McPherson et al. (1992), Newman et al. (2000)
Lutjanus malabaricus	am, Lp, Li, Wwb, Wwi, GSI, t-L	McPherson et al. (1992), Newman et al. (2000)

Continuation of Table B.1		
**species**	**data**	**references**
Lutjanus sebae	am, Lp, Li, Wwb, Wwi, GSI, t-L	McPherson et al. (1992), Newman et al. (2000)
Lutjanus carponotatus	am, Lp, Li, Wwb, Wwi, Ri, t-L	Newman et al. (2000)
Lutjanus vitta	ab, am, Lp, Li, Wwb, Wwi, Ri, t-L	Newman et al. (2000)
Lutjanus synagris	ab, am, Lp, Li, Wwb, Wwi, Ri, t-L	Aschenbrenner et al. (2017)
Lutjanus kasmira	ab, am, Lp, Li, Wwb, Wwi, Ri, t-L	Morales-Nin and Ralston (1990)
Caulolatilus affinis	am, Lp, Li, Wwb, Wwi, Ri, t-L	Ramírez-Pérez et al. (2011)
Lopholatilus chamaeleonticeps	am, Lp, Li, Wwb, Wwi, Ri, t-L, L-Ww	Dawson (2021)
Lobotes surinamensis	ab, am, Lp, Li, Wwb, Wwp, Wwi, Ri, t-L, t-Ww, L-Ww	Franks et al. (2001), Mickle and many others (2016)
Boops boops	ab, ap, am, Lb, Lp, Li, Wwb, Wwp, Wwi, Ri, t-L, L-Ww	Boufersaoui and Tamourt (2018), Boufersaoui and Zeroual (2018), Houria (2015)
Lagodon rhomboides	am, Lp, Li, Wwb, Wwp, Wwi, Ri, t-L	Nelson (2002)
Sarpa salpa	am, Lp, Li, Wwb, Wwp, Wwi, Ri, t-L	van der Walt and Beckley (1997)
Spicara smaris	am, Lp, Li, Wwb, Wwp, Wwi, Ri, t-L	Tsangridis and Filippousis (1994)
Argyrozona argyrozona	am, Lp, Li, Wwb, Wwp, Wwi, Ri, t-L	Brouwer and Griffiths (2004)
Dentex dentex	ah, ab, aj, ap, am, Lh, Lb, Lj, Lp, Li, Wwi, GSI, t-L	Lika et al. (2014), Papandroulakis (2013)
Pagellus erythrinus	ah, ab, aj, ap, am, Lh, Lb, Lp, Li, Wwi, GSI, t-L	Lika et al. (2014), Papandroulakis (2013)
Pagellus acarne	ab, ap, am, Lb, Lp, Li, Wwb, Wwp, Wwi, Ri, t-L, L-W	Boufersaoui (2016), Gaetani (1935)
Lithognathus mormyrus	ab, ap, am, Lb, Lp, Li, Wwb, Wwp, Wwi, Ri, t-L, L-W	Boufersaoui (2016), Divanach et al. (1983)
Acanthopagrus berda	ab, ap, am, Lb, Lp, Li, Wwb, Wwi, GSI	Connell (1996), Garratt (1993), Mathews and Samuel (1991), Papandroulakis (2013), Sheaves et al. (1999), Smith and Smith (1986)
Acanthopagrus schlegelii	ap, am, Lp, Li, Wwb, Wwi, GSI, t-L	Law and de Mitcheson (2018)
Archosargus probatocephalus	ab, ap, am, Lp, Li, Wwb, Wwp, Wwi, Ri, t-L, t-Ww	Beckman et al. (1991)
Diplodus puntazzo	ah, ab, aj, ap, am, Lh, Lb, Lj, Li, Wwi, GSI, t-L	Lika et al. (2014), Papandroulakis (2013), Papandroulakis et al. (2005a)
Diplodus sargus	ab, am, Lh, Lp, Li, Wwb, Wwi, Ri, t-L	Gordoa and Molí (1997)
Diplodus annularis	ab, am, Lh, Lp, Li, Wwb, Wwi, Ri, t-L	Gordoa and Molí (1997)
Diplodus vulgaris	ab, am, Lp, Li, Wwb, Wwi, Ri, t-L	Gordoa and Molí (1997)
Diplodus cervinus	ab, am, Lp, Li, Wwb, Wwp, Wwi, Ri, t-L	Pajuelo et al. (2003)
Pagrus auriga	ab, am, Lp, Li, Wwb, Wwi, GSI, t-L	Pajuelo et al. (2006a,b)
Rhabdosargus sarba	am, Lp, Li, Wwb, Wwp, Wwi, Ri, t-L	Radebe et al. (2002)
Sparus aurata	ah, ab, aj, ap, am, Lh, Lb, Lp, Li, Wwb, Wwj, Wwp, Wwi, GSI, t-L	Anonimous (2008), Kraljevic and Dulcic (1997), Lika et al. (2014), Papandroulakis (2013)

Continuation of Table B.1		
**species**	**data**	**references**
Centracanthus cirrus	am, Lp, Li, Wwb, Ri, t-L	Ozaydin et al. (2000)
Siganus sutor	am, Lp, Li, Wwb, Wwp, Wwi, t-L, Ww-N	A.Al-Marzouqi et al. (2011), Nelson et al. (1992)
Siganus rivulatus	ab, am, Lp, Li, Wwb, Wwp, Wwi, Ri, t-L	A.Al-Marzouqi et al. (2011), Bilecenoglu and Kaya (2002)
Scatophagus argus	am, Lp, Li, Wwb, Wwp, Wwi, t-L, L-N	Gandhi (1998), Gupta (2016)
Priacanthus macracanthus	am, Lp, Li, Wwb, Wwp, Wwi, GSI, t-L	Joung and Chen (1992), Liu et al. (2001)
Cepola macrophthalma	am, Lp, Li, Wwb, Ri, t-L, L-Ww	Rodríguez et al. (2017), Stergiou et al. (1992)
Capros aper	ap, am, Lp, Li, Wwb, Wwp, Wwi, Ri, t-L	Hussy et al. (2012)
Lophius piscatorius	ah, ab, ap, am, Lh, Lp, Li, Wwi, Ri, t-L, L-Ww	Dorel (1986), Hislop et al. (2001), Ofstad et al. (2013)
Lophius budegassa	am, Lp, Li, Wwb, Wwi, Ri, t-L, t-Ww	Landa et al. (2001)
Lophius vomerinus	am, Lp, Li, Wwb, GSI, t-L, L-Ww	Maartens et al. (1999)
Balistes vetula	am, Lp, Li, Wwb, Wwi, Ri, t-L, L-Ww	de Albuquerque et al. (2011), Simmons (2008)
Stephanolepis hispidus	am, Lp, Li, Wwb, Wwp, Wwi, Ri, t-L	Mancera-Rodríguez and Castro-Hernández (2004, 2015)
Mola mola	am, Lb, Lp, Li, Wwb, Ww_t, Wwi, R_L	Pope et al. (2010), Schmidt (1921)
Masturus lanceolatus	am, Lp, Li, Wwb, R_L, t-L, L-Ww	Liua et al. (2009), Pope et al. (2010)
Sphoeroides maculatus	am, Lp, Li, Wwb, Wwi, t-L, L-Ww, L-N	Lyczkowski-Shultz (1971), Merriner and Laroche (1977)
Epigonus crassicaudus	am, Lp, Li, Wwb, Wwi, Ri, t-L	Contreras-Reyes and Arellano-Valle (2013), Okamoto et al. (2011)
Pempheris adusta	am, Lp, Li, Wwb, Wwp, Wwi, Ri, t-L	Koeda et al. (2013, 2016)
Pempheris schwenkii	am, Lp, Li, Wwb, Wwp, Wwi, t-L, L-N	Koeda et al. (2013, 2016, 2012)
Polyprion americanus	am, Lp, Li, Wwb, Wwp, Wwi, Ri, t-L	Papadaki et al. (2018), Peres and Haimovici (2004)
Stereolepis gigas	aj, am, Lj, Lp, Li, Wwb, Wwi, Ri, t-L_f	Benseman and Allen (2018), Hawk and Allen (2014)
Scorpis aequipinnis	ap, am, Lp, Li, Wwb, Wwp, Wwi, Ri, t-L	Coulson et al. (2012)
Elassoma zonatum	ab, am, Lp, Li, Wwb, Wwp, Wwi, Ri, t-L	
Acantharchus pomotis	am, Lp, Li, Wwb, Wwp, Wwi, Ri, t-L_f, t-Ww_f	Pardue (1993)
Centrarchus macropterus	am, Lp, Li, Wwb, Wwp, Wwi, Ri, t-L	
Enneacanthus chaetodon	am, Lp, Li, Wwb, Wwp, Wwi, Ri, t-L, L-Ww	Schwartz (1961)
Enneacanthus gloriosus	am, Lp, Li, Wwb, Wwp, Wwi, Ri, t-L_f	Snyder and Peterson (1999)
Pomoxis annularis	am, Lp, Li, Wwb, Wwp, Wwi, Ri, t-L	
Pomoxis nigromaculatus	ab, am, Lp, Li, Wwb, Wwi, Ri, t-L	Pope et al. (1996), Pope and Willis (1998)
Archoplites interruptus	am, Lp, Li, Wwb, t-L, L-Ww, L-N	Vigg and Kucera (1981)
Ambloplites rupestris	am, Lp, Li, Wwb, Wwp, Wwi, Ri, t-L	Ryan and Harvey (1971)

Continuation of Table B.1		
**species**	**data**	**references**
Micropterus coosae	am, Lp, Li, Wwb, Wwp, Wwi, Ri, t-L_f, L-Ww	Knight (2011), Parsons (1954)
Micropterus dolomieu	ab, ap, am, Lb, Lp, Li, Wwb, Wwp, Wwi, Ri, t-Ww	Welsh et al. (2017)
Micropterus punctulatus	am, Lp, Li, Wwb, Wwp, Wwi, Ri, t-L	
Micropterus salmoides	ab, am, Lp, Li, Wwb, Wwi, Ri, t-L	Gash and Bass (1973)
Micropterus treculii	am, Lp, Li, Wwb, Wwp, Wwi, Ri, t-L	
Lepomis auritus	am, Lp, Li, Wwb, Wwp, Wwi, Ri, t-L	
Lepomis cyanellus	ab, ap, am, Lb, Lp, Li, Wwb, Wwp, Wwi, Ri, t-L	
Lepomis gulosus	ab, am, Lp, Li, Wwb, Wwi, Ri, t-L	Gash and Bass (1973)
Lepomis humilis	am, Lp, Li, Wwb, Wwp, Wwi, Ri, t-L	Barney and Anson (1923)
Lepomis macrochirus	ab, ap, am, Lb, Li, Wwp, Wwi, Ri, t-L, t-Ww	Estes (1949), Hicks and Pierce (2012), Hogue and Kilambi (1975), Mecozzi (2008), Parr (2013), Swingle and Smith (1943), Tanner and Moffett (1995)
Lepomis marginatus	am, Lp, Li, Wwb, Wwp, Wwi, Ri, t-L	
Lepomis megalotis	am, Lp, Li, Wwb, Wwp, Wwi, Ri, t-L	
Lepomis microlophus	am, Lp, Li, Wwb, Wwp, Wwi, Ri, t-L	
Lepomis miniatus	am, Lp, Li, Wwb, Wwp, Wwi, Ri, t-L	
Lepomis symmetricus	am, Lp, Li, Wwb, Wwp, Wwi, Ri, t-L	
Siniperca chuatsi	ab, am, Lb, Lp, Li, Wwb, Wwp, Wwi, Ri, Ww-JX_T, Ww-r_T	Liu et al. (1998)
Amniataba caudavittata	am, Lp, Li, Wwb, Wwp, Wwi, t-L, L-N	Potter et al. (1994), Wise et al. (1994)
Epinephelus marginatus	ab, ap, am, Lb, Lp, Li, Wwb, Wwp, Wwi, R_L, t-L	Cunha et al. (2009), Glamuzina et al. (1998), G"othel (1992), Heemstra and Randall (1993), Reñones et al. (2010), Seyboth et al. (2011)
Epinephelus morio	am, Lp, Li, Wwb, Wwi, R_L, t-L	Jones (2000)
Epinephelides armatus	am, Lp, Li, Wwb, Wwi, GSI, t-L	Moore et al. (2007)
Hyporthodus octofasciatus	am, Lp, Li, Wwb, Wwi, GSI, t-L	Wakefield et al. (2013, 2015)
Serranus scriba	ap, am, Lb, Lp, Li, Wd0, Wwb, Wwp, t-L, L-Ww, L-N	Alonso-Fernández et al. (2011), Alós et al. (2013, 2010), Alvarez-Ellacuria (2017)
Serranus cabrilla	am, Lp, Li, Wwb, Wwp, Wwi, Ri, t-L	Tserpes and Tsimenides (2001)
Mycteroperca microlepis	am, Lp, Li, Wwb, Wwi, Ri, t-L	Strelcheck et al. (2003)
Percina caprodes	am, Lp, Li, Wwb, Wwp, Wwi, Ri, t-L	
Percina macrolepida	am, Lp, Li, Wwb, Wwp, Wwi, Ri, t-L	
Percina maculata	am, Lp, Li, Wwb, Wwi, Ri, t-L	Karr (1963)
Percina nigrofasciata	am, Lp, Li, Wwb, Wwi, t-L, L-N, Ww-N	Karr (1963), Mathur (1973)
Percina notogramma	am, Lp, Li, Wwb, Wwp, Wwi, Ri, t-L	Link (1970)
Percina peltata	am, Lp, Li, Wwb, Wwp, Wwi, Ri, t-L	Link (1970)
Percina phoxocephala	am, Lp, Li, Wwb, Wwi, Ri, t-L	Karr (1963)
Percina sciera	am, Lp, Li, Wwb, Wwp, Wwi, Ri, t-L	Page and Smith (1970)

Continuation of Table B.1		
**species**	**data**	**references**
Percina shumardi	am, Lp, Li, Wwb, Wwp, Wwi, Ri, t-L	
Percina tanasi	ab, ap, am, Lb, Lp, Li, Wwb, Ri, L-Ww, t-L	Starnes (1977)
Ammocrypta beanii	am, Lp, Li, Wwb, Wwi, t-L, L-N	Hems and Rooks (1984)
Ammocrypta clara	am, Lp, Li, Wwb, Wwp, Wwi, Ri, t-L	Drake et al. (2008)
Ammocrypta pellucida	ab, am, Lp, Li, Wwb, Wwi, Ri, t-L	Drake et al. (2008), Wallus et al. (1990)
Crystallaria asprella	am, Lp, Li, Wwb, Wwi, Ri, t-L	George et al. (1996)
Nothonotus bellus	ab, ap, am, Lb, Lp, Li, Wwb, Wwi, Ri, t-L	Fisher (1990)
Nothonotus juliae	am, Lp, Li, Wwb, Wwi, Ri, t-L	James and Taber (1986)
Nothonotus maculatus	am, Lb, Lp, Li, Wwb, Wwi, Ri, dLb	Page and Burr (1991), Ruble et al. (2016)
Nothonotus rubrus	am, Lp, Li, Wwb, Wwi, GSI, t-L	Irwin and Foltz (2012), Knight and Ross (1992)
Nothonotus vulneratus	am, Lb, Lp, Li, Wwb, Wwi, Ri, dLb	Page and Burr (1991), Ruble et al. (2016)
Nothonotus wapiti	am, Lb, Lp, Li, Wwb, Wwi, Ri, dLb	Page and Burr (1991), Ruble et al. (2016)
Etheostoma asprigene	ap, am, Lp, Li, Wwb, Wwi, t-L, L-N	Cummings et al. (1984)
Etheostoma bellator	ap, am, Lp, Li, Wwb, Wwi, Ri, t-L, L-N	Khudamrongsawat and Kuhajda (2007), Page and Burr (1991)
Etheostoma blennioides	ap, am, Lp, Li, Wwb, Wwi, Ri, t-L	Fahy (1954)
Etheostoma barbouri	ap, am, Lp, Li, Wwb, Ri, t-L, t-Ww, L-Ww	Flynn (1975)
Etheostoma caeruleum	ap, am, Lp, Li, Wwb, Wwi, Ri, t-L	Grady and Bart (1984)
Etheostoma chermocki	am, Lp, Li, Wwb, Ri, t-L, L-Ww	Khudamrongsawat et al. (2005)
Etheostoma coosae	am, Lp, Li, Wwb, Wwi, Ri, t-L	O'Neil and Drawer (1981)
Etheostoma cragini	am, Lp, Li, Wwb, Wwi, GSI, t-L	Taber et al. (1986)
Etheostoma flabellare	am, Lp, Li, Wwb, Ri, t-L, t-Ww	Karr (1964)
Etheostoma gracile	ap, am, Lp, Li, Wwb, Wwi, Ri, t-L	Braasch and Smith (1967)
Etheostoma kennicotti	am, Lp, Li, Wwb, Wwi, t-L, L-N	Page (1975)
Etheostoma microperca	ap, am, Lp, Li, Wwb, Wwi, Ri, t-L	Johnsom and Hatch (1991)
Etheostoma nigrum	am, Lp, Li, Wwb, Wwi, Ri, t-L	Karr (1963)
Etheostoma olivaceum	am, Lp, Li, Wwb, Wwi, t-L, L-N	Page (1980)
Etheostoma olmstedi	am, Lp, Li, Wwb, Wwi, t-L, L-N	Tsai (1972)
Etheostoma pallididorsum	am, Lp, Li, Wwb, Wwi, t-L, L-N	Hambrick and Robison (1979)
Etheostoma percnurum	ap, am, Lp, Li, Wwb, Wwi, Ri, t-L	Layman (1991)
Etheostoma perlongum	ab, am, Lp, Li, Wwb, Wwi, Ri, t-L	Shute et al. (1982)
Etheostoma proeliare	am, Lp, Li, Wwb, Wwi, Ri, t-L	Barton and Powers (2010), Burr and Page (1978)
Etheostoma pyrrhogaster	ab, am, Lp, Li, Wwb, Wwp, Wwi, Ri, t-L	Carney and Burr (1989)
Etheostoma radiosum	ap, am, Lp, Li, Wwb, Wwi, Ri, t-L	Scalet (1971)
Etheostoma raneyi	ap, am, Lp, Li, Wwb, Wwi, Ri, t-L	Johnston and Haag (1996)
Etheostoma sagitta	ap, am, Lp, Li, Wwb, Wwi, Ri, t-L, t-Ww	Lowe (1979)
Etheostoma scotti	am, Lp, Li, Wwb, Wwi, Ri, t-L	Barton and Powers (2010)
Etheostoma simoterum	ap, am, Lp, Li, Wwb, Wwi, t-L, L-N, L-Ww	Page and Mayden (1981)

Continuation of Table B.1		
**species**	**data**	**references**
Etheostoma smithi	ab, am, Lp, Li, Wwb, Wwi, t-L, L-N	Page and Burr (1976)
Etheostoma spectabile	am, Lb, Lp, Li, Wwb, Wwi, Ri, t-L_T	West (1966)
Etheostoma squamiceps	ap, am, Lp, Li, Wwb, Wwi, t-L, L-N, L-Ww	Page (1974)
Etheostoma striatulum	am, Lp, Li, Wwb, Wwi, t-L, L-N	Page (1980)
Etheostoma swaini	am, Lp, Li, Wwb, Wwi, t-L, L-N	Ruple et al. (1984)
Etheostoma tetrazonum	ab, am, Lp, Li, Wwb, Wwi, Ri, t-L	Taber and Taber (1983)
Etheostoma trisella	ab, ap, am, Lp, Li, Wwb, Wwi, Ri, t-L	Ryon (1986)
Etheostoma zonistium	ab, am, Lp, Li, Wwb, Wwp, Wwi, Ri, t-L	Carney and Burr (1989)
Sander canadensis	am, Lp, Li, Wwb, Wwp, Wwi, t-L_f, L-N, L-Ww	Carufel (1963), Johnston et al. (2012)
Sander lucioperca	ap, am, Lp, Li, Wwb, Wwp, Wwi, Ri, t-L, L-Ww, T-ab	Aarts (2007)
Sander vitreus	ab, am, Lp, Li, Wwb, Wwp, Wwi, Ri, t-L	Aarts (2007), He et al. (2005)
Sander volgensis	ab, am, Lp, Li, Wwb, Wwp, Wwi, GSI, t-L	Aarts (2007), Kuznetsov (2010), Mishenko et al. (2016)
Zingel asper	am, Lp, Li, Wwb, Wwi, Ri, t-L	Danancher et al. (2007)
Gymnocephalus cernuus	ab, ap, am, Lh, Lb, Lp, Li, Wwb, Wwi, Ri, t-L_f, L-Ww	van Emmerik (2004)
Perca flavescens	am, Lp, Li, Wwb, Wwp, Wwi, Ri, t-L	Ryan and Harvey (1980)
Perca fluviatilis	ab, ap, am, Lb, Lp, Li, Wwi, t-L, L-N	Jellyman (1980), Persson et al. (2004)
Lethrinus lentjan	am, Lp, Li, Wwb, Ri, t-L, L-Ww	Degoon and Ali (2013), Wassef (1991)
Lethrinus mahsena	am, Lp, Li, Wwb, Ri, t-L, L-Ww	Degoon and Ali (2013), Wassef (1991)
Chaenocephalus aceratus	ab, ap, am, Lj, Lp, Li, Wwb, Wwp, Wwi, Ri, t-L	Traczyk (2015)
Eleginops maclovinus	ab, am, Lp, Li, Wwb, Wwp, Wwi, t-L	Brickle et al. (2005a), Licandeo et al. (2006)
Notothenia coriiceps	ab, am, Lp, Li, Wwb, Wwp, Wwi, t-L, L-Ww, L-N	Cali et al. (2017), Linkowski and Zukowski (1980), Postlethwait et al. (2016), White et al. (1996)
Notothenia rossi	ab, am, Lp, Li, Wwb, Wwp, Wwi, t-L	Burchett (1983)
Patagonotothen guntheri	ap, am, Lp, Li, Wwb, Wwp, Wwi, Ri, t-L	Brickle et al. (2005b), Collins et al. (2008)
Pleuragramma antarcticum	ap, am, Li, Wwb, Wwi, t-L	Hubold and Tomo (1989)
Dissostichus eleginoides	ab, ap, am, Lp, Li, Wwb, Wwi, Ri, t-L	Horn (2002)
Dissostichus mawsoni	ab, ap, am, Lp, Li, Wwb, Wwi, Ri, t-L	Horn (2002)
Platycephalus fuscus	am, Lp, Li, Wwb, Wwp, Wwi, GSI, t-L, L-Ww	Gray and Barnes (2008), Hicks et al. (2015)
Platycephalus indicus	ab, am, Lp, Li, Wwb, Wwi, GSI, t-L, t-Ww	Masuda et al. (2000)
Chelidonichthys lucerna	ah, ab, am, Lh, Lb, Lp, Li, Wwi, t-L, L-Ww, L-N	Dulcic et al. (2001), Ismen and Ismen (2004)

Continuation of Table B.1		
**species**	**data**	**references**
Eutrigla gurnardus	ab, ap, am, Lp, Li, Wwb, Wwp, Wwi, Ri, t-L	Magnussen (2007)
Helicolenus dactylopterus	am, Lb, Lp, Li, Wwi, Ri, t-L	Allain and Lorance (2000), Munk and Nielsen (2005)
Helicolenus percoides	am, Lb, Lp, Li, Wwi, Ri, t-L	Paul and Horn (2009)
Sebastes auriculatus	am, Lp, Li, Wwb, Ri, t-L, L-Ww	Love and Johnson (1998)
Sebastes capensis	am, Lp, Li, Wwb, Wwi, Ri, t-L	Andrewan and Hecht (1996)
Sebastes mentella	am, Lp, Li, Wwb, Ri, t-L, t-Ww	Bakay and Mel'nikov (2008), Saborido-Rey et al. (2015)
Sebastes minor	am, Lp, Li, Wwb, Wwi, Ri, t-L, t-Ww	Kolpakov (2006)
Sebastes rastrelliger	am, Lp, Li, Wwb, Ri, t-L, L-Ww	Love and Johnson (1998)
Sebastes rufus	am, Lp, Li, Wwb, Wwi, Ri, t-L	Watters et al. (2006)
Sebastes taczanowskii	am, Lp, Li, Wwb, Wwi, Ri, t-L, t-Ww	Kolpakov (2006)
Scorpaena maderensis	ab, am, Lp, Li, Wwb, Wwi, Ri, t-L	Mesa et al. (2005)
Pontinus clemensi	ap, am, Lp, Li, Wwb, Wwi, GSI, t-L	Jarrin et al. (2018)
Anoplopoma fimbria	ab, am, Lp, Li, Wwb, Wwp, Wwi, Ri, t-L	Alderdice et al. (1988), Clarke et al. (1999), Head et al. (2014)
Anarhichas denticulatus	ab, ap, am, Lb, Lp, Li, Wwi, Ri, t-L, t-Ww	Andriyashev (1954), Munk and Nielsen (2005), Shevelev and Johannesen (2011), Wienerroither et al. (2011)
Anarhichas lupus	ab, ap, am, Lb, Lp, Li, Wwi, R_L, t-L, T-ab	Nelson and Ross (1992), Pavlov and Moksness (1995)
Anarhichas minor	ab, ap, am, Lb, Lp, Li, Wwb, Wwp, Wwi, Ri, t-Ww_T	Imsland et al. (2006), Shevelev and Johannesen (2011)
Zoarces viviparus	ab, ap, am_T, Lb, Lp, Li, Wwb, Wwp, Wwi, Ww_L, rB_T, R_L	Brodte (2001), van der Veer (2009), Vetemaa (1999), Witte (2009)
Zoarces elongatus	am, Lp, Li, Wwb, Wwp, Wwi, Ri, t-L, t-Ww, L-N	Poezzhalova-Chegodaeva (2016)
Lycodes raridens	am, Lp, Li, Wwb, Ri, t-L, t-Ww	Balanov et al. (2006)
Gymnelus viridis	am, Lp, Li, Wwb, Wwi, Ri, t-L	Dunbrack and Green (2017)
Hadropareia middendorffii	am, Lp, Li, Wwb, Wwp, Wwi, Ri, t-L, t-Ww, L-N	Poezzhalova-Chegodaeva (2016)
Stichaeus punctatus	am, Lp, Li, Wwb, Ri, t-L, t-Ww	Dunbrack and Green (2017), Farwell et al. (1976)
Alectrias alectrolophus	am, Lp, Li, Wwb, Ri, t-L_f, t-Ww_f, L-Ww_f	Kolpakov and Milovankin (2014), Kolpakov and Miroshnik (2007)
Pholis fangi	am, Lp, Li, Wwb, Wwi, GSI, t-L	Hwang et al. (2008), Sawyer (1967), Vallis et al. (2007)
Pholis gunnellus	am, Li, Wwb, Wwp, Wwi, t-Ww, Ww-WwR	Sawyer (1967), Vallis et al. (2007)
Gasterosteus aculeatus	ab, ap, am, Lb, Lp, Li, Wwb, Wwp, Wwi, Ri, t-L_f	Jones and Hynes (1950), Paepke (1983), Wootton (1973), Wright et al. (2004)

Continuation of Table B.1		
**species**	**data**	**references**
Pungitius pungitius	ab, ap, am, Lb, Lp, Li, Wwb, Wwp, Wwi, Ri, t-L	Jones and Hynes (1950), Paepke (1983), Wright et al. (2004)
Culaea inconstans	ab, am, Lp, Li, Wwb, Wwp, Wwi, Ri, t-L	Acere and Lindey (1986), Stewart et al. (2007)
Apeltes quadracus	ab, am, Lp, Li, Wwb, Wwp, Wwi, Ri, t-L	Craig and FitzGerald (1982), Schwarz (1965)
Hexagrammos agrammus	am, Lp, Li, Wwb, Wwp, Wwi, Ri, t-L, t-Ww	Kurita et al. (1991)
Hexagrammos octogrammus	am, Lp, Li, Wwb, Wwp, Ri, t-L, t-Ww	Shestakov and Nazarkin (2006)
Hexagrammos stelleri	am, Lp, Li, Wwb, Wwp, Wwi, Ri, t-L, t-Ww	Shestakov and Nazarkin (2006)
Cyclopterus lumpus	ah, ab, am, Lb, Lp, Li, Wwi, Ri, Ww-dWw_T, t-L	Nytrø (2013), Thorsteinsson (1983)
Scorpaenichthys marmoratus	ab, ap, am, Lb, Lp, Li, Wwi, t-L, L-Ww, Ww-N	Grebel and Cailliet (2010), O'Connell (1953)
Myoxocephalus scorpius	ah, ab, am, Lh, Lj, Lp, Li, Wwp, Wwi, ss, L-N, Ww-N	Luksenburg et al. (2004)
Myoxocephalus thompsonii	ap, am, Li, Wwb, Wwi, Ri, t-L	Geffen and Nash (1992)
Batrachocottus baicalensis	am, Lp, Li, Wwb, Ri, t-L, t-Ww	Tolmacheva et al. (2008)
Paracottus knerii	am, Lp, Li, Wwb, Ri, t-L, t-Ww	Tolmacheva and Bogdanov (2010)
Cottus aleuticus	am, Lp, Li, Wwb, Wwp, Wwi, t-L, L-N, L-Ww	Greenbank (1966), Patten (1971)
Cottus asperrimus	am, Lp, Li, Wwb, Wwi, Ri, t-L	Daniels (1987)
Cottus bairdii	am, Lp, Li, Wwb, Wwp, Wwi, t-L, L-N	Bailey (1952), Patten (1971)
Cottus beldingii	am, Lp, Li, Wwb, Wwi, t-L, L-N	Ebert and Summerfelt (1969), Patten (1971)
Cottus carolinae	am, Lp, Li, Wwb, Wwp, Wwi, Ri, t-L	Gebhard and Perkin (2017)
Cottus cognatus	am, Lp, Li, Wwb, Wwp, Wwi, Ri, t-L	Bond et al. (2016)
Cottus confusus	am, Lp, Li, Wwb, Wwp, Wwi, t-L, L-N	Hughes and Peden (1984), Patten (1971)
Cottus extensus	am, Lp, Li, Wwb, Wwp, Wwi, Ri, t-L	Ruzycki et al. (1998)
Cottus gobio	am, Lp, Li, Wwb, Wwi, Ri, t-L, tL_m, tL_f	Peters (2009)
Cottus hangiongensis	am, Lp, Li, Wwb, Wwi, Ri, t-L	Goto (1989)
Cottus klamathensis	am, Lp, Li, Wwb, Wwi, Ri, t-L	Daniels (1987)
Cottus leiopomus	am, Lp, Li, Wwb, Wwp, Wwi, t-L, L-N	Meyer et al. (2008)
Cottus pitensis	am, Lp, Li, Wwb, Wwi, Ri, t-L	Daniels (1987)
Cottus volki	am, Lp, Li, Wwb, t-L, t-Ww, L-Ww, L-N	Kolpakov (2011)
Gymnocanthus herzensteini	am, Lp, Li, Wwb, Ri, t-L, L-Ww	Panchenko (2010)
Gymnocanthus detrisus	am, Lp, Li, Wwb, Ri, t-L, L-Ww	Panchenko (2010, 2012)
Gymnocanthus pistilliger	am, Lp, Li, Wwb, Ri, t-L, L-Ww	Panchenko (2012)

Continuation of Table B.1		
**species**	**data**	**references**
Gymnocanthus intermedius	am, Lp, Li, Wwb, Ri, t-L, L-Ww	Panchenko (2012)
Taurocottus bergii	am, Lp, Li, Wwb, Ri, t-L, L-Ww	Panchenko et al. (2015)
Leptocottus armatus	am, Lp, Li, Wwb, Wwp, Wwi, Ri, t-L	Weiss (1962)

Table B.2: The codes of the data types as presented in Table B.1. Zero variate data left, uni-variate data right. Life history events: b birth, s settlement, j end of acceleration, p puberty, m death, i death. T stands for temperature.

**code**	**description**	**code**	**description**
ah	age at h	t-Le	time, embryo length
ah_T	age at h (several T))	t-Le_T	time, embryo length (several T)
ab	age at birth	t-L	time, length
ab_T	age at birth (several T)	t-L_T	time, length (several T)
ab_f	age at birth (several f)	t-L_f	time, length (several f)
as	age at s	t-L_fT	time, length (several f, T)
aj	age at j	t-dL	time, change in length
ap	age (or time since birth) at p	t-VY	time, yolk volume
am	age at death (life span)	t-Wwe	time, embryo wet weight
am_T	age at death (several T)	t-Ww	time, wet weight
L0	length at 0	t-Ww_f	time, wet weight (several f)
Lh	length at h	t-Ww_T	time, wet weight (several T)
Lb	length at b	t-Ww_fT	time, wet weight (several f, T)
Ls	length at s	t-WwR	time, gonad wet weight
Lj	length at j	t-WdYe	time, embryo dry weight (yolk)
Lp	length at p	t-Wde	time, embryo dry weight (total)
Li	length at i	t-Wd_T	time, dry weight (several T)
L_t	length at time t	t-E	time, energy content
L_W	length at time weight	t-p+	time, heat production
dLb	change in length at b	t-N	time, number of offspring
dLp	change in length at p	t-N_f	time, number of offspring (several f)
dLj	change in length at j	t-JX_T	time, ingestion rate (several T)
Ww0	wet weight at 0	t-JCe	time, embryo CO_2 production
Wwh	wet weight at h	t-JOe	time, embryo O_2 consumption
Wwb	wet weight at b	t-JNe	time, embryo NH_3 production
Wwj	wet weight at j	t-S	time, surviving fraction
WwR	wet weight at R	t-S_f	time, surviving fraction (several f)
Wwp	wet weight at p	t-dC	time, condition factor
Wwi	wet weight at i	X-JX	food density, ingestion rate
Ww_L	wet weight at length	L-L	length, length (different length measures)
Ww_t	wet weight at time	L-dL	length, change in length
Wd0	dry weight at 0	L0-Lt	length at capture, length at recapture
Wdh	dry weight at h	L-Ww	length, wet weight
Wdb	dry weight at b	L-Ww_f	length, wet weight (several f)

Table B.3: Continuation of Table B.3.

code	description	code	description
Wdb_f	dry weight at b (several f)	L-Wde_T	length, embryo dry weight (several T)
Wdj	dry weight at j	L-Wd	length, dry weight
E0	reserve energy at 0	L-N	length, number of eggs/offspring
rB	von Bertalanffy growth rate	L-N_f	length, number of eggs/offspring (several f)
rB_T	von Bertalanffy growth rate (several T)	L-X	length, cumulated food ingested
Ri	reproduction rate at i	L-JO_T	length, O_2 consumption (several T)
R_L	reproduction rate at length	Ww0-Wwt	wet weight at capture, wet weight at recapture
R_W	reproduction rate at weight	Ww-dWw_T	wet weight, change in wet weight (several T)
GSI	gonado-somatic index	Ww-N	wet weight, number of eggs/offspring
Ni	(total) number of offspring at i	Ww-WwR	wet weight, clutch wet weight
JO_W	O2 consumption at W	Ww-R	wet weight, reproduction rate
ss	supply stress	Ww-JX_T	wet weight, ingestion rate (several T)
		Ww-JO	wet weight, O_2 consumption
		Ww-JO_T	wet weight, O_2 consumption (several T)
		T-ah	temperature, age at h
		T-ab	temperature, age at b
		T-aj	temperature, age at j
		T-dL	temperature, change in length
		T-dWw_f	temperature, change in wet weight (several f)
		T-JX	temperature, food consumption
		T-JO	temperature, O_2 consumption
		T-JO_f	temperature, O_2 consumption (several f)
		T-JN_W	temperature, NH_3 production (several W)

References

A.Al-Marzouqi, Jayabalan, N., Al-Nahdi, A., and Al-Anbory, I. (2011). Reproductive biology of the white-spotted rabbitfish, Siganus canaliculatus (Park, 1797) in the Arabian Sea coast of Oman. Western Indian Ocean J. Mar. Sci., 10:73–82.

Aarts, T. W. P. M. (2007). Kennisdocument snoekbaars, Sander lucioperca (Linnaeus, 1758). Technical report, Sportvisserij Nederland.

Abaunza, P., Gordo, L., Karlou-Riga, C., Murta, A., Eltink, A. T. G. W., Santamaria, M. T. G., Zimmermann, C., Hammer, C., Lucio, P., Iversen, S. A., Molloy, J., and Gallo, E. (2003). Growth and reproduction of horse mackerel, Trachurus trachurus (Carangidae). Reviews in fish biology and fisheries, 13:27–61.

Abbasi, F. and Ghorbani, R. (2010). Study on population dynamics of Spirlin (Alburnoides eichwaldii) in TilAbad, Kaboudval and ShirAbbad streams, Golestan Province. PhD thesis, Gorgan university of Agricultural Sciences and Natural Resources, Iran.

Abdussamad, E. M., Pillai, N. G. K., Zacharia, P. U., and Jeyabalan, K. (2011). Dorab fishery of Gulf of Mannar waters and population characteristics of the species Chirocentrus dorab (forskål, 1775) and Chirocentrus nudus Swainson, 1839. Indian J. Fish., 58:19–23.

Acere, T. O. and Lindey, C. C. (1986). Age, growth and life history of Culaea inconstans (Pisces: Gasterostidae) in Delta March Lake Manitoba. Hydrobiologia, 135:35–44.

Acosta, A. (2000). Estimation of growth and mortality of bay anchovy, Anchoa mitchilli, in Florida bay, Florida U.S.A. In Creswell, R. L., editor, Proc. 51st Gulf and Caribbean Fisheries Institute, St. Croix, US Virgin Islands, November 1998. Fort Pierce, Florida.

Aguado-Gimenez, F. and Garcia-Garcia, B. (2005). Growth, food intake and feed conversion rates in captive Atlantic bluefin tuna (Thunnus thynnus Linnaeus, 1758) under fattening conditions. Aquacult. Res., 36:610–614.

Agüera, A. and Brophy, D. (2012). Growth and age of Atlantic saury, Scomberesox saurus saurus (Walbaum), in the northeastern Atlantic Ocean. Fisheries Research, 131-133:60–66.

Ahlstrom, E. H., Amaoka, K., Hensley, D. A., Moser, H. G., and Sumida, B. Y. (1984). Pleuronectiformes: development. Ontogeny and systematics of fishes, 1.

Al-Husaini, M., Al-Baz, A., Al-Ayoub, S., Safar, S., Al-Wazan, Z., and Al-Jazzaf, S. (2002). Age, growth, mortality, and yield-per-recruit for nagroor, Pomadasys kakaan, in Kuwait's waters. Fisheries Research, 59:101–115.

Alavi, M. H., Karami, M., Abdoulhay, H., and Ghadirnejad, S. H. (2005). Length, weight and age relationships of the Persian sturgeon Acipenser persicus in the southeast of og the Caspian sea: A case study at Turkaman station during summer catching. Iranian Journal of Natural Resources, 3:603–614.

Alderdice, D. F. and Forrester, C. R. (1971). Effects of salinity and temperature on embryonic development of the petrale sole (eopsetta iordani). J. Fish. Res. Bd. Canada, 28:727–744.

Alderdice, D. F., Jensen, J. O. T., and Velsen, F. P. J. (1988). Preliminary trials on incubation of sablefish eggs (Anoplopoma fimbria). Aquaculture, 69:271–290.

Alessandro, R. (2014). Field measurement from Marettimo Island.

Allain, V. and Lorance, P. (2000). Age estimation and growth of some deep-sea fish from the northeast Atlantic Ocean. Cybium, 24(3, suppl):7–16.

Allen, M. A. and Hassler., T. (1985). Chinook salmon. species profiles: Life histories and environmental requirements of coastal fishes and invertebrates (Pacific Southwest). Technical report, U.S. Fish and Wildlife Service.

Allman, R. J. (2007). Small-scale spatial variation in the population structure of vermilion snapper (Rhomboplites aurorubens) from the northeast Gulf of Mexico. Fisheries Research, 88:88–99.

Almukhtar, M. A., Jasim, W., and Mutlak, F. (2016). Reproductive biology of hilsa shad Tenualosa ilisha (Teleostei: Clupeidae) during spawning migration in the Shatt Al Arab River and southern Al Hammar Marsh, Basra, Iraq. Journal of Fisheries and Aquatic Science, 11:43–55.

Alonso-Fernádez, A. and Palmer, J. A. M. (2014). Variability in reproductive traits in the sex-changing fish, Coris julis, in the Mediterranean. Mediterranean Marine Science, 15:106–114.

Alonso-Fernández, A., Alós, J., Grau, A., Domínguez-Petit, R., and Saborido-Rey, F. (2011). The use of histological techniques to study the reproductive biology of the hermaphroditic mediterranean fishes Coris julis, Serranus scriba and Diplodus annularis. Mar Coast Fish., 3:145–159.

Alonso-fernandez, A., Vergara, A. R., and Saborido-Rey, F. (2010). Embryonic development and spawning pattern of Trisopterus luscus (Teleostei: Gadidae) under controlled conditions. Journal of the Marine Biological Association of the United Kingdom, ?:?

Alonzo, S. H., Key, M., Ish, T., and MacCall, A. D. (2004). Status of the California sheephead (Semicossyphus pulcher) stock. Technical report, Center for Stock Assessment Research (CSTAR) and the Institute of Marine Sciences, University of California Santa Cruz.

Alós, J., Alonso-Fernández, A., Catalán, I. A., Palmer, M., and Lowerre-Barbieri, S. (2013). Reproductive output traits of the simultaneous hermaphrodite Serranus scriba in the western Mediterranean. Scientia Marina, 77(2):331–340.

Alós, J., Palmer, M., Balle, S., Grau, A. M., and Morales-Nin, B. (2010). Individual growth pattern and variability in Serranus scriba: a Bayesian analysis. ICES Journal of Marine Science, 67(3):502–512.

Alp, A., Kara, C., Uckardes, F., Carol, J., and Garcia-Berthou, E. (2011). Age and growth of the European catfish (Silurus glanis) in a Turkish reservoir and comparison with introduced populations. Rev Fish Biol Fisheries, 21:283–294.

Alt, K. T. (1973). Contributions to the biology of the Bering cisco (Coregonus laurettae) in Alaska. J. Fish. Res. Board Canada, 30(12):1885–1888.

Alvarez-Ellacuria, I. (2017). Personal lab observation, 2017.

Amezcuaa, F., Soto-Avila, C., and Green-Ruiz, Y. (2006). Age, growth, and mortality of the spotted rose snapper Lutjanus guttatus from the southeastern Gulf of California. Fisheries Research, 77:293–300.

Amin, S. M. N., Arshad, A., Haldar, G. C., Shohaimi, S., and Ara, R. (2005). Estimation of size frequency distribution, sex ratio and length-weight relationship of hilsa (Tenualosa ilisha) in the Bangladesh water. Research Journal of Agriculture and Biological Sciences, 1:61–66.

Anastasopoulou, A. K. and Papaconstantinou, C. (2006). Age and growth of Chlorophthalmus agassizi (B. 1840) in ten Ionian Sea. In 8th Hel. Symp. Oceanogr. & Fish. in Thessaloniki, greeceVolume: Book of Abstracts, page 212.

Anderson, R. O. and Neumann, R. M. (1996). Length, weight, and associated structural indices, in fisheries techniques. In Murphy, B. E. and Willis, D. W., editors, Fisheries Techniques. American Fisheries Society.

Andrewan, T. G. and Hecht, D. T. (1996). Age and growth of the soldier, Sebastes capensis (Pisces: Scorpaenidae) at Tristan da Cunha and Gough Island, South Atlantic Ocean. J. Zool., Lond., 238:125–135.

Andrews, A. H. (1997). Age and growth of the Pacific grenadier (Family Macrouridae, Coryphaenoides acrolepis) with age estimate validation using improved radiometric ageing technique. PhD thesis, California State Universities, Faculty of Moss Landing Marine Laboratories.

Andrews, J. W. and Matsuda, Y. (1975). The influence of various culture conditions on the oxygen consumption of channel catfish. Transactions of the American Fisheries Society, 104:322–327.

Andrews, J. W. and Stickney, R. R. (1972). Interactions of feeding rates and environmental temperature on growth, food conversion, and body composition of channel catfish. Transactions of the American Fisheries Society, 101:94–99.

Andriyashev, A. P. (1954). Fishes of the Northern Seas of the U. S. S. R. Izdatelstvo Akademii Nauk SSSR. translated from russian.

Andriyashev, A. P. (1964). Fishes of the northern seas of the U.S.S.R. Smithsonian Institution of the National Science Foundation, Washington D.C.

Angelidis, P., Pournara, I., and Photis, G. (2005). Glass eels (Anguilla anguilla) growth in a recirculating system. Mediterranean Marine Science, 4:99–106.

Ankley, G. T. and Johnson, R. D. (2004). Small fish models for identifying and assessing the effects of endocrine-disrupting chemicals. ILAR Journal, 45(4):469–483.

Anonimous (2008). Scientific report of efsa prepared by working group on seabass/seabream welfare on animal welfare aspects of husbandry systems for farmed European seabass and gilthead seabream.

Anonymous (1998). Oregon chub (Oregonichthys Crameri): Recovery plan. Technical report, US Fish and Wildlife Service, Oregon State Offfice, Portland, Oregon.

Anonymous (2001). Guide to federally listed endangered and threatened species of north carolina. Technical report, North Carolina Natural Heritage Program, Division of Parks and Recreation, North Carolina Department of Environment and Natural Resources.

Aragón-Noriega, E. A. (2014). Modeling the individual growth of the gulf corvina, Cynoscion othonopterus (Pisces: Sciaenidae), using a multi-model approach. Ciencias Marinas, 40:149–161.

Arantes, C. C., Castello, L., Stewart, D. J., Cetra, M., and Queiroz, H. L. (2010). Population density, growth and reproduction of arapaima in an Amazonian river-floodplain. Ecology of Freshwater Fish.

Archdeacon, T. P. and Bonar, S. A. (2009). Captive breeding of endangered Mohave tui chub. North American Journal of Aquaculture, 71:360–362.

Arellano-Martínez, M., Ceballos-Vazquez, B. P., and Galvan-Magana, F. (2007). Reproductive cycle of the cortez angelfish pomacanthus zonipectus (gill, 1863) (pomacanthidae) from the gulf of california, mexico. Hidrobiologica, 17:241–248.

Arenzon, A., Peret, A. C., and Bohrer, M. B. C. (1999). Reproduction of the annual fish Cynopoecilus melanotaenia (Regan, 1912) in a temporary water body in Rio Grande do Sul, Brazil (Cyprinodontiformes, Rivulidae). Hydrobiologia, 411:65–70.

Arenzon, A., Peret, A. C., and Bohrer, M. B. C. (2001). Growth of the annual fish Cynopoecilus melanotaenia (Regan, 1912) based in a temporary water body population in Rio Grande do Sul State, Brazil (Cyprinodontiformes, Rivulidae). Rev. Brasil. Biol., 61(1):117–123.

Arimoro, F. O. and Ofojekwu, P. C. (2003/2004). Incidence of feeding, growth and survival of the toothed carp, Aphyosemion gardneri larvae reared on the freshwater rotifer, Brachionus calyciflorus. Tropical Freshwater biology, 12/13:35–43.

Aschenbrenner, A., Freitas, M. O., Rocha, G. R. A., de Moura, R. L., Francini-Filho, R. B., Minte-Vera, C., and Ferreira, B. P. (2017). Age, growth parameters and fisheries indices for the lane snapper in the Abrolhos Bank, SW Atlantic. Fisheries Research, 194:155–163.

Assuncao, M. I. d. S. and Schwassmann, H. O. (1995). Reproduction and larval development of Electrophorus electricus on Marajo Island (Para, Brazil). Ichthyol. Explor. Freshwaters, 6:175–184.

Atsushi Nanamia, T. T. (2005). Age and growth of the mudskipper Boleophthalmus pectinirostris in Ariake Bay, Kyushu, Japan. Fisheries Research, 74:24–34.

Auer, S. K., Dick, C. A., Metcalfe, N. B., and Reznick, D. N. (2018). Metabolic rate evolves rapidly and in parallel with the pace of life history. Nature Communications, 9(1):1–6.

Augustine, S. (2009). unpublished 2009, f = 1.

Azevedo, J. M. N. and Homem, N. (2002). Age and growth, reproduction and diet of the red blenny Parablennius ruber (Blenniidae). Cybium, 26:129–133.

Azevedo, J. M. N. and Simas, A. M. V. (2000). Age and growth, reproduction and diet of a sublittoral population of the rock goby Gobius paganellus (Teleostei, Gobiidae). Hydrobiologia, 440:129–135.

Babatunde, S. L. (2011). Morphometric and meristics characteristics of Erpetoichthys calabaricus from wetland of Ogun water-side local government area. Technical report, Universtity of Agriculture, Abeokuta, Ogun State, Nigeria.

Bacheler, N. M. (2002). Ecology of bigmouth sleepers (Eleotridae: Gobiomorus dormitor) in a Puerto Rico reservoir. PhD thesis, North Carolina State Univ.

Bacheler, N. M., Neal, J. W., and Noble, R. L. (2004). Reproduction of a landlocked diadromous fish population: Bigmouth sleepers Gobiomorus dormitor in a reservoir in Puerto rico. Caribbean Journal of Science, 40(2):223–231.

Bachman, B. A., Kraus, R., Peterson, C. T., Grubbs, R. D., and Peters, E. C. (2018). Growth and reproduction of Echeneis naucrates from the eastern Gulf of Mexico. J Fish Biol., 93:755–758.

Badsha, K. and Sainsbury, M. (1978). Aspects of the biology and heavy metal accumulation of Ciliata mustela.

Bagarinao, T. U. (1991). Biology of milkfish (Chanos chanos Forsskål).

Bagatto, B., Pelster, B., and Burggren, W. W. (2001). Growth and metabolism of larval zebrafish: effects of swim training. Journal of Experimental Biology, 204:4335–4343.

Bagenal, T. B. (1963). The fecundity of witches in the Firth of Clyde. Journal of Mar. Biol. Assoc. UK, 43.

Bailey, J. E. (1952). Life history and ecology of the sculpin Cottus bairdi punctulatus in Southwestern Montana. Copeia, 1952(4):243–255.

Bailey, M. M. (1969). Age, growth, and maturity of the longnose sucker Catostomus catostomus of western Lake Superior. J. Fish, Res. Brd Canada, 26:1289–1299.

Bakay, Y. I. and Mel'nikov, S. P. (2008). Biological and ecological characteristics of deepwater redfish Sebastes mentella (Scorpaenidae) at different depths in the pelagial of the Irminger Sea. Journal of Ichthyology, 48:68–80.

Baker, J. A. and Heins, D. C. (1994). Reproductive life history of the North American madtom catfish, Noturus hildebrandi (Bailey & Taylor 1950), with a review of data for the genus. Ecology of Freshwater Fish, 3:167–175.

Baker, L. L., Wiff, R., Quiroz, J. C., Flores, A., Cespedes, R., Barrientos, M. A., Ojeda, V., and Gatica, C. (2014). Reproductive ecology of the female pink cusk-eel (Genypterus blacodes): evaluating differences between fishery management zones in the Chilean austral zone. Environ. Biol. Fish, 97:1083–1093.

Balanov, A. A., Badaev, O. Z., Napazakov, V. V., and Chuchukalo, V. I. (2006). Distribution and some biological features of Lycodes raridens (Zoarcidae) in the western part of the Bering Sea. Journal of Ichthyology, 46:148–155.

Ballagh, A. C., Welch, D. J., Newman, S. J., Allsop, Q., and Stapley, J. M. (2012). Stock structure of the blue threadfin (Eleutheronema tetradactylum) across northern Australia derived from life-history characteristics. Fisheries Research, 121-122:63–72.

Bang, A., Grønjær, P., and Malte, H. (2004). Individual variation in the rate of oxygen consumption by zebrafish embryos. Journal of Fish Biology, 64:1285–1296.

Baras, E. and Lucas, M. C. (2005). Individual growth trajectories of sibling Brycon moorei raised in isolation since egg stage, and their relationship with aggressive behaviour. Journal of Fish Biology, 66:996–1024.

Baras, E. and Lucas, M. C. (2010). Individual growth trajectories of sibling Brycon moorei raised in isolation since egg stage, and their relationship with aggressive behaviour. Journal of Fish Biology, 77:985–997.

Barber, W. E., Williams, D. C., and Minckley, W. L. (1970). Biology of the gila spikedace, Meda fulgida, in Arizona. Copeia, 1970(1):9–18.

Barbieri, L. R., Chittenden, M. E., and Lowerre-Barbieri, S. K. (1994). Maturity, spawning, and ovarian cycle of Atlantic croaker, Micropogonias-Undulatus, in the Chesapeake Bay and adjacent coastal waters. VIMS Articles, 594:13–99.

Barney, R. L. and Anson, B. J. (1923). Life history and ecology of the orange-spotted sunfish Lepomis humilis. Technical report, Dept of Commerce, Washington, Bureau of fisheries.

Barriga, J. P., Battini, M. A., Garcia-Asorey, M., Carrea, C., Macchi, P. J., and Cussac, V. E. (2012). Intraspecific variation in diet, growth, and morphology of landlocked Galaxias maculatus during its larval period: the role of food availability and predation risk. Hydrobiologia (2012) 679:, 679.

Barton, S. D. and Powers, S. L. (2010). Life-history aspects of the Cherokee darter, Etheostoma scotti (Actinopterygii: Percidae), an imperiled species in Northern Georgia. Southeastern Naturalist, 9(4):687–698.

Bartsch, P., Gembells, S., and Piotrowski, T. (1997). The embryonic and larval development of Polypterus senegalus Cuvier, 1829: its staging with reference to external and skeletal features, behaviour and locomotory habits. Acta Zoologica (Stockholm), 78:309–328.

Basto-Rosales, M. E. R., de Oca, G. A. R.-M., Carrillo-Farnés, O., Alvarez-González, C. A., Badillo-Zapata, D., and Vega-Villasante, F. (2019). Growth of Dormitator latifrons under different densities in concrete tanks. Tropical and Subtropical Agroecosystems, 22:499–503.

Battes, K. W. and Stoica, I. (2005). Bitterling growth biology (Rhodeus amarus L.) in the Bistria River. Analele Univ. Oradea, Fasc. Biologie, 12:21–29.

Bayer, A. (2005). Control data from OECD tests, report number M-247419-01-1 F0 generation rep. 1 day 0.

Beacham, T. D. (1982). Fecundity of coho salmon (Oncorhynchus kisutch) and chum salmon (O. keta) in the northeast Pacific Ocean. Canadian Journal of Zoology, 60:1463–1469.

Beacham, T. D. and Murray, C. B. (1990). Temperature, egg size, and development of embryos and alevins of 5 species of Pacific salmon - a comparative analysis. Transactions of the American Fisheries Society, 119:927–945.

Beacham, T. D. and Murray., C. B. (1993). Fecundity and egg size variation in North-American Pacific salmon (Oncorhynchus). Journal of Fish Biology, 42:485–508.

Beacham, T. D., Withler, C., and Morly, R. B. (1985). Effect of egg size on incubation time and alevin and fry size in chum salmon (Oncorhynchus keta) and coho salmon (Oncorhynchus kisutch). Canadian Journal of Zoology, 63:847–850.

Beadouin, R., Ginot, V., and Monod, G. (2008). Growth characteristics of eastern mosquitofish Gambusia holbrooki in a northern habitat (Brittany, France). Journal of Fish Biology, 73:2468–2484.

Beamesderfer, R. C. (1992). Reproduction and early life history of northern squawfish, Ptychocheilus oregonensis, in Idaho's St. Joe River. Environmental Biology of Fishes, 35(3):231–241.

Beamish, R. J. (1973). Determination of age and growth of populations of the white sucker (Catostomus commersoni) exhibiting a wide range in size at maturity. Journal of the Fisheries Research Board of Canada, 30:607–616.

Beaudouin, R., Goussen, B., Piccini, B., Augustine, S., Devillers, J., Brion, F., and Pèry, A. R. R. (2015). An individual-based model of zebrafish population dynamics accounting for energy dynamics. PloS one, 10(5).

Beckman, D. W. and Hutson, C. A. (2012). Validation of aging techniques and growth of the river redhorse, Moxostoma carinatum, in the James River, Missouri. The Southwestern Naturalist, 57(3):240–247.

Beckman, D. W., Stanley, A. L., Render, J. H., and Wilson, C. A. (1991). Age and growth-rate estimation of sheepshead Archosargus probatocephalus in Louisiana waters using otoliths. Fishery Bulletin, U.S., 89:1–8.

Beekman, J. (2007). Kennisdocument sneep, Chondrostoma nasus (Linnaeus, 1758). Technical report, Sportvisserij Nederland.

Beekman, J. and van Emmerik, W. A. M. (2005). gestippelde alver Alburnoides bipunctatus (Bloch, 1782). Technical report, Sportvisserij Nederland.

Beekman, J. and van Emmerik, W. A. M. (2007). Kennisdocument blankvoorn Rutilus rutilus (Linnaeus, 1758). Technical report, Sportvisserij Nederland.

Beelen, P. (2008). Kennisdocument zeelt, Tinca tinca (Linnaeus, 1758). Technical report, Sportvisserij Nederland.

Beers, M. C. (2005). Kennisdocument riviergrondel, Gobio gobio (Linnaeus, 1758). Technical report, Sportvisserij Nederland.

Belk, M. C. (1998). Age and growth of june sucker (Chasmistes liorus) from otoliths.

Bengsten, D. A., , Barkman, R. C., and Berry, W. J. (1987). Relationships between maternal size, egg diameter, time of spawning season, temperature, and length at hatch of Atlantic silverside, Menidia menidia. J. Fish Biol., 31:697–704.

Bengtsson, B.-E. (1974). The effects of zinc on the mortality and reproduction of the minnow Phoxinus phoxinus L. Archives of Environmental Contamination and Toxicology, 2(4):342–355.

Benjamin, J. R., Wetzel, L. A., Martens, K. D., Larsen, K., and Connolly, P. J. (2014). Spatio-temporal variability in movement, age, and growth of mountain whitefish (Prosopium williamsoni) in a river network based upon PIT tagging and otolith chemistry. Can. J. Fish. Aquat. Sci., 71:131–140.

Bennett, M. G. and Kuhajda, B. R. (2008). Life history aspects of the black madtom, Noturus funebris (Siluriformes: Ictaluridae), in the Cahaba River Drainage, Central Alabama, USA. Journal of Freshwater Ecology, 23(3):365–372.

Bennett, M. G., Kuhajda, B. R., and Khudamrongsawat, J. (2010). Life-history attributes of the imperiled frecklebelly madtom, Noturus munitus (Siluriformes: Ictaluridae), in the Cahaba River System, Alabama. Southeastern Naturalist, 9(3):507–520.

Benseman, S. A. and Allen, L. G. (2018). Distribution and recruitment of young-of-the-year giant sea bass, Stereolepis gigas, off southern California. Copeia, 106:312–320.

Berg, O. K., Hendry, A. P., B. Svendsen, C. B., Arnekleiv, J. V., and Lohrmann, A. (2001a). Maternal provisioning of offspring and the use of those resources during ontogeny: variation within and between Atlantic salmon families. Functional Ecology, 15:13–23.

Berg, O. K., Hendry, A. P., Svendsen, B., Bech, C., Arnekleiv, J. V., and Lohrmann, A. (2001b). Maternal provisioning of offspring and the use of those resources during ontogeny: variation within and between Atlantic salmon families. Functional Ecology, 15:13–23, Table 1.

Bergstad, O. A., øA. S. Høines, and Jørgensen, T. (2002). Growth of sandeel, Ammodytes marinus, in the northern North Sea and Norwegian coastal waters. Fisheries Research, 56:9–23.

Berois, N., Garcia, G., and de Sa, R. O. (2016). Anual fishes: life history strategy, diverty and evolution. CRC Press, Boca Raton.

BERRA, T. M. and ADAY, D. D. (2004). Otolith description and age-and-growth of Kurtus gulliveri from Northern Australia. Journal of Fish Biology, 65:354–362.

BERRA, T. M. and Neira, F. J. (2003). Early life history of the nerseryfish, Kurtus gulliveri (Percoformes: Kurtidae), from Northern Australia. Copeia, 2003:384–390.

Best, J., Adatto, I., Cockington, J., James, A., and Lawrence, C. (2010). A novel method for rearing first feeding larval zebrafish: polyculture with Type L saltwater rotifers (Brachionus plicatilis). Zebrafish, 352 7(3):89–295.

Bestgen, K. R. and Compton, R. I. (2007). Reproduction and culture of suckermouth minnow. North American Journal of Aquaculture, 69:345–350.

Betti, P., Brown, D. R., Temperoni, B., Machinandiarena, L., and Ehrlich, M. D. (2014). Larval growth of hake (Merluccius hubbsi) in the Patagonian shelf: Analysis of two reproductive seasons. Fisheries Research, 160.

Beyger, L. A. (2009). Growth, Survivability, and Reproductive Effects of Pulse-Dosed Endosulfan on Jordanella floridae (Florida flagfish) Over One Complete Life-cycle. PhD thesis, Univ. Otario, Institute of Technology.

Bhujel, R. C., Little, D. C., and Hossain, A. (2007). Reproductive performance and the growth of pre-stunted and normal Nile tilapia (Oreochromis niloticus) broodfish at varying feeding rates. Aquaculture, 273:71–79.

Bigelow, H. B., Bradbury, M. G., Dymond, J. R., Greeley, J. R., Hildebrand, S. F., Mead, G. W., Miller, R. R., Rivas, L. R., Schroeder, W. L., Suttkus, R. D., and Vladykov, V. D. (1963). Fishes of the western North Atlantic. Part three. Mar. Res., Yale Univ., New Haven, Sears Found.

Bilecenoglu, M. and Kaya, M. (2002). Growth of the marbled spinefoot Siganus rivulatus Forsskøal, 1775 (Teleostei: Siganidae) introduced to antalya Bay, eastern Mediterranean Sea (Turkey). Fisheries Research, 54:279–285.

Billman, E. J., Tjarks, B. J., and Belk, M. C. (2011). Effect of predation and habitat quality on growth and reproduction of a stream fish. Ecology of Freshwater Fish, 20:102–113.

Bird, F. H. (1975). Biology of the blue and tui chubs in East and Paulina Lakes, Oregon. PhD thesis, Oregon State University.

Birstein, V. J., Waldman, J. R., and Bemis, W. E. (2002). Sturgeon biodiversity and conservation. Kluwer, Dordrecht.

Björnsson, B., Steinarsson, A., and Arnason, T. (2007). Growth model for Atlantic cod (Gadus morhua): Effects of temperature and body weight on growth rate. Aquaculture, 271:216–226.

Blackett, R. F. (1962). Some phases in the life history of the Alaskan blackfish, Dallia pectoralis. Copeia, 1962:124–130.

Blažek, R., Polačik, M., and Reichard, M. (2013). Rapid growth, early maturation and short generation time in African annual fishes. EvoDevo, 4:24.

Blaxter, J. H. S. (1968). Rearing herring larvae to metamorphosis and beyond. Journal of the Marine Biological Association of the United Kingdom, 49:17–28.

Blaxter, J. H. S., Danielssen, D., Moksness, E., and Øiestad, V. (1983). Description of the early development of the halibut Hippoglossus hippoglossus and attempts to rear the larvae past first feeding. Marine Biology, 73:99–107.

Bochkarev, N. A., Zuykova, E. I., Pestryakova, L. A., Zakharov, E. S., Romanov, V. I., Sokolov, V. V., and Politov, D. V. (2018). Siberian whitefish (Coregonus lavaretus pidschian, Coregonidae) from the Anabar River: Morphogenetic structure of the population. Russian Journal of Genetics, 54(9):1078–1088.

Bodola, A. (1964). Life history of the gizzard shad, Dorosoma cepedianum (Le Sueur), in western Lake Erie. Fishery Bulletin of the Fish and Wildlife Service, 65:391–425.

Boehler, S. (2012). The fathead minnow embryo as a model for the development of alternative testing methods in ecotoxicology. Diplomarbeit, University of Heidelberg.

Bolle, L. J., Dickey-Collas, M., van Beek, J. K. L., Erftemeijer, P. L. A., Witte, J. I., van der Veer, H. W., and Rijnsdorp, A. D. (2009). Variability in transport of fish eggs and larvae. iii. Effects of hydro-dynamics and larval behaviour on recruitment in plaice. Mar Ecol Prog Ser, 390:195–211.

Bolle, L. J., Rijnsdorp, A. D., van Neer, W., Millner, R. S., van Leeuwen, P. I., Ervynck, A., Ayers, R., and Ongenae, E. (2004). Growth changes in plaice, cod, haddock and saithe in the North Sea: a comparison of (post-)medieval and present-day growth rates based on otolith measurements. Journal of Sea Research, 51.

Bond, M. J., Jones, N. E., and Haxton, T. J. (2016). Growth and life history patterns of a small-bodied stream fish, Cottus cognatus, in hydropeaking and natural rivers of Northern Ontario. River Res. Applic., 32:721–733.

Bonnett, M. L. (1990). Age and growth of alpine galaxias (Galaxias paucispondylus Stokell) and longjawed galaxias (G. prognathus Stokell) in the Rangitata River, New Zealand. New Zealand Journal of Marine and Freshwater Research, 24.

Bonnett, M. L. (1992). Spawning in sympatric alpine galaxias (Galaxias paucispondylus Stokell) and longjawed galaxias (G. prognathus Stokell) in a South Island, New Zealand, high-country stream. New Zealand Natural Sciences, 19.

Borges, L. (2000). Age and growth of the snipefish, Macrorhamphosus spp., in the Portuguese continental waters. J. Mar. Biol. Ass. UK, 80:147–153.

Boros, G., Sály, P., and Vanni, M. J. (2015). Ontogenetic variation in the body stoichiometry of two fish species. Oecologia, 179:329–341.

Bortone, S. A. (2003). Biology of the spotted seatrout. CRC Press.

Bouchard, C., Mollard, S., Suzuki, K., Robert, D., and Fortier, L. (2016). Contrasting the early life histories of sympatric Arctic gadids Boreogadus saida and Arctogadus glacialis in the Canadian Beaufort Sea. Polar Biology, 39(6):1005–1022.

Bouchereau, J. L., Quignard, J. P., Tomansi, J. A., and Capape, C. (1990). Sexual cycle, condition, fecundity and spawning of Pomatoschistus minutus (Pallas, 1770) (Gobiidae), from the Gulf of Lion, France. Cybium, 14:251–267.

Bouchereau, J. L., Quignard, J. P., Tomansi, J. A., Joyeux, J. C., and Capape, C. (1989). La population de Pomatoschistus minutus (Pallas, 1770) de la Lagune de Mauguio, France. Cah. Biol. Mar., 30:487–514.

Boufersaoui, S. (2016). Biologie et évaluation des stocks de trois sparidés de la région centre de l'algérie: Lithognathus mormyrus (Linnaeus, 1758), Pagrus pagrus (Linnaeus, 1758) et Pagellus acarne (Risso, 1826).

Boufersaoui, S., Meradef, B., and Samer, A. (2021). Effet des paramètres du milieu sur la reproduction et la dynamique de population de Pegusa lascaris (Risso, 1810) des eaux centre algériennes selon la théorie DEB. Technical report, ENSSMAL Univ., Algiers.

Boufersaoui, S. and Saidani, M. (2015). Technical report, ENSSMAL Univ., Algiers.

Boufersaoui, S. and Tamourt, A. (2018). Elaboration d' outil de simulation de l' evaluation et de la gestion de pecheries mixtes multiflottiles. Technical report, ENSSMAL Univ., Algiers.

Boufersaoui, S. and Zeroual, H. (2018). Estimation des paramètres du modèle bioénergétique DEB pour étudier la croissance et la reproduction de quelques espèces des côtes algériennes. Technical report, ENSSMAL Univ., Algiers.

Boughamou, N., Derbal, F., and Kara, H. (2015). Aspects of the reproductive biology of peacock wrasse Symphodus tinca (Linnaeus, 1758) (Labridae) off the coast of North-Eastern Algeria. Cahiers de Biologie Marine, 56:127–136.

Boujard, T., Lecomte, F., Renno, J.-F., Meunier, F., and Neveu, P. (1991). Growth in four populations of Leporinus friderici (Bloch, 1794) (Anostomidae, Teleostei) in French Guiana. Journal of Fish Biology, 38:387–397.

Boulcott, P., Clarke, J., and Wright, P. J. (2017). Effect of size on spawning time in the lesser sandeel Ammodytes marinus. Journal of Fish Biology, 91:362–367.

Boy, C. C., Morriconi, E., and Calvo, J. (2007). Reproduction in puyen, Galaxias maculatus (Pisces: Galaxiidae), in the southernmost extreme of distribution. J. Appl. Ichthyol., 23.

Braasch, M. E. and Smith, P. W. (1967). The life history of the slough darter, Etheostoma gracile, (Pisces, Percidae. Technical report, Dept Energy and Natural Resources; Illinois Institute for Natural Resources.

Braunbeck, T., Hinton, D. E., and Streit, B. (1998). Fish ecotoxicology. Birkuäser.

Bregazzi, P. R. and Kennedy, C. R. (1980). The biology of pike, Esox lucius L., in southern eutrophic lakes. J. Fish Biol., 17:91.

Brickle, P., Arkhipkin, A. I., and Shcherbich, Z. N. (2005a). Age and growth in a temperate euryhaline notothenioid, Eleginops maclovinus from the Falkland Islands. J. Mar. Biol. Ass. U.K., 85:1217–1221.

Brickle, P., Laptikhovsky, V., Arkhipkin, A., and Portela, J. (2005b). Reproductive biology of Patagonotothen ramsayi (Regan, 1913) (Pisces: Nototheniidae) around the Falkland Islands. Polar Biol, NA:NA.

Brodte, E. (2001). Wachstum und Fruchtbarkeit der Aalmutterarten Zoarces viviparus (L.) und Pachycara brachycephalum (Pappenheim) aus unterschiedlichen klimatischen Regionen. PhD thesis, University of Bremen.

Brouwer, S. L. and Griffiths, M. H. (2004). Age and growth of Argyrozona argyrozona (Pisces: Sparidae) in a marine protected area: an evaluation of methods based on whole otoliths, sectioned otoliths and mark-recapture. Fisheries Research, 67:1–12.

Brown, J. O. (2016). Environmental effects on fecundity in mothers of the least killifish, Heterandria formosa. PhD thesis.

Brown, L. R. (1990). Age, growth, feeding, and behavior of Sacramento squawfish (Ptychocheilus grandis) in Bear Creek, Colusa Co., California. The Southwestern Naturalist, 35(3):249–260.

Brownell, C. L. (1983). Early growth rate and feeding of a small group of laboratory-reared saury, Scomberesox saurus scombroides (Pisces: Scomberesocidae). South African Journal of Marine Science, 1:245–248.

Bruch, R. M. (2008). Modeling the population dynamics an sustainability of lake sturgeon in the Winnebago system. Wisconsin. PhD thesis, Univeristy of Wisconsin-Milwaukee.

Bruch, R. M., Miller, G., and Hansen, M. J. (2006). Fecundity of lake sturgeon (Acipenser fulvescens, Rafinesque) in Lake Winnebago, Wisconsin, USA. J. Appl. Ichthyol., 22 (Suppl 1):116–118.

Bruger, G. (1974). Age, growth, food habits, and reproduction of bonefish Albula vulpes, in South Florida Waters. Technical report.

Bundy, D. S. (1970). Reproduction and growth of the Tule perch Hysterocarpus traskii (Gibbons) with notes on its ecology. PhD thesis, University of the Pacific.

Burchett, M. S. (1983). Age and growth of the Antarctic fish Notothenia rossi from South Georgia. Br. Antacrt. Surv. Bull., 60:45–61.

Burr, B. M. and Mayden, R. L. (1982). Life history of the brindled madtom Noturus miurus in Mill Creek, Illinois (Pisces: Ictaluridae). The American Midland Naturalist, 107(1):25–41.

Burr, B. M. and Page, L. M. (1978). Life-history aspects of the cypress darter, Etheostoma proeliare in max creek, illinois. Technical report, Illinois Natural history survey, Urbana, Illinois.

Burton, M. L. (2002). Age, growth and mortality of mutton snapper, Lutjanus analis, from the east coast of Florida, with a brief discussion of management implications. Fisheries Research, 59:31–41.

Buslov, A. V., Sergeeva, N. P., and Ilyin, O. I. (2010). Embryonic development of the Pacific cod Gadus macrocephalus (Gadidae). Russian Journal of Marine Biology, 36(7):526–538.

Byrne, D. M. (1976). Life History of the Spotfin Killififish, Fundulus luciae (Pisces, Cyprinodontidae), in Fox Creek Marsh, Virginia. PhD thesis, College of William and Mary in Virginia.

Bystydzieńska, Z. E., Phillips, A. J., and Linkowski, T. B. (2010). Larval stage duration, age and growth of blue lanternfish Tarletonbeania crenularis (Jordan and Gilbert, 1880) derived from otolith microstructure. Environ Biol Fish, 89.

Cabiddu, S., Follesa, M. C., Porcu, C., and Cau, A. (2010). Gonad development and reproduction in the monoecious species Chlorophthalmus agassizi (Actinopterygii: Aulopiformes: Chlorophthalmidae) from the Sardinian waters (central-western Mediterranean). ACTA ICHTHYOLOGICA ET PISCATORIA, 40(2).

Caiola, N. A., Vargas, M. J., and de Sostoa, A. (2001). Life history pattern of the endangered Valencia toothcarp, Valencia hispanica (Actinopterygii: Valenciidae) and its implications for conservation.

Caldwell, C. A., Falco, H., Knight, W., Ulibarri, M., and Gould, W. R. (2019). Reproductive potential of captive rio grande silvery minnow. North American Journal of Aquaculture, 81:47–54.

Cali, F., Riginella, E., Mesa, M. L., and Mazzoldi, C. (2017). Life history traits of Notothenia rossii and N. coriiceps along the southern Scotia Arc. Polar Biology, ?:?

Calta, M. (2000). Morphological development and growth of chub, Leuciscus cephalus, larvae. J. Appl. Ichthyol., 16:83–85.

Canavate, J. P. and Fernandez-Diaz, C. (1999). Influence of co-feeding larvae with live and inert diets on weaning the sole *Solea senegalensis* onto commercial dry feeds. Aquaculture, 174(3-4):255–263.

Carballo, C., Firmino, J., Anjos, L., Santos, S., Power, D. M., and Manchado, M. (2018). Short- and long-term effects on growth and expression patterns in response to incubation temperatures in Senegalese sole. Aquaculture, 495(April):222–231.

Cardoso, L. G. and Haimovici, M. (2011). Age and changes in growth of the king weakfish Macrodon atricauda (günther, 1880) between 1977 and 2009 in southern Brazil. Fisheries Research, 111:177–187.

Carney, D. A. and Burr, B. M. (1989). Life histories of the bandfin darter, Etheostoma zonistium, and the firebelly darter, Etheostoma pyrrhogaster, in Western Kentucky. Technical report, Illinois Natural History Survey.

Cartwright, J. W. (1959). Egg size and egg number in some freshwater fish of British Columbia. PhD thesis, Univ. of British Columbia.

Cartwright, M., Beauchamp, D., and M.D., B. (1998). Quantifying cutthroat trout (Oncorhynchus clarkii) predation on sockeye salmon (Oncorhynchus nerka) fry using a bioenergetics approach. Canadian journal of fisheries and aquatic sciences, 55(5):1285–1295.

Carufel, L. H. (1963). Life history of saugers in Garrison Reservoir. The Journal of Wildlife Management, 27(3):450–456.

Carvalho, F. M. and Castello, J. P. (2013). Argentine anchovy (Engraulis anchoita) stock identification and incipient exploitation in southern Brazil. Lat. Am. J. Aquat. Res., 41:820–827.

Carvalho, M. G., Moreira, C., Rodrigues, S., Queiroga, H., Santos, P. T., and Correia, A. T. (2012). Age, growth and sex of Lipophrys pholis from the north coast of Portugal. http://www.fc.up.pt/pessoas/ptsantos/artigos/carvalhoetal2014PosterAgegrowthsex_Lipophrys_pholis.pdf.

Casas, J. M. and Pineiro, C. (2000). Growth and age estimation of greater fork-beard (Phycis blennoides brünnich, 1768) in the north and northwest of the Iberian Peninsula (ICES Division VIIIc and IXa). Fisheries Research, 47:19–25.

Casazza, T. L., Ross, S. W., Necaise, A. M., and Sulak, K. J. (2005). Reproduction and mating behavior of Cheilopogon melanurus off North Carolina. http://fl.biology.usgs.gov/posters/Coastal_Ecology/Cheilopogon_melanurus/cheilopogon_melanurus.html.

Castellani, C. and Edwards, M. (2017). Marine Plankton: A practical guide to ecology, methodology, and taxonomy. Oxford Univ. Press.

Castello, L. and Catello, J. P. (2003). Anchovy stocks (Engraulis anchoita) and larval growth in the SW Atlantic. Fisheries Research, 50:409–421.

Castillo-Jordán, C., Cubillos, L. A., and Navarro, E. (2010). Inter-cohort growth rate changes of common sardine (Strangomera bentincki) and their relationship with environmental conditions off central southern Chile. Fisheries Research, 105:228–236.

Catalan, I. A., Folkvord, A., Palomers, I., Quilez-Badia, G., Kallianoti, F., Tselepides, A., and Kallianotis, A. (2012). Growth and feeding patterns of european anchovy (Engraulis encrasicolus) early life stages in the Aegean Sea (NE Mediterranean). Estuarine Coastal and Shelf Science, 86:299–312.

Caulton, M. S. (1977). The effect of temperature on routine metabolism in Tilapia rendalli Boulenger. Journal of Fish Biology, 11(6):549–553.

Caulton, M. S. (1982). Feeding, metabolism and growth of tilapias: some quantitative considerations. In Pullin, R. S. V. and Lowe-McConnell, R. H., editors, The biology and culture of tilapias, ICLARM conference proceedings 7, pages 157–183, Manila, Philippines. ICLARM.

Çoker, T., Akyol, O., Özaydın, O., Leblebici, S., and Tosunoğlu, Z. (2008). Determination of batch fecundity in Uranoscopus scaber Linnaeus, 1758 from the Aegean Sea, Turkey. J. Appl. Ichthyol., 24:85–87.

Chai, X., Zhu, Y., Wang, Y., and Hu, Z. (2015). Study on the embryonic development of Trichiurus japonicus in the East China Sea. Journal of Zhejiang Ocean University (Natural Science), 34:429–432.

Chale-Matsau, J., Govender, A., and Beckley, L. (1999). Age and growth of the queen mackerel Scomberomorus plurilineatus from KwaZulu-Natal, South Africa. Fisheries Research, 44:121–127.

Chan, M. D. and Parsons, G. R. (2000). Aspects of brown madtom, Noturus phaeus, life history in northern Mississippi. Copeia, 2000(3):757–762.

Chandrasoma, J. and De Silva, S. S. (1981). Reproductive biology of Puntius sarana, an indigenous species, and Tilapia rendalli (melanopleura), an exotic, in an ancient man-made lake in Sri Lanka. Aquaculture Research, 12(1):17–28.

Chang, B. D. and Navas, W. (1984). Seasonal variations in growth, condition and gonads of Dormitator latifrons (Richardson) in the Chone River Basin, Ecuador. J. Fish Biol., 24:637–647.

Chebanov, M. S. and Galich, E. V. (2013). Sturgeon Hatchery Manual.

Chernoivanova, L. A. (2017). Growth of young Pacific herring Clupea pallasii of Peter the Great Bay (Sea of Japan). Journal of Ichthyology, 57:372–379.

Cherr, G. N. and Clark, W. H. (1982). Fine structure of the envelope and micropyles in the eggs of the white sturgeon, Acipenser transmontanus Richardson. Develop., Growth and Differ, 24:341–352.

Chigbu, P. and Sibly, T. H. (1994). Relationships between abundance, growth, egg size and fecundity in landlocked population of longfin smelt Spirinchus thaleichthys. J. Fish Biol., 45:1–15.

Childress, J. J., Taylor, S. M., Cailliet, G. M., and Price, M. H. (1980). Patterns of growth, energy utilization and reproduction in some meso- and bathypelagic fishes off Southern California. Marine Biology, 61.

Choat, J. H. and Axe, L. M. (1996). Growth and longevity in acanthurid fishes; an analysis of otolith increments. Mar Ecol Prog Ser, 134:15–26.

Choo, C. K. and Liew, H. C. (2006). Morphological development and allometric growth patterns in the juvenile seahorse Hippocampus kuda Bleeker. Journal of Fish Biology, 69:426–445.

Chowdhury, M. A., Roy, N. C., and Chowdhury, A. (2020). Growth, yield and economic returns of striped catfish (Pangasianodon hypophthalmus) at different stocking densities under floodplain cage culture system. Egyptian Journal of Aquatic Research, 46:91–95.

Chung, K. and Woo, N. Y. S. (1999). Age and growth by scale analysis of pomacanthus imperator (teleostei: Pomacanthidae) from dongsha islands, southern china. Environmental Biology of Fishes, 55:399–412.

Cisneros-Mata, M. A., Montemayor-López, G., and Romá-Rodríguez, M. J. (1995). Life history and conservation of Totoaba macdonaldi. Conservation Biology, 9:806–814.

Clark, F. N. (1925). The life history of Leuresthes tenuis, an atherine fish with tide controlled spawning habits. Technical report, STATE OF CALIFORNIA FISH AND GAME COMMISSION.

Clarke, A. D., Lewis, A., Telmer, K. H., and Shripton, J. M. (2007). Life history and age at maturity of an anadromous smelt, the eulachon Thaleichthys pacificus (Richardson). Journal of Fish Biology, pages 1479–1493.

Clarke, W. C., Jensen, J. O. T., Klimek, J., and Pakula, Z. (1999). Rearing of sablefish (Anoplopoma fimbria) from egg to juvenile. Bull. Aquacul. Assoc. Canada, 99:11–12.

Clay, T. A. (2004). Growth, survival and cannibalism rates of alligator gar Atractosteus spatula in recirculating aquaculture systems. PhD thesis, Thesis Univ Maryland, Baltimore.

Clugston, J. P. and Cooper, E. L. (1982). Growth of the common eastern madtom, Noturus insignis in Central Pennsylvania. The American Midland Naturalist, 1960(1):9–16.

Coad, B. W. (2018). `http://www.briancoad.com/SpeciesAccounts/DescriptionandAcipenseridaetoChanidae.htm`.

Coburn, M. M. (1986). Egg diameter variation in Eastern North American Minnows (Pisces: Cyprinidae): Correlation with vertebral number, habitat and spawning behavior. The Ohio Journal of Science, 86(1):110–120.

Cohen, D. M., Inada, T., Iwamoto, T., Scialabba, N., and Whitehead, P. J. P. (1990). FAO species catalogue: vol. 10 gadiform fishes of the world (order gadiformes), an annotated and ilustrated

catalogue of Cods. Hakes, grenadiers and other gadiform fishes known to date, volume 10 of 125. FOOD AND AGRICULTURE ORGANIZATION OF THE UNITED NATIONS.

Cole, B., Kotol, P., and Haring, M. (1999). Spawning and production of the lemon tetra Hyphessobrycon pulchripinnis. Technical Report 142, Center for Tropical and Subtropical Aquaculture.

Cole, K. S. (2010). Reproduction and sexuality in marine fishes: Patterns and processes. Univ of California Press.

Coleman, M. and Fausch, K. (2007). Cold summer temperature regimes cause a recruitment bottleneck in age-0 Colorado River cutthroat trout reared in laboratory streams. Transactions of the american fisheries society, 136(3):639–654.

Collins, M. A., Shreeve, R. S., Fielding, S., and Thurston, M. H. (2008). Distribution, growth, diet and foraging behaviour of the yellow-fin notothen Patagonotothen guntheri (Norman) on the Shag Rocks shelf (Southern Ocean). Journal of Fish Biology, 72:271–286.

Comfort, A. (1983). Effect of delayed and resumed growth on the longevity of a fish (Lebistes reticulatus Peters) in captivity. Gerontologia (Basel), 8:150–155.

Connell, A. (1996). Sea fishes spawning pelagic eggs in the St Lucia Estuary. South African Journal of Zoology, 31(1):37–41.

Conover, D. O. and Ross, M. R. (1982). Patterns in seasonal abundance, growth and biomass of the Atlantic silverside, Menidia menidia, in a New England estuary. Estuaries, 5:275–286.

Contreras-Reyes, J. E. and Arellano-Valle, R. B. (2013). Growth estimates of cardinalfish (Epigonus crassicaudus) based onscale mixtures of skew-normal distributions. Fisheries Research, 147:137–144.

Contreras-Reyes, J. E., Arellano-Valle, R. B., and Canales, T. M. (2014). Comparing growth curves with asymmetric heavy-tailed errors: Application to the southern blue whiting (Micromesistius australis). Fisheries Research, 159.

Copp, G. H., Tarkan, A. S., Masson, G., Godard, M. J., Koščo, J., Kováč, V., Novomeská, A., Miranda, R., Cucherousset, J., Pedicillo, G., and Blackwell, B. G. (2016). A review of growth and life-history traits of native and non-native european populations of black bullhead Ameiurus melas. Rev Fish Biol Fisheries, 26:441–469.

Cossington, S., Hesp, S. A., Hall, N. G., and Potter, I. C. (2010). Growth and reproductive biology of the foxfish Bodianus frenchii, a very long-lived and monandric protogynous hermaphroditic labrid. Journal of Fish Biology, 77:600–626.

Costa, L. R. F., Barthem, R. B., Albernaz, A. L., Bittencourt, M. M., and Villacorta-Correa, M. A. (2013). Modelling the growth of tambaqui, Colossoma macropomum (Cuvier, 1816) in floodplain lakes: model selection and multimodel inference. Braz. J. Biol., 73:397–340.

Couillard, M. A., Cabana, G., Dery, J. F., Daigle, G., and Dodson, J. J. (2011). Ontogenetic habitat shifts of the Atlantic tomcod (Microgadus tomcod) across an estuarine transition zone. Estuaries and Coasts, 34.

Coulson, P. G., Potter, I. C., and Hall, N. G. (2012). The biological characteristics of Scorpis aequipinnis (Kyphosidae), including relevant comparisons with those of other species and particularly of a heavily exploited congener. Fisheries Research, 125-126:272–282.

Cowley, D. E., Alleman, J., McShane, R. R., Shirey, P. D., and Sallenave, R. (2013). Cosewic assessment and status report on the cutlip minnow Exoglossum maxillingua in Canada. Technical report.

Cowley, D. E., Shirey, P. D., and Hatch, M. D. (2006). Ecology of the Rio Grande silvery minnow (Cyprinidae: Hybognathus amarus) inferred from specimens collected in 1874. Reviews in Fisheries Science, 14(1-2):111–125.

Crabtree, R. E., Cyr, E., and Dean, J. M. (1995). Age and growth of tarpon, Megalops atlanticus, from south Florida waters. Fishery Bulletin, 93:619–628.

Craig, D. and FitzGerald, G. J. (1982). Reproductive tactics of four sympatric sticklebacks (Gasterosteidae). Ehv. Biol. Fish., 7(4):369–375.

Craig, P. C., Griffiths, W. B., Haldorson, L., and McElderry, H. (1982). Ecological studies of Arctic cod (Boreogadus saida) in Beaufort Sea coastal waters, Alaska. Canadian Journal of Fisheries and Aquatic Sciences, 39(3):395–406.

Craig, P. C. and Poulin, V. A. (1975). Movements and growth of Arctic grayling (Thymallus arcticus) and juvenile Arctic char (Salvelinus alpinus) in a small Arctic stream, Alaska. Journal of the Fisheries Research Board of Canada, 32(5):689–697.

Crane, D. P., Cornett, M. R., Bauerlien, C. J., Hawkins, M. L., Isermann, D. A., Hansbarger, J. L., Kapuscinski, K. L., Meerbeek, J. R., Simonson, T. D., and Kampa, J. M. (2020). Validity of age estimates from muskellunge (Esox masquinongy) fin rays and associated effects on estimates of growth. Canadian Journal of Fisheries and Aquatic Sciences, 77:69–80.

Crechriou, R., Zintzen, V., Moore, L., and Roberts, C. D. (2015). Length-weight relationships of 33 fish species from New Zealand. J. Appl. Inchthyol., 31:558–561.

Cripe, G. M., Hemmer, B. L., and Goodman, L. R. (2008a). Development of a methodology for successful multigeneration life-cycle testing of the estuarine sheepshead minnow, Cyprinodon variegatus. Archives of Environmental Contamination and Toxicology, 56:500–508.

Cripe, G. M., Hemmer, B. L., and Goodman, L. R. (2008b). Development of a methodology for successful multigeneration life-cycle testing of the estuarine sheepshead minnow, Cyprinodon variegatus. Archives of Environmental Contamination and Toxicology, 56:500–508.

Crivelli, A. J. (1981). The biology of the common carp Cyprinus carpio l in the camargue, southern france. Technical report, Station biologic de la Tour du Valat, Arles, France.

Cuellar, N., Sedberry, G. R., and Wyanski, D. M. (1996). Reproductive seasonality, maturation, fecundity, and spawning frequency of the vermilion snapper Rhomboplites aurorubens, off the southeastern united states. Fishery Bulletin, 94:635–653.

Cummings, K. S., Grady, J. M., and Burr, B. M. (1984). The life history of the mud darter, Etheostoma asprigene, in Lake Creek, Illinois. Technical report, Dept Energy and Natural Resources.

Cunha, M., Quental, H., Barradas, A., Pousoã-Ferreira, P., Cabrita, E., and Engrola, S. (2009). Rearing larvae of dusky grouper, Epinephelus marginatus (Lowe, 1834), (Pisces: Serranidae) in a semi-extensive mesocosm. Scientia Marina, 73(S1):201–212.

Cunha, N. L., Catella, A. C., and Kinas, M. A. (2007). Growth parameters estimates for a small fish of the Pantanal, Brazil: Moenkhausia dichroura (Characiformes; Characidae). Braz. J. Biol., 67:293–297.

Cunningham, J. T. (1891). On the reproduction and development of the conger. J. Mar. Biol. Assoc. U.K., 2:16–42.

Curtis, J. M. R. and Vincent, A. C. J. (2006). Life history of an unusual marine fish: survival, growth and movement patterns of Hippocampus guttulatus Cuvier 1829. Journal of Fish Biology, 68:707–733.

da Fonseca, A. P., Volcan, M. V., Sampaio, L. A., Romano, L. A., and Robaldo, R. B. (2013). Growth of critically endangered annual fish Austrolebias wolterstorffi (Cyprinodontiformes: Rivulidae) at different temperatures. Neotropical Ichthyology, 11(4):837–844.

da Silva, A. M. A., Maunder, M. N., Schaefer, K. M., and Fuller, D. W. (2015). Improved growth estimates from integrated analysis of direct agingand tag-recapture data: An illustration with bigeye tuna (Thunnus obesus) of the eastern Pacific Ocean with implications for management. Fisheries Research (), 163:119–126.

Dahle, S. P. (2001). Studies of Topeka shiner (Notropis topeka) life history and distribution in Minnesota. PhD thesis, University of Minnesota.

Dahlke, F., Nahrgang, J., Mortensen, A., Puvanendran, V., Pörtner, H.-O., and Storch, D. (2017). Supplement to: Dahlke, F et al. (in prep.): Effects of ocean warming and acidification on Atlantic cod and polar cod.

Damme, v. (2007). excelsheet: Mackerel fecundity 2007 v1_1. Survey data on fecundity.

Danancher, D., Labonne, J., Gaudin, P., and Joly, P. (2007). Scale measurements as a conservation tool in endangered Zingel asper (Linnaeus, 1758). Aquatic Conserv: Mar. Freshw. Ecosyst., 17:712–723.

Daniel, M., Faria, R., Cavalcante-Neto, A., Allaman, I. B., Gomes, A. D., aes Moreira, R. G., Hallerman, E. M., and Hilsdorf, A. W. S. (2019). The potential of Hoplias malabaricus (Characiformes: Erythrinidae), a Neotropical carnivore, for aquaculture. Aquaculture and Fisheries, 4:89–97.

Daniels, R. A. (1987). Comparative life histories and microhabitat use in three sympatric sculpins (Cottidae: Cottus) in northeastern California. Environmental Biology of Fishes, 19(2):93–110.

Datsky, A. V. (2016). Biological features of the common fish species in Olyutorsky-Navarin region and the adjacent waters of the Bering Sea: 1. Gadidae (cods) family. Journal of Ichthyology, 56.

Dauble, D. D. (1980). Life history of the bridgelip sucker in the Central Columbia River. Transactions of the American Fisheries Society, 109(1):92–98.

Dauble, D. D. (1986). Life history and ecology of the largescale sucker (Castostomus macrocheilus) in the Columbia River. The American Midland Naturalist, 116(2):356–367.

Davidson, J. W., Kenney, P. B., Manor, M., Good, C. M., Weber, G. M., Aussanasuwannakul, A., Turk, P. J., Welsh, C., and Summerfelt, S. T. (2014). Growth performance, fillet quality, and reproductive maturity of rainbow trout (Oncorhynchus mykiss) cultured to 5 kilograms within freshwater recirculating systems. Journal of Aquaculture Research and Development, 5(4).

Davis, J. R. and Louder, D. E. (1971). Life history and ecology of the cyprinid fish Notropis petersoni in North Carolina waters. Transactions of the American Fisheries Society, 100(4):726–733.

Dawson, K. (2021). Age, growth, and otolith microchemistry of the golden tilefish (Lopholatilus chamaeleonticeps) in the NW Atlantic. PhD thesis, Michigan Technological University.

Day, O. J., Jones, D. A., and Howell, B. R. (1996). Food consumption, growth and respiration of sole, *Solea solea* (L.), during early ontogeny in a hatchery environment. Aquaculture Research, 27:831–839.

Dayaratne, P. and Gjøsaeter, J. (1986). Age and growth of four Sardinella species from Srilanka. Fisheries Research, 4:1–33.

de Albuquerque, C. Q., Martins, A. S., de Oliveira Leite Jr, N., de Araujo, J. N., and Ribeiro, A. M. (2011). Age and growth of the queen triggerfish Balistes vetula (Tetraodontiformes, Balistidae) of the central coast of Brazil. Brazilian Journal of Oceonagraphy, 59:231–239.

de Ciechomski, J. D. (2013). Development of the larvae and variations in the size of the eggs of the Argentine anchovy, Engraulis anchoita Hubbs and Marini. ICES Journal of Marine Science, 30:281–290.

de Laak, G. A. J. (2009). Kennisdocument elft. Technical report, Sportvisserij Nederland.

de Laak, G. A. J. (2010). Kennisdocument blankvoorn Rutilus rutilus (Linnaeus, 1758). Technical report, Sportvisserij Nederland.

de León, J. L. P., Rodríguez, R., and Leó, G. (2013). Life-history patterns of Cuban poeciliid fishes (Teleostei: Cyprinodontiformes). Zoo biology, 32:251–256.

de Santana, C. D., Vari, R. P., and Wosiacki, W. B. (2013). The untold story of the caudal skeleton in the electric eel (Ostariophysi: Gymnotiformes: Electrophorus). PlosOne, July 24.

de Wilt, R. S. and van Emmerik, W. A. M. (2008). Kennisdocument karper. Cyprinus carpio (linnaeus 1758). Technical report, Sportvisserij Nederland.

de Zárate, V. O. and Babcock, E. A. (2016). Estimating individual growth variability in albacore (Thunnus alalunga) from the North Atlantic stock: Aging for assessment purposes. Fisheries Research, 180 ():54–66.

Deacon, J. E., Taylor, F. R., and Pedretti, J. W. (1995). Egg viability and ecology of devils hole pupfish: Insights from captive propagation. The Southwestern Naturalist, 40(2):216–223.

Deacon, J. E. and Williams, J. E. (2010). Retrospective evaluation of the effects of human disturbance and goldfishintroduction on endangered pahrump poolfish. Western North American Naturalist, 70(4):425–436.

Deason, H. J. and Hile, R. (1947). Age and growth of the kiyi, Leucichthys kiyi Koelz, in Lake Michigan. Transactions of the American Fisheries Society, 74(1):88–142.

Degoon, A. S. O. A. and Ali, S. M. (2013). On some reproductive aspects of the sky emperor, Lethrinus mahsena (Pieces) in the Sudanese Red Sea. SJBS (B), 17:51–62.

Delsman, H. C. (1930). Fish eggs and larvae from the Java Sea on Chirocentrus hypelosoma and dorab. Treubia, 12:46–50.

DeMartini, E. E., Moore, T. O., and Plummer, K. M. (1983). Reproductive and growth dynamics of hyperprosopon argenteum (embiotocidae) near san diego, california. Env. Biol. Fish., 8:29–38.

Demirhan, S. A., Can, M. F., and Seyhan, K. (2007). Age and growth of stargazer (Uranoscopus scaber L., 1758) in the southeastern Black Sea. J. Appl. Ichthyol., 23:4692–694.

Deniel, C. (1981). Les poissons plats [Teleosteens, Pleuronectiformes] en baie de Douarnenez : reproduction, croissance et migration. PhD thesis, Universite de Bretagne Occidentale - Brest.

Deniel, C. (1985). Le trident Raniceps raninus (Linnaeus, 1758) (Teleosteen, Gadidae) de la Cote Nord du Finistere: croissance en longueur et relation longueur-masse. Cybium, 9.

Deniel, C. (1990). Comparative study of growth of flatfishes on the west coast of Brittany. Journalof Fish Biology, 37.

Denoncourt, R. F. and Messersmith, J. F. A. (1982). Growth and reproduction of the satinfin shiner, Cyprinella analostana, Codorus Creek, Pennsylvania. Proceedings of the Pennsylvania Academy of Science, 56(1):81–86.

Dessier, A. (2015). Analyse du compartiment mésozooplanctonique et écologie alimentaire printanière de la sardine, Sardina pilchardus (Walbaum, 1782), et de l'anchois, *Engraulis encrasicolus* (Linné, 1758) adultes dans le Golfe de Gascogne. PhD thesis, Univ. La Rochelle, La Rochelle.

Dettlaff, A., Ginsburg, A. S., and Schmalhausen, O. I. (1993). Sturgeon Fishes: developmental Biology and Aquaculture. Springer-Verlag, Berlin.

Devauchelle, N., Alexandre, J. C., Corre, N. L. E., and Letty, Y. (1987). Spawning of sole (*Solea solea*) in captivity. Aquaculture, 66:125–147.

Dieterman, D. J., Roberts, E., Braaten, P. J., and Galat, D. L. (2006). Reproductive development in the sicklefin chub in the Missouri and Lower Yellowstone Rivers. The Prairie Naturalist, 28:113–130.

Dikel, S., Alev, M. V., Kiriş, G. A., and Kumlu, M. (2002). Growth and yield of two tilapia species Tilapia zillii and Tilapia rendalli raised in floating cages in Seyhan Dam Lake. Journal of the Faculty of Agriculture, 17(2):93–98.

Dinis, Maria Teresa ans Ribeiro, L., Soares, F., and Sarasquete, C. (1999). A review on the cultivation potential of *Solea senegalensis* in Spain and in Portugal. Aquaculture, 176(1-2):27–38.

Dinkins, G. R. and Shute, P. W. (1996). Life histories of Noturus baileyi and Noturus flavipinni (Pisces; Ictaluridae) two rare madtom catfishes in Citico Creek, Monroe County, Tennessee. Bulletin Alabama Mus. Nat. Hist., 18:43–69.

Divanach, P., Kentouri, M., and Tiselius, P. (1983). Données préliminaires sur la technique de production, la croissance et la survie des larves de marbré Lithognathus mormyrus. Aquaculture, 31:245–256.

Dominguez-Castanedo, O., Uribe, M. C., and Rosales-Torres, A. M. (2017). Life history strategies of annual killifish Millerichthys robustus (Cyprinodontiformes: Cynolebiidae) in a seasonally ephemeral water body in Veracruz, México. Environ Biol Fish.

Donald, D. B. and Kooyman, A. H. (2011). Food, feeding habits, and growth of goldeye, Hiodon alosoides(Rafinesque), in the waters of the Athabasca Delta. Ca. J. Zool., 55:1038–1047.

D'Onchia, G., Basanisi, M., and Tursi, A. (2000). Population structure, age and growth of macrourid fish from the upper slope of the Eastern-Central Mediterranean. Journal of Fish Biology, 56:1217–1238.

Donkers, P. (2011). Age, growth and maturity of the common carp (Cyprinus carpio) in lake crescent and sorell. Technical report, Inland Fisheries Service, Tasmania, Australia.

Dorel, D. (1986). Poissons de l Atlantique Nord-Est: relations taille-poids. IFREMER.

Döring, Neumann, S. I., Sloterdijk, H., and Ekau, W. (2018). Seasonal growth differences of larval Hemirhamphus picarti (Hemiramphidae) in the Sine Saloum estuary, Senegal. Fisheries Research, 34:97–102.

dos Santos, A. M. V. and Rossi-Wongtschowski, C. L. D. B. (2007). Age and growth of the Argentine hake Merluccius hubbsi Marini, 1933 in the Brazilian south-Southeast Region during 1996-2001. Neotropical Ichthyology, 5.

Dovel, W. (1960). Larval development of the oyster toadfish, Opsanus tau. Chesapeake Sci., 1(3-4):187–195.

Drake, D., Power, M., Koops, M. A., Doka, S. E., and Mandrak, N. E. (2008). Environmental factors affecting growth of eastern sand darter (Ammocrypta pellucida). Can. J. Zool., 86:714–722.

Drew, R. E., Rodnick, K. J., Settles, M., Wacyk, J., Churchill, E., Powell, M. S., Hardy, R. W., Murdoch, G. K., Hill, R. A., and Robison, B. D. (2008). Effect of starvation on transcriptomes of brain and liver in adult female zebrafish (Danio rerio). Physiological Genomics, 35:283–295.

Dua, A. and Kumar, K. (2006). Age and growth patterns in Channa marulius from Harike wetland (a Ramsar site), Punjab, India. Journal of Environmental Biology, 27(2):377–380.

Dulcic, J. (1998). Larval growth of sprat, Sprattus sprattus phalericus, larvae in the Northern Adriatic. Fisheries Research, 36:117–126.

Dulcic, J., Bazdaric, B., Grubisic, L., Tutman, P., and Dragicevic, B. (2009). Embryonic and larval development of garpike from the Adriatic Sea. Integrative Zoology, 4:272–276.

Dulcic, J., Grubisic, L., Katavic, I., and Skakelja, N. (2001). Embryonic and larval development of the tub gurnard Trigle Lucerna (Pisces: Triglidae). J. Mar. Biol. Ass. U.K., 81:313–316.

Dulcic, J. and Kraljevic, M. (1995). Age, growth and mortality of damselfish (Chromis chromis L.) in the eastern middle Adriatic. Fisheries Research, 22:255–264.

Dulcic, J., Kraljevic, M., and Cetinic, P. (1994). Length-weight relationship in damselfish (Chromis chromis L. 1758) from the eastern Adriatic during spawning. Acta Icthyologica et Piscatoria, 24:147–154.

Dulčić, J., Grubišić, L., Pallaoro, A., and Glamuzina, B. (2008). Embryonic and larval development of big-scale sand smelt Atherina boyeri (Atherinidae). Cybium, 32:27–32.

Dulčić, J., Soldo, A., and Jardas, I. (2005). Adriatic sea small-scale fisheries. Technical Report 15, AdriaMed Technical Documents, AdriaMed Technical Consultation on Adriatic Sea Small-Scale Fisheries, Split, Croatia.

Dunbrack, R. and Green, J. M. (2017). Life history differences and latitudinal variation in recruitment in two species of Arctic-Boreal Perciform fishes, the fish doctor Gymnelus viridis and the Arctic Shanny Stichaeus punctatus. Journal of Ichthyology, 57:380–392.

Durant, D. F., Shireman, J. V., and Gasaway, R. D. (1979). Reproduction, growth and food habits of seminole killifish, Fundulus seminolis, from two central Florida lakes. The American Midland Naturalist, 102(1):127–133.

Dwyer, K. S., Treble, M. A., and Campana, S. E. (2016). Age and growth of Greenland halibut (Reinhardtius hippoglossoides) in the Northwest Atlantic: A changing perception based on bombradiocarbon analyses. Fisheries Research, 179:342–350.

Dwyer, K. S., Walsh, S. J., and Campana, S. E. (2003). Age determination, validation and growth of Grand Bank yellowtail flounder (Limanda ferruginea). ICES Journal of Marine Science, 60:1123–1138.

Ebert, V. W. and Summerfelt, R. C. (1969). Contributions to the life history of the piute sculpin, Cottus beldingii Eigenmann abd Eigenmann, in Lake Tahoe. Calif. Fish and Game, 55(2):100–120.

Economou, A. N., Daoulan, C., and Psarras, T. (1991). Growth and morphological development of chub, Leuciscus cephalus (L.), during the first year of life. Journal of Fish Biology, 39:393–408.

Edberg, K. L. and Powers, S. L. (2010). Life-history aspects of Fundulus stellifer (southern studfish) (Actinopterygii: Fundulidae) in Northern Georgia.

Egami, N. and Etoh, H. (1969). Life span data for the small fish, (Oryzias latipes). Exp. Geront., 4:127–129.

Ehrenbaum, E. (1894). Beiträge zur Naturgeschichte einiger Elbfische (Osmerus eperlanus L., Clupea finta Cuv., Acerina cernua L., Acipenser sturio L.). Beilage zu den Mitt Deutsch Seefisch, 10:49.

Einum, S. and Fleming, I. A. (2000). Selection against late emergence and small offspring in Atlantic salmon (Salmo salar). Evolution, 54(2):628–639.

Einum, S., Fleming, I. A., Cote, I. M., and Reynolds, J. D. (2003). Population stability in salmon species: effects of population size and female reproductive allocation. Journal of Animal Ecology, 72:811–821.

El-Halfawy, M. M., Amin, A. M., and Ramadan, A. M. (2007). Growth and reproduction of female brushtooth lizardfish Saurida undosquamis (Richardson) from the Gulf of Suez, Egypt. E.U. Journal of Fisheries and Aquatic Science, 24.

Elliot, J. M. (1975). The growth rate of brown trout (Salmo trutta L.) fed on maximum rations. Journal of Animal Ecology, 44:805–821.

Elliot, J. M. (1984). Numerical changes and population regulation in young migratory trout Salmo trutta in a Lake District stream, 1966-83. Journal of animal ecology, 53:327–350.

Elliot, J. M. (1994). Quantitative ecology and the brown trout. Oxford University Press, USA.

Emery, L. and Wallace, D. C. (1974). The age and growth of the blacknose shiner, Notropis heterolepis Eigenmann and Eigenmann. The American Midland Naturalist, 91(1):242–243.

EPA (2002). Method 1004.0: Sheepshead minnow, Cyprinodon variegatus, larval survival and growth test; chronic toxicity. Technical report, EPA.

Erreaa, A. and Danulatb, E. (2001). Growth of the annual fish, Cynolebias viarius (Cyprinodontiformes), in the natural habitat compared to laboratory conditions. Environmental Biology of Fishes, 61:261–268.

Esin, E. V. (2015). Stream resident Dolly Varden Salvelinus malma of Kamchatka Peninsula. Journal of Ichthyology, 55:224–239.

Esmaeilpour Poodeh, S. and H, R. (2010). A survey on population dynamic of Caspian Spirlin (Alburnoides eichwaldii) in ZarrinGol, Iran. PhD thesis, Gorgan university of Agricultural Sciences and Natural Resources, Iran.

Espitia-Manrique, C. H., ao Batista Kochenborger Fernandes, J., Sakomura, N. K., Ángel Andrés Arias-Vigoya, do Nascimento, T. M. T., da Silva, E. P., and Mansano, C. F. M. (2017). Description of growth and body composition of freshwater angelfish (Pterophyllum scalare) by gompertz model. R. Bras. Zootec, 46(8):631–637.

Estes, C. (1949). The fecundity of the bluegill (Lepomis macrochirus) in certain small east Texas reservoirs. PhD thesis, North Texas State College.

Etnier, D. A. and Starnes, W. C. (1993). The Fishes of Tennessee. The University of Tennessee Press. Knoxville.

Evens, D. H. (1969). Life history studies of the Lahontan redside, Richardsonius egregius, in Lake Tahoe. Calif. Fish and Game, 55(3):197–212.

Eveson, J. P., Million, J., Sardenne, F., and Croizier, G. L. (2015). Estimating growth of tropical tunas in the Indian Ocean usingtag-recapture data and otolith-based age estimates. Fisheries Research, 163:58–68.

Faber, D. (2013). Der Japanische Reiskaerpfling Oryzias latipes als Testorganismus im Fish-Sexual-Development- und Fish-Full-Life-Cycle-Test - Effekte endokrin aktiver Substanzen unter variablen Expositionsszenarien. PhD thesis, Univ. of Aachen.

Fahy, W. E. (1954). The life history of the northern greenside darter, Etheostoma blennioides blennioides Rafinesque. Journal of the Elisha Mitchell Scientific Society, 70:139–205.

Falahatkar, B., Akhavan, S., Tolouei Gilani, M., and Abbasalizadeh, A. (2013). Sex identificattion and sexual maturity stages in farmed great sturgeon, Huso huso L. through biopsy. Iranian Journal of Veterinary Research, 2:133–139.

Faria, C., Alamada, V. C., and Goncalves, E. J. (1996). Juvenile recruitment, growth and maturation of Lipophrys pholis (Pisces: Blenniidae), from the west coast of Portugal. Journal of Fish Biology, 49:727–730.

Faria, C., Gil, F., Santos, R. S., and Almada, V. C. (2010). A comparison between the ontogeny of two related blenniid species Parablennius gattorugine and Parablennius ruber (Pisces: Blenniidae). J Mar Bio Assoc UK, 90:1263–1268.

Farwell, M. K., Green, J. M., and Pepper, V. A. (1976). Distribution and known life history of Stichaeus punctatus in the Northwest Atlantic. Copeia, 1976:598.

Faunce, C. H., Patterson, H. M., and Lorenz, J. J. (2002). Age, growth, and mortality of the Mayan cichlid (Cichlasoma urophthalmus) from the southeastern Everglades. Fish. Bull., 100:42–50.

Fava, J. A. and fa Tsai, C. (1974). The life history of the pearl dace, Semotilus margarita, in Maryland. Chesapeake Science, 15(3):159–162.

Fechhelm, R. G., Fitzgerald, P. S., Bryan, J. D., and Gallaway, B. J. (1993). Effect of salinity and temperature on the growth of yearling Arctic cisco (Coregonus autumnalis) of the Alaskan Beaufort Sea. Journal of Fish Biology, 43:463–474.

Fee, E. (1965). Life history of the northern common shiner, Notropis cornutus frontalis, in Boone County, lowa. Proceedings of the Iowa Academy of Science, 72(1):Article 41.

Felin, F. E. (1951). Growth characteristics of the poeciliid fish Platypoecilus maculatus. Copeia, 1951(1):15–28.

Fernandez-Delgado, C., Hernando, J. A., Herreraa, M., and Bellido, M. (1988). Age, growth and reproduction of Aphanius iberus (Cuv. & Vaal., 1846) in the lower reaches of the Guadalquivir rivir (south-west Spain). Freshwater Biology, 20:227–234.

Fernández-Delgado, C. and Herrera, M. . (1995). Age structure, growth and reproduction of Leuciscus pyrenaicus in an intermittent stream in the Guadalquivir river basin, southern Spain. Journal of Fish Biology, 46:371–380.

Fey, D. P. and Węsłlawski, J. M. (2017). Age, growth rate, and otolith growth of polar cod (Boreogadus saida) in two fjords of Svalbard, Kongsfjorden and Rijpfjorden. Oceanologia, 59:576–584.

Finn, R. N., Fyhn, H. J., and Evjen, M. S. (1995a). Physiological energetics of developing embryos and yolk-sac larvae of Atlantic cod (Gadus morhua). i. Respiration and nitrogen metabolism. Marine Biology, 124:355–369.

Finn, R. N., Henderson, J., and Fyhn, H. J. (1995b). Physiological energetics of developing embryos and yolk-sac larvae of Atlantic cod (Gadus morhua). i1. Lipid metabolism and enthalpy balance. Marine biology, 124:371–379.

Firdaus, M., Soemarno, S., Bintoroc, G., and dan Tri Djoko Lelonod (2017). Growth and age structure of nomei (Harpadon nehereus, Ham. 1822) in Juata Laut waters of Tarakan Island, North Borneo, Indonesian. International Journal of Sciences: Basic and Applied Research (IJSBAR), 31.

Fisher, W. L. (1990). Life history and ecology of the orangefin darter Etheostoma bellum (Pisces: Percidae). The American Midland Naturalist, 123:268–281.

fisheries organization, I. (2017). Annual performance report. Technical report.

Fleming, I. A. (1996). Reproductive strategies of Atlantic salmon: ecology and evolution. Reviews in Fish Biology and Fisheries, 6:Tables 2, 3 and 5.

Flinn, S., Midway, S., and Ostrowski, A. (2019). Age and growth of hardhead catfish and gafftopsail catfish in coastal Louisiana, USA. Marine and Coastal Fisheries: Dynamics, Management, and Ecosystem Science, 11(1):362–371.

Flower, S. (1935). Further notes on the duration of life in animals. - I. Fishes: as determined by otolith and scale - readings and direct observations on living animals. Proceedings of the Zoological Society of London, 265:page 274.

Flynn, R. (1975). The life history of the teardrop darter, Etheostoma barbouri Kuehne and Small, in Kentucky. PhD thesis, Western Kentucky Univ.

Foch, R. C. (2018). Weathering the storm: the implications of wave exposure on the distribution, phenotype and growth of a temperate reef fish. PhD thesis, Victoria University of Wellington.

Fonds, M. (1973). Sand gobies in the Dutch Wadden Sea (Pomatoschistus, Gobiidae, Pisces). Netherlands Journal of Sea Research, 6:417–478.

Fonds, M. (1979). Laboratory observations on the influence of temperature and salinity on the development of the eggs and growth of the larvae of *Solea solea* (Pisces). Marine Ecology - Progress Series, 1:91–99.

Fonds, M., Cronie, R., Vethaak, A. D., and Puyl, P. v. d. (1992). Metabolism, food consumption and growth of plaice (Pleuronectes platessa) and flounder (Platichthys flesus) in relation to fish size and temperature. Neth. J. Sea Res., 29:127–143.

Fonds, M., Drinkwaard, B., Resink, J. W., Eysink, G. G. J., and Toet, W. (1989). Measurements of metabolism, food intake and growth of *Solea solea* (L.) fed with mussel meat or with dry food. Aquaculture: a biotechnology in progress, 2:1851–874.

Fonds, M. and Saksena, V. P. (1977). The daily food intake of young soles (*Solea solea*, L.) in relation to their size and the water temperature. 3rd meeting of the ICES working group on Mariculture, Brest (France), 10-13 Mai 1977 - Actes de Colloques du C.N.E.X.O., 4.

Forbes, E. L., Preston, C. D., and Lokman, P. M. (2010). Zebrafish (Danio rerio) and the egg size versus egg number trade off: effects of ration size on fecundity are not mediated by orthologues of the Fec gene. Reprod. Fert. Dev., 22:1015–1021.

Forrester, C. R. (1964). Rate of development of eggs of rock sole (Lepidopsetta bilineata Ayres). Journal of the Fisheries Research Board of Canada, 21:1533–1534.

Forsgren, K. L. and Lowe, C. G. (2006). The life history of weedy seadragons, Phyllopteryx taeniolatus (Teleostei: Syngnathidae). Marine and Freshwater Research, 67:313–322.

Foster, N. R. (1967). Comparative studies on the biology of killifishes (Pisces: Cyprinodontidae). PhD thesis, Cornell University, Ithaca, N.Y. 369 pp.

Foster, N. R., Cairns, J., and Kaesler, R. L. (1969). The flagfish, Jordanella floridae, as a laboratory animal for behavioral bioassay studies. Proceedings of the Academy of Natural Sciences of Philadelphia, 121:129–152.

Fraker, M. E., Snodgrass, J. W., and Morgan, F. (2002). Differences in growth and maturation of blacknose dace (Rhinichthys atratulus) across an urban-rural gradient. Copeia, 2002:1122–1127.

Francis, M. (2001). Coastal fishes of New Zealand: an identification guide, 3rd edn. Reed Books, Auckland. Reed Books.

Francis, M., Griggs, L., and Maolagáin, C. O. (2004). Growth rate, age at maturity, longevity and natural mortality rate of moonfish (Lampris guttatus). Technical report, National Institute of Water and Atmospheric Research Limited (NIWA).

Franks, J. S., Ogle, J. T., Hendon, J. R., Barnes, D. N., and Nicholson, L. C. (2001). Growth of captive juvenile tripletail Lobotes surinamensis. Gulf and Caribbean Research, 13:67–70.

Frantz, T. C. and Cordone, A. J. (1965). Introduction of the bonneville cisco (Prosopium gemmifer Snyder) into Lake Tahoe, California and Nevada. Calif. Fish and Game, 51(4):270–275.

Freitas, V. (2010). own data.

Frenkel, V. and Goren, M. (2000). Factors affecting growth of killifish, Aphanius dispar, a potential biological control of mosquitoes. Aquaculture, 184:255–265.

Fricke, R. (1994). Tripterygiid Fishes of Australia, New Zealand and the Southwest Pacific Ocean: With Descriptions of 2 New Genera and 16 New Species (Teleostei), volume 24. Lubrecht & Cramer Limited.

Frimodt, C. (1995). Multilingual illustrated guide to the worlds commercial coldwater fish. Wiley-Blackwell, Berlin.

From, J. and Rasmussen, G. (1991). Growth of rainbow trout, Oncorhynchus mykiss (Walbaum, 1792) related to egg size and temperature.

Frost, W. E. (1943). The natural history of the minnow, Phoxinus phoxinus. Journal of Animal Ecology, 12.2:139–162.

Frost, W. E. and Kipling, C. (1980). The growth of charr, Salvelinus willughbii Gunther, in Windermere. J. Fish Biol., 16:279–289.

Fuchs, E. H. (1967). Life history of the emerald shiner, Notropis atherinoides, in Lewis and Clark Lake, South Dakota. Transactions of the American Fisheries Society, 96(3):247–256.

Fukuhara, O. (1983). Development and growth of laboratory reared Engraulis japonica (Houttuyn) larvae. J. Fish Biol., 23:641–652.

Fukuhara, O. and Takao, K. (1988). Growth and larval behaviour of Engraulis japonica in captivity. J. Appl. Ichthyol., 4:158–167.

Furness, A. I. and Reznick, D. N. (2014). The comparative ecology of a killifish (Rivulus hartii) across aquatic communities differing in predation intensity. Evolutionary Ecology Research, 16:249–265.

Furukawa, S., Ohshimo, S., Tomoe, S., Shiraishi, T., Nakatsukaa, N., and Kawabe, R. (2012). Age, growth, and reproductive characteristics of dolphinfish Coryphaena hippurus in the waters off west Kyushu, northern East China Sea. Fish Sci, 78:1153–1162.

Gaas, v. d. (1977). Technical report, NIOZ.

Gaetani, D. d. (1935). Sviluppo embrionale e stadi post-embrionali negli sparidi, 3, Pagellus acarne. Archivio di Oceanografia e Limnologia, 2:47–66.

Gale, W. F. (1983). Fecundity and spawning frequency of caged bluntnose minnows fractional spawners. Transactions of the American Fisheries Society, 112(3):398–402.

Galois, R., Lagardre, F., and Richard, P. (1990). Changes in biochemical composition and otolith microstructure of larval common soleas, *Solea solea* (L.) under experimental starvation. La mer, 28:273–285.

Gandhi, V. (1998). Studies on the ecology and biology of butterfish Scatophagus argus in Mandapam coastal region. PhD thesis, Madurai Kamaraj University, Madurai.

Garbina, T., Castello, J. P., and Kinas, P. G. (2014). Age, growth, and mortality of the mullet Mugil liza in Brazil's southern and southeastern coastal regions. Fisheries Research, 149:61–68.

Garcia, A. M. (2014). Invasive species of the Pacific Northwest: Northern pikeminnow, Ptychocheilus oregonensis. Technical report, U.S. Fish and Wildlife Service.

Garc'ia, C. B., Troncoso, W., Sánchez, S., and Perdomo, L. (2008). Contribution to vital statistics of a guppy Poecilia reticulata Peters (Pisces: Cyprinodontiformes: Poecillidae) pond population in Santa Marta, Colombia. Pan-American Journal of Aquatic Sciences, 3:335–339.

García-Lizárraga, M. A., Soto-Franco, F. E., de Jesús, J. M., Velazco-Arce, R., Velázquez-Abunader, J. I., Ramírez-Pérez, J. S., and na Messina, E. P. (2011). Population structure and reproductive behavior of Sinaloa cichlid Cichlasoma beani (Jordan, 1889) in a tropical reservoir. Neotropical Ichthyology, 9(3):593–599.

García-Mederos, A. M., Tuset, V. M., Santana, J. I., and González, J. A. (2010). Reproduction, growth and feeding habits of stout beardfish Polymixia nobilis (Polymixiidae) off the Canary Islands (NE Atlantic). J. Appl. Ichthyol., 26:872–880.

Gargeda, M. V. C., Sakakura, Y., and Hagiwara, A. (2004). Early development of the self-fertilizing mangrove killifish Rivulus marmoratus reared in the laboratory. Ichthyol Res, 51:309–315.

Garratt, P. A. (1993). Spawning of riverbream, Acanthopagrus berda, in Kosi Estuary. South African Journal of Zoology, 28(1):26–31.

Garrido, S., Saiz, E., Peters, J., Ré, P., Alvarez, P., Cotano, U., Herrero, D. L., de Murguía, A. M., and Irigoìen, X. (2012). Effect of food type and concentration on growth and fatty acid composition of early larvae of the anchovy (Engraulis encrasicolus) reared under laboratory conditions. Journal of Experimental Marine Biology and Ecology, 434-435(0):16–24.

Gartner, J. (1991). Life histories of three species of lanternfishes (Pisces: Myctophidae) from the eastern Gulf of Mexico II. Age and growth patterns. Marine Biology, 111.

Gartner, J. (1993). Patterns of reproduction in the dominant lanternfish species (Pisces: Myctophidae) of the Eastern Gulf of Mexico, with a review of reproduction among tropical-subtropical Myctophidae. Bulletin of Marine Science, 52.

Gash, S. L. and Bass, J. C. (1973). Age, growth and population structures of fishes from acid and alkaline strip-mine lakes in Southeast Kansas. Technical report.

Geary, R. E. and Moyle, P. B. (1980). Aspects of the ecology of the hitch, Lavinia exilicauda (Cyprinidae), a persistent native cyprinid in Clear Lake, California. The Southwestern Naturalist, 25:385–390.

Gebhard, A. E. and Perkin, J. S. (2017). Assessing riverscape-scale variation in fish life history using banded sculpin (Cottus carolinae). Environ Biol Fish, 100:1397–1410.

Geffen, A. J. and Nash, R. D. M. (1992). The life-history strategy of deepwater sculpin, Myoxocephalus thompsonii (Girard), in Lake Michigan: dispersal and settlement patterns during the first year of life. J. Fish Biol., 41 (suppl B):101–110.

Geffroy, B. and Simon, O. (2013). Effects of a Spirulina platensis-based diet on zebrafish female reproductive performance and larval survival rate. Cybium, 37:31–38.

George, S. G., Slack, W. T., and Douglas, N. H. (1996). Demography, habitat, reproduction, and sexual dimorphism of the crystal darter, Crystallaria asprella (Jordan), from south-central Arkansas. Copeia, 1996(2):68–78.

George, S. G., Slack, W. T., and Hoover, J. J. (2012). A note on the fecundity of pallid sturgeon. Journal of Applied Ichthyology, pages 1–4.

Gerhard, G. S., Kauffman, E. J., Wang, X., Stewart, R., Moore, J. L., Kasales, C. J., Demidenko, E., and Cheng, K. C. (2002). Life spans and senescent phenotypes in two strains of zebrafish (Danio rerio). Experimental Gerontology, 37(8-9):1055–1068.

G.H., E. and M., H. (2004). Maturity changes in Norwegian spring-spawning herring before, during and after a major population collapse. Fishseries Research, 66:299–310.

Ghasemi, N., F., N., Imani, A., and Shahrooz, R. (2018). Ontogeny of proteolytic enzymes (pepsin, trypsin and chymotrypsin) of stellate Acipenser stellatus from hatching up to day 50 post hatch. Iranian Scientific Fisheries Journal, 1:107–118.

Ghoneum, M. M. H. and Egami, N. (1982). Age related changes in morphology of the thymus of the fish, Oryzias latipes. Experimental Gerontology, 17:33–40.

Ghosh, S. (2014). Fishery, reproductive biology and diet characteristics of bombay duck Harpadon nehereus from the Saurashtra coast. Indian Journal of Marine Sciences, 43.

Giacoletti, A. (2016). Field measurement from Palermo coast line.

Gibson, J. R. and Fries, J. N. (2005). Culture studies of the devils river minnow. North American Journal of Aquaculture, 67:294–303.

Gibson, R. N. and Ezzi, I. A. (1980). The biology of the scaldfish, Arnoglossus laterna (Walbaum) on the west coast of Scotland. J. Fish Biol., 17.

Gill, H. S. and Weatherley, A. H. (1984). Protein, lipid and caloric contents of bluntnose minnow, Pimephales notatus Rafinesque, during growth at different temperatures. J. Fish. Biol., 25:491–500.

Gillanders, B. M. (1995). Reproductive biology of the protogynous hermaphrodite Achoerodus viridis (Labridae) from south-eastern Australia. Marine and Freshwater Research, 46:999–1008.

Gjösæter, J. (1973). Age, growth, and mortality of the mygtophid fish, Benthosema glaciale (Reinhardt), from Western Norway. Sarsia, 52.

Gjøsæter, J. (1981). Life history and ecology of the myctophid fish Notoscopelus elongatus kroeyeri from the Northeast Atlantic. FiskDir. Skr. Ser. HavUnders., 17.

Gjøsæter, J. and Tilseth, S. (1988). Spawning behaviour, egg and larval development of the myctophid fish Benthosema pterotum. Mar. Biol., 98.

Glamuzina, B., Skaramuca, B., Glavic, N., Kozvul, V., Dulcic, J., and Kraljevic, M. (1998). Egg and early larval development of laboratory reared dusky grouper, Epinephelus marginatus (Lowe, 1834) (Picies, Serranidae). Scientia Marina, 62(4):373–378.

Glazenburg, B. (1983). Een experimenteel onderzoek naar de groei van 0-groep schol en bot in relatie tot de watertemperatuur. Technical report, NIOZ, Texel. unpublished data, internal report.

Glynn, P. W., Enochs, I. C., McCosker, J. E., and Graefe, A. N. (2008). First record of a pearlfish, Carapus mourlani, inhabiting the Aplysiid Opisthobranch mollusc Dolabella auricularia. Pacific Science, 62:593–601.

Gomiero, L. M., Carmassi, A. L., and de Souza Braga1, F. M. (2007). Crescimento e mortalidade de Brycon opalinus (Characiformes, Characidae) no Parque Estadual da Serra do Mar, Mata Atlântica, Estado de são Paulo. Biota Neotropica, 7:bn00207012007.

Gonzáalez, R. A. (2006). Age and growth of the Argentine sandperch Pseudopercis semifasciata (Cuvier, 1829) Berg, 1899 in the San Matí?as Gulf (Patagonia, Argentina). Fisheries Research, 79:120–128.

Gonzales-Doncel, M., Carbonell, G., Garcia-Maurino, J. E., Sastre, S., Beltran, E. M., and Torija, C. F. (2016). Effects of dietary 2,2', 4,4'-tetrabromodiphenyl ether (bde-47) exposure in growing medaka fish(Oryzias latipes). Aquatic Toxicology, 178:141–152.

Goodchild, S. C. (2016). Life history and interspecific co-persistence of native imperiled fishes in single species and multi-species ex situ refuges. PhD thesis, North Dakota State Univ.

Goodson, M. S., Giske, J., and Rosland, R. (1995). Growth and ovarian development of Maurolicus muelleri during spring. Marine Biology, 124.

Gordoa, A. and Molí, B. (1997). Age and growth of the sparids Diplodus vulgaris, D. sargus and D. annularis in adult populations and the differences in their juvenile growth patterns in the north-western Mediterranean Sea. Fisheries Research, 33:123–129.

Gordoa, A., Molí, B., and Raventós, N. (2000). Growth performance of four wrasse species on the north-western Mediterranean coast. Fisheries Research, 45:43–50.

Gosmann, M. A. and de Oliveira Nuner, A. P. (2015). Egg incubation and larval rearing of piava, Leporinus obtusidens: Effect of ph. Bol. Inst. Pesca , Sao Paulo, 41:319–326.

G"othel, H. (1992). Fauna marina del Mediterráneo. Ediciones Omega, S.A., Barcelona.

Goto, A. (1989). Growth patterns, and age and size at maturity in female Cottus hangiongensis, with special reference to their life-history variation. Japanese Journal of Ichthyology, 36(1):90–99.

Gould, A. L., Dougan, K. E., Koenigbauer, S. T., and Dunlap, P. V. (2016). Life history of the symbiotically luminous cardinalfish Siphamia tubifer (Perciformes: Apogonidae). Journal of Fish Biology, 89:1359–1377.

Gould, W. (1985). Aspects of the biology of the flathead chub (Hybopsis gracilis) in Montana. 45(2):332–336.

Grabowski, T. B., Isely, J. J., and Weller, R. R. (2004). Age and growth of flathead catfish, Pylodictus olivaris Rafinesque, in the Altamaha River System, Georgia. Journal of Freshwater Ecology, 19(3):411–418.

Grabowski, T. B., Young, S. P., Isely, J. J., and Ely, P. C. (2012). Age, growth, and reproductive biology of three Catostomids from the Apalachicola River, Florida. Journal of Fish and Wildlife Management, 3(2):223–237.

Grady, J. M. and Bart, H. L. (1984). Life history of Etheostoma caeruleum (Pisces: Percidae) in Bayou Sara, Louisiana and Mississippi. In Lindquist, D. G. and Page, L. M., editors, Environmental biology of darters, pages 71–81. Dr W. Junk Publishers, The Hague.

Graham, K., Irwin, E. R., Hubert, W. A., and Rabeni, C. F. (1999). A review of the biology and management of blue catfish. In Catfish 2000: proceedings of the international ictalurid symposium, volume 24, pages 37–49.

Grandcourt, E. M., Abdessalaam, T. Z. A., and Francis, F. (2006). Age, growth, mortality and reproduction of the blackspot snapper, Lutjanus fulviflamma (Forsskål, 1775), in the southern Arabian Gulf. Fisheries Research, 78:203–210.

Grant, S. and Tonn, W. (2002). Effects of nutrient enrichment on recruitment of age-0 fathead minnows (pimephales promelas): potential impacts of environmental change on the boreal plains. Canadian Journal of Fisheries and Aquatic Sciences, 59:759–767.

Gray, C. A. and Barnes, L. M. (2008). Reproduction and growth of dusky flathead (Platycephalus fuscus) in NSW estuaries. Technical report, NSW Department of Primary Industries.

Gray, R. H. and Dauble, D. D. (1979). Biology of the sandroller in the Central Columbia River. Transactions of the American Fisheries Society, 108:645–649.

Grebel, J. M. and Cailliet, G. M. (2010). Age, growth, and maturity of cabezon (Scorpaenichthys marmoratus) in California. California Fish and Game, 96(1):36–52.

Greely, T. M., Gartner, J. V., and Torres, J. J. (1999). Age and growth of Electrona antarctica (Pisces: Myctophidae), the dominant mesopelagic fish of the Southern Ocean. Marine Biology, 133.

Greenbank, J. (1966). Life history of the sculpin (Cottus aleuuticus Gilbert, in Karluke Lake, Alaska. Technical report, United States Departntent of the Interior Fish and Wildlife Service Bureau of Commercial Fisheries Biological Laboratory, Auke Bay, Alaska.

Greenfield, D. W., Ross, S. T., and Deckert, G. D. (1970). Some aspects of the life history of the Santa Ana sucker, Catostomus (Pantosteus) santaanae (Snyder). Calif. Fish and Game, 56(3):166–179.

Griffiths, W. B., Gallaway, B. J., Gazey, W. J., and Dillinger, R. E. (1992). Growth and condition of Arctic cisco and broad whitefish as indicators of causeway-induced effects in the Prudhoe Bay Region, Alaska. Transactions of the American Fisheries Society, 121(5):557–577.

Gruber, K. and Wieser, W. (1983). Energetics of development of the alpine charr, Salvelinus alpinus, in relation to temperature and oxygen. J. Comp. Physiol., 149:485–493.

Guan, R., Wang, X., and Ke, G. (1994). Glass eels (Anguilla anguilla) growth in a recirculating system. Journal of Fish Biology, 45:653–660.

Güçlü, S. S. and Küçük, F. (2008). Population age, sex structure, growth and diet of Aphanius mento Heckel in: Russegger, 1843 (Cyprinodontidae: Teleostei), at Kırkgöz Spring, Antalya-Türkiye. Turkish Journal of Fisheries and Aquatic Sciences, 8:269–274.

Güçlü, S. S. and Küçük, F. (2011). Reproductive biology of Aphanius mento (Heckel in: Russegger, 1843) (Osteichthyes: Cyprinodontidae) in Kırkgöz Spring (Antalya-Turkey). Turkish Journal of Fisheries and Aquatic Sciences, 11:323–327.

Güçlü, S. S., Turna, I. I., Güçlü, Z., and Gülle, I. (2007). Population structure and growth of Aphanius anatoliae sureyanus Neu, 1937 (Osteichthyes: Cyprinodontidae), endemic to Burdur Lake, Turkey. Zoology in the Middle East, 41(1):63–69.

Güçü, S. S. and Ömer Erdoğan (2017). Age, growth, sex ratio and feeding of Knipowitschia caucasica (Berg, 1916) (Actinopterygii, Gobiidae) in non-native species of Eğirdir Lake (Turkey). Acta Biologica Turcica, 30:1–6.

Guerreiro, A., Amadio, S., Fabré, N., and Batista, V. (2018). Estimation of growth parameters of five fish species (Actinopterygii) caught in the Central Amazon. Acta Ichthyologica et Piscatoria, 48:303–313.

Gunna, J. S., Clear, N. P., Carter, T. I., Rees, A. J., Stanley, C. A., Farley, J. H., and Kalish, J. M. (2008). Age and growth in southern bluefin tuna, Thunnus maccoyii (Castelnau): Direct estimation from otoliths, scales and vertebrae. Fisheries Research, 92:207–220.

Gupta, S. (2016). An overview on morphology, biology, and culture of spotted scat Scatophagus argus (Linnaeus 1766). Reviews in Fisheries Science & Aquaculture, 24:203–212.

H., S. (2004). The reproduction biology of chub (Leuciscus cephalus L. 1758) in Topcam Dam Lake (Aydın, Turkey). Turk. J. Vet. Anim. Sci., 28:693–699.

H. Pérez-Espana, F. G.-M. and Abitia-Cárdenas, L. A. (1998). Growth, consumption, and productivity of the California killifish in Ojo de Liebre Lagoon, Mexico. Journal of Fish Biology, 52:1068–1077.

H. Tanaka), H. Kagawa, H. O. (2001). Production of leptocephali of japanese eel Anguilla japonica in captivity. Aquaculture, 201:51–60.

H.A. Lamtane and, H. P. and Ndaro, S. (2007). Reproductive biology of Gerres oyena (Pisces: Gerreidae) along the Bagamoyo Coast, Tanzania. Western Indian Ocean J. Mar. Sci., 6(1):29–35.

Haag, W. R., Commens-Carson, A., and Warren, M. L. (2007). Life history variation in the Yazoo shiner (Notropis rafinesquei) in three Mississippi Streams. Am. Midl. Nat., 158:306–320.

Hales, L. S. and Reitzh, E. J. (1992). Historical changes in age and growth of Atlantic croaker, Micropogonias undulatus (Perciformes: Sciaenidae). Journal of Archoeological Science, 19:13–99.

Hamamoto, S., Kumagai, S., Nosaka, K., Manabe, S., Kasuga, A., and Iwatsuki, Y. (1992). Reproductive behavior, eggs and larvae of a Lutjanid fish, Lutjanus stellatus, observed in an aquarium. Japanese Journal of Ichthyology, 30:219–228.

Hambrick, P. S. and Robison, H. W. (1979). Life history aspects of the paleback darter, Etheostoma pallididorsum (Pisces: Percidae), in the Caddo River System, Arkansas. The Southwestern Naturalist, 24(3):475–484.

Hamed, M. K., Alsop, F. J., and Laughlin, T. F. (2009). Life history traits of the Tennessee dace (Phoxinus tennesseensis) in Northeast Tennessee. Am. Midl. Nat., 160:289–299.

Hamman, R. L. (1982). Spawning and culture of humpback chub. The Progressive Fish Culturist, 44(4):213–216.

Hanks, J. H. and Belk, M. C. (2004). Threatened fishes of the world: Iotichthys phlegethontis Cope, 1874 (Cyprinidae). Environmental Biology of Fishes, 71:378.

Hardy, R. S. and Litvak, M. K. (2004). Effects of temperature on the early development, growth, and survival of shortnose sturgeon, Acipenser brevirostrum, and Atlantic sturgeon, Acipenser oxyrhynchus, yolk-sac larvae. Environmental Biology of Fishes, 70:145–154.

Harrington, R. W. (1948). The life cycle and fertility of the bridled shiner, Notropis bifrenatus (Cope). The American Midland Naturalist, 39(1):83–92.

Hasebe, M., Kanda, S., and Oka, Y. (2016). Female-specific glucose sensitivity of gnrh1 neurons leads to sexually dimorphic inhibition of reproduction in medaka. Endocrinology, 157(11):4318 – 4329.

Haug, T. and Gulliksen, B. (1988). Fecundity and oocyte sizes in ovaries of female atlantic halibut, Hippoglossus hippoglossus (l.). Sarsia, 73('):259–261.

Haugen, G. N. (1969). Life history, habitat and distribution of the lake sturgeon Acipenser fulvescens in the South Saskatchewan River, Alberta. PhD thesis, Montana state University.

Hauser, W. J. (1968). Life history of the mountain sucker (Catostomus platyrhynchus) in Montana. PhD thesis, Montana State Univ.

Hawk, H. A. and Allen, L. G. (2014). Age and growth of the giant sea bass, stereolepis gigas. CalCOFI Rep., 55:43.

Hayashi, A., Watanabe, H., Ishida, M., and Kawaguchi, K. (2001). Growth of Myctophum asperum (Pisces: Myctophidae) in the Kuroshio and transitional waters. Fisheries Science, 67.

He, J. X., Rudstam, L. G., Forney, J. L., Vandevalk, A. J., and Steward, D. J. (2005). Long-term patterns in growth of Oneida Lake walleye: a multivariate and stage-explicit approach for applying the von Bertalanffy growth function. Journal of Fish Biology, 66:1459–1470.

Head, M. A., Keller, A. A., and Bradburn, M. (2014). Maturity and growth of sablefish, Anoplopoma fimbria, along the U.S. West Coast. Fisheries Research, 159:56–67.

Heemstra, P. C. and Randall, J. E. (1993). Groupers of the world (family Serranidae, subfamily Epinephelinae). An annotated and illustrated catalogue of the grouper, rockcod, hind, coral grouper and lyretail species known to date. FAO Species Catalogue. Vol. 16. Rome: FAO. FAO Fish. Synop., 125(16):1–382.

Heese, T. (1984). On some problems in biology of minnow, Phoxinus phoxinus (l.) (Cyprinidae) in the river Skawa. Acta Ichthyologica et Piscatoria, 14:25–42.

Heimowitz, P. and Fuller, P. (2007). http://depts.washington.edu/oldenlab/wordpress/wp-content/uploads/2013/03/Rhinogobius-brunneus_2007R.pdf.

Heins, D. and Clemmer, G. H. (1976). The reproductive biology, age and growth of the North American cyprinid, Notropis longirostris (Hay). J. Fish Biol., 8:365–379.

Heins, D. C. (1981). Life history pattern of Notropis sabinae (Pisces: Cyprinidae in the lower Sabine River dranage of Louisiana and Texas. Tulane Studies in Zoology and Botany, 22(2):67–85.

Heins, D. C. and Bresnick, G. I. (1975). The ecological life history of the cherryfin shiner, Notropis roseipinnis. Trans. Am. Fish. Soc., 3:516–523.

Hellawell, J. M. (1969). Age determination and growth of the grayling Thymallus thymallus (L.) of the River Lugg, Herefordshire. J Fish Biol, 1:373–382.

Helms, D. R. (1974). Age and growth of shovelnose sturgeon, Scaphirhynchus platorynchus (Rafinesque), in the Mississippi River. Proceedings of the Iowa Academy of Science, 81(2):73–75.

Heming, T. A. (1982). Effects of temperature on utilization of yolk by chinook salmon (Oncorhynchus tshawytscha) eggs and alevins. Canadian Journal of Fisheries and Aquatic Sciences, 39:184–190.

Hems, D. C. and Rooks, J. R. (1984). Life history of the naked sand darter, Ammocrypta beani, in southeastern Mississippi. In Linquist, D. G. and Page, L. M., editors, Environmental biology of darters, pages 714–722. Dr. W. Junk Publ., The Hague.

Henrich, S. (1988). Variation in offspring sizes of the poeciliid fish Heterandria formosa in relation to fitness. Oikos, 51:13–18.

Henshell, D. S., Sparks, D. W., Simon, T. P., and Tosick, M. J. (2006). Age structure and growth of Semotilus atromaculatus (Mitchill) in PCB-contaminated streams. Journal of Fish Biology, 68:44–62.

Hereford, M. E. (2014). Home range, spatial dynamics, and growth of Moapa dace (Moapa coriacea). PhD thesis, University of Nevada, Reno.

Herrera, M. and Fernández-Delgado, C. (1994). The age, growth and reproduction of Chondrostoma polylepis willkommi in a seasonal stream in the Guadalquivir River basin (southern Spain). Journal of Fish Biology, 44:11–22.

Herrera, M., Hernando, J. A., Fernandez-Delgado, C., and Bellido, M. (1988). Age, growth and reproduction of the barbel, Barbus sclateri (Guenther, 1868), in a first-order stream in southern Spain. J. Fish Biol., 33:371–381.

Hettler, W. F. (1984). Description of eggs, larvae, and early juveniles of the gulf menhaden Brevoortia patronus, and comparisons with Atalantic manhaden B. tyrannus, and yellowfin menhaden, B. smithi. Fishery Bulletin, 85:85–95.

Hicks, C. and Pierce, R. (2012). Bluegill sunfish production in missouri. University of Missouri Extensions - Agriculture, 47:10.

Hicks, T., Kopf, R. K., and Humphries, P. (2015). Fecundity and egg quality of dusky flathead (Platycephalus fuscus) in East Gippsland, Victoria. Technical report, Inst for land, water and society.

Hislop, J. R. G., Gallego, A., Heath, M. R., Kennedy, F. M., Reeves, S. A., and Wright, P. J. (2001). A synthesis of the early life history of the anglerfish, Lophius piscatorius (Linnaeus 1758) in northern British waters. ICES Journal of Marine Science, 58:70–86.

Hislop, J. R. G. and Hall, W. B. (1974). The fecundity of whiting Merlangius merlangus (L.) in the North Sea, the Minch and at Iceland. J. Cons. int. Explor. Mer., 36(1).

Hlophe, S. N. (2011). The feeding and growth of Tilapia rendalli in relation to its aquaculture potential. PhD thesis, University of Limpopo, South Africa.

Hogue, J. and Kilambi, R. (1975). Age and growth of bluegill, Lepomis macrochirus Rafinesque, from Lake Fort Smith, Arkansas. Journal of the Arkansas Academy of Science, 29:43–46.

Hohler, D. B. (1981). Dwarfed Population of Catostomus rimiculus (Catostomidae: Pisces) in Jenny Creek, Jackson County, Oregon. PhD thesis, Oregon State Univ.

Holder, D. S. and Powers, S. L. (2010). Life-history aspects of the rainbow shiner, Notropis chrosomus (Teleostei: Cyprinidae), in Northern Georgia. Southeastern Naturalist, 9(2):347–358.

Hong, S. (1980). A study on the age and growth of the hairtails in northern in Po Hai and Huang Hai. Journal of Fisheries of china, 4:362–370.

Hoolihan, J. P. (2006). Age and growth of indo-pacific sailfish, Istiophorus platypterus, from the Arabian Gulf. Fisheries Research, 78:218–226.

Hop, H., Tonn, W. M., and Welch, H. E. (1997). Bioenergetics of arctic cod (Boreogadus saida) at low temperatures. Canadian Journal of Fisheries and Aquatic Sciences, 54(8):1772–1784.

Horn, P. (2002). Age and growth of Patagonian toothfish (Dissostichus eleginoides) and Antarctic toothfish (D. mawsoni) in waters from the New Zealand subantarctic to the Ross Sea, Antarctica. Fisheries Research, 56:275–287.

Hosseini-Shekarabi, S. P., Valinassab, T., Bystydzieńska, Z., and Linkowski, T. (2015). Age and growth of Benthosema pterotum (Alcock, 1890) (Myctophidae) in the Oman Sea. J. Appl. Ichthyol., 31.

Houria, H. (2015). Own measurements.

House, R. and Wells, L. (1973). Age, growth, spawning season, and fecundity of the trout-perch (Percopsis omiscomaycus) in southeastern Lake Michigan. Journal of the Fisheries Research Board of Canada, 30(8):1221–1225, 10.1139/f73–193.

Hoyt, R. D. (1971). Age and growth of the silverjaw minnow, Ericymba buccata Cope, in Kentucky. The American Midland Naturalist, 86(2):257–275.

Huber, M. and Bengtson, D. A. (1999). Effects of photoperiod and temperature on the regulation of the onset of maturation in the estuarine fish Menidia beryllina (Cope) (Atherinidae). Journal of Experimental Marine Biology and Ecology, 240:285–302.

Hubold, G. and Tomo, A. P. (1989). Age and growth of Antarctic silverfish Pleuragramma antarcticum Boulenger, 1902, from the Southern Weddell Sea and Antarctic Peninsula. Polar Biol., 9:205–212.

Hughes, G. W. and Peden, A. E. (1984). Life history and status of the shorthead sculpin (Cottus confusus: Pisces, Cottidae) in Canada and the sympatric relationship to the slimy sculpin (Cottus cognatus). Canadian Journal of Zoology, 62(2):306–311.

Hulbert, J., Bonner, T. H., Fries, J. N., Garrett, G. P., and Pendergrass, D. R. (2007). Early development of the Devils River minnow, Dionda diaboli (Cyprinidae). The Southwestern Naturalist, 52(3):378–385.

Hussain, A., Shakir, H. A., Ali, S., and Qazi, J. I. (2015). Growth coefficient and fecundity of Chitala chitala (Osteoglossiformes: Notopteridae) from the River Ravi, Pakistan. The Journal of Animal & Plant Sciences, 25:401–405.

Hussy, K., Coad, J. O., Farrell, E. D., Clausen, L. W., and Clarke, M. W. (2012). Sexual dimorphism in size, age, maturation, and growth characteristics of boarfish (Capros aper) in the Northeast Atlantic. ICES Journal of Marine Science, 69:1729–1735.

Hutchings, J. A. and Jones, M. E. B. (1998). Life history variation and growth rate thresholds for maturity in Atlantic salmon, Salmo salar. Canadian Journal of Fisheries and Aquatic Sciences, 55(S1):Table A1.

Hutchings, K., Griffiths, M. H., and Field, J. G. (2006). Regional variation in the life history of the canary drum Umbrina canariensis (Sciaenidae), in South African waters. Fisheries Research, 77:312–325.

Huysentruyt, F., Moerkerke, B., Devaere, S., and Zrdiaens, D. (2009). Early development and allometric growth in the armoured catfish Corydoras aeneus (Gill, 1858). Hydrobiologia, 627:45–54.

Hwang, S.-D., Lee, T.-W., and Hwang, S.-W. (2008). Age, growth and life history of gunnel, Pholis fangi, in the Yellow Sea. Fisheries Research, 93:72–76.

Hyndes, G. A. and Potter, I. C. (1996). Comparisons between the age structures, growth and reproductive biology of two co-occurring sillaginids, Sillago robusta and S. bassensis, in temperate coastal waters of Australia. J. Fish Biol., 49:14–32.

I. Balık, H., Çubuk, R., Özkök, and Uysal, R. (2006). Some characteristics and size of carp (Cyprinus carpio l. 1758) population in the lake karamik (afyonkarahisar/turkey). Technical report, Mediterranean Fisheries Research. Antalya. Turkey.

ICES (2012). Report of the working group on acoustic and egg surveys for sardine and anchovy in ICES areas VIII and IX (WGACEGG), Fuengirola (Malaga), Spain, 26-30 November 2012. Technical report, ICES.

Ilieş, I., Sîrbulescu, R. F., and Zupanc, G. K. (2014). Indeterminate body growth and lack of gonadal decline in the brown ghost knifefish (Apteronotus leptorhynchus), an organism exhibiting negligible brain senescence. Can. J. Zool., 92:947–953.

Imani, M. and Falahatkar, B. (2017). Larval development of Persian Sturgeon (Acipenser persicus) with emphasis on determination on start feeding. Journal of Animal Researches, 3:301–311.

Imsland, A. K., Foss, A., Sparboe, L. O., and Sigurdsson, S. (2006). The effect of temperature and fish size on growth and feed efficiency ratio of juvenile spotted wolffish Anarhichas minor. J. Fish Biol., 68:1107–1122.

Ingram, A., Ibbotson, A., and Gallagher, M. (20aa). The ecology and managements of the European grayling Thymallus thymallus (Linnaeus). Technical report, Institute of freshwater ecology.

Ingram, T. (2007). Age, Growth, and Fecundity of Alabama Shad (Alosa alabamae) in the Apalachicola River, Florida. PhD thesis, Clemson University.

Inslee, T. D. (1983). Spawning and hatching of the colorado squawfish (Ptychocheilus lucius). Technical report, U.S. Fish and Wildlife Service P.O. Box 217 Dexter, New Mexico 88230.

Iqbal, K. M., Masuda, Y., Suzuki, H., and Shinomiya, A. (2006). Age and growth of the Japanese silver-biddy, Gerres equulus, in western Kyushu, Japan. Fisheries Research, 77:45–52.

Iqbal, K. M., Suzuki, H., Ohtomi, J., and Masuda, Y. (2008). Batch fecundity of the Japanese silver-biddy Gerres equulus in the Yatsushiro Sea, western Kyushu, Japan. Aquaculture Sci, 56:409–413.

Irwin, S. W. and Foltz, J. W. (2012). Reproductive characteristics of the turquoise darter (Etheostoma inscriptum) in the upper Piedmont of South Carolina. Copeia 2012, 2012(2):237–242.

Ishikawa, T. and Tachihara, K. (2010). Life history of the nonnative convict cichlid Amatitlania nigrofasciata in the Haebaru Reservoir on Okinawa-jima Island, Japan. Environ Biol Fish, 88:283–292.

Ismen, A., Arslan, M., Yigin, C. C., and Bozbay, N. A. (2013). Age, growth, reproduction and feeding of john dory, Zeus faber (Pisces: Zeidae), in the Saros Bay (North Aegean Sea). J. Appl. Ichthyol, 29:125–131.

Ismen, A. and Ismen, P. (2004). Age, growth and reproduction of tub gurnard (Chelidonichthys lucerna L. 1758). Turk J. Vet. Anim. Sci., 28:289–295.

Iwamatsu, T. and Egami, N. (2004). Stages of normal development in the medaka Oryzias latipes. Mechanisms of Development, 121:605–618.

J, B. V. (1993). Sturgeons and paddlefishes: threatened fishes in need of conservation.

J., G. A. (2002). Length of herring larvae in relation to age and time of hatching. Journal of Fish Biology, 60:479–485.

Jakov, D., Kožul, V., Kraljevic, M., Skaramuca, B., Glamuzina, B., and Ré, P. (1999). Embryonic and larval development of the brown wrasse Labrus merula (Pisces: Labridae). Journal of the Marine Biological Association of the UK, 79:327–332.

Jakupsstovu, S. and Haug, T. (1988). Growth, sexual maturation, and spawning season of atlantic halibut Hippoglossus hippoglossus, in faroese waters. Fisheries Research, 6(3):201–215.

James, P. W. and Taber, C. A. (1986). Reproductive biology and age and growth of the Yoke darter, Etheostoma juliae. Copeia, 2:536–540.

James, R. and Sampath, K. (2003). Effects of meal frequency on growth and reproduction in the ornamental red swordtail, Xiphophorus helleri. The Israeli Journal of Aquaculture - Bamidgeh, 55(3):197–207.

Jarrin, J. R. M., Andrade-Vera, S., Reyes-Ojedis, C., and de Léon, P. S. (2018). Life history of the mottled scorpionfish, Pontinus clemensi, in the Galapagos Marine Reserve. Copeia, 106(3):515–523.

Jasper, J. R. and Evenson, D. F. (2006). Length-girth, length-weight, and fecundity of Yukon River chinook salmon Oncorhynchus tshawytscha. Technical report, Alaska Department of Fish and Game. Divisions of Sport Fish and Commercial Fisheries.

Javier Tomás, J. P. (2000). Otolith microstructure examination and growth patterns of Vinciguerria nimbaria (Photichthyidae) in the tropical Atlantic Ocean. Fisheries Research, 46.

Jearald, A. and B.E., B. (1971). Fecundity, age and growth and condition of channel catfish in an Oklahoma reservoir. Proceedings of the Oklahoma Academy of Science, 51:15–22.

Jeffrey, M. L. (2007). Larval development of Achoerodus viridis (Pisces: Labridae), the Australian eastern blue groper. Ichthyological Research, 51:46–51.

Jellyman, D. J. (1980). Age, growth, and reproduction of perch, Perca fluviatilis L., in Lake Pounui. New Zealand Journal of Marine and Freshwater Research, 14(4):391–400.

Jenssen, M. T. S., Borgstrom, R., Salbu, B., and Rosseland, B. O. (2010). The importance of size and growth rate in determining mercury concentrations in European minnow (Phoxinus phoxinus) and brown trout (Salmo trutta) in the subalpine lake, øvre Heimdalsvatn. Hydrobiologia, 642:115–126.

Jester, D. B. (1973). Life history, ecology, and management of the smallmouth buffalo Ictiobus bubalus (Rafinesque) with reference to Elephant Butte Lake. Technical report.

Jiang, Y., Zhang, C., Ye, Z., and Tian, Y. (2018). Analyses of egg size, otolith shape, and growth revealed two components of small yellow croaker in Haizhou Bay spawning stock*. Journal of Oceanology and Limnology.

Jirsa, D., Drawbridge, M., and Stuart, K. (2007). Spawning of a captive population of California sheephead, Semicossyphus pulcher. Journal of the World Aquaculture Society, 38(1):122–128.

Joanna, G., Dariusz, P., Mirosław, P., Serhan, T. A., Lidia, M., and Magdalena, L.-K. (2011). Life-history traits of Amur sleeper, Perccottus glenii, in the invaded vistula river: early investment in reproduction but reduced growth rate. Hydrobiologia, 661:197–210.

Jobes, F. W. (1943). The age, growth, and bathymetric distribution of Reighard's chub, Leucichthys reighardi Koelz, in Lake Michigan. Transactions of the American Fisheries Society, 72(1):108–135.

Johannes, M. R. S., McQueen, D. J., Stewart, T. J., and Post, J. R. (1989). Golden shiner (Notemigonus crysoleucas) population abundance: Correlations with food and predators. Can. J. Fish. Aquat. Sci., 46:810–817.

Jóhannsdóttir, S. and Vilhjálmsson, H. (1999). Fecundity of Icelandic capelin Mallotus villosus (Müller). Rit Fiskideildar, 16:263–270.

Johnsom, J. D. and Hatch, J. T. (1991). Life history of the least darter Etheostoma microperca at the northwestern limits of its range. Am. Midl. Nat., 125:87–103.

Johnson, J. B., Belk, M. C., and Shiozawa, D. K. (1995). Age, growth, and reproduction of leatherside chub (Gila copei). Great Basin Naturalist, 55(2):l83–187.

Johnson, J. E. (1970). Age, growth, and population dynamics of threadfin shad, Dorosoma petenense (günther), in Central Arizona Reservoirs. Transactions of the American Fisheries Society, 99:739–753.

Johnson, J. E. (1971). Maturity and fecundity of threadfin shad, Dorosoma petenense (günther), in Central Arizona Reservoirs. Transactions of the American Fisheries Society, 100:74–85.

Johnson, R. P. (1959). Life history of the bigmouth buffalofish Ictiobus cyprinellus in Saskatchewan. PhD thesis, Dept biol. Univ. Saskatchewan.

Johnston, C. E. and Haag, W. R. (1996). Life history of the Yazoo darter (Percidae: Etheostoma raneyi), a species endemic to north-central Mississippi. Tulane Studies in Zoology and Botany, 30:47–60.

Johnston, C. E. and Knight, C. L. (1999). Life-history traits of the bluenose shiner, Pteronotropis welaka (Cypriniformes: Cyprinidae. Copiea, 1999(1):200–205.

Johnston, T. A., Lysack, W., and Leggett, W. C. (2012). Abundance, growth, and life history characteristics of sympatric walleye (Sander vitreus) and sauger (Sander canadensis) in Lake Winnipeg, Manitoba. Journal of Great Lakes Research, 38:35–46.

Jolly, D. M. and Powers, S. L. (2008). Life-history aspects of Notropis xaenocephalus (Coosa shiner) (Actinopterygii: Cyprinidae) in Northern Georgia. Southeastern Naturalist, 7(3):449–458.

Jomes, A. (1974). Sexual maturity, fecundity and growth of the turgot Scophthalmus maximum L. J. Mar. biol. Ass. U.K, 54:109–125.

Jones, A. (1972). Studies on egg development amd larval rearing of turgot, Scophthalmus maximus L., and brill, Scophthalmus rhombus L., in the laboratory. J. Mar bio. Ass. U.K., 52:965–986.

Jones, A. (1973). Observations on the growth of turbot larvae Scophthalmus maximus L. reared in the laboratory. Aquaculture, 2:149–155.

Jones, C. M. (2000). Fitting growth curves to retrospective size-at-age data. Fisheries Research, 46(3):123–129.

Jones, G. P. and McCormick, M. I. (2002). Numerical and energetic processes in the ecology of coral reef fishes. In Sale, P. F., editor, Coral reef fishes, chapter 10, pages 221–242. Academic Press.

Jones, J. W. and Hynes, H. B. N. (1950). The age and growth of Gasterosteus aculeatus, Pygosteus pungitius and Spinachia vulgaris as shown by their otoliths. J. Anim. Ecol., 19:59–73.

Jones, N. and McCarthy, I. (2013). Conservation aquaculture rearing techniques for the European smelt Osmerus eperlanus. Technical report, Seafare, Sustainable and Environmentally friendly Aquaculture for the Atlantic region of Europe.

Jones, R. and Hislop, J. R. G. (1972). Investigations into the growth of haddock, Melanogrammus aeglefinud (L.) and whiting, Merlangius merlangus (L.) in aquaria. J. Cons. int. Explor. Mer., 34.

Jordan, D. S. and Evermann, B. W. (1896-1900). The fishes of North and Middle America: a descriptive catalogue of the species of fish-like vertebrates found in the waters of North America, north of the Isthmus of Panama. Smithsonian Institution.

Joshi, K., Das, S., Khan, A., Pathak, R., and Sarkar, U. (2016). Reproductive biology of snow trout, Schizothorax richardsonii (Gray, 1832) in a tributary of river Alaknanda, India and their conservation implications. International Journal of Zoological Investigations, 2:109–114.

Joung, S.-J. and Chen, C.-T. (1992). Age and growth of the big eye Priacanthus macracanthus from the surrounding water of Guei-Shan Island, Taiwan. Nippon Suisan Gakkaishi, 58:481–488.

J.R, K. and N.A, H. (2012). The behavioural thermal preference of the common triplefin (Forsterygion lapillum) tracks aerobic scope optima at the upper thermal limit of its distribution. Journal of Thermal Biology, 37(2):118–124.

Jungwirth, M. and Winkler, H. (1984). The temperature dependence of embryonic development og grayling (Thymallus, thymallus), Danube salmon (Hucho hucho), Arctic char (Salvelinus alpinus) and brown trout (Salmo trutta fario). Aquaculture, 38:315–327.

Jusup, M., Klanjscek, T., Matsuda, H., and Kooijman., S. A. L. M. (2011). A full lifecycle bioenergetic model for bluefin tuna. Plos Biology, 6:e21903.

Jutila, E., Jokikokko, E., and Julkunen, M. (2006). Long-term changes in the smolt size and age of Atlantic salmon, Salmo salar L., in a northern Baltic river related to parr density, growth opportunity and postsmolt survival. Ecology of Freshwater Fish, 15:321–330.

Kadharsha, K., Mohanchander, P., Lyla, P. S., and Khan, S. A. (2013). Feeding and reproductive biology of Saurida undosquamis (Richardson, 1848) from Parangipettai coast, southeast coast of India. Fisheries Research, 16.

Kadison, E., D'Alessandro, E. K., Davis, G. O., and Hood, P. B. (2010). Age, growth, and reproductive patterns of the great barracuda, Sphyraena barracuda, from the Florida Keys. BULLETIN OF MARINE SCIENCE, 86:773–784.

Kadwade, P. V. (1971). Age and growth of Polydactylus indicus (Shaw). Indian Journal of fisheries, 18:165–169.

Kam, L. E. and Leung, P. (2002). Size economies of a Pacific threadfin Polydactylus sexfilis hatchery in Hawaii. Journal of the World Aquaculture Society, 33:165–169.

Kamilov, B. G. (2014). Age and growth of silver carp (Hypophthalmichthys molitrix Val.) in Tudakul Reservoir, Uzbekistan. Croatian Journal of Fisheries, 72:12–16.

Kanak, M. K. and Tachihara, K. (2006). Age and growth of Gerres oyena (forsskål, 1775) on Okinawa Island, Japan. J. Appl. Ichthyol., 22:310–313.

Kang, I. J., Yokota, H., Oshima, Y., Tsuruda, Y., Oe, T., Imada, N., Tadokoro, H., and Honjo, T. (2002). Effects of bisphenol a on the reproduction of Japanese medaka (Oryzias latipes). Environmental Toxicology and Chemistry, 21:2394–2400.

Kangombe, J. (2004). Development of feeding protocols for Tilapia rendalli in Malawi reared in semi-intensive culture systems. PhD thesis, Memorial University of Newfoundland.

Karatas, M. and Seze, M. (2005). Reproduction characteristics of populations of carp inhabiting in almus dam lake, turkey. Technical report, fao.

Karlou-Riga, C. (2000). Otolith morphology and age and growth of Trachurus mediterraneus (Steindachner) in the Eastern Mediterranean. Fisheries Research, 46:69–82.

Karr, J. R. (1963). Age, growth, and food habits of johnny, slenderhead and blacksided darters of boone county, Iowa. Proceedings of the Iowa Academy of Science, 70:228–236.

Karr, J. R. (1964). Age, growth, fecundity and food habits of fantail darters in Boone County, lowa. 71(1):274–280.

Kaya, M., Benli, H. A., Katagan, T., and Ozaydin, O. (1999). Age, growth, sex-ratio, spawning season and food of golden banded goatfish, Upeneus moluccensis Bleeker (1855) from the Mediterranean and south Aegean Sea coasts of Turkey. Fisheries Research, 41:317–328.

Keenlyne, K. D. and Jenkins, L. G. (1993). Age at sexual maturity of the pallid sturgeon. Transactions of the American Fisheries Society, 122(3):393–396.

Keenlyne, K. D. and Maxwell, S. J. (1993). Length conversions and length-weight relations for pallid sturgeon. North American Journal of Fisheries Management, 13(2):395–397.

Keeton, D. (1963). Growth of fishes in the Des Moines River, Iowa, with particular reference to water levels. PhD thesis, Iowa State University.

Keivani, Y., Nasri, M., Abbasi, K., and Abdoli, A. (2016). Atlas of inland water fishes of Iran. Iranian Department of Environment.

Kelley, S. W. (2012). Age and growth of spawning longnose gar (Lepisosteus osseus) in a north central Texas reservoir. Western North American Naturalist, 72:69–77.

Kemp, J., Jenkins, G. P., and Swearer, S. E. (2013). Assessing the intrinsic resilience of a particularly fast-growing teleost prey species (red cod, Pseudophycis bachus). Marine and Freshwater Research, 64:130–138.

Kennedy, J., Geffen, A. J., and Nash, R. D. M. (2007). Maternal influences on egg and larval characteristics of plaice (Pleuronectes platessa L.). J. Sea Res., 58:65–77.

Kennedy, J. L., Sigler, W. F., and Kucera, P. A. (1978). The reproductive ecology of the Tahoe sucker, Catostomus tahoensis, in Pyramid Lake, Nevada. Great Basin Naturalist, 38(2):Article 3.

Kennedy, M. and Fitzmaurice, P. (1974). Biology of the rudd Scardinius erythrophthalmus (L) in Irish waters. Proceedings of the Royal Irish Academy. Section B: Biological, Geological, and Chemical Science, 74:245–303.

Kennedy, S. E. (1977). Life history of the Leon Springs pupfish, Cyprinodon bovinus. Copeia, 1977(1):93–103.

Kent, D., Drost, H. E., Fisher, J., Oyama, T., and Farrell, A. P. (2016). Laboratory rearing of wild Arctic cod *Boreogadus saida* from egg to adulthood. Journal of Fish Biology, 88:1241–1248.

Kepner, W. G. (1982). Reproductive Biology of the Longfin Dace (Agosia chrysogaster) a Sonoran Desert stream, Arizona. PhD thesis, Arizona State University.

Kevrekidis, T., Kokkinakis, A. K., and Koukouras, A. (1990). Some aspects of the biology and ecology of Knipowitschia caucasica (Teleostei: Gobiidae) in the Evros Delta (North Aegean Sea). Helgoländer Meeresunters., 44:173–187.

Khudamrongsawat, J., Arrington, D. A., Kuhajda, B. R., and Rype, A. L. (2005). Life history of the endangered vermilion darter (Etheostoma chermocki) endemic to the Black Warrior River System, Alabama. Journal of Freshwater Ecology, 20:469–477.

Khudamrongsawat, J. and Kuhajda, B. R. (2007). Life history of the warrior darter (Etheostoma bellator) and comparison with the endangered vermilion darter (Etheostoma chermocki). Journal of Freshwater Ecology, 22:241–248.

Kieffer, J. D., Alsop, D., and Wood, C. M. (1998). A respirometric analysis of fuel use during aerobic swimming at different temperatures in rainbow trout (Oncorhynchus mykiss). Journal of Experimental Biology, 201(22):3123–3133.

Kilambi, R. V. (1986). Age, growth and reproductive strategy of the snakehead, Ophicephalus striatus Bloch, from Sri Lanka. J. Fish Biol., 29:13–22.

Kilic, S. and Becer, Z. (2016). Growth and reproduction of chub (Squalius cephalus) in Lake Yeniçağa, Bolu, Turkey. Int. J. Agric. Biol., 18:419–424.

Killen, S. S. (2014). Growth trajectory influences temperature preference in fish through an effect on metabolic rate. Journal of Animal Ecology, 83:1513–1522.

Kimura, S. (1995). Growth of the clupeid fishes, Stolothrissa tanganicae and Limnothrissa miodon, in the Zambian waters of Lake Tanganyika. Journal of Fish Biology, 47:569–575.

King, D. P. F., OToole, M. J., and Robertson, A. A. (1977). Early development of the South African Maasbanker Trachurus trachurus at controlled temperatures. Fisheries Bulletin of South Africa, 9:16–22.

Kingsford, M. J. (1985). The demersal eggs and planktonic larvae of Chromis dispilus (Teleostei: Pomacentridae) in north-eastern New Zealand coastal waters. New Zealand Journal of Marine and Freshwater Research, 19:429–438.

Kingsford, M. J., Finn, M. D., O'Callaghan, M. D., Atema, J., and Gerlach, G. (2014). Planktonic larval duration, age and growth of Ostorhinchus doederleini (Pisces: Apogonidae) on the southern Great Barrier Reef, Australia. Mar. Biol., 161:245–259.

Kingsford, M. J., O'Callaghan, M. D., Liggins, L., and Gerlach, G. (2017). The short-lived neon damsel Pomacentrus coelestis: implications for population dynamics. Journal of Fish Biology, 90:2041–2059.

Kinzelbach, R. (1987). Das ehemalige Vorkommen des Störs, Acipenser sturio (Linnaeus, 1758), im Enizugsgebiet des Rheins (Chondrostei: Acipenseridae). Z. Angew. Zool., 74:167–200.

Kir, M., Sunar, M., and Altindag, B. (2017). Thermal tolerance and preferred temperature range of juvenile meagre acclimated to four temperatures. Journal of Thermal Biology, 65:125–129.

Kirschbaum, F. and Schugardt, C. (2002). Reproductive strategies and developmental aspects in mormyrid and gymnotiform fishes. Journal of Physiology-Paris, 557-566:96.

Kiyashko, V. I., Osipov, V. V., and Slyn'ko, Y. V. (2006). Size-age characteristics and population structure of Clupeonella cultriventris at its naturalization in the Rybinsk Reservoir. Journal of Ichthyology, 46:64–73.

Kjesbu, O. S., Klungsoyr, J., Kryvi, H., Witthames, P. R., and Greer Walker, M. (1991). Fecundity, atresia, and egg size of captive Atlantic cod (Gadus morhua) in relation to proximate body composition. Can.J.Fish. Aquat. Sci., 48:2333–2343.

Kjesbu, O. S., Witthames, P. R., Solemdal, P., and Greer Walker, M. (1998). Temporal variations in the fecundity of Arcto-Norwegian cod (Gadus morhua) in response to natural changes in food and temperature. Journal of Sea Research, 40:303–321.

Klemetrsen, A., Amundsen, P.-A., Dempson, J. B., Jonsson, N., O"Connell, M. F., and Mortensen, E. (2003). Atlantic salmon Salmo salar L., brown trout, Salmo trutta L. and Arctic charr Salvelinus alpinus (L.): a review of aspects of their life histories. Ecology of freshwater fish, 12:1–59.

Klimogianni, A., Pagoulatou, M., Trageli, M., and Hotos, G. (2013). Investigation on early development, the feeding ability and larval survival under starvation in common meagre, Argyrosomus regius (Asso 1891). Journal of Aquatic Science, 1:1–6.

Kneib, R. T. and Stiven, A. E. (1978). Growth, reproduction, and feedisg of Fundulus heteroclitus (L.) on a North Carolina salt marsh. J. exp mar. Biol. Ecol, 52:121–140.

Knight, J. G. and Ross, S. T. (1992). Reproduction, age and growth of the Bayou darter Etheostoma rubrum (Pisces, Percidae): An endemic of Bayou Pierre. Am. Midl. Nat., 127:91–105.

Knight, J. R. (2011). Age, growth, home range, movement, and habitat selection of redeye bass (Micropterus coosae) from the middle Tallapoosa River tributaries (Alabama, USA). PhD thesis, Auburn Univ.

Koc, H., Erdogan, Z., M., T., and Treer, T. (2007). Age, growth and reproductive characteristics of chub, Leuciscus cephalus (L., 1758)in the Ikizcetepeler Dam Lake (Balikesir), Turkey. J. Appl. Ichthyol., 23:19–24.

Koeda, K., Fukagawa, T., Ishihara, T., and Tachihara, K. (2013). Reproductive biology of nocturnal reef fish Pempheris sp. (Pempherididae) in Okinawa Island, Japan. Galaxea, Journal of Coral Reef Studies, special issue:221–228.

Koeda, K., Ishihara, T., Fukagawa, T., and Tachihara, K. (2016). Life cycle differences between two species of genus Pempheris based on age determination around Okinawa-jima Island. Ichthyol Res, 63:519–528.

Koeda, K., Ishihara, T., and Tachihara, K. (2012). The reproductive biology of Pempheris schwenkii (Pempheridae) on Okinawa Island, southwestern Japan. Zoological studies, 51:1086–1093.

Koli, L. (1990). Suomen kalat. [Fishes of Finland]. Werner Söderström Osakeyhtiö., Helsinki. 357 p.

Kolpakov, E. V. (2011). Biology of Volk's sculpin Cottus volki (Cottidae) from the Serebryanka River (Central Primor'e). Journal of Ichthyology, 51:178–186.

Kolpakov, E. V. and Deminab, E. I. (2011). Biology, abundance, and life cycle of big-head Far East goby Gymnogobius urotaenia (Gobiidae) from the Serebryanka River (Central Primor'e). Journal of Ichthyology, 51:73–83.

Kolpakov, E. V. and Milovankin, P. G. (2014). Size-age structure, growth, and feeding of stone cockscomb Alectrias alectrolophus (Pisces: Stichaeidae) from Olga Bay of the Sea of Japan. Journal of Ichthyology, 54:372–376.

Kolpakov, E. V. and Miroshnik, V. V. (2007). Stone cockscomb Alectrias alectrolophus (Stichaedae) – a new representative of cold-water ichthyofauna in the waters of northern Primorskii Krai. Journal of Ichthyology, 47:194–197.

Kolpakov, N. V. (2006). On the biology of rockfishes Sebastes minor and S. taczanowskii (Sebastidae) from the coastal waters of Northern Primorye. Journal of Ichthyology, 46:311–321.

Kolpakov, N. V., Kim, L. N., and Milovankin, P. G. (2014). Landlocked South Asian Dolly Varden char Salvelinus curilus (Salmonidae) from the Basin of Petrovskoe Reservoir (Petrovka River, Primorye). Journal of Ichthyology, 54:428–432.

Kondo, M., Maeda, K., Hirashima, K., and Tachihara, K. (2013). Comparative larval development of three amphidromous Rhinogobius species, making reference to their habitat preferences and migration biology. Marine and Freshwater Research, 64:249–266.

Konecna, M. (2012). Reproduction mode of European bitterling (Rhodeus amarus, Bloch, 1782) determined through rapid oocyte counts and size determination using digital imaging. J. Appl. Ichthyol., 28:806–810.

Kopec, J. A. (1949). Ecology, breeding habits and young stages of Crenichthys baileyi, a Cyprinodont fish of Nevada. Copeia, 1949(1):56–61.

Korlyakov, K. A. and Mukhachev, I. S. (2009). On the European smelt Osmerus eperlanus introduced to Bolshoi Kisegach Lake in the South Urals. Journal of Ichthyology, 49:668–673.

Korzelecka-Orkisz, A., Szalast, Z., Pawlos, D., Smaruj, I., nski, A. T., Szulc, J., and Formicki, K. (2012). Early ontogenesis of the angelfish, Pterophyllum scalare Schultze, 1823 (Cichlidae). Neotropical Ichthyology, 10(3):567–576.

Koshelev, V. N., Mikheev, P. B., and Shmigirilov, A. P. (2014). Age and growth of kaluga Acipenser dauricus from the estuary of the Amur and its lagoon. Journal of Ichthyology, 54:165–176.

Kottelat, M. and Freyhof, J. (2007). Handbook of European Freshwater Fishes. Maurice Kottelat (privately published), Berlin.

Koutrakis, E. T. and Sinis, A. I. (1994). Growth analysis of grey mullets (Pisces, Mugilidae) as related ?? age and site. Israel J. Zool., 40:37–53.

Kováč, V. (1995). Reproductive behaviour and early development of the European mudminnow Umbra krameri. Folia Zoologica, 44:57–80.

Kozowski, K., Kozowski, J., nski, P. P., and Martyniak, A. (2010). Age and growth of vendace, Coregonus albula (L.), from Lake Wigry (northeast Poland). Arch. Pol. Fish., 18:239–245.

Kraljevic, M. and Dulcic, J. (1997). Age and growth of gilt-head sea bream (Sparus aurata L.) in the Mirna Estuary, Northern Adriatic. Fisheries Research, 31:249–255.

Kruesi, K., Rosenthal, G. G., and Alcaraz, G. (2011). Growth and male ornamentation in Xiphophorus montezumae. Marine and Freshwater Behaviour and Physiology, iFirst:1–11.

Krüger, F. (1965). Zur Mathematik des tierischen Wachstums I. Grundlagen einer neuen Wachstumsfunktion. Helgoländer wiss. Meeresunters, 49:78–136.

Krüger, F. (1969). Das asymptotische Wachstum der Fische - ein nonsens? Helgoländer wiss. Meeresunters., 19:1123–1138.

Kucera, P. A. (1978). Reproductive biology of the tui chub, Gila bicolor, in Pyramid Lake, Nevada. The Great Basin Naturalist, 38(2):203–207.

Kudo, T. and Mizuguchi, K. (2000). Growth of large and small forms of pond smelt Hypomesus nipponensis in Lake Kasumigaura, Japan. Threatened Irish Freshwater Fishes, 104B(3):57–66.

Kuhajda, B. and Rider, S. (2016). Status of the imperiled Alabama sturgeon (Scaphirhynchus suttkusi Williams and Clemmer, 1991). J Appl Ichthyol, 32:15–29.

Kunz, K. L., Frickenhaus, S., Hardenberg, S., Torild, J., Leo, E., Pörtner, H.-O., Schmidt, M., Windisch, H. S., Knust, R., and Mark, F. C. (2016). Individual whole-animal parameters of polar cod (Boreogadus saida) and atlantic cod (Gadus morhua) acclimated to ocean acidification and warming conditions. In Kunz, K. L. e. a., editor, New encounters in Arctic waters: a comparison of metabolism and performance of polar cod (Boreogadus saida) and Atlantic cod (Gadus morhua) under ocean acidification and warming. PANGAEA. In supplement to: Polar Biology, 39(6), 1137-1153; 10.1594/PANGAEA.867390.

Kupren, K., Mamcarz, D., and D., K. (2011). Effect of variable and constant thermal conditions on embryonic and early larval development of fish from the genus Leuciscus (Cyprinidae, Teleostei). Czech J. Anim. Sci., 56(2):70–80.

Kupren, K., Trabska, I., Zarski, D., Krejszeff, S., Palinska-Zarska, K., and Kucharczyk, D. (2014). Early development and allometric growth patterns in burbot lota lota l. Aquacult Int, 22.

Kurita, Y., Sano, M., and Shimizu, M. (1991). Age and growth of the hexagrammid fish Hexagrammos agrammus at Aburatsubo, Japan. Nippon Suisan Gakkaishi, 57(7):1293–1299.

Kuznetsov, V. A. (2010). Growth, size-age structure of catches and reproduction of the Volga zander Sander volgensis (Percidae) in the upper part of the Volga stretch of the Kuibyshev Reservoir. Journal of Ichthyology, 50:772–777.

Kwok, K. Y. and Ni, I.-H. (1999). Reproduction of cutlassfishes Trichiurus spp. from the South China Sea. Mar. Ecol. Prog. Ser., 176:39–47.

Kwok, K. Y. and Ni, I.-H. (2000). Age and growth of cutlassfishes, Trichiurus spp., from the South China Sea. Fish. Bull., 98:748–758.

Laak, G. A. J. d. (2008). Vlagzalm Thymallus thymallus (Linnaeus, 1758). Technical report, Sportvisserij Nederland, Postbus 162, 3720 AD Bilthoven.

Labropoulou, M. and Papaconstantinou, C. (2000). Comparison of otolith growth and somatic growth in two macrourid fishes. Fisheries Research, 46:177–188.

Lai, H. L. and Gunderson, D. R. (1987). Effects of ageing errors on estimates of growth, mortality and yield per recruit for walleye pollock (Theragra chalcogramma). Fisheries Research, 5.

Landa, J., Pereda, P., Duarte, R., and Azevedo, M. (2001). Growth of anglerfish (Lophius piscatorius and L. budegassa) in Atlantic Iberian waters. Fisheries Research, 51:363–376.

Landa, J., Pérez, N., and Pifieiro, C. (2002). Growth patterns of the four spot megrim (Lepidorhombus boscii) in the northeast Atlantic. Fisheries Research, 55:141–152.

Landa, J., Pifieiro, C., and Pérez, N. (1996). Megrim (Lepidorhombus whiffiagonis) growth patterns in the northeast Atlantic. Fisheries Research, 26:279–294.

Last, J. M. (1978). The food of four species of Pleuronectiform larvae in the eastern English Channel and southern North Sea. Marine Biology, 45.

Lauff, R. F. and Wood, C. M. (1996). Respiratory gas exchange, nitrogenous waste excretion, and fuel usage during starvation in juvenile rainbow trout, Oncorhynchus mykiss. J Comp Physiol B, 165:542–551.

Law, C. S. W. and de Mitcheson, Y. S. (2018). Age and growth of black seabream Acanthopagrus schlegelii (Sparidae) in Hong Kong and adjacent waters of the northern South China Sea. J Fish Biol., 93(1):382–390.

Lawrence, C., Ebersole, J. P., and Kesseli, R. V. (2008). Rapid growth and out-crossing promote female development in zebrafish (Danio rerio). Environmental Biology of Fishes, 81:239–246.

Layman, S. R. (1991). Life history of the relict, duskytail darter, Etheostoma (Catonotus) sp., in Little River, Tennessee. Copeia, 1991:471–485.

Lea, B. E. (1930). Mortality in the tribe of Norwegian herring. Rapports et Proces-Verbaux Des Reunions, 65:100–117.

LeBrasseur, R. J. and Parker, R. R. (1964). Growth rate of Central British Columbia pink salmon (Oncorhynchus gorbuscha). Journal of the Fisheries Research Board of Canada, 21(5):1101–1128.

Lee, B., Cockroft, K., Arkhipkin, A. I., Wing, S. R., and Randhawa, H. S. (2019). Age, growth and mortality estimates for the ridge-scaled grenadier Macrourus carinatus (Günther, 1878) in the south-western Atlantic. Fisheries Research, 218:174–185.

Lee, S., Sonmez, O., Hung, S. S., and Fadel, J. G. (2017). Development of growth rate, body lipid, moisture, and energy models for white sturgeon (Acipenser transmontanus) fed at various feeding rates. Animal Nutrition, 3:46e60.

Legendre, M., Subagja, J., and Slembrouck, J. (1998). Absence of marked seasonal variations in sexual maturity of Pangasius hypophthalmus brooders held in ponds at the Sukamandi station. In Proc mid-term workshop of the Catfish Asia Project, Cantho, Vietnam, 11-15 May 1998.

Legendre, M., Teugels, G. G., Cauty, C., and Jalabert, B. (1992). A comparative study on morphology, growth rate and reproduction of Clarias gariepinus (Burchell, 1822), Heterobranchus longifilis Valenciennes, 1840, and their reciprocal hybrids (Pisces, Clariidae). Journal of Fish Biology, 40:59–79.

Lehtinen, S. and Echelle, A. A. (1979). Reproductive cycle of Notropis boops (Pisces: Cyprinidae) in Brier Creek, Marshall County, Oklahoma. The American Midland Naturalist, 102(2):237–243.

Leijzer, T. B. (2006). Diklipharder Chelon labrosus (Risso, 1827). Technical Report 17, Kennisdocumet Sportvisserij Nederland.

Lein, I., Holmefjord, I., and Rye, M. (1997). Effects of temperature on yolk sac larvae of atlantic halibut Hippoglossus hippoglossus l. Aquaculture, 157:123–135.

Leis, J. M. (1989). Larval biology of butterflyfishes (Pisces, Chaetodontidae): What do we really know? Envir. Biology of Fishes, 25:87–100.

Leis, J. M. and Carson-Ewart, B. M. (2000). The Larvae of Indo?Pacific Coastal Fishes: An Identification Guide to Marine Fish Larvae (Fauna Malesiana Handbook,). Brill.

Leiser, J. K. and Itzkowitz, M. (2003). The breeding system of an endangered pupfish (Cyprinodon elegans). Western North American Naturalist, 63(1):article 16.

Lema, S. C. and Nevitt, G. A. (2006). Testing an ecophysiological mechanism of morphological plasticity in pupfish and its relevance to conservation efforts for endangered Devils Hole pupfish. The Journal of Experimental Biology, 209:3499–3509.

Lenaerts, A. W., Coulter, A. A., Feiner, Z. S., and Goforth, R. R. (2015). Egg size variability in an establishing population of invasive silver carp Hypophthalmichthys molitrix (Valenciennes, 1844). Aquatic Invasions, 10:449–461.

Leonardos, I. and Sinis, A. (1998). Reproductive strategy of Aphanius fasciatus Nardo, 1827 (Pisces: Cyprinodontidae)in the Mesolongi and Etolikon lagoons (W. Greece). Fisheries Research, 35:171–181.

Leonardos, I. and Sinis, A. (1999). Reproductive strategy of Aphanius fasciatus Nardo, 1827 (Pisces: Cyprinodontidae) in the Mesolongi and Etolikon lagoons (W. Greece). Fisheries Research, 40:227–235.

Leonardos, I. and Sinis, A. (2000). Age, growth and mortality of Atherina boyeri Risso, 1810 (Pisces: Atherinidae) in the Mesolongi and Etolikon lagoons (W. Greece). Fisheries Research, 45:81–91.

Letcher, B. H. and Bengtson, D. A. (1993). Effect of food density and temperature on feeding and growth of young inland silversides (Menidia menidia). Journal of Fish Biology, 43:671–686.

Levesque, J. C. (2014). Age and growth of juvenile ladyfish (Elops sp) in Tampa Bay, Florida, USA. International Journal of Fisheries and Aquatic Studies, 2(2):145–157.

Li, C. (1983). A study on the individual fecundity and its dynamics of Trichiurus japonicus Forskal) in the East China Sea. Journal of Fisheries of China, 3:220–239.

Li, Z., Shan, X., Jin, X., and Dai, F. (2011). Long-term variations in body length and age at maturity of the small yellow croaker (Larimichthys polyactis Bleeker, 1877) in the Bohai Sea and the Yellow Sea, China. Marine Fisheries, 110:67–74.

Licandeo, R. R., Barrientos, C. A., and González, M. T. (2006). Age, growth rates, sex change and feeding habits of notothenioid fish Eleginops maclovinus from the central-southern Chilean coast. Environ. Biol Fish., 77:51–61.

Lika, K., Kooijman, S., and Papandroulakis, N. (2014). Metabolic acceleration in Mediterranean Perciformes. J. Sea Res., 94:37–46.

Lika, K., Pavlidis, M., Mitrizakis, N., Samaras, A., and Papandroulakis, N. (2015). Do experimental units of different scale affect the biological performance of european sea bass Dicentrarchus labrax larvae? Journal of Fish Biology, 86:1271–1285.

Lim, K., Le, M., An, C., Kim, S., Park, M., and Chang, Y. (2010). Reproductive cycle of yellow croaker (Larimichthys polyactis in southern waters off Korea. Japanese Society of Fisheries Science, 76:971–980.

Ling, C., Juan, Y., Chun, S., Wen-Jian, W., Yun-Zhi, Y., and Yi-Feng, C. (2015). Age, growth and reproduction of the Chinese minnow, Phoxinus oxycephalus in the Jiulongfeng Nature Reserve of the Huangshan Mountain. ACTA HYDROBIOLOGICA SINICA, 39(1):29–37.

Link, G. W. (1970). Comparative age and growth of two darters, Percina peltata (Stauffer) and Percina notogramma (Raney and Hubbs), in Virginia. PhD thesis, University of Richmond.

Linkowski, T. B. and Zukowski, C. (1980). Observation on the growth of Notothenia coriiceps neglecta Nybelin and Notothenia rossi marmorata Fischer in Admiralty Bay (King George Island, South Shetland Islands). Polish Polar Research, 1:155–162.

Liu, J., Cui, Y., and Liu, J. (1998). Food consumption and growth of two piscivorous fishes, the mandarin fish and the Chinese snakehead. Journal of Fish Biology, 53:1071–1083.

Liu, K.-M., Hung, K.-Y., and Chen, C.-T. (2001). Reproductive biology of the big eye Priacanthus macracanthus in the north-eastern waters off Taiwan. Fisheries Science, 67:1008–1014.

Liu, R. K. and Walford, R. L. (1966). Increased growth and life-span with lowered ambient temperature in the annual fish, Cynolebias adloffi. Nature, 212:1277–1278.

Liua, K.-M., Leea, M.-L., Joung, S.-J., and Chang, Y.-C. (2009). Age and growth estimates of the sharptail mola, Masturus lanceolatus, in waters of eastern Taiwan. Fisheries Research, 95:154–160.

Llompart, F. M., Colautti, D. C., Maiztegui, T., Cruz-Jiménez, A. M., and Baigún, C. R. M. (2013). Biological traits and growth patterns of pejerrey Odontesthes argentinensis. Journal of Fish Biology, 82:458–474.

Lobo, C. and Erzini, K. (2001). Age and growth of ray's bream (Brama brama) from the south of Portugal. Fisheries Research, 51:343–347.

Lockwood, S. J. (1988). The Mackerel - Its biology, assessment and the management of a fishery. Fishing News Books Ltd.

Longeneckera, K. and Langston, R. (2005). Life history of the Hawaiian blackhead triplefin, Enneapterygius atriceps (Blennioidei, Tripterygiidae). Environmental Biology of Fishes, 73(243-251).

Lønning, S., Kjørsvik, E., Haug, T., and Gulliksen, B. (1982). The early development of the halibut, Hippoglossus hippoglossus (l.), compared with other marine teleosts. Sarsia, 67:85–91.

Lorenzo, J. M. and Pajuelo, J. G. (1995). Biological parameters of the roudi escolar Promethichthys Prometheus (Pisces: Gempylidae) off the Canary Islands. Fisheries Research, 24:65–71.

Lorenzoni, M., Corboli, M., Ghetti, L., Pedicillo, G., and Carosi, A. (2007). Growth and reproduction of the goldfish Carassius auratus: a case study from Italy. In Gherardi, editor, Biological Invaders in Inland Waters, volume ch13, pages 259–274.

Lou, D. C. (1993). Growth in juvenile Scarus rivulatus and Ctnechaetus binotatus: a comparison of families Scaridae and Acanthuridae. Journal of fish Biology, 42:15–23.

Love, J. W. (2004). Age, growth, and reproduction of spotted gar, Lepisosteus oculatus (Lepisosteidae), from the Lake Pontchartrain Estuary, Louisiana. The Southwestern Naturalist, 49:18–23.

Love, M. S. and Johnson, K. (1998). Aspects of the life histories of grass rockfish, Sebastes rastrelliger, and brown rockfish, S. auriculatus, from southern California. Fish. Bull., 87:100–109.

Lowe, J. E. (1979). The Life History, Behavior, and Ecology of Etheostoma sagitta (Jordan and Swain). PhD thesis, Univ of Tennessee, Knoxville.

Lucano-Ramírez, G., Ruiz-Ramírez, S., Palomera-Sánchez, F. I., and González-Sansón, G. (2011). Reproductive biology of the Pacific sierra Scomberomorus sierra (Pisces, Scombridae) in the central Mexican Pacific. Ciencias Marinas, 37:249–260.

Luksenburg, J. A., Pedersen, T., and Falk-Petersen, I. B. (2004). Reproduction of the shorthorn sculpin Myoxocephalus scorpius in northern Norway. Journal of Sea Research, 51:157–166.

Lund, I., Steenfeldt, S. J., Suhr, K. I., and Hansen, B. W. (2008). A comparison of fatty acid composition and quality aspects of eggs and larvae from cultured and wild broodstock of common sole (*Solea solea*, L.). Aquaculture Nutrition, 14(6):544–555.

Luo, B., Lu, J., and Huang, S. (1983). maturation of the hairtails, Trichiurus japonicus Haumela (Pisces, Trichiuridae): I. The process of maturation and peculiarities. Oceanologia et Liminologia Sinica, 14:55–65.

Luther, G. (1985). Age and growth of the fishes of the genus Chirocentrus Cuvier. J. Mar. Bio. Ass. India, 27:50–67.

Lyczkowski-Shultz, J. (1971). Age and Growth of the Northern Puffer, Sphoeroides maculatus (Bloch and Schneider). PhD thesis, College of William and Mary - Virginia Institute of Marine Science.

Ma, Q., Yan, J., and Y.P., R. (2017). Linear mixed-effects models to describe length-weight relationships for yellow croaker (Larimichthys Polyactis) along the north coast of China. PLoS ONE, 12.

Maartens, L., Booth, A. J., and Hecht, T. (1999). The growth of monkfish Lophius vomerinus in Namibian waters, with a comparison of otolith and illicia methods of ageing. Fisheries Research, 44:139–148.

MacNair, L. S., Domeier, M. L., and Chun, C. S. Y. (2001). Age, growth, and mortality of california halibut, Paralichthys californicus, along southern and central California. Fish. Bull., 99:588–600.

Macpherson, E. and Raventos, N. (2005). Settlement patterns and post-settlement survival in two Mediterranean littoral fishes: influences of early-life traits and environmental variables. Marine Biology, 148:167–177.

Magnussen, E. (2007). Interpopulation comparison of growth patterns of 14 fish species on Faroe Bank: are all fishes on the bank fast-growing? Journal of Fish Biology, 71.

Maitland, P. and Lyle, A. (2010). The smelt Osmerus eperlanus in Scotland. Technical report, Freshwater Biology Association.

Malca, E., Barimo, J. F., Serafy, J. E., and Walsh, P. J. (2009). Age and growth of the gulf toadfish Opsanus beta based on otolith increment analysis. Journal of Fish Biology, 75:1750–1761.

Malkin, E. M. and Andrianova, S. B. (2008). Biology and traits of the formation of stock of big-eyed shad Alosa saposchnikowii. Journal of Ichthyology, 48:443–451.

Maltais, E., Daigle, G., Colbeck, G., and Dodson, J. J. (2010). Spawning dynamics of american shad (Alosa sapidissima) in the St. Lawrence River, Canada-USA. Ecology of Freshwater Fish, 19:586–594.

Mancera-Rodriguez, N. J. (2017). Reproductive biology of Brycon henni (Teleostei: Bryconidae) and conservation strategies for the Nare and Guatapé rivers, Magdalena River Basin, colombia. Rev. biol. trop, 65:1105–1119.

Mancera-Rodríguez, N. J. and Castro-Hernández, J. J. (2004). Age and growth of Stephanolepis hispidus (Linnaeus, 1766) (Pisces: Monacanthidae), in the Canary Islands area. Fisheries Research, 66:381–386.

Mancera-Rodríguez, N. J. and Castro-Hernández, J. J. (2015). Reproductive biology of the planehead filefish Stephanolepis hispidus (Pisces: Monacanthidae), in the Canary Islands area. Ichtyological research, 62:258–267.

Mansueti, R. J. (1962). Eggs, larvae, and young of the hickory shad, alosa mediocris, with comments on its ecology in the estuary. Chesapeake Science, 3:173–205.

Marammazi, J. G., Eskandari, G. R., Al-Mukhtar, M. A., and B. H, K. (2016). Study of spawning season and spawing ground of soboor (Tenualosa ilisha, Ham. Bunch., 1822) during its migration in Khuzestan rivers. Iranian Journal of Fisheries Sciences, 4:89–102.

Marinaro, H., Ismael, H., Montserrat, R., José, F., José, M., and José, I. (2007). First results on spawning, larval rearing and growth of the wedge sole (Dicologoglossa cuneata) in captivity, a candidate species for aquaculture. Aquacult Int, 16:69–84.

Marinaro, J. (1991). Eggs of the Microchirus (Soleidae) from the Bay of Algiers. Mar. Biol. Assoc. UK, 71:89–101.

Marinović, Z., Lujićc, J., Bolić-Trivunović, V., and Marković, G. (2016). Comparative study of growth in Carassius gibelio (Bloch, 1782) and Rutilus rutilus (L., 1758) from two Serbian reservoirs: Multi-model analysis and inferences. Fisheries Research, 173:11–19.

Marks, D. E. (1999). Life history charaxteristics of the sharpnose shiner (Notropis oxyrhynchus) and the smalleye shiner (Notropis buccula) in the Brazos River, Texas. PhD thesis, Texas Tech Univ.

Martin, F. D. and Drewry, G. E. (1978). Development of Fishes of the Mid-Atlantic Bight; an atlas of egg, larval and juvenile stages, volume 6: Stromateidae through Ogcocephalidae. Fish and Wildlife Service: U.S. Department of the Interior.

Martinez-Cardenas, L., Valdez-Hernández, E. F., González-Diaz, A. A., Soria-Barreto, M., neda Chavez, M. R. C., Ruiz-Velazco, J. M., na Messina, E. P., and Robles-Bermudez, A. (2014). Preliminary observations on Cichlasoma beani in culture conditions. Lat. Am. J. Aquat. Res., 42(3):639–643.

Martins, C., Pinheiro, P., Travassos, P., and Hazin, F. (2007). Prelimary results on reproductive biology of blue marlin, Makaira nigricans (Lacépède, 1803) in the tropical Western Atlantic Ocean. Col. Vol. Sci. Pap. ICCAT, 60(5):1636–1642.

Marty, G. D., Hinton, D. E., and Cech, J. J. J. (1995). Notes: Oxygen consumption by larval Japanese medaka with inflated or uninflated swim bladders. Transactions of the American Fisheries Society, 124:623–627.

Martyn, H. A. and Schmulbach, J. C. (1978). Bionomics of the flathead chub, Hybopsis gracilis (Richardson). Proceedings of the Iowa Academy of Science, 85:Article 5.

Mary, C. M. S., Gordon, E., and Hale, R. E. (2004). Environmental effects on egg development and hatching success in Jordanella floridae, a species with parental care. Journal of Fish Biology, 65:760–768.

Mason, W. T., Clugston, J. P., and Foster, A. M. (1992). Growth of laboratory-held gulf of Mexico sturgeon (Acipenser oxyrhynchus desotoi). The Progressive Fish-Culturist, 54:59–61.

Masuda, H., Amaoka, K., Araga, C., and Uyeno, T. (1984). The fishes of the Japanese Archipelago. Tokai University Press.

Masuda, Y., Ozawa, T., Onoue, O., and Hamada, T. (2000). Age and growth of the flathead, Platycephalus indicus, from the coastal waters of west Kyushu, Japan. Fisheries Research, 46:113–121.

Mathews, C. P. and Samuel, M. (1991). Growth, mortality and length-weight parameters for some Kuwaiti fish and shrimp. Fishbyte, 9(2):30–33.

Mathur, D. (1973). Some aspects of life history of the blackbanded darter, Percina nigrofasciata (Agassiz), in Halawakee Creek, Alabama. The American Midland Naturalist, 89(2):381–393.

Matić-Skoko, S., Ferri, J., Škeljo, F., Bartulovi'c, V., Glavić, K., and Glamuzina, B. (2011). Age, growth and validation of otolith morphometrics as predictors of age in the forkbeard, Phycis phycis (Gadidae). Fisheries Research, 112:52–58.

Maulvault, A. L., Marques, A., Rosa, R., Mendes, A., Pousao-Ferreira, P., and Anacleto, P. (2019). Experimental data from MARE, University of Lisbon. SOON TO BE PUBLISHED.

For using this data outside of the script before publication please contact Jose Moreira (j.miguel.moreira@tecnico.ulisboa.pt) or Patricia Anacleto (panacleto@ipma.pt).

Maurakis, A. G. and Green, T. D. (2001). Aspects of the life history of the cutlips minnow, Exoglossum maxillingua, from two Eeastern Pennsylvania streams. Verginia J. Sci, 52(1):25–34.

Mayden, R. L., Burr, B. M., and Dewey, S. L. (1980). Aspects of the life history of the Ozark madtom, Noturus albater, in southeastern Missouri (Pisces: Ictaluridae). The American Midland Naturalist, 104(2):335–340.

Mayden, R. L. and Walsh, S. J. (1984). Life history of the least madtom Noturus hildebrandi (Siluriformes: Ictaluridae) with comparisons to related species. The American Midland Naturalist, 112(2):349–368.

MAYER, I., SHACKLEY, S., and WITTHAMES, P. (1990). Aspects of the reproductive biology of the bass, Dicentrarchus labrax L. 11. Fecundity and pattern of oocyte development. J. Fish Biol., 36:141–148.

McAda, C. W. and Wydoski, R. S. (1985). Growth and reproduction of the flannelmouth sucker, Catostomus latipinnis, in the upper Colorado River basin, 1975-76. The Great Basin Naturalist, 45:281–286.

McArley, T. J., Hickey, A., and Herbert, N. A. (2017). Chronic warm exposure impairs growth performance and reduces thermal safety margins in the common triplefin fish (Forsterygion lapillum). Journal of Experimental Biology, 220(19):3527–3535.

McBride, R. S. and Thurman, P. E. (2003). Reproductive biology of Hemiramphus brasiliensis and H. balao (Hemiramphidae): Maturation, spawning frequency, and fecundity. Biol. Bull., 204:57–67.

McCallum, M. L. (2012). Notes on the diet and egg clutches of the pirate perch (Aphredoderus Sayanus) from Central Arkansas. Southeastern Naturalist, 11:543–545.

McCollum, A., Geubtner, J., and Hunt von Herbing, I. (2006). Metabolic costs of feeding in Atlantic cod (Gadus morhua) larvae using microcalorimetry. ICES Journal of Marine Science, 63:335–339.

McDermott, C. J. and Shima, J. S. (2006). Ontogenetic shifts in microhabitat preference o the temperate reef fish Forsterygion lapillum: implications or population limitation. Marine Ecology Progress Series, 320:259–266.

McFarlane, G. A., Jensen, J. O. T., Andrews, W. T., and Groot, E. P. (1991). Egg and yolk sac larval development of Pacific halibut (Hippoglossus stenolepis). Technical Report 24, International Pacific Halibut Commission P.O. Box 95009 Seattle, Washington 98145-2009, U.S.A.

McIlwain, J. L., Claereboudt, M. R., Al-Oufi, H. S., Zaki, S., and Goddard, J. S. (2005). Spatial variation in age and growth of the kingfish (Scomberomorus commerson) in the coastal waters of the Sultanate of Oman. Fisheries Research, 73:283–298.

McPherson, G., Squire, L., and O'Brien, J. (1992). Reproduction of three dominant Lutjanus species of the Great Barrier reef interreef fishery. Asian Fish. Sci., 5:15–24.

Mecozzi, M. (2008). Bluegill- (Lepomis macrochirus). Technical report, Wisconsin Department of Natural Resources.

Memis, D., Ercan, E., Celikkale, M. S., Timur, M., and Zarkua, Z. (2009). Growth and survival rate of russian sturgeon (Acipenser gueldenstaedtii) larvae from fertilized eggs to artificial feeding. Turkish Journal of Fisheries and Aquatic, 9:47–52.

Mendiola, D., Alvarez, P., Cotano, U., and De Murguia, A. M. (2007). Early development and growth of the laboratory reared north-east Atlantic mackerel. Journal of Fish biology, 70:911–933.

Mendiola, D., Alvarez, P., Cotano, U., Etxebeste, E., and De Murguia, A. M. (2006). Effects of temperature on development and mortality of atlantic mackerel fish eggs. Fish Res, 80:158–168.

Mensinger, A. F., Stephenson, K. A., Pollema, S. L., Richmond, H. E., Price, N., and Hanlon, R. T. (2001). Mariculture of the toadfish Opsanus tau. Biol. Bull., 201:282–283.

Mensink, P. J. (2014). Factors influencing the recruitment, growth and reproduction of a temperate reef fish, Forsterygion lapillum. PhD thesis, Victoria Univ of Wellington.

Merriner, J. V. and Laroche, J. L. (1977). Fecundity of the northern puffer, Sphoeroides maculatus, from Chesapeake Bay. Chesapeake Science, 18(1):81–83.

Mesa, M. L., Mesa, G. L., and Micalizzi, M. (2005). Age and growth of Madeira scorpionfish, Scorpaena maderensis Valenciennes, 1833, in the central Mediterranean. Fisheries Research, 74:265–272.

Messieh, S. N. (1977). Population structure and biology of alewives (Alosa pseudoharengus) and blueback herring (A. aestivalis) in the Saint John River, New Brunswick. Env. Biol. Fish., 2:195–210.

Metin, G., Ilkyza, A. T., and Kinacucgil, H. T. (2008). Growth, mortality, and reproduction of poor cod (Trisopterus minutus Linn., 1758) in the Central Aegean Sea. Turk. J. Zool., 32:43–51.

Meyer, K. A., Cassinelli, J. D., and Elle, F. S. (2008). Life history characteristics of the wood river sculpin, Cottus leiopomus (Cottidae), in Idaho. Copeia, 2008(3):648–655.

Mickle, P. and many others (2016). Biological profile for tripletail in the Gulf of Mexico and the Western Central Atlantic. Technical Report 258, Gulf States Marine Fisheries Commission.

Mies, M., Guth, A. Z., Scozzafave, M. S., and Sumida, P. Y. G. (2004). Spawning behaviour and activity in seven species of ornamental dottybacks. J. Zoo and Aquarium Res, 2:117–122.

Mikheeva, P. B., Ostrovskiia, V. I., Semenchenko, N. N., Novomodnyi, G. V., Shmigirilov, A. P., Antonov, A. L., and Barabanshchikov, E. I. (2012). Biological features of Low Amur grayling Thymallus tugarinae (Salmoniformes: Thymallidae): 2. Growth. Journal of Ichthyology, 52:628–638.

Mikheeva, P. B., Ostrovskiia, V. I., Semenchenko, N. N., Novomodnyi, G. V., Shmigirilov, A. P., Antonov, A. L., and Barabanshchikov, E. I. (2013). Biological characteristics of Lower Amur grayling Thymallus tugarinae (Salmoniformes: Thymallidae): 2. Rreproductive biology. Journal of Ichthyology, 53:281–292.

Mills, C. A. (1987). The life history of the minnow Phoxinus phoxinus (L.) in a productive stream. Freshwater Biology, 17.1:53–67.

Mills, C. A. and A:, E. (1985). The biology of Phoxinus phoxinus (L.) and other litoral zone fishes in lake konnevesi, central finland. Annales Zoologici Fennici, 22:1–12.

Mills, M. D., Belk, M. C., Rader, R. B., and Brown, J. E. (2004). Age and growth of least chub, Iotichthys phlegethontis, in wild populations. Western North American Naturalist, 64(3):409–412.

Milton, D. A. and Arthington, A. H. (1985). Reproductive strategy and growth of the australian smelt, Retropinna semoni Weber) (Pisces : Retropinnidae), and the olive perchlet, Ambassis nigripinnis (De Vis) (Pisces : Ambassidae), in Brisbane, south-eastern Queensland. J. Mar. Fresw. Res., 36:329–341.

Milton, D. A., Blaber, S. J. M., and Rawlinson, N. J. F. (1991). Age and growth of three species of tuna baitfish (genus: Spratelloides) in the tropical Indp-Pacific. Journal of fish Biology, 39:849–866.

Milton, D. A., Blaber, S. J. M., and Rawlinson, N. J. F. (1993). Age and growth of three species of clupeids from Kiribati, tropical central south Pacific. Journal of fish Biology, 43:89–1008.

Mims, S. D., Lazur, A., Shelton, W. L., Gomelsky, B., and Chapman, F. (2002). Species profile production of sturgeon. Technical report, Southern Regional Aquaculture Center.

Minckley, C. O. and Klaassen, H. E. (1969). Life history of the plains killifish, Fundulus kansae (Garman), in the Smoky Hill River, Kansas. Transactions of the American Fisheries Society, 98(3):460–465.

Mishenko, A. V., Raspopov, V. M., Ali Attaala, M., Begmanova, A., Bogatov, I. A., Sergeeva, J., Bakhareva, A. A., and Grozesku, J. N. (2016). Evaluation of the physiological state of the Volga pikeperch (Sander volgensis) fingerlings raised in a closed water system. International Journal of Fisheries and Aquatic Studies, 4:408–413.

Mitton, J. B. and Lewis, W. M. (1989). Relationships between genetic variability and life history features of bony fishes. Evolution, 43(8):1712–1723.

Miyashita, S., Sawada, Y., Okada, T., Murata, O., and Kumai, H. (2001). Morphological development and growth of laboratory-reared larval and juvenile Thunnus thynnus (Pisces: Scombridae). Fish. Bull., 99:601–616.

Miyashita, S., Tanaka, Y., Sawada, Y., Murata, O., Hattori, N., Takii, K., Mukai, Y., and Kumai, H. (2000). Embryonic development and effects of water temperature on hatching of the bluefin tuna, Thunnus thynnus. Suisanzoushoku, 48:199–207. in Japanese.

Mohammadi-Darestani, M., Mousavi-Sabet, H., Vatandoust, S., and Ahmadnejad, M. (2016). Age, growth and reproduction of the Iranian goby, Ponticola iranicus (Perciformes: Gobiidae) from the southern Caspian Sea basin. Journal of Ichthyology, 56:578–587.

Moku, M., Ishimaru, K., and Kawaguchi, K. (2001). Growth of larval and juvenile Diaphus theta (Pisces: Myctophidae) in the transitional waters of the western North Pacific. Ichthyol Res, 48.

Mongeau, J.-R., Dumont, P., and Cloutier, L. (1992). La biologie du suceur cuivré (Moxostoma hubbsi) comparée à celle de quatre autres espèces de moxostoma (M. anisurum, M. carinatum, M. macrolepidotum et M. valenciennesi). Canadian Journal of Zoology, 70:1354–1364.

Monteiro-Ribas, W. M. and Bonecker, A. C. T. (2001). Artificial fertilization and development in laboratory of Mugil Liza (Valenciennes, 1836) (Osteichthyes, Mugilidae). Bulletin of Marine Science, 68:427–433.

Monteleone, D. M. and Houde, E. D. (1990). Influence of maternal size on survival and growth of striped bass Morone saxatilis Walbaum eggs and larvae. J. Exp. Mar. Biol. Ecol., 140:1–11.

Moodie, G. E. E. and Lindsey, C. C. (1972). Life-history of a unique cyprinid fish, the chiselmouth (Acrocheilus alutaceus), in British Columbia. Syesis, 5:55–61.

Moody-Carpentera, C., Mitchell, Z., and Colombo, R. (2015). Unpublished data from Wabash River, Eastern Illinois University.

Moore, B. R., Simpfendorfer, C. A., Newman, S. J., Stapley, J. M., Allsop, Q., Sellin, M. J., and Welch, D. J. (2012). Spatial variation in life history reveals insight into connectivity and geographic population structure of a tropical estuarine teleost: king threadfin, Polydactylus macrochir. Fisheries Research, 125-126:214–224.

Moore, S. E., Hesp, S. A., Hall, N. G., and Potter, I. C. (2007). Age and size compositions, growth and reproductive biology of the breaksea cod Epinephelides armatus, a gonochoristic serranid. Journal of Fish Biology, 71:1407–1429.

Morales-Nin, B. and Ralston, S. (1990). Age and growth of Lutjanus kasmira (Forskøl) in Hawaiian waters. J. fish. Biol., 36:191–203.

Morales-Nin, B. and Sena-Carvalho, D. (1996). Age and growth of the black scabbard fish (Aphanopus carbo) off Madeira. Fisheries Research, 25:239–251.

Morales-Nine, B., Massuti, E., and Stefanescu, C. (1996). Bathymetric distribution and growth patterns of Bathypterois mediterraneus from the north-western Mediterranean Sea. Journal of Fish Biology, 49, suppl A.

Morato, T., Afonso, P., Carvalho, N., Lourinho, P., Santos, R. S., Krug, H. M., and Nash, R. D. M. (2007). Growth, reproduction and recruitment patterns of the wide-eyed flounder, Bothus podas Delaroche (Pisces: Bothidae), from the Azores. Marine Biology Research, 3:403–411.

Morato, T., Afonso, P., Santos, R. S., Krug, H. M., and Nash, R. D. M. (2003). The reproduction, age and growth of the spotted rockling. Journal of Fish Biology, 62.

Morbey, Y. E., Couture, P., Busby, P., and Shuter, B. J. (2010). Physiological correlates of seasonal growth patterns in lake trout Salvelinus namaycush. J. Fish Biol., 77:2298–2314.

Moreno, T. and Morales-Nin, B. (2003). Age determination and validation on otoliths of the sand-smelt Atherina presbyter (Cuvier, 1829) (Pisces: Atherinidae) from the central-east Atlantic. Fisheries Research, 62:77–87.

Morgan, D. L., Gill, H. S., and Potter, I. C. (2000). Age composition, growth and reproductive biology of the salamanderfish Lepidogalaxias salamandroides: a re-examination. Environmental Biology of Fishes, 57.

Morioka, S. and Matsumoto, S. (2007). Otolith development and daily increment formation in redbreast tilapia Tilapia rendalli larvae and juveniles. Aquaculture Science, 55(4):649–650.

Morita, K. (2001). The growth history of anadromous white-spotted charr in northern Japan: a comparison between river and sea life. J. Fish Biol., 59:1556–1565.

Morris, L. A. (1965). Age and growth of the river carpsucker, Carpiodes carpio, in the Missouri River. The American Midland Naturalist, 73(2):423–429.

Moser, M. L., Israel, J. A., Neuman, M., Lindley, S. T., Erickson, D. L., McCovey, B. W., and Klimley, A. P. (2016). Biology and life history of green sturgeon (Acipenser medirostris Ayres, 1854): state of the science. J. Appl. Ichthyol., 32:67–86.

Moses, B. S. (1988). Growth, mortality and potential yield of bonga, Ethmalosa fimbriata (Bowdich 1825) of Nigerian inshore waters. Fisheries Research, 6:233–247.

Moshayedi, F., Eagderi, S., Jalili, P., and Mousavi-Sabet, H. (2015). Allometric growth pattern and morphological development of sailfin molly - Poecilia latipinna (Cyprinodontiformes, Poeciliidae) during early development. Poeciliid Research, 2015, Volume 5, Issue 1., 5(1):1–7.

Motlagh, A. T. (2001). An estimate of growth parameters, moetality rates and yield-per-recruit for beluga (Huso huso) living in Caspian Sea. Iranian Journal of Fisheries Sciences, 2:25–48.

Mousavi, S. and Ghafor, A. (2014). On the conditions impressing sturgeon fish. International Journal of Advanced and Applied Sciences, 4:1–5.

Moyle, P. B., Baxter, R. D., Sommer, T., Foin, T. C., , and Matern, S. A. (2004). Biology and population dynamics of Sacramento splittail (Pogonichthys macrolepidotus) in the San Francisco Estuary: A review. Environmental Biology of Fishes, 2(2):231–241.

Moyle, P. B. and Marciochi, A. (1975). Biology of the Modoc sucker, Catostomus microps, in Northeastern California. Copeia, 1975(3):556–560.

Muir, A. M., Vecsei, P., Power, M., Krueger, C. C., and Reist, J. D. (2014). Morphology and life history of the Great Slave Lake ciscoes (Salmoniformes: Coregonidae. Ecology of Freshwater Fish, 23:453–469.

Mukai, Y., Tuzan, A. D., Shaleh, S. R. M., and Manjaji-Matsumoto, B. M. (2010). Development of sensory organs and changes of behavior in larvae of the sutchi catfish, Pangasianodon hypophthalmus. Fish Sci, 76:921–930.

Muller, B. (2012). Observations of flame chub spawning, egg and larva development. American Currents, 37(1):13–15.

Munk, P. and Nielsen, J. G. (2005). Eggs and larvae of North Sea fishes. biofolia, Frederiksberg, DK.

Murie, D. J., Parkyn, D. C., Nico, L. G., Herod, J. J., and Loftus, W. F. (2009). Age, differential growth and mortality ratesin unexploited populations of florida gar,an apex predator in the Florida Everglades. Fisheries Management and Ecology, 16:315–322.

Murphy, B. F., Leis, J. M., and Kavanagh, K. D. (2007). Larval development of the Ambon damselfish Pomacentrus amboinensis, with a summary of pomacentrid development. Journal of Fish Biology, 71:569–584.

Murua, H. (2003). Population structure, growth and reproduction of roughhead grenadier on the Flemish Cap and Flemish Pass. J. Fish Biol., 63:356–373.

Museth, J., Borgstrøm, R., Brittain, J., Herberg, I., and Naalsund, C. (2002). Introduction of the European minnow into a subalpine lake: habitat use and long-term changes in population dynamics. Journal of Fish Biology, 60:1308–1321.

Muus, B. and Dahlstrøm, P. (1968). Süsswasserfische. BLV Verlagsgesellschaft.

Mylonas, C., Mitrizakis, N., Castaldo, C., Pervino, C., Papadaki, M., Sigelaki, I., Lika, K., Kooijman, S. A. L. M., and Papandroulakis, N. (2013). Reproduction of hatchery-produced meagre Argyrosomus regius in captivity ii. Hormonal induction of spawning and monitoring of spawning kinetics, egg production and egg quality. Aquaculture, 414–415:318–327.

Nagelkerken, I. (2017). Unpublished data.

Nahrgang, J., Varpe, O., Korshunova, E., Murzina, S., Hallanger, I. G., Vieweg, I., and Berge, J. (2014). Gender specific reproductive strategies of an Arctic key species (Boreogadus saida) and implications of climate change. PLOS ONE, 9:1–11.

Narasimham, K. A. (1976). Age and growth of ribbonfish, Trichiurus trichiurus Linnaeus. Indian J. Fish., 23:174–182.

Nava-Ortega, R. A., Espino-Barr, E., Gallardo-Cabello, M., Garcia-Boa, A., Puente-Gómez, M., and Cabral-Solís, E. G. (2012). Growth analysis of the Pacific sierra Scomberomorus sierra in Colima, méxico. Revista de Biología Marina y Oceanografía, 47:273–281.

Nazari, S., Pourkazemi, M., Khoshkholgh, M. R., and Azizzadeh, L. (2013). Population structure and variation in persian sturgeon (Acipenser percicus) from the Caspian Sea as determind

from mitochondrial DNA sequences of the control region. Progress in Biological Sciences, 2:67–80.

Nelson, G. A. (2002). Age, growth, mortality, and distribution of pinfish (Lagodon rhomboides) in Tampa Bay and adjacent Gulf of Mexico waters. Fish. Bull., 100:582–592.

Nelson, G. A. and Ross, M. R. (1992). Distribution, growth and food habits of the Atlantic wolffish (Anarhichas lupus) from the Gulf of Maine-Georges Bank region. J. Northw. Atl. Fish. Sci., 13:53–61.

Nelson, S. G., Lock, S. A., and Collins, L. A. (1992). Growth of the rabbitfish Siganus randalli Woodland in relation to the feasibility of its culture on Guam. Technical report, Univ Guam Marine Lab.

Neuhold, J. M. (1954). Age and Growth of the Utah Chub, Gila atraria (Girard), in Panguitch Lake and Navajo Lake, Utah, From Scales and Opercular Bones. PhD thesis, Utah State Univ.

Newman, S. J., Cappo, M., and Williams, D. (2000). Age, growth, mortality rates and corresponding yield estimates using otoliths of the tropical red snappers, Lutjanus erythropterus, L. malabaricus and L. sebae, from the central Great Barrier Reef. Fisheries Research, 48:1–14.

nez Aguirre, A. L. I., Gallardo-Cabello, M., and Carrara, X. C. (1999). Growth analysis of striped mullet, Mugil cephalus, and white mullet, M. curema (Pisces: Mugilidae), in the Gulf of Mexico. Fish. Bull., 97:861–872.

Nieland, D. L., Thomas, R. G., and Wilson, C. A. (2002). Age, growth, and reproduction of spotted seatrout in Barataria Bay, Louisiana. Transactions of the American Fisheries Society, 131:245–259.

Nieman, R. L. and Wallace, D. C. (1974). The age and growth of the blackstripe topminnow, Fundulus notatus Rafinesque. The American Midland Naturalist, 92(1):203–205.

Ninness, M. M., Stevens, E. D., and Wright, P. A. (2006). Removal of the chorion before hatching results in increased movement and accelerated growth in rainbow trout (Oncorhynchus mykiss) embryos. Journal of Experimental Biology, 209(10):1874–1882.

Nisbet, R. M., Jusup, M., Klanjscek, T., and Pecquerie, L. (2012). Integrating Dynamic Energy Budget (DEB) theory with traditional bioenergetic models. J. Exp. Biol., 215:892–902.

Nitschke, P. and Mather, M. (2001). A comparison of length-, weight-, and age-specific fecundity relationships for cunner in Cape Cod Bay. North American Journal of Fisheries Management, 21.

Njouokou, S., Tchoumboue, J., Nguenga, D., Pouomogne, V., Gaillard, J., Sulem, S., and Bidzanga, N. (2013). Détermination des caractéristiques de maturité sexuelle du Cyprinus carpio en zone d'altitude camerounaise. Technical report, Institut de recherche Agricole pour de Développement (IRAD), Foumban, Cameroun.

Noegroho, T., Boer, M., Adrianto, L., and Sulistiono (2018). Biological characteristics of Indo-Pacific king mackerel (Scomberomorus guttatus, Bloch and Schneider 1801) in Moro waters part of Kepulauan Riau, Indonesia. Earth and Environmental Science, 176:012022.

nonez Velázquez, C. Q., Nevarez-Martínez, M. O., and Gluyas-Millán, M. G. (2000). Growth and hatching dates of juvenile Pacific sardine Sardinops caeruleus in the Gulf of California. Fisheries Research, 48:99–106.

Nunes, C., Silva, A., Marques, V., and Ganias, K. (2011). Integrating fish size, condition, and population demography in the estimation of Atlantic sardine annual fecundity. Ciencias Marinas, 37.4B:565–584.

Nytrø, A. V. (2013). The effect of temperature and fish size on growth of juvenile lumpfish (Cyclopterus lumpus L.). PhD thesis, University of Tromsø.

O'Connell, C. P. (1953). The life history of the cabezon, Scorpaenichthys marmoratus (Ayres). California Department of Fish and Game, Fish Bulletin, 93.

Ofstad, L. H., Angus, C., Pedersen, T., and Steingrund, P. (2013). Age and growth of anglerfish (Lophius piscatorius) in Faroese waters. Fisheries Research, 139:51–60.

Öhlund, G., Nordwall, F., Degerman, E., and Eriksson, T. (2008). Life history and large-scale habitat use of brown trout (Salmo trutta) and brook trout (Salvelinus fontinalis) – implications for species replacement patterns. Can. J. Fish. Aquat. Sci, 65:633–644.

Ojanguren, A. F. and Brana, F. (2003). Thermal dependence of embryonic growth and development in brown trout. Journal of fish biology, 62:580–590.

Okamoto, M., Motomura, H., and Asahida, T. (2011). Redescription of a poorly known deepwater cardinalfish, Epigonus affinis (Actinopterygii: Perciformes: Epigonidae), and comparison with related species. Species diversity, 16:85–92.

O'Kelley, C. T. and Powers, S. L. (2007). Life-history aspects of Hypentelium etowanum (Alabama hog sucker) (Actinopterygii: Catostomidae) in Northern Georgia. Southeastern Naturalist, 6(3):479–490.

Okgerman, H., Elp, M., and Yardimci, C. H. (2011). Growth, the length-weight relationship, and reproduction in vimba (Vimba vimba L. 1758) sampled from an oligo-mesotrophic lake in northwest Anatolia (Turkey). Turk J Zool, 35:87–96.

Oldfield, R. G. (2009). Growth patterns in midas cichlids are not consistent with a hypothesis of socially controlled sex determination. Copeia, 2009(1):71–77.

Olinger, C. T., Peoples, B. K., and Frimpong, E. A. (2016). Reproductive life history of Heterandria bimaculata (Heckel, 1848) (Poeciliinae: Poeciliidae) in the Honduran interior highlands: Trait variation along an elevational gradient. Neotropical Ichthyology, 14(1):e150050.

Oliva-Paterna, F. J., Torralva, M. M., and Fernandez-Delgado, C. (2002). Age, growth and reproduction of cobitis paludica in a seasonal stream. Journal of Fish Biology, 60:389–404.

Olivotto, I., Rollo, A., Sulpizio, R., Avella, M., Tosti, L., and Carnevali, O. (2006). Breeding and rearing the sunrise dottyback Pseudochromis flavivertex: the importance of live prey enrichment during larval development. Aquaculture, 255:480–487.

Olsen, Z., McDonald, D., and Bumguardner, B. (2018). Intraspecific variation in life history strategies and implications for management: A case study of black drum (Pogonias cromis) in the Upper Laguna Madre, Texas USA. Fisheries Research, 207:55–62.

Olson, A. D. and Martin, T. H. (2016). Life history traits of the mirror shiner, Notropis spectrunculus, in Western North Carolina. Southeastern Naturalist, 15(1):102–114.

O'Neil, P. and Drawer, P. O. (1981). Life history of Etheostoma coosae (pisces, Percidae) in Barraree Creek, Alabama. Tulane Studies in Zoology and Botany, 23(1):75–84.

Oosten, J. V. and Hile, R. (1949). Age and growth of the lake whitefish, Coregonus Clupeaformis (Mitchill), in Lake Erie. Transactions of the American Fisheries Society, 77:178–249.

Orlov, A. M., Kuznetsova, E. N., and Mukhametov, I. N. (2011). Age and growth of the Pacific halibut Hippoglossus stenolepis and the size-age composition of its catches in the northwestern part of the Pacific Ocean. Journal of Ichthyology, 51:306–323.

Orlova, A. M., Vedishcheva, E. V., Trofimova, A. O., and Orlova, S. Y. (2018). Growth and age of the roughhead grenadier Macrourus berglax in waters off Southwest Greenland. Journal of Ichthyology, 58:389–395.

Orsi, J. A. and Jaenicke, H. W. (1996). Marine distribution and origin of prerecruit chinook salmon, Oncorhynchus tshawytscha, in southeastern Alaska. Fishery Bulletin, 94:482–497.

Ortega-Salas, A. A., Cortés, I., and Reyes-Bustamante, H. (2009). Fecundity, growth, and survival of the angelfish Pterophyllum scalare (Perciformes: Cichlidae) under laboratory conditions. Rev. Biol. Trop. (Int. J. Trop. Biol. ISSN-0034-7744), 57(3):741–747.

Ortega-Salas1, A. and Reyes-Bustamante, H. (2006). Initial sexual maturity and fecundity of the goldfish Carassius auratus (Perciformes: Cyprynidae) under semi-controlled conditions. Rev. Biol. Trop. (Int. J. Trop. Biol. ISSN-0034-7744), 54(4):1113–1116.

Ortiz-Delgado, J. B., Funes, V., and Sarasquete, C. (2019). The organophosphate pesticide -OP-malathion inducing thyroidal disruptions and failures in the metamorphosis of the Senegalese sole, *Solea senegalensis*. BMC Veterinary Research, 15(1):1–21.

Ostrovsky, I. and Walline, P. (1999). Growth and production of the dominant pelagic fish, Acanthobrama terraesanctae, in subtropical Lake Kinneret, Israel. Journal of Fish Biology, 54:18–32.

O'Sullivan, S., Moriarty, C., FitzGerald, R. D., Davenport, J., and Mulcahy, M. F. (2003). Age, growth and reproductive status of the European conger eel, Conger conger (L.) in Irish coastal waters. Fisheries Research, 64:55–69.

Otterlei, E., Nyhammer, G., Folkvord, A., and Stefansson, S. O. (1999). Temperature and size dependent growth of larval and early juvenile Atlantic cod (Gadus morhua): a comparative study of norwegian coastal cod and northeast arctic cod. Can. J. Fish. Aquat. Sci., 56:2099–2111.

Ouabadi, T. (2015). PhD thesis, ENSSMAL Univ., Algiers.

Oxenford, H. A., Hunte, W., Deane, R., and Campana, S. E. (1994). Otolith age validation and growth-rate variation in flyingfish (Hirundichthys affinis) from the eastern Caribbean. Marine Biology, 118:585–592.

Ozaydin, O., Bilecenoglu, M., and Kaya, M. (2000). Age and growth of the curled picarel Centracanthus cirrus Rafinesque, 1810 (Osteichthyes: Ccentracanthidae) in Northern Cyprus, Eastern Mediterranean Sea. Acta Adriat., 42(2):35–42.

Paepke, H.-J. (1983). Die Stichlinge., volume 10 of Die Neue Brehm-Buecherei. A. Ziemsen Verlag DDR, WittenBerg Lutherstadt.

Page, L. M. (1974). The life history of the spottail darter, Etheostoma squamiceps, in Big Creek, Illinois, and Ferguson Creek, Kentucky. Technical report, Dept Energy and Natural Resources, Illenois Natural History Survey.

Page, L. M. (1975). The life history of the stripetail darter, Etheostoma kennicotti, in big creek, illinois. Technical report, State of Illinois, Department of Registration and Education, NATURAL HISTORY SURVEY DIVISION.

Page, L. M. (1980). The life histories of the Etheostoma olivaceum and Etheostoma striatulum, two species of darters in central tennessee. Technical report, State of Illinois, Department of Registration and Education, NATURAL HISTORY SURVEY DIVISION.

Page, L. M. and Burr, B. M. (1976). The life history of the slabrock darter, Etheostoma smithi, in ferguson creek, kentucky. Technical report, State of Illinois, Department of Registration and Education, NATURAL HISTORY SURVEY DIVISION.

Page, L. M. and Burr, B. M. (1991). A Field Guide to Freshwater Fishes of North America, north of Mexico. Houghton Mifflin Company.

Page, L. M. and Mayden, R. L. (1981). The life history of the Tennessee snubnose darter, Etheostoma simoterum, in Bruce Creek, Tessessee. Technical report, Dept Energy and Natural Resources; Illinois Institute for Natural Resources.

Page, L. M. and Smith, P. W. (1970). The life history of the dusky darter, Percina sciera, in the Embarras River, Illinois. Technical report, ILLINOIS NATURAL HISTORY SURVEY, Urbana, Illinois.

Pajuelo, J. G. and Lorenzo, J. M. (2000). Biology of the sand smelt, Atherina presbyter (Teleostei: Atherinidae), off the Canary Islands (central-east Atlantic). Environmental Biology of Fishes, 59:91–97.

Pajuelo, J. G., Lorenzo, J. M., and Domínguez-Seoane, R. (2003). Age estimation and growth of the zebra seabream Diplodus cervinus cervinus (Lowe, 1838) on the Canary Islands shelf (Central-east Atlantic). Fisheries Research, 62:97–103.

Pajuelo, J. G., Martínez, I., González, J. A., Lorenzo, J. M., García-Mederos, A., Domínguez-Seoane, R., and Hernández-Cruz, C. M. (2006a). Growth pattern and age estimation of the coastal sparid fish Pagrus auriga on the Canary Islands shelf. Fisheries Research, 82:7–13.

Pajuelo, J. G., Socorro, J., González, J. A., Lorenzo, J. M., Pérez-Penalvo, J. A., Martínez, I., and Hernáandez-Cruz, C. M. (2006b). Life history of the red-banded seabream Pagrus auriga (Sparidae) from the coasts of the Canarian archipelago. J. Appl. Ichthyol., 22:430–436.

Palazón-Fernaández, J. L., Arias, A. M., and Sarasqete, C. (2001). Aspects of the reproductive biology of the toadfish, Halobatrachus didactylus (Schneider, 1801) (Pisces: Batrachoididae). Sci. Mar., 65(2):131–138.

Palmer, C. J. and Culley, M. B. (2006). The egg and early life stages of the sandsmelt, Atherina presbyter Cuvier. Journal of Fish Biology, 24:537–544.

Panchenko, V. V. (2010). Age and growth of far eastern staghorn sculpin Gymnocanthus herzensteini (Cottidae) in Peter the Great Bay (the Sea of Japan). Journal of Ichthyology, 50:295–300.

Panchenko, V. V. (2012). Growth and age of staghorn sculpins of the genus Gymnocanthus (C"ottidae) in Peter the Great Bay and adjacent waters of Primorye (Sea of Japan). Journal of Ichthyology, 52:226–238.

Panchenko, V. V., Pushina, O. I., Milovankin, P. G., and Nuzhdin, V. A. (2015). Distribution and some features of biology of Berg's longhorn sculpin Taurocottus bergii (Cottidae) in the northwestern part of the Sea of Japan. Journal of Ichthyology, 55:388–396.

Panek, F. M. and Weis, J. S. (2012). Age, growth, and reproduction of the eastern mudminnow (Umbra pygmaea) at the Great Swamp National Wildlife Refuge, New Jersey. Northeastern Naturalist, 19:217–228.

Panhwar, S. K., Liub, Q., and Siddiqui, G. (2013). Growth, mortality and stock assessment of kelee shad, Hilsa kelee (fam: Clupeidae) in the coastal waters of Pakistan. Journal of Ichthyology, 53:365–371.

Panter, G., Glennon, Y., Robinson, J., Hargreaves, A., and Murray-Smith, R. (2012). Effects of the anti-androgen, bicalutamide, in a reduced life-cycle study with the fathead minnow (Pimephales promelas). Aquatic toxicology, 114-115:31–38.

Papadaki, M., Peleteiro, J. B., Alvarez-Blázquez, B., Villanueva, J. L. R., Linares, F., Vilar, A., Rial, E. P., Lluch, N., Fakriadis, I., Sigelaki, I., and Mylonas, C. C. (2018). Age and growth of southwestern Atlantic wreckfish Polyprion americanus. Fishes, 3:43.

Papadakis, I., Kentouri, M., Divanach, P., and Mylonas, C. (2013). Ontogeny of the digestive system of meagre Argyrosomus regius reared in a mesocosm, and quantitative changes of lipids in the liver from hatching to juvenile. Aquaculture, 388–391:76–88.

Papandroulakis (2012). pers. com.

Papandroulakis (2013). pers comm.

Papandroulakis, N., Kentouri, M., Maingot, E., and Divanach, P. (2005a). Mesocosm: a reliable technology for larval rearing of Diplodus puntazzo and Diplodus sargus sargus. Aquaculture International, 12:345–355.

Papandroulakis, N., Lika, K., Kristiansen, T. S., Oppedal, F., Divanach, P., and Pavlidis, M. (2014). Behaviour of european sea bass, Dicentrarchus labrax L., in cages - impact of early life rearing conditions and management. Aquaculture Research, 49:1545–1558.

Papandroulakis, N., Mylonas, C. C., Maingot, E., and Divanach, P. (2005b). First results of greater amberjack (Seriola dumerili) larval rearing in mesocosm. Aquaculture, 250:155–161.

Pappantoniou, A., Dale, G., and Schmidt, R. E. (1964). Aspects of the life history of the cutlips minnow, Exoglossum maxillingua, from two Eeastern Pennsylvania streams. J. Freshw. Ecol., 2(5):440–458.

Pardue, G. B. (1993). Life history and ecology of the mud sunfish (Acantharchus pomotis). Copeia, 1993(2):533–540.

Paredes, F. and Bravo, R. (2005). Reproductive cycle, size at first maturation and fecundity in the golden ling, Genypterus blacodes, in Chile. New Zealand Journal of Marine and Freshwater Research, 39:1085–1096.

Parka, C.-B., Jun-ya Aokia, J., Leec, J.-S., Nagae, M., Lee, Y.-D., Sakakuraf, Y., Hagiwarab, A., and Soyano, K. (2010). The effects of 17β-estradiol on various reproductive parameters in the hermaphrodite fish Kryptolebias marmoratus. Aquatic Toxicology, 96:273–279.

Parker, H. L. (1964). Natural history of Pimephales vigilax (Cyprinidae). The Southwestern Naturalist, 8(4):228–235.

Parr, C. (2013). Lepomis macrochirus (on-line). `https://animaldiversity.org/accounts/Lepomis_macrochirus/`.

Parra, G. and Yufera, M. (2001). Comparative energetics during early development of two marine fish species, *Solea senegalensis* (Kaup) and *Sparus aurata* (L.). The Journal of experimental biology, 204(Part 12):2175–2183.

Parrish, P. R., Dyar, E. E., Enos, J. M., and Wilson, W. G. (1978). Chronic toxicity of chlordane, trifluralin, and pentachlorphenol to sheepshead minnows (Cyprinodon variegatus). Technical report, Environmental Research Laboratory, Office Of Research and Development, U.S. Environmental Protection Agency.

Parrott, J. and Balakrishnan, V. (2017). Life-cycle exposure of fathead minnows to environmentally relevant concentrations of the β-blocker drug propranolol. Environmental Toxicology and Chemistry, 36:1644–1651.

Parsley, M. J. and Kofoot, E. (2013). Effects of incubation substrates on hatch timing and success of white sturgeon (*Acipenser transmontanus*) embryos. Technical Report 5180, U.S. Department of the Interior; U.S. Geological Survey.

Parsons, J. W. (1954). Growth and habits of the redeye bass. Transactions of the American Fisheries Society, 83(1):202–211.

Patimar, R., Nadjafypour, E., Yaghouby, M., and Nadjafy, M. (2010). Reproduction characteristics of a stunted population of rudd, Scardinius erythrophthalmus (Linnaeus, 1758) living in the Anzali Lagoon (the southwest Caspian Sea, Iran). Journal of Ichthyology, 50:1060–1065.

Patten, B. G. (1971). Spawning and fecundity of seven species of Northwest American cottus. The American Midland Naturalist, 85(2):493–506.

Patterson, J. T., Allgood, T. G., and Green, C. C. (2013). Intraspecific variation in reproductive potential with maternal body size in gulf killifish Fundulus grandis. Aquaculture, 384-387:134–139.

Paul, L. J. and Horn, P. L. (2009). Age and growth of sea perch (Helicolenus percoides) from two adjacent areas off the east coast of South Island, New Zealand. Fisheries Research 95, 95:169–180.

Paulsen, H., Kjesbu, O. S., Buehler, V., Case, R. A. J., Clemmesen, C., Carvalho, G., Hauser, L., Hutchinson, W., Moksness, E., Otterå, H., Thorsen, A., and Svaasand, T. (2009). Effects of egg size, parental origin and feeding conditions on growth of larval and juvenile cod Gadus morhua. Journal of Fish Biology, 75:516–537.

Pavlov, D. A., Emel'yanova, N. G., Ha, V. T., and Thuan, L. T. B. (2013). Age and growth of manybar goatfish Parupeneus multifasciatus (Mullidae) from the Nha Trang Bay of the South China Sea. Journal of Ichthyology, 53:478–485.

Pavlov, D. A., Emel'yanova, N. G., Ha, V. T., and Thuan, L. T. B. (2015). Otolith morphology, age, and growth of freckled goatfish Upeneus tragula (Mullidae) in the coastal zone of Vietnam. Journal of Ichthyology, 55:363–372.

Pavlov, D. A. and Moksness, E. (1995). Development of wolffish eggs at different temperature regimes. Aquaculture Internat, 3:315–335.

Pawiroredjo, P. A. (2004). Temperature effects on spawning and fingerling production of channel catfish Ictalurus punctatus. PhD thesis, Louisiana State University.

Payne, A. I. (1976). The determination of age and growth from the scales in Barbus liberiensis (Pisces, Cyprinidae). Technical report.

Pecquerie, L. (2008). Bioenergetic modelling of the growth, development and reproduction of a small pelagic fish: the Bay of Biscay anchovy. PhD thesis, VU University Amsterdam and Rennes University Agrocampus.

Pecquerie, L., Petitgas, P., and Kooijman, S. (2009). Modeling fish growth and reproduction in the context of the Dynamic Energy Budget theory to predict environmental impact on anchovy spawning duration. Journal of Sea Research, 62:93–105.

Penaz, M. and Tesch, F. (1970). Sex rate and growth of eel (Anguilla anguilla) in different biotopes of North Sea and River Elbe.. Berichte der Deutschen Wissenschaftlichen Kommission für Meeresforschung, 21:290.

Penning de Vries, F. W. T. (1969). Enige metingen over het standaard metabolism en het actief metabolism bij Gobiidae. Int. Rep. Neth. Inst. Sea Res., 1969(2).

Perera-Garcia, M. A., Mendoza-Carranza, M., Contreras-Sánchez, W., Ferrara, A., Huerta-Ortiz, M., and Hernandez-Gomez, R. E. (2013). Comparative age and growth of common snook Centropomus undecimalis (Pisces: Centropomidae) from coastal and riverine areas in Southern Mexico. Rev. Biol. Trop., 61:807–819.

Peres, M. B. and Haimovici, M. (2004). Age and growth of southwestern Atlantic wreckfish Polyprion americanus. Fisheries Research, 66:157–169.

Perkin, J. S., Shattuck, Z. R., and Bonner, T. H. (2012). Life history aspects of a relict ironcolor shiner Notropis chalybaeus population in a novel spring environment. The American Midland Naturalist, 167(1):111–126.

Persson, L., Claessen, D., Roos, A. M. d., Byström, P., Sjögren, S., Svanbäck, R., Wahlstrm, E., and Westman, E. (2004). Cannibalism in a size-structured population: energy extraction and control. Ecological monographs, 74:135–157.

Peter, H. K. (1999). Fecundity and egg sizes of pelagic fishes, Stolothrissa tanganicae and Limnothrissa miodon in relation to size of the fish. `https://www.geo.arizona.edu/nyanza/pdf/Peter.pdf`. No date found, supervised by Peter McIntyre.

Peters, J. (2009). Kennisdocument donderpad het geslacht cottus.

Peterson, D., Vecsei, P., and Hochleithner, M. (2006). Threatened fishes of the world: Acipenser ruthenus Linnaeus, 1758 (Acipenseridae). Environmental Biology of Fishes.

Peterson, M. S., Nicholson, L. C., Snyder, D. J., and Fulling, G. L. (1999). Growth, spawning preparedness, and diet of Cycleptus meridionalis (Catostomidae). Transactions of the American Fisheries Society, 128:900–908.

Philippart, J.-C. and Ruwet, J.-C. (1982). Ecology and distribution of tilapias. In Pullin, R. S. V. and Lowe-McConnell, R. H., editors, The biology and culture of tilapias, number 7 in ICLARM conference proceedings, pages 15–59, Manila, Philippines. International Center for Living Aquatic Resources Management.

Phillips, E. C., Ewert, Y., and Speares, P. A. (2007). Fecundity, age and growth, and diet of Fundulus diaphanus (banded killifish) in Presque Isle Bay, Lake Erie. Northeastern Naturalist, 14(2):269–278.

Pikitch, E. K., Doukakis, P., Lauck, L., Chakrabarty, P., and Erickson, D. L. (2005). Status, trends and management of sturgeon and paddlefish fisheries. Fish Fish, 6:233–265.

Pineiro, C. and Sainza, M. (2003). Age estimation, growth and maturity of the European hake (Merluccius merluccius (Linnaeus, 1758)) from Iberian Atlantic waters. ICES Journal of Marine Science, 60.

Pipe, R. K. and Walker, P. (1987). The effect of temperature on development and hatching of scad, Trachurus trachurus L., eggs. Journal of Fish Biology, 31:675–682.

Pitargue, C. K. (2007). Husbandry Manual For White's Seahorse. Western Sydney Institute of TAFE, Richmond, Australia.

Planas, M., Quintas, P., Chamorro, A., and Silva, C. (2010). Female maturation, egg characteristics and fatty acids profile in theseahorse Hippocampus guttulatus. Animal Reproduction Science, 122:66–73.

Plath, M., Sarbu, A., Erkoc, K., Bierbach, D., Jourdan, J., and Schleucher, E. (2013). Energetic costs of group-living? a reversed "group effect" in shoaling minnows (Phoxinus phoxinus). Bulletin of Fish Biology, 14:1–10.

Poezzhalova-Chegodaeva, E. A. (2016). Biology of eelpout Zoarces elongatus (Zoarcidae) from Tauysk Bay, the Sea of Okhotsk. Journal of Ichthyology, 56:569–577.

Pombo, M., Denadai, M. R., Bessa, E., Santos, F. B., de Faria, V. H., and Turra, A. (2014). The barred grunt Conodon nobilis (Perciformes: Haemulidae) in shallow areas of a tropical bight: spatial and temporal distribution, body growth and diet. Helgol Mar Res, 68:271–279.

Pompei, L., Franchi, E., Giannetto, D., and Lorenzoni, M. (2012). Growth and reproductive properties of tench, Tinca tinca Linnaeus, 1758 in Trasimeno Lake (Umbria, Italy). Knowledge and Management of A quatic Ecosystems (2012) 406, 07, 406(7).

Pope, E. C., Hays, G. C., Thys, T. M., Doyle, T. K., Sims, D. W., Queiroz, N., Hobson, V. J., Kubicek, L., and Houghton, J. D. R. (2010). The biology and ecology of the ocean sunfish

Mola mola: a review of current knowledge and future research perspectives. Rev Fish Biol Fisheries.

Pope, K. L., Geraets, W. T., and Willis, D. W. (1996). Egg development in a high-density black crappie (Pomoxis nigromaculatus) population. Journal of Freshwater Ecology, 11(4):451–458.

Pope, K. L. and Willis, D. W. (1998). Early life history and recruitment of black crappie (Pomoxis nigromaculatus) in two South Dakota waters. Ecology of Freshwater Fish, 7:56–68.

Porcu, C., Follesa, M. C., Grazioli, E., Deiana, A. M., and Cau, A. (2010). Reproductive biology of a bathyal hermaphrodite fish, Bathypterois mediterraneus (Osteichthyes: Ipnopidae) from the south-eastern Sardinian Sea (central-western Mediterranean). Journal of the Marine Biological Association of the United Kingdom, 90.

Postlethwait, J. H., Yan, Y., Desvignes, T., Allard, C., Titus, T., Francois, N. R. L., and Detrich, H. W. (2016). Embryogenesis and early skeletogenesis in the Antarctic bullhead notothen, Notothenia coriiceps. Developmental Dynamics, 245:1066–1080.

Potter, I. C., Neira, F. J., Wise, B. S., and Wallace, J. H. (1994). Reproductive biology and larval development of the terapontid Amniataba caudavittata, including comparisons with the reproductive strategies of other estuarine teleosts in temperate Western Australia. Journal of Fish Biology, 45:57–74.

Poulson, T. L. (1963). Cave adaptation in Amblyopsid fishes. The American Midland Naturalist, 70(2):257–290.

Povoa, I., Davie, A., Treasurer, J., and Migaud, H. (2011). Broodstock spawning and larviculture of whiting (Merlangius merlangus L.) reared in captivity. Aquaculture Research, 42.

Powles, P. M. and Sandeman, I. M. (2008). Growth, summer cohort output, and observations on the reproduction of brook silverside, Labidesthes sicculus (Cope) in the Kawartha Lakes, Ontario. Environ Biol Fish, 82:421–431.

Prasad, R. R., Jaiswar, A. K., Reddy, S. B., Chakraborty, S. K., Palaniswamy, R., and Parida, P. (2005). Growth, mortality and yield per recruit of Polynemus heptadactylus (Cuvier) (Teleostei: Polynemidae) from Mumbai waters, India. Fisheries Research, 76:155–161.

Prodanov, K. (1997). Studies & revies 68; General fisheries council for the Mediterranean. Technical report, FAO, Rome.

Prosch, R. M. (1991). Reproductive biology and spawning of the myctophid Lampanyctodes hectoris and the sternoptychid Maurolicus muelleri in the southern Benguela ecosystem. S. Afr. J. mar. Sci., 10.

Puchala, E. A., Parrish, D. L., and Ogle, D. H. (2018). Size and age of stonecats in Lake Champlain; estimating growth at the margin of their range to aid in population management. North American Journal of Fisheries Management, 38:1316–1323.

Q. Queiros, . (2017). Experimental data not yet published: 4 ponds.

Qin, J., Xiang, M., Jia, M., Cheng, F., Zhang, L., Schmidt, B. V., Liu, J., , and Xie1, S. (2020). Combined opportunistic and equilibrium life-history traits facilitate successful invasions of the Shimofuri goby (Tridentiger bifasciatus). Aquatic Invasions, 15(in press).

Queiroz, H. (2000). Natural history and conservation of pirarucu, Arapaima gigas, at the Amazonian Varzea: red giants in muddy waters. PhD thesis, University of St. Andrews.

Quigley, D. T. G., Igoe, F., and OConnor, W. (2004). The European smelt Osmerus eperlanus L. in Ireland: General biology, ecology, distribution and status with conservation recommendations. Threatened Irish Freshwater Fishes, 104B(3):57–66.

Raczynski, M., Czerniejewski, P., Witkowska, M., and Kiriaka, B. (2008). Age and growth rate of roach (Rutilus rutilus L.) from 3 lakes used for recreational fishing. Teka Kom. Ochr. Kszt. Środ. Przyr., 5A:106–116.

Radebe, P. V., Mann, B. Q., Beckley, L. E., and Govender, A. (2002). Age and growth of Rhabdosargus sarba (Pisces: Sparidae), from Kwazulu-Natal, South Africa. Fisheries Research, 58:193–201.

Rahman, M. J. and Cowx, I. G. (2006). Lunar periodicity in growth increment formation in otoliths of hilsa shad (Tenualosa ilisha, Clupeidae) in Bangladesh waters. Fisheries Research, 81:342–344.

Raibley, P. T. and Jahn, L. A. (1991). Characteristics of commercially harvested channel catfish from areas of the Mississippi River along Illinois: Commercial harvest and the 15.0– in minimum length limit. Journal of Freshwater Ecology, 6:363–376.

Rakes, P. L. (1989). Life History and Ecology of the Barrens Topminnow, undulus julisia Williams and Etnier (Pisces, Fundulidae). PhD thesis, Univ. of Tennessee, Knoxville.

Ramírez-García, A. and Piller, K. R. (2018). Aspects of the reproductive biology and growth of the Mississippi silvery minnow, Hybognathus nuchalis (Agassiz, 1855) (Teleostei: Cyprinidae) from the Pearl River, Louisiana. Southeastern Fishes Council Proceedings, 58:1113–1116.

Ramírez-Pérez, J. S., Melo-Barrera, F. N., and Ayala-Bobadilla, L. E. (2011). Age and growth of pacific golden-eye tilefish (Caulolatilus affinis) in the central region of the Gulf of California. Ciencias Marinas, 37:71–85.

Ramos-Miranda, J., Bejarano-Hau, K., Flores-Hernández, D., and Ayala-Pérez, L. A. (2009). Growth, mortality, maturity, and recruitment of the star drum (Stellifer lanceolatus) in the southern Gulf of Mexico. Ciencias Marinas, 35:245–257.

Raney, E. C. and Lachner, E. A. (1946). Age, growth, and habits of the hog sucker, Hypentelium nigricans (LeSueur), in New York. The American Midland Naturalist, 36(1):76–86.

Raventos, N. and Planes, S. (2009). Maternal size effects on early life traits of the temperate fish Symphodus roissali. Aquat. Biol., 4:1–6.

Raymond, J. A. (1986). Growth of wild and hatchery juvenile coho salmon in an interior Alaska stream. Technical report, Alaska Dept of Fish and Game.

Ré, P. (1996). Anchovy spawning in the Mira Estuary (southwestern Portugal). Scientia Marina, 60(Suppl.2):141–153.

Ré, P. and Meneses, I. (2008). Early Stages of Marine Fishes Occurring in the Iberian Peninsula. IPIMAR, Lisboa. 283 p.

Reñones, O., Grau, A., Mas, X., Riera, F., and Saborido-Rey, F. (2010). Reproductive pattern of an exploited dusky grouper Epinephelus marginatus (Lowe 1834) (Pisces: Serranidae) population in the western Mediterranean. Scientia Marina, 74(3):523–537.

Reed, R. J. (1957). Phases of the life history of the rosyface shiner, Notropis rubellus, in Northwestern Pennsylvania. Copeia, 1957(4):286–290.

Reed, R. J. (1971). Biology of the fallfish, Semotilus corporalis (Pisces, Cyprinidae). Transactions of the American Fisheries Society, 100(4):717–725.

Reed, R. J. and Moulton, J. C. (1973). Age and growth of blacknose dace, Rhinichthys atratulus and longnose dace, R. cataractae in Massachusetts. American Midland Naturalist, 90:206.

Regner, S. (1996). Effects of environmental changes on early stages and reproduction of anchovy in the Adriatic Sea. Scientia Marina, 60 (Supl. 2):167–177.

Régnier, T., Gibb, F. M., and Wright, P. J. (2018). Temperature effects on egg development and larval condition in the lesser sandeel, Ammodytes marinus. J. Sea Research, 134:34–41.

Reid, S. M. (2009). Age, growth and mortality of black redhorse (Moxostoma duquesnei) and shorthead redhorse (M. macrolepidotum) in the Grand River, Ontario. J. Appl. Ichthyol., 25:178–183.

Reinartz, R. (2002). Sturgeons in the danube river. Literature study on behalf of IAD. Landesfischerei-verband for Bayern e.V. and Bezirk Oberpfalz, pages 1–150.

Remoissenet, G., Maamaatuaiahutapu, M., Buchet, V., Goguenheim, J., Brito, T., Cuzon, G., and Gaxiola, G. (2012). Age and growth of stargazer (Uranoscopus scaber L., 1758) in the southeastern Black Sea. https://www.was.org/meetings/ShowAbstract.aspx?Id=26809.

Resh, C. A., Galaska, M. P., and Mahon, A. R. (2018). Genomic analyses of Northern snakehead (Channa argus) populations in North America. PeerJ.

Rey, J., Fernández-Peralta, L., García, A., Nava, E., Clemente, M. C., Otero, P., Villar, E. I., and Pineiro, C. G. (2016). Otolith microstructure analysis reveals differentiated growth historiesin sympatric black hakes (Merluccius polli and Merluccius senegalensis). Fisheries Research, 179.

Reznick, D. N. (1996). Unpublished dataset.

Reznick, D. N. and Bryant, M. J. (1996). Life-history evolution in guppies (Poecilia reticulata: Poeciliidae). V. Genetic basis of parallelism in life histories. The American Naturalist, 147(3):339–359.

Reznick, D. N., Bryant, M. J., Roff, D., Ghalambor, C. K., and Ghalambor, D. E. (2004). Effect of extrinsic mortality on the evolution of senescence in guppies. Nature, 431(7012):1095.

Ribeiro, L., Engrola, S., and Dinis, M. T. (2017). Weaning of Senegalese sole (*Solea senegalensis*) postlarvae to an inert diet with a co-feeding regime. Ciencias Marinas, 31(12):327–337.

Ribeiro, L., Sarasquete, C., and Dinis, M. T. (1999). Histological and histochemical development of the digestive system of *Solea senegalensis* (Kaup, 1858) larvae. Aquaculture, 171(3-4):293–308.

Richards, W. J. (2003). Early stages of Atlantic fishes: An identificatiob guide of the western central North Atlantic. Taylor & Francis.

Richards, W. J. (2005). Early Stages of Atlantic Fishes: An Identification Guide for the Western Central North Atlantic, volume 1. Taylor & Francis.

Richards, W. J. (2008). Identification Guide of The early life history stages of fishes from the Waters of Kuwait in the Arabian Gulf, Indian Ocean. Kuwait Institute for Scientific Research, P.O.Box 24885, 13109 Safat, Kuwait.

Rico, V., Lorenzo, J. M., Gonz'alez, J. A., Krug, H. M., Mendonça, A., Gouveia, E., and Dias, M. A. (2001). Age and growth of the alfonsino Beryx splendens Lowe, 1834 from the Macaronesian archipelagos. Fisheries Research, 49:233–240.

Rijnsdorp, A. D. and Ibelings, B. (1989). Sexual dimorphism in the energetics of reproduction and growth of North Sea plaice, Pleuronectes platessa L. J. Fish Biol., 35:401–415.

Rinne, J. N. (1982). Movement, home range, and growth of a rare southwestern trout in improved and unimproved habitats. North American Journal of Fisheries Management, 2(2):150–157.

Roberts, M. E., Burr, B. M., Whiles, M. R., and Santucci, V. J. (2006). Reproductive ecology and food habits of the blacknose shiner, Notropis heterolepis, in Northern Illinois. The American Midland Naturalist, 155(1):70–83.

Robertson, D. A. (1977). Planktonic eggs of the lanternfish, Lampanyctodes hectoris (family Myctophidae). Deep-Sea Research, 24.

Robertson, S. M., Bonner, T. H., and Fries, J. N. (2016). Effects of habitat utilization on the life histories of two imperiled, sympatric Dionda (Cyprinidae) in the Rio Grande Basin, Texas. The Southwestern Naturalist, 175(2):222–232.

Robillard, E., Reiss, C. S., and Jones, C. M. (2009). Age-validation and growth of bluefish (Pomatomus saltatrix) along the east coast of the United States. Fisheries Research, 95:65–75.

Robinson, J. M., Jirka, K. J., and Chiott, J. A. (2010). Age and growth analysis of the central mudminnow, Umbra limi (Kirtland, 1840). J. Appl. Ichtthyol., 26:89–94.

Robotham, P. W. J. (1981). Age, growth and reproduction of a population of spined loach, Cobitis taenia (L.). Hydrobiologia, 85:129–136.

Rochard, E., P., W., Castelnaud, G., and Lepage, M. (1991). Elements de systematique et de biologie des populations sauvages d esturgeons. In Williot, P., editor, Acipenser, pages 475–507. Cemagref, Antony.

Rodrigues, K. A., Leonarduzzi, E., Macchi, G. J., and Militelli, M. I. (2018). Maternal condition, fecundity and oocyte quality of argentine hake (Merluccius hubbsi) from the Northern stock. Fisheries Research, 197.

Rodríguez, J. M., Alemany, F., and Garcia, S. (2017). A guide to the eggs and larvae of 100 common western mediterranean sea bony fish species. Technical report, FAO.

Romo-Curiel, A. E., Herzka, S. Z., Sosa-Nishizaki, O., Sepulveda, C. A., and Aalbers, S. A. (2015). Otolith-based growth estimates and insights into populationstructure of white seabass, Atractoscion nobilis, off the Pacific coast of North America. Fisheries Research, 161:374–383.

Rosen, R. S. (1976). Distribution, Age, and Growth, and Feeding Ecology of Paddlefish (Polyodon spathula) In Unaltered Missouri River, South Dakota. PhD thesis, South Dakota Stat University.

Rosenfield, J. and Sandoval-Green, C. (2004). The role of hybrid vigor in the replacement of pecos pupfish by its hybrids with sheepshead minnow. Conservation Biology, 18.6:1589–1598.

Ruble, C. L., Rakes, P. L., Shute, J. R., and Welsh, S. A. (2016). Captive propagation, reproductive biology, and early life history of Etheostoma wapiti (boulder darter), E. vulneratum (wounded darter), and E. maculatum (spotted darter). SOUTHEASTERN NATURALIST, 15:115–126.

Ruiz-Domínguez, M. and Quinonez-Velázquez, C. (2018). Age, growth, and mortality of Opisthonema libertate on the coasts of northwestern Mexico. Ciencias Marinas, 44:235–250.

Ruple, D. L., McMichael, R. H., and Baker, J. A. (1984). Life history of the gulf darter, Etheostoma swaini (Pisces: Percidae). Environmental Biology of Fishes, 11(2):121–130.

Ruyet, J. P.-L., Mahe, K., Bayon, N. L., and Delliou, H. L. (2004). Effects of temperature on growth and metabolism in a mediterranean population of European sea bass, Dicentrarchus labrax. Aquaculture, 237:269–280.

Ruzycki, J. R., Wurtsbaugh, W. A., and Lay, C. (1998). Reproductive ecology and early life history of the lacustrine sculpin, Cottus extensus (Teleostei, Cottidae. Envir. Biol. Fishes, 53:117–127.

Ryan, P. M. and Harvey, H. H. (1971). Growth of rock bass, Ambloplites rupestris, in relation to the morphoedaphic index as an indicator of an environmental stress. J. Fish. Res. Board Can., 34:2079–2088.

Ryan, P. M. and Harvey, H. H. (1980). Growth responses of yellow perch, Perca flauescens (Mitchill), to lake acidification in the La Clothe Mountain Lakes of Ontario. Env. Biol. Fish., 5(2):97–108.

Ryon, M. G. (1986). The life history and ecology of Etheostoma trisella (Pisces: Percidae). The American Midland Naturalist, 115:73–86.

S.-P. Wang, C.-H. L. and Chiang, W.-C. (2010). Age and growth analysis of swordfish (Xiphias gladius) in the Indian Ocean based on the specimens collected by Taiwanese observer program. IOTC-2010-WPB-08.

S.-P. Wang, C.-L. S. and Yeh, S.-Z. (2003). Sex ratios and sexual maturity of swordfish (Xiphias gladius L.) in the waters of Taiwan. Zoological Studies, 42:529–539.

S.A., E. (2020). Age and growth of iridescent toothcarp Aphanius mento (Heckel, 1843) (Cyprinodontidae) in Seyhan Reservoir (Southeastern Mediterranean, Turkey). Iranian Journal of Fisheries Sciences, 19(5):2340–2353.

Saavedra, M., Masdeu, M., Hale, P., Sibbons, C. M., and Holt, W. V. (2014). Dietary fatty acid enrichment increases egg size and quality of yellow seahorse Hippocampus kuda. Animal Reproduction Science, 69:54–61.

Saborido-Rey, F., Domínguez-Petit, R., Garabana, D., and Sigurosson, P. (2015). Fecundity of Sebastes mentella and Sebastes norvegicus in the Irminger sea and Icelandic waters. Ciencias Marinas, 41:107–124.

Saika, A. K., Singh, A. S. K., and Biswas, S. P. (2013). Reproductive biology of Channa punctatus (Bloch) from paddy field of Sivasagar, Assam. Int. J. Curr. Res., 5(3):542–546.

Sakakura, Y. and Noakes, D. L. G. (2000). Age, growth, and sexual development in the self-fertilizing hermaphroditic fish Rivulus marmoratus. Environmental Biology of Fishes, 59:309–317.

Sakaris, P. C. and Irwin, E. R. (2008). Validation of daily ring deposition in the otoliths of age-0 channel catfish. North American Journal of Fisheries Management, 28:212–218.

Sanchez-Camara, J., Booth, D. J., and Turon, X. (2005). Reproductive cycle and growth of Phyllopteryx taeniolatus. Journal of Fish Biology, 67:133–148.

Sánchez-Iturbe, A., Flores-Coto, C., and Zavala-García, F. (2006). Seasonal variation in growth and mortality of larval Syacium gunteri (Piscis: Paralichthyidae) in the southern Gulf of Mexico (1992 and 1993). Ciencias Marinas, 32(1B):83–95.

Santos, P. (1995). Growth, mortality and maturation of Lepidorhombus boscii in Portuguese waters. Technical report, ICES Demersal Fish Committee, C.M.

Santos-Martínex, A. and Ardboleda, S. (1993). Aspectos biologicos y ecologicos del macabi Elops saurus Linnaeus (Pisces: Elopidae) en la Cienaga Grande de Santa Marta y Costa Adyacente, Caribe Colombiano. An. Inst. Invest. Mar. Punta Betin, 22:77–96.

Sarkar, U. K., Deepak, P. K., Negi, R. S., Singh, S., and Kapoor, D. (2006). Captive breeding of endangered fish Chitala chitala (Hamilton-Buchanan) for species conservation and sustainable utilization. Biodiversity & Conservation, 15:3579–3589.

Sarkar, U. K., Negi, R. S., Deepak, P. K., Lakra, W. S., and Paul, S. K. (2008). Biological parameters of the endangered fish Chitala chitala (Osteoglossiformes: Notopteridae) from some Indian rivers. Fisheries Research, 90:170–177.

Sarmiento-Lezcano, A.-N., Triay-Portella, R., Castro, J. J., Rubio-Rodríguez, U., and Pajuelo, J. G. (2018). Age-based life-history parameters of the mesopelagic fish Notoscopelus resplendens (Richardson, 1845) in the Central Eastern Atlantic. Fisheries Research, 204.

Savinykh, V. F. and Baytalyuk, A. A. (2010). New data on biology of the pearlfish Maurolicus imperatorius (Sternopthychidae) from the Emperor Seamount Chain. Journal of Ichthyology, 50.

Savoy, T. F. and Crecco, V. A. (2004). American shad early life history and recruitment in the Connecticut River: A 40-year summary. American Fisheries Society Monograph, 9:407–417.

Sawyer, P. J. (1967). Intertidal life-history of the rock gunnel, Pholis gunnellus, in the Western Atlantic. Copeia, 1967:55–61.

Sayer, M. D. J., Gibson, R. N., and Atkinson, R. J. A. (1995). Growth, diet and condition of goldsinny on the west coast of Scotland. Journal of Fish Biology, 46:317–340.

Sayer, M. D. J., Gibson, R. N., and Atkinson, R. J. A. (1996). Growth, diet and condition of corkwing wrasse and rock cook on the west coast of Scotland. Journal of Fish Biology, 49:76–94.

Scalet, C. G. (1971). Life History of the Orangebelly Darter Etheostoma radiosum cyanorum (Osteichthyes, Percidae. PhD thesis, Univ. Oklahoma.

Scarnecchia, D. L. and Bergersen, E. P. (1986). Production and habitat of threatened greenback and Colorado River cutthroat trouts in Rocky Mountain headwater streams. Transactions of the american fisheries society, 115(3):382–391.

Schaeffer, J. S. (2004). Population dynmaics of bloaters Coregonus hoyi in Lake Huron, 1980-1998. Ann. Zool. Fennic., 41:271–279.

Schaner, E. and Sherman, K. (1960). Observations on the fecundity of the tomcod, Microgadus tomcod (Walbaum). Copeia, 1960(4).

Scheerer, P., Jacobs, S., Terwilliger, M., Miller, S., Gunckel, S., Richardson1, S., and Heck, M. (2011). Status, distribution, and life history investigations of Warner suckers, 2006-2010. Technical report, FISH DIVISION Oregon Department of Fish and Wildlife.

Scheerer, P. D. and McDonald, P. J. (2003). Age, growth, and timing of spawning of an endangered minnow, the Oregon chub (Oregonichthys crameri), in the Willamette Basin, Oregon. Northwestern Naturalist, pages 68–79.

Scheibly, J. F. (2003). Life history of the northern madtom, Noturus stigmosus (Siluriformes: Ictaluridae), in the Licking River, Kentucky. PhD thesis, Morehead State University.

Schilling, T. F. (2002). The morphology of larval and adult zebrafish. In Nusslein-Volhard, C. and Dahm, R., editors, Zebrafish: a practical guide, pages 59–83. Oxford University Press Inc., New York.

Schmidt, J. (1921). New studies of sun-fishes made during the "dana" expedition. Nature, 107:76–79.

Schnitzler, J., Dussenne, M., Frédérich, B., and Das, K. (2017). Post-embryonic development of sheepshead minnow Cyprinodon variegatus: a staging tool based on externally visible anatomical traits. Ichthyological Research, 64:29–36.

Schoenherr, A. A. (1988). A review of the life hostory and status of the desert pupfish, Cyprinodon macularius. Bull. Southern California Acad. Sci., 87(3):104–134.

Schönweger, G., Schwerte, T., and Pelster, B. (2000). Temperature-dependent development of cardiac activity in unrestrained larvae of the minnow Phoxinus phoxinus. American Journal of Physiology-Regulatory, Integrative and Comparative Physiology, 279:R1634–R1640.

Schoone, C. H. and van Breugel, M. (2006). Kennisdocument kolblei, Abramis (of Blicca) bjoerkna (Linnaeus, 1758). Technical report, Sportvisserij Nederland.

Schranka, S. J. and Guy, C. S. (2002). Age, growth, and gonadal characteristics of adult bighead carp, Hypophthalmichthys nobilis, in the lower Missouri River. Environmental Biology of Fishes, 64:443–450.

Schwartz, F. J. (1961). Food, age, growth, and morphology of the blackbanded sunfish, Enneacanthus c. chaetodon, in Smithville Pond, Maryland. Chesapeake Science, 2(1/2):82–88.

Schwarz, F. J. (1965). Age, growth, and egg complement of the stickleback Apeltes quadracus at Solomons, Maryland. Chesapeake Sci., 6(2):116–118.

Scoppettone, C. G., Burge, H. L., and Tuttle, P. L. (1992). Life history, abundance, and distribution of moapa dace (Moapa coriacea). Great Basin Naturalist, 52:216–225.

Scoppettone, G. G. (1988). Growth and longevity of the cui-ui and longevity of other catostomids and cyprinids in western North America. Transactions of the American Fisheries society, 117:301–307.

Scoppettone, G. G., Harvey, J. E., and Heinrich, J. (2004). Conservation, status, and life history of the endangered white riverspinedace, Lepidomeda albivallis (Cyprinidae). Western North American Naturalist, 64(1):38–44.

Scott, J. H. and Mayden, R. L. (2008). Life history of the tricolor shiner, Cyprinella trichroistia (Teleostei: Cyprinidae), in Alabama. Southeastern Naturalist, 7(1):69–80.

Scott, R. N. (1952). The time of spawning age and secondary sex character of adult chubs (Mylocheilus caurinum) from Flathead Lake Montana as correlated with histological changes in their testes. PhD thesis, Univ. of Montana.

Serchuk, F. M. and Cole, C. F. (1974). Age and growth of the cunner, Tautogolabrus adspersus (Walbaum) (Pisces: Labridae)in the Weweantic River estuary, Massachusetts. Chesapeake Science, 15(4).

Settles, W. (1974). Some Aspects of the Life History of the Southern Redbelly Dace, Chrosomus Erythrogaster Rafinesque, in Ivy Creek, Warren County, Kentucky. PhD thesis, Western Kentucky University.

Seyboth, E., Condini, M. V., Albuquerque, C. Q., Varela, A. S., Velasco, G., Vieira, J. P., and Garcia, A. M. (2011). Age, growth, and reproductive aspects of the dusky grouper Mycteroperca marginata (Actinopterygii: Epinephelidae) in a man-made rocky habitat in southern Brazil. Neotropical Ichthyology, 9:849–856.

Sezen, S. and Olmez, M. (2010). Production of Aphanius mento (Heckel, 1843) under controlled conditions. A.A.C.L. Bioflux, 5:94–98.

Shaw, M., Diekman, R., van der Kooij, J., Milligan, S., Bromley, P., and Righton, D. (2008). T assessment of the diets of cod Gadus morhus and whiting Merlangius merlangus juveniles in a frontal region close to the Norwefgiena Trench: co-existence of competition? Journal of Fish Biology, 73.

Sheaves, M. J., Molony, B. W., and Tobin, A. J. (1999). Spawning migrations and local movements of a tropical sparid fish. Marine Biology, 133(1):123–128.

Shelbourn, J. E., Clarke, W. C., and Levings, C. D. (1995). Effects of lowered temperature on growth of juvenile Nechako River chinook salmon (Oncorhynchus tshawytscha) at three ration levels. Technical report, Canadian Data Report of Fisheries and Aquatic Sciences.

Shemonaev, E. V. and Kirilenko, E. V. (2009). Some features of biology of the round goby Neogobius melanostomus (Perciformes, Gobiidae) in waters of Kuibyshev Reservoir. Journal of Ichthyology, 49:454–459.

Shepherd, M. E. and Huish, M. T. (1978). Age, growth, and diet of the pirate perch in a coastal plain stream of North Carolina. Transactions of the American Fisheries Society, 107:457–459.

Shestakov, A. V. (2017). Biology of round whitefish Prosopium cylindraceum (Coregonidae) of the Anadyr River. Journal of Ichthyology, 57:257–264.

Shestakov, A. V. and Nazarkin, M. V. (2006). On the biology of the white-spotted greenling Hexagrammos stelleri and the masked greenling H. octogrammus (Hexagrammidae) from Taui Bay of the Sea of Okhotsk. Journal of Ichthyology, 46:677–680.

Shevelev, M. S. and Johannesen, E. (2011). Wolfish. In Jakobsen, T. and Ozhigin, V. K., editors, Wolfish, chapter 5.10, pages 329–337. The Barents Sea. Ecosystem, Resources, Managment. Half a century of Russian-Norwegian cooperation.

Shi, D., Zhang, K., Cai, Y., Geng, P., Xu, Y., Sun, M., and Chen, Z. (2020). Population structure of Trichiurus japonicus in northern China Sea and parameters and its growth, mortality and maturatty. South China Fisheries Science, 16:51–59.

Shima, J. S. and Swearer, S. E. (2009). Larval quality is shaped by matrix efects: implications of connectivity in a marine metapopulation. Ecology, 90(5):1255–1267.

Shinozaki-Mends, R., Hazin, F. H. V., de Oliviera, P. G., and de Carvalho, F. C. (2007). Reproductive biology of the squirrelfish, Holocentrus adscensionis (Osbeck, 1765), caught off the coast of Pernambuco, Brazil. Scientia Marina, Barcelona (Spain), 71(4):715–722.

Shirafuji, N., Watanabe, Y., Takeda, Y., and Kawamura, T. (2007). Maturation and spawning of Spratelloides gracilis Clupeidae in temperate waters off Cape Shionomisaki, central Japan. Fisheries Science, 73:623–632.

Shui, B. (2000). Study on the reproductive ability of Larimichthys polyactis and its change. Journal of Zejiang Ocean University, 19:58–69.

Shute, P. W., Shute, J. R., and Lindquist, D. G. (1982). Age, growth and early life history of the waccamaw darter, Etheostoma perlongum. Copeia, 1982(3):561–567.

Siddique, J. C., Psenicka, M., and Linhart, O. (2014). A review of the structure of sturgeon egg membrances and of the associated terminology. Applied Ichthyology, pages 1246–1255.

Silberschneider, V., Gray, C. A., and Stewart, J. (2009). Age, growth, maturity and the overfishing of the iconic sciaenid, Argyrosomus japonicus, in south-eastern, Australia. Fisheries Research, 95:220–229.

Silva, A., Carrera, P., Massé, J., Uriarte, A. D., Santos, M. B., Oliveira, P. B., Soares, E., Porteiro, C., and Stratoudakis, Y. (2008). Geographic variability of sardine growth across the northeastern Atlantic and the Mediterranean Sea. Fisheries Research, 90:56–69.

Silva, A., Santos, M. B., Caneco, B., Pestana, G., Porteiro, C., Carrera, P., and Stratoudakis, Y. (2006). Temporal and geographic variability of sardine maturity at length in the north-eastern Atlantic and the western Mediterranean. ICES Journal of Marine Science, 63:663–676.

Silva, M. N. and Gordo, L. S. (1997). Age, growth and reproduction of the black goby, Gobius niger, from Obidos Lagoon, Portugal. Cah. Biol. Mar., 38:175–180.

Simmons, B. R. and Beckman, D. (2012). Age determination, growth, and population structure of the striped shiner and duskystripe shiner. Transactions of the American Fisheries Society, 141:846–854.

Simmons, C. M. (2008). Gray triggerfish Balistes capriscus, reproductive behaviour, early life history, and competitive iteractions between red snapper, Lutjanus campechanus, in the nothern Gulf of Mexico. PhD thesis, Auburn University.

Simon, T. P. and Wallus, R. (2003). Reproductive Biology and Early Life History of Fishes in the Ohio ..., Volume 3, volume 3. CRC Press.

Simon, V., Elleboode, R., Mahé, K., Legendre, L., Ornelas?Garcia, P., Espinasa, L., and Rétaux, S. (2017). Comparing growth in surface and cave morphs of the species Astyanax mexicanus: insights from scales. EvoDevo, 8:23.

Singh, D. and Sharrna, R. C. (1995). Age and growth of a Himalayan teleost Schizothorax richardsonii (Gray) from the Garhwal Hills (India). Fisheries Research, 24:321–329.

Sion, L., Maiorano, P., Carlucci, R., Capezzuto, F., Indennidate, A., Tursi, A., and D'Onghia, G. (2012). Review of the literature on age and growth of grenadiers in the Mediterranean and new data on age of Trachyrincus scabrus (Macrouridae) in the Ionian Sea. Journal of Ichthyology, 52(10):740–749.

Skjærven, K. H., Finn, R. N., Kryvi, H., and Fyhn, H. J. (2003). Yolk resorption in developing plaice (Pleuronectes platessa. In Browman, H. I. and Skifsrevik, A. B., editors, The Big Fish Bang., page 193. Institute of Marine Research, Bergen Norway.

Slater, S. B. (2005). Life history and diet of the Shokihaze goby Tridentiger barbatus in the San Francisco estuary. PhD thesis, California State University, Sacramento.

Smederevac-Lalić, M. M., Skorić, S. B., Višnjić-Jeftić, Z. V., Djikanović, V. D., and Mićković, B. M. (2015). Growth and weight-length relationship of Burbot Lota lota (L.) (Lotidae) in the Danube River at Bačka Palanka (Serbia). Acta zool. bulg., 67.

Smietana, P (1992). Assessment of the length growth rate of five-bearded rockling [Ciliata mustela L.] of the Wadden Sea origin. Acta Ichthyologica et Piscatoria, 1.

Smith, C. and Reichard, M. (2013). A sperm competition model for the European bitterling (Rhodeus amarus). Behaviour, 150:1709–1730.

Smith, C. L., Tyler, J. C., and Feinberg, M. N. (1981). Population ecology and biology of the pearlfish (Carapus bermudensis) in the lagoon at Bimini, Bahamas. Bull. Mar. Sci., 31:876–902.

Smith, J. L. B. and Smith, M. M. (1986). Sparidae. In Smith, M. M. and Heemstra, P. C., editors, Smiths sea fishes, pages 580–594. Springer-Verlag, Berlin.

Smith, N. G., JOones, C. M., and van Montfrans, J. (2008). Spatial and temporal variability of juvenile spotted seatrout Cynoscion nebulosus growth in Chesapeake Bay. Journal of Fish Biology, 73:597–607.

Snyder, D. J. and Peterson, M. S. (1999). Life history of a peripheral population of bluespotted sunfish Enneacanthus gloriosus (Holbrook), with comments on geographic variation. The American Midland Naturalist, 141(2):345–357.

Sohoni, P., Tyler, C., Hurd, K., Caunter, J., Hetheridge, M., Williams, T., Woods, C., Evans, M., Toy, R., Gargas, M., and Sumpter, J. (2001). Reproductive effects of long-term exposure to bisphenol a in the fathead minnow (Pimephales promelas). Environ Sci Technol, 35(14):2917–2925.

Sossamon, M. K. (1990). The Life History of the Flame Chub, Hemitremia flammea (Jordan and Gilbert), in Pond Creek, Loudon County, Tennessee. PhD thesis, University of Tennessee, Knoxville.

Spafford, M. D. (1999). Trout-perch Percopsis omiscomaycus (Walbaum) and Lake Chub Couesius plumbeus (Agassiz) as Sentinel Monotoring Species in the Athabasca River, Alberta. PhD thesis, Univ of Alberta.

Spence, R., Gerlach, G., Lawrence, C., and Smith, C. (2008). The behaviour and ecology of the zebrafish, Danio rerio. Biol. Rev., 83:13–34.

Spencer, H. B., Hussein, W. R., and Tchounwou, P. B. (2006). Growth inhibition in Japanese medaka(Oryzias latipes) fish exposed to tetrachloroethylene. Journal of Environmental Biology, 27:1–5.

Sponaugle, S., Denit, K. L., Luthy, S. A., Serafy, J. E., and Cowen, R. K. (2005). Growth variation in larval Makaira nigricans. Journal of Fish Biology, 66:822–835.

Stalmans, J. and Kestemont, P. (1991). Production de juveniles de vairon Phoxinus phoxinus L. à partir de larves obtenues en conditions controlées. Bulletin francais de la pêche et de la pisciculture, 320:29–37.

Stanley, R. D. (1972). The effect of egg size on size and viability of newly hatched medaka (Oryzias latipes) and surf smelt (Hypomesus pretiosus pretiosus). PhD thesis, Univ. of British Columbia.

Stark, J. W. (2004). A comparison of the maturation and growth of female flathead sole in the central Gulf of Alaska and south-eastern Bering Sea. Journal of Fish Biology, 64:876–889.

Stark, J. W. and Somerton, D. A. (2002). Maturation, spawning and growth of rock soles off Kodiak Island in the Gulf of Alaska. Journal of Fish Biology, 61:417–431.

Starks, T. A., Miller, M. L., and Long, J. M. (2016). Early life history of three pelagic-spawning minnows Macrhybopsis spp. in the lower Missouri River. Journal of Fish Biology, 88:1335–1349.

Starnes, L. B. and Starnes, W. C. (1985). Ecology and life history of the mountain madtom, noturus eleutherus (pisces: Ictaluridae). The American Midland Naturalist, 114:331–341.

Starnes, W. C. (1977). The Ecology and Life History of the Endangered Snail Darter, Percina (Imostoma) tanasi Etnier. PhD thesis, University of Tennessee, Knoxville.

Stasiak, R. H. (1978). Reproduction, age, and growth of the finescale dace, Chrosomus neogaeus, in Minnesota. Transactions of the American Fisheries Society, 107(5):720–723.

Stequert, B., Menard, F., and Marchal, E. (2003). Reproductive biology of Vinciguerria nimbaria in the equatorial waters of the eastern Atlantic Ocean. Journal of Fish Biology, 62.

Stergiou, K. I., Economidis, P., and Sinis, A. (1992). Age, growth and mortality of red bandfish, Cepola macrophthalma (L.), in the western Aegean Sea (Greece). Journal of Fish Biology, 40:395–418.

Stewart, D., Reist, J., Carmichael, T., Sawatzky, C., and Mochnacz, N. (2007). Fish life history and habitat use in the Northwest Territories: brook stickleback (Culaea inconstans). Technical report, Canadian Manuscript Report of Fisheries and Aquatic Sciences.

Stewart, J. and Hughes, J. M. (2007). Age validation and growth of three commercially important hemiramphids in south-eastern Australia. Journal of Fish Biology, 70:65–82.

Stewart, T. R., Ogle, D. H., Gorman, O. T., and Vinson, M. R. (2016). Age, growth, and size of lake superior pygmy whitefish (Prosopium coulterii). The American Midland Naturalist, 175:24–36.

Steyn, G. J., Gagiano, C. L., Deacon, A. R., and du Preez, H. H. (1996). Notes on the induced reproduction and development of the tigerfish, Hydrocynus vittatus (Characidae), embryos and larvae. Environmental Biology of Fishes, 47:387–398.

Stockwell, J. D., Ebener, M. P., Black, J. A., Gorman, O. T., Hrabik, T. R., ronald E. Kinnunen, Mattes, W. P., Oyadomari, J. K., Schram, S. T., Schreiner, D. R., Sitar, S. P., and Yule,

D. L. (2009). A synthesis of cisco recovery in Lake Superior: Implications for native fish rehabilitation in the Laurentian Great Lakes. North American Journal of Fisheries Management, 29:626–652.

Strelcheck, A. J., Fitzhugh, G. R., Colemana, F. C., and Koenig, C. C. (2003). Otolith-fish size relationship in juvenile gag (Mycteroperca microlepis) of the eastern Gulf of Mexico: a comparison of growth rates between laboratory and field populations. Fisheries Research, 60:255–265.

Sule, M. J. and Skelly, T. M. (1985). The life history of the shorthead redhorse, Moxostoma macrolepidotun in the Kankakee River Drainage, Illinois, in the James River, Missouri. Technical report, State of Illinois, Dept of Energy and Nature Resources; Nat. His Survey Division.

Sumagaysay, N. S. and Borlongan, I. G. (1995). Growth and production of milkfish (Chanos chanos) in brackishwater ponds: effects of dietary protein and feeding levels. Aquaculture, 132:273–283.

Summerfelt, R. C. and Minckley, C. O. (1969). Aspects of the life history of the sand shiner, Notropis stramineus (Cope), in the Smoky Hill River, Kansas. Transactions of the American Fisheries Society, 98(3):444–453.

Sumpter, J. P., le Bail, P. Y., Pickering, A. D., Pottinger, T. G., and Carragher, J. (1991). The effect of starvation on growth and plasma growth hormone concentrations of rainbow trout, Oncorhynchus mykiss. General and Comparative Endocrinology, 83:94–102.

Sun, C.-L., Chang, H.-Y., Liu, T.-Y., Yeh, S.-Z., and Chang, Y.-J. (2015a). Reproductive biology of the black marlin, Istiompax indica, offsouthwestern and eastern Taiwan. Fisheries Research, 166:12–20.

Sun, C.-L., Su, N.-J., Yeh, S.-Z., and Chang, Y.-J. (2013). Sex-specific growth parameters and natural mortalilty rates for blue marlin (Makaira nigricans) in the Northwest Pacific Ocean. Technical report, Working document submitted to the ISC Billfish Working Group Workshop, 16-23 January 2013, Honolulu, Hawaii, USA.

Sun, C.-L., Yeh, S.-Z., Liu, C.-S., Su, N.-J., and Chiang, W.-C. (2015b). Age and growth of black marlin (Istiompax indica) off eastern Taiwan. Fisheries Research, 166:4–11.

Susatyo, P., Setyaningrum, N., Winarni, E. T., and Titi Chasanah, A. (2018). Reproduction characteristics of rice field eel (Monopterus albus Zuieuw) on several functionally changed lands in Banyumas Regency. The Journal of Tropical Life Science, 8(2):177–186.

Sutton, T. M., Grier, A. C., and Frankland, L. D. (2009). Stock structure and dynamics of longnose gar and shortnose gar in the Wabash River, Indiana-Illinois. Journal of Freshwater Ecology, 24:657–666.

Swartz, R. C. and Engel, W. A. v. (1968). Length, weight, and girth relations in the toadfish, Opsanus tau. Chesapeake Sci., 9(4):249–253.

Sweetman, P. C., Haddy, J. A., and Robertson, S. (2018). Multi-decadal variation in cohort specific sex ratios and otolith increment growth characteristics of juvenile blue grenadier (Macruronus novaezelandiae). Fisheries Research, 201.

Swingle, H. S. and Smith, E. V. (1943). Factors affecting the reproduction of bluegill bream and largemouth black bass in ponds. Agricultural Experiment Station of the Alabama Polytechnic Institute, 8:67–74.

Szczepkowski, M., Szczepkowska, B., Krzywosz, T., Wunderlich, K., and Stabinsk, R. (2010). Growth rate and reproduction of a brood stock of European whitefish (Coregonus lavaretus L.) from Lake Galadus under controlled rearing conditions. Arch. Pol. Fish., 18:3–11.

Taber, C. A. and Taber, B. A. (1983). Reproductive biology and age and growth of the Missouri saddled darter Etheostoma tetrazonum. The American Midland Naturalist, 109(2):222–229.

Taber, C. A., Taber, B. A., and Topping, M. S. (1986). Population structure, growth and reproduction of the Arkansas darter, Etheostoma cragini(Percidae). The Southwestern Naturalist, 31(2):207–214.

Takagi, K., Yatsu, A., Moku, M., and Sassa, C. (2006). Age and growth of lanternfishes, Symbolophorus californiensis and Ceratoscopelus warmingii (Myctophidae), in the Kuroshio-Oyashio transition zone.

Takahashi, M., Yoneda, M., Kitano, H., Kawabata, A., and Saito, M. (2005). Growth of juvenile chub mackerel Scomber japonicus in the western North Pacific Ocean: with application and validation of otolith daily increment formation. Journal of Fish Biology, 67:1619–1630.

Takegaki, T. (2008). Threatened fishes of the world: Boleophthalmus pectinirostris (Linnaeus 1758) (Gobiidae). Environmental Biology of Fishes, 81:373–374.

Tanner, D. and Moffett, M. (1995). Effects of diflubenzuron on the reproduction success of the bluegill sunfish, lepomis macrochirus. Environmetal Toxicology and Chemistry, 14:1345–1355.

Taylor, C. M. and Miller, R. J. (1990). Reproductive ecology and population structure of the plains minnow, Hybognathus placitus (Pisces: Cyprinidae), in Central Oklahoma. American Midland Naturalist, 123(1):32–39.

Taylor, R. and Willis, T. (1998). Relationships amongst length, weight and growth of north-eastern New Zealand reef fishes. Marine and freshwater Research, 49:255–260.

Teal, L. (2003). IMARES data base.

Teh, S. J. and Hinton, D. E. (1998). Gender-specific growth and hepatic neoplasia in medaka (Oryzias latipes). Aquatic Toxicology, 41:141–159.

Teixeira, C. M. and Cabral, H. N. (2010). Comparative analysis of the diet, growth and reproduction of the soles, *Solea solea* and *Solea senegalensis*, occurring in sympatry along the Portuguese coast. Journal of the Marine Biological Association of the United Kingdom, 90(5):995–1003.

Terwilliger, M. R., Reece, T., and Markle, D. F. (2010). Historic and recent age structure and growth of endangered Lost River and shortnose suckers in Upper Klamath Lake, Oregon. Environ Biol Fish, 89:239–252.

Tesch, F. (2003). The Eel. Blackwell, Oxford. p. 173 Table 3.10 last row.

Thompson, B. W. (2003). An Ecological/Life History Comparison of Two Whitefish Species in Bear Lake, Utah/Idaho. PhD thesis, Utah State University.

Thompson, D., Hargrave, S., Morgan, G., and Powers, S. L. (2017). Life-history aspects of Chrosomus oreas (mountain redbelly dace) in Catawba Creek, Virginia. Technical report.

Thompson, D. A., Bentley, J. S., and Powers, S. L. (2015). Life-history aspects of Moxostoma cervinum (blacktip jumprock) in the Roanoke river, Virginia. Virginia Journal of Science, 66(4):391–401.

Thorpe, J., Miles, M., and Keay, D. (1984a). Developmental rate, fecundity and egg size in Atlantic salmon, Salmo salar L. Aquaculture, 43(1-3):Figure 1, page 294, extracted using WebPlotDigitizer.

Thorpe, J., Miles, M., and Keay, D. (1984b). Developmental rate, fecundity and egg size in Atlantic salmon, Salmo salar L. Aquaculture, 43(1-3):Figure 1, page 294, extracted using WebPlotDigitizer.

Thorsen, A., Witthames, P. R., Marteinsdøttir, G., Nash, R. D. M., and Kjesbu, O. S. (2010). Fecundity and growth of Atlantic cod (Gadus morhua L.) along a latitudinal gradient. Fisheries Research, 104:45–55.

Thorsteinsson, V. (1983). Some aspects of the biology and the fisheries of the Lumpfish (Cyclopterus lumpus). PhD thesis, State University of New York at Stony Brook.

Thresher, R. E. and Brothers, E. B. (1985). Reproductive ecology and biogeography of indo-west pacific angelfishes (pisces: Pomacanthidae). Evolution, 39:878–887.

Ticina, V. and Matic-Skoko, S. (2012). Age, growth and mortality of scaldfish (Arnoglossus laterna Walbaum, 1792) from the Adriatic Sea. J. Appl. Ichthyol., 28.

Timmons, T. J., Ramsey, J. S., and Bauer, B. H. (1983). Life history and habitat of the blackfin sucker, Moxostoma atripinne (Osteichthyes: Catostomidae). Copeia, 1983(2):538–541.

Tojeira, I., Faria, A. M., Henriques, S., Faria, C., and Gonçalves, E. J. (2012). Early development and larval behaviour of two clingfishes, Lepadogaster purpurea and Lepadogaster lepadogaster (Pisces: Gobiesocidae). Environ Biol Fish, 93:449–459.

Tolmacheva, Y. P. and Bogdanov, B. E. (2010). Special traits of growth and feeding of the stone sculpin Paracottus knerii (Cottidae) in water bodies of different types. Journal of Ichthyology, 50:529–535.

Tolmacheva, Y. P., Gavrilova, A. V., Bogdanov, B. E., Dzyuba, E. V., Veinberg, I. V., Rozhkova, N. A., Maksimova, N. V., and Zubina, L. V. (2008). Seasonal dynamics of growth and feeding of big-headed sculpin Batrachocottus baicalensis (Cottidae) in the Cape Berezovyi Area (Southern Baikal). Journal of Ichthyology, 48:241–248.

Tolonen, A. (1997). Size-specific food selection and growth in benthic whitefish, Coregonus lavaretus (L.), in a subarctic lake. Boreal Environment Research, 2:387–399.

Torstensen, E. (1992). Fecundity studies on sprat, Sprattus sprattus L., from a fjord on the Norwegian Skagerrak coast. Technical report, Flodevigen rapportser 1.

Traczyk, R. J. (2015). Age, growth and distribution of the Antarctic fish Chaenocephalus aceratus based on otoliths. Journal of Environmental Science and Engineering, A 4:401–419.

Travis, J., Farr, J. A., McManus, M., and Trexler, J. C. (1989). Environmental effects on adult growth patterns in the male sailfin molly, Poecilia latipinna (Poeciliidae). Environmental Biology of Fishes, 26:119–127.

Tsai, C.-F. (1972). Life history of the eastern Johnny darter, Etheostoma olmstedi Storer, in cold tailwater and sewage-polluted water. Transactions of the American Fisheries Society, 101(1):80–88.

Tsangridis, A. and Filippousis, N. (1994). Analysis of two models for picarel (Spicara smaris L.) growth using Schnute's micro-simplex nonlinear estimation procedure. Fisheries Research, 20:181–189.

Tserpes, G. and Tsimenides, N. (2001). Age, growth and mortality of Serranus cabrilla (Linnaeus, 1758) on the Cretan shelf. Fisheries Research, 51:27–34.

Tsikliras, A. C. and Antonopoulou, E. (2006). Reproductive biology of round sardinella (Sardinella aurita) in the north-eastern Mediterranean. Scientia Marina, 70:281–290.

Tucker, J. W. (1998). Marine Fish Culture. Springer-Science+Business Media.

Tumilson, R. and Hardage, J. O. (2014). Growth and reproduction in the ouachita madtom (Noturus lachneri) at the periphery of its distribution. Journal of the Arkansas Academy of Science, 68:Article 19.

Türkmen, M., Erdogan, O., Yildirim, A., and Akyurt, I. (2002). Reproduction tactics, age and growth of Capoeta capoeta umbla Heckel 1843 from the aşkale Region of the Karasu River, Turkey. Fisheries Research, 54:317–328.

Turner, J. S. and Snelson, F. F. (1984). Population structure, reproduction and laboratory behavior of the introduced Belonesox belizanus (Poeciliidae) in Florida. Environmental Biology of Fishes, 10(l/2):89–100.

Turnpenny, A. W. H., Bamber, R. N., and Henderson, P. A. (1981). Biology of the sand-smelt (Atherina presbyter Valenciennes) around Fawley power station. Journal of Fish Biology, 18:417–427.

Tyler, C. R., Pottinger, T. G., Santos, E., Sumpter, J. P., Price, S.-A., Brooks, S., and Nagler, J. J. (1996). Mechanisms controlling egg size and number in the rainbow trout, Oncorhynchus mykiss. Biology of reproduction, 54:8–15.

Tyus, H. M. and Nikirk, N. J. (1990). Abundance, growth, and diet of channel catfish, Ictalurus punctatus, in the Green and Yampa Rivers, Colorado and Utah. The Southwestern Naturalist, 35:188–198.

Urbach, D., Kang, M., Kang, S., Seong, K. B., Kim, S., Dieckmann, U., and Heino, M. (2012). Growth and maturation of Korean chum salmon under changing environmental conditions. Fisheries Research, 134-136:104–112.

Uribe, M. C., Grier, H. J., and Parenti, L. R. (2012). Ovarian structure and oogenesis of the oviparous goodeids Crenichthys baileyi (Gilbert, 1893) and Empetrichthys latos Miller, 1948 (Teleostei, Cyprinodontiformes). Journal of Morphology, 273:371–387.

Ursin, E. (1967). A mathematical model of some aspects of fish growth, respiration, and mortality. J. Fish. Res. Board Can., 24:2355–2453.

Valdez, R., Ryel, R., and Carothers, S. (2002). Colorado pikeminnow(Ptychocheilus lucius) recovery goals. Technical report, U.S. Fish and Wildlife Service Mountain-Prairie Region(6) Denver, colorado.

Valle, C., Bayle, J. T., and Ramos, A. A. (2003). Weight-length relationships for selected fish species of the western Mediterranean Sea. Journal of Applied Ichthyology, 19:261–262.

Vallis, L., MacLatchy, D. L., and Munkittrick, K. R. (2007). Assessment of the potential of the rock gunnel (Pholis gunnellus) along the Atlantic coast of Canada as a species for monitoring the reproductive impacts of contaminant exposures. Environ Monit Assess, 128:183–194.

Van Aerle, R., Runnalis, T., and Tyler, C. R. (2004). Ontogeny of gonadal sex development relative to growth in fathead minnow. Journal of fish biology, 64(2):355–369.

van der Hammen, T. (2011). IMARES Frisbe database.

van der Veer, H. (2009). (NIOZ), pers. comm.

van der Walt, B. A. and Beckley, L. E. (1997). Age and growth of Sarpa salpa (Pisces: Sparidae) off the east coast of South Africa. Fisheries Research, 31:241–248.

van Emmerik, W. A. M. (2004). Kennisdocument pos, Gymnocephalus cernuus (Linnaeus, 1758). Technical report, Sportvisserij Nederland.

van Emmerik, W. A. M. (2008). Kennisdocument brasem Abramis brama (Linnaeus, 1758). Technical report, Sportvisserij Nederland.

Van Leeuwen, T. E., McLennan, D., McKelvey, S., Stewart, D. C., Adams, C. E., and Metcalfe, N. B. (2016). The association between parental life history and offspring phenotype in Atlantic salmon. Journal of Experimental Biology, 219(3):Figures 1 and S2.

Vanicek, C. D. and Kramer, R. H. (1969). Life history of the Colorado squawfish, Ptychocheilus lucius, and the Colorado chub, Gila robusta, in the Green River in Dinosaur National Monument, 1964-1966. Transactions of the American Fisheries Society, 98(2):193–208.

Vargas-Chacoff, L., Ruiz-Jarabo, I., Pascoa, I., Goncalves, O., and Mancera, J. M. (2014). Yearly growth and metabolic changes in earthen pond-cultured meagre Argyrosomus regius. Scientia Marina, 78:193–202.

Varley, J. D. and Livesay, J. C. (1976). Utah ecology and life history of the utah chub, Gila atraria, in flaming gorge reservoir, utah-wyoming. Technical Report 76-16, Utah Division of Wildlife Resources.

Vastano, A. R., Able, K. W., Jensen, O. P., López-Duarte, P. C., Martin, C. W., and Roberts, B. J. (2017). Age validation and seasonal growth patterns of a subtropical marsh fish: The Gulf killifish, Fundulus grandis. Environ Biol Fish, 100:1315–1327.

Vdovin, A. N., Chetyrbotsky, A. N., and Boiko, M. I. (2017). Growth dynamics of the barfin plaice Liopsetta pinnifasciata (Pleuronectidae) in Peter the Great Bay (Sea of Japan). Journal of Ichthyology, 57:365–371.

Vecsei, P. and Artyukhin, E. (2001). Threatened fishes of the world: Acipenser persicus Borodin. Environmental Biology of Fishes, page 160.

Vedishcheva, E. V., Orlov, A. M., Orlov, S. Y., and Trofimova, A. O. (2016). First data on the age, growth processes, and otoliths of snub-nosed spiny eel Notacanthus chemnitzii (Notacanthidae). Journal of Ichthyology, 56:890–898.

Vega-Cendejas, M., Mexicano-Cintora, G., and Arce, A. M. (1997). Biology of the thread herring Opisthonema oglinum (Pisces: Clupeidae) from a beach seine fishery of the Campeche bank, mexico. Fisheries Research, 30:117–126.

Velasco, J. C., Rinc'on, P. A., and Lobón-Carviá (1990). The age, growth and reproduction of the cyprinid Rutilus lemmingii (Steindachner, 1866) in the River Huebra, Central Spain. Journal of Fish Biology, 36:469–480.

Velsen, F. P. J. (1987). Temperature and incubation in Pacific salmon and rainbow trout: Compilation of data on median hatching time, mortality and embryonic staging. Technical Report 626, Canadian Data Report of Fisheries and Aquatic Science.

Venkatesh, B. and Shetty, H. (1978). Studies on the growth rate of the grass carp Ctenopharyngodon idella (Valenciennes) fed on two aquatic weeds and a terrestrial grass. Aquaculture, 13:45–53.

Vetemaa, M. (1999). Reproduction biology of the viviparous blenny (Zoarces viviparus L.) in Kattegat. Fiskeriverket rapport, 1999:81–96.

Vieira, A. R., Neves, A., Sequeira, V., Paiva, R. B., and Gordo, L. S. (2014). Age and growth of forkbeard, Phycis phycis, in Portuguese continental waters. Journal of the Marine Biological Association of the United Kingdom, 94:623–630.

Vigg, S. and Kucera, P. A. (1981). Contributions to the life history of Sacramento perch, Archoplites interruptus, in Pyramid Lake, Nevada. The Great Basin Naturalist, 41(3):278–289.

Vinagre, C. (2007). Ecology of the juveniles of the soles *Solea solea* (Linnaeus, 1758) and *Solea senegalensis* (Kaup, 1858), in the Tagus estuary. PhD thesis, University of Lisbon.

Vincent, A. C. J. and Giles, B. G. (2003). Correlates of reproductive success in a wild population of Hippocampus whitei. Journal of Fish Biology, 63:344–355.

Vives, S. P. (1987). Aspects of the life history of the slender madtom Noturus exilis in Northeastern Oklahoma (Pisces: Ictaluridae). The American Midland Naturalist, 117(1):167–176.

Vondracek, B., Wurtsbaugh, W. A., and Cech, J. J. (1988). Growth and reproduction of the mosquitofish, Gambusia affinis, in relation to temperature and ration level: consequences for life history. Environmental Biology of Fishes, 21:45–57.

Žiliukien, V. and Žiliukas, V. (2010). Growth of pike Esox lucius L. in Lake Rubikiai (Lithuania). J. Appl. Ichthyol., 26:898–903.

Wakefield, C. B., Newman, S. J., Marriott, R. J., Boddington, D. K., and Fairclough, D. V. (2013). Contrasting life history characteristics of the eightbar grouper Hyporthodus octofasciatus (Pisces: Epinephelidae) over a large latitudinal range reveals spawning omission at higher latitudes. ICES Journal of Marine Science, 70:485–497.

Wakefield, C. B., Williams, A. J., Newman, S. J., Bunel, M., Boddington, D. K., Vourey, E., and Fairclough, D. V. (2015). Variations in growth, longevity and natural mortality for the protogynous hermaphroditic eightbar grouper Hyporthodus octofasciatus between the Indian and Pacific oceans. Fisheries Research, 172:26–33.

Walford, R. L. and Liu, R. K. (1965). Husbandrty, life span, and growth rate of the annual fish, Cynolebias adloffi E. Ahl. Exp. Geront., 1:161–171.

Walkusz, W., Paulic, J. E., Williams, W. J., Kwasniewski, S., and Papst, M. H. (2011). Distribution and diet of larval and juvenile Arctic cod (*Boreogadus saida*) in the shallow Canadian Beaufort Sea. Journal of Marine Systems, 84(3):78–84.

Wallus, R. and Simon, T. P. (2026). Reproductive Biology and Early Life History of Fishes in the Ohio River Drainage, volume 5. Taylor and Francis, Boca Raton.

Wallus, R., Yeager, B. L., and Simon, T. P. (1990). The reproductive biology and early life history of fishes of the Ohio river, volume 1: Acipenseridae through Esocidae. Tennessee Valley Authority.

Wan, R. and Meng, Z. (2003). The artificial insemination and hatching of Trichiurus japonicus. Journal of Fisheries of China, 27:188–192.

Wang, J. C. S. (1986). Fishes of the sacramento-san joaquin estuary and adjacent waters, california: A guide to the early life histories. Technical report, Interagency Ecological Program.

Wanshu, H. and Qiyong, Z. (2004). Induced nest spawning and artificial hatching of the fertilized eggs of mudskipper, Boleophthalmus pectinirostris. Chinese Journal of Oceanology and Limnology, 22:408–413.

Wanzenböck and Wanzenböck, S. (1993). Temperature effetcs on incubation time and growth of juvenile whitefin gudgeon, Gobio albipinnatus Lukash. Journal of Fish Biology, 42:35–46.

Warlen, S. M. (1988). Age and growth of larval gulf menhaden, Brevoortia patronus, in the northern Gulf of Mexico. Fishery Bulletin, 86:77–90.

Warren, M. L. and Brooks, M. (2014). Freshwater Fishes of North America: Volume 1: Petromyzontidae to Catostomidae. John Hopkins Univ. Press, Baltimore.

Warren, M. L. J. and Burr, B. M. (2014). Freshwater Fishes of North America: Volume 1: Petromyzontidae to Catostomidae. JHU Press, Science.

Wassef, E. A. (1991). Comparative growth studies on Lethrinus lentjan, lacépède 1802 and lethrinus mahsena, forsskål 1775 (Pisces, Lethrinidae) in the Red Sea. Fisheries Research, 11:75–92.

Watanabe, K., Jensen, K., Orlando, E., and G.T., A. (2007). What is normal? a characterization of the values and variability in reproductive endpoints of the fathead minnow, Pimephales promelas. Comparative Biochemistry and Physiology, Part C: Toxicology and Pharmacology, 146:348–356.

Watters, D. L., Kline, D. E., Coale, K. H., and Cailliet, G. M. (2006). Radiometric age confirmation and growth of a deep-water marine fish species: The bank rockfish, Sebastes rufus. Fisheries Research, 81:251–257.

Weatherley, A. H. and Gill, H. S. (1981). Recovery growth following periods of restricted rations and starvation in rainbow trout Salmo gairdneri Richardson. J. Fish Biol., 18:195–208.

Weber, L., Higgins, P., Carlson, R., and Janz, D. (2003). Development and validation of methods for measuring multiple biochemical indices of condition in juvenile fishes. Journal of fish biology, 63:637–658.

Weimans, P. A. D. M. (2007). Kennisdocument barbeel, Barbus barbus (Linnaeus, 1758). Technical report, Sportvisserij Nederland.

Weiss, E. F. (1962). The age, growth and food habits of the marine cottid Leptocottus armatus. PhD thesis, Montana State College Bozeman, Montana.

Wells, L. and House, R. (1974). Life history variation of the spottail shiner (Notropis hudsonius) in southern Lake Michigan, the Kalamazoo River, and western Lake Erie. Technical report, U.S. Dept of the Interior, fish and Wildlife Service, Bureau of Sport Fisheries and Wildlife, Washington D.C.

Welsh, D. P., Wiegmann, D. D., Angeloni, L. M., Newman, S. P., Miner, J. G., and Baylis, J. R. (2017). Condition-dependent reproductive tactics in male smallmouth bass: evidence of an inconsistent birthdate effect on early growth and age at first reproduction. Journal of Zoology, 302:244–251.

West, B. W. (1966). Growth rates at various temperatures of the orange-throat darter Etheostoma spectabile (Agassiz). Arkansas Academy of Science Proceedings, 20:50–53.

Weyl, O. L. F. and Hecht, T. (1998). The biology of Tilapia rendalli and Oreochromis mossambicus (Pisces: Cichlidae)in a subtropical lake in Mozambique. South African Journal of Zoology, 33(3):178–188.

Wheeler, A. (1978). Key to the fishes of Northern Europe. Frederick Warne Ltd., London, UK.

White, M. G., Veit, R. R., North, A. W., and Robinson, K. (1996). Egg-shell morphology of the Antarctic fish, Notothenia rossi Richardson, and the distribution and adundance of pelagic eggs at South Georgia and Notothenia rossi marmorata Fischer in Admiralty Bay (King George Island, South Shetland Islands). Antarctic Science, 8:267–271.

White, S. (2012). Distribution and life history of Chrosomus sp. cf. saylori in the Upper Clinch River Watershed, Virginia. Master's thesis, Virginia Polytechnic Institute and State University, Blacksburg, VA.

White, W. B. and Culver, D. C. (2012). Encyclopedia of caves. Elsevier.

Wienerroither, R., Johannesen, E., Dolgov, A., Byrkjedal, I., Bjelland, O., Drevetnyak, K., Eriksen, K. B., Høines, r., Langhelle, G., H., L., Prokhorova, T., Prozorkevich, D., and Wenneck, T. (2011). Atlas of the Barents Sea fishes. IMR/PINRO.

Wieser, W. (1985). Developmental and metabolic constraints of the scope for activity in young rainbow trout (Salmo Gairdneri). Journal of Experimental Biology, 118(1):133–142.

Wieser, W., Schiemer, F., Goldschmidt, A., and Kotrschal, K. (1992). Environmental biology of European cyprinids. Springer.

Wiff, R., Ojeda, V., and Quiroz, J. C. (2007). Age and growth in pink cusk-eel (Genypterus blacodes) off the Chilean austral zone: evaluating differences between management fishing zones. J. Appl. Inchthyol., 23:270–272.

Wijmans, P. A. D. M. (2009). Kennisdocument kroeskarper, Carassius carassius (Linnaeus, 1758). Technical report, Sportvisserij Nederland.

Wilhelm, M., Moloney, C., Paulus, S., and Roux, J.-P. (2017). Fast growth inferred for northern Benguela shallow-water hake Merluccius capensis using annual survey- and monthly commercial length-frequency distributions. Fisheries Research, 193.

Williot, P., Rochard, E., and Kirschbaum, F. (2009). Acceptability and prerequisites for the successful introduction of sturgeon species. In Carmona, R., Domezain, A., Garcia, G. M., Hernando-Casal, J., Rodriguez, F., and Ruiz, R. M., editors, Biology, conservation and sustainable development of sturgeons, Fish & fisheries series, pages 369–384. Springer, New York.

Winther, J. K. (1962). Age and Growth of the Longfin Dace, Agosia chrysogaster Girard. PhD thesis, University of New Mexico.

Wise, B. S., Potter, I. C., and Wallace, J. H. (1994). Growth, movements and diet of the terapontid Amniataba caudavittata in an Australian estuary. Journal of Fish Biology, 45:917–931.

Witte, H. (2009). (NIOZ), pers. comm.

Witthames, P. and Greer Walker, M. (1995). Determinacy of fecundity and oocyte atresia in sole (*Solea solea*) from the Channel, the North Sea and the Irish Sea). Aquatic Living Resources, 8:91–109.

Wong, J. M. and Benzie, J. A. H. (2003). The effects of temperature, Artemia enrichment, stocking density and light on the growth of juvenile seahorses, Hippocampus whitei (Bleeker, 1855), from Australia. Aquaculture, 228:107–121.

Woodland, R. J. (2005). Age, growth and recruitment of Hudson River shortnose sturgeon (Acipenser brevirostrum). PhD thesis, University of Maryland.

Woodward, R. L. and Wissing, T. E. (1976). Age, growth, and fecundity of the quillback (Carpiodes cyprinus) and highfin (C. velifer) carpsuckers in an Ohio Stream. Transactions of the American Fisheries Society, 105(3):411–415.

Wootton, R. J. (1973). The effect of size of food ration on egg production in the female three-spined stickleback Gasterosteus aculeatus L. J. Fish Biol., 5:89–96.

Wright, H. A., Wootton, R. J., and Barber, I. (2004). Interpopulation variation in early growth of threespine sticklebacks (Gasterosteus aculeatus) under laboratory conditions. Can. J. Fish. Aquat. Sci., 61:1832–1838.

Wu, H., Cheng, G., Zhou, J., and Wang, J. (1985). Study on the growth of the hairtails Trichiurus japonicus in northern China Sea and parameters and its growth, mortality and maturaty. South China Fisheries Science, 16:51–59.

Yan, L., Li, J., Shen, D., Yu, L., and Ling, L. (2006). Variations in diet composition and feeding intensity of small yellow croaker Larimichthys polyactis bleeker in the southern Yellow Sea and northern East China Sea. Marine Fisheries, 28:117–123.

Yan, L., Liu, Z., Ling, J., and Yuan, X. (2014). On the evolution of biological characteristics and resources of small yellow croaker. Marine Fisheries, 36:481–488.

Yang, M. S. and Xiong, B. X. (2010). Age and growth of Monopterus albus Zuiew, 1793 (Synbranchidae). J. Appl. Ichthyol., 26:488–490.

Yanik, T., Hisar, S. A., and Bölükbas, C. (2002). Early development and growth of Arctic charr (Salvelinus alpinus) and rainbow trout (Oncorhynchus mykiss) at a low water temperature. The Israeli Journal of Aquaculture, 54(2):73.

Yanwirsal, H. (2013). Reproductive styles of Osteoglossomorpha with emphasis on Notopterus notopterus and Osteoglossum bicirrhosum. PhD thesis, Humboldt-Univ Berlin.

Yilmaz, S., Yazicioglu, O., Yazici, R., and Polat, N. (2015). Age, growth and reproductive period of white bream, Blicca bjoerkna (L., 1758) in Lake Ladik, Turkey. Journal of Limnology and Freshwater Fisheries Research, 1(1):9–18.

Yoneda, M., Sakai, T., Tokimura, M., Horikawa, H., and Matsuyama, M. (2002). Age and growth of the lizardfish Saurida sp. 1 in the East China sea using otolith ring marks. Fisheries Research, 55.

Young, J. W., Bulman, C. M., Blaber, S. J. M., and Wayte, S. E. (1988). Age and growth of the lanternfish Lampanyctodes hectoris (Myctophidae) from eastern Tasmania, Australia. Marine Biology, 99.

Young, M. (2009). Greenback cutthroat trout (Oncorhynchus clarkii stomias): A technical conservation assessment.

Yoğurtçuoğlu, B. and Ekmekçi, F. G. (2012). Life-history traits of Aphanius danfordii (Boulenger, 1890) (Pisces: Cyprinodontidae), endemic to kızılırmak Basin (Turkey). J. Appl. Ichthyol., 35:1–6.

Yufera, M., Parra, G., Santiago, R., and Carrascosa, M. (1999). Growth, carbon, nitrogen and caloric content of *Solea senegalensis* (Pisces: Soleidae) from egg fertilization to metamorphosis. Marine Biology, 134(1):43–49.

Zabel, R. W. and Achord, S. (2004). Relating size of juveniles to survival within and among populations of chinook salmon. Ecology, 85:795–806.

Zanuy, S. and Carrillo, M. (1985). Annual cycles of growth, feeding rate, gross conversion efficiency and hematocrit levels of sea bass (Dicentrarchus labrax) adapted to two different osmotic media. Aquaculture, 44:11–25.

Zekeria, Z. A. (2003). Butterflyfishes of the Southern Red Sea: Ecology and population dynamics. PhD thesis, Groningen University, the Netherlands.

Zekeria, Z. A., Weertman, S., Samuel, B., Kale-ab, T., and Videler, J. J. (2006). Growth of Chaetodon larvatus (Chaetodontidae: Pisces) in the southern Red Sea. Marine Biology, 148:1113–1122.

Zervas, P. G. (2010). Age, Reproduction, Growth, Condition and Diet of the Introduced Yellow Bass, Morone mississippiensis, in Barren River Lake, Kentucky. PhD thesis, Western Kentucky Univ.

Zhan, W., Lou, B., Chen, R., Mao, G., Liu, F., Xu, D. D., Wang, L., Ma, T., and Xu, Q. (2016). Observation of embryonic, larva and juvenile development of small yellow croaker, Larimichthys polyactis. Oceanologia et Limnologia Sinica, 47:1033–1039.

Zhang, G., Li, X., Jin, X., Zhu, J., and Dai, F. (2010). Changes of biological characteristics of small yellow croaker (Larimichthys polyactis) in the central and southern Yellow Sea. Acta Ecologica Sinica, 30:6854–6861.

Zhang, Z.-M., Xie, C.-X., Ding, H.-P., Liu, C.-J., Ma, X.-F., and Ca, L.-G. (2016). Age and growth of bream Abramis brama (Linnaeus, 1758) in the downstream section of Irtysh River in China. Applied Ichtyology, 32:105–109.

Zorica, B. and Kec, V. C. (2013). Age, growth and mortality of the garfish, Belone belone (L. 1761) in the eastern Adriatic Sea. J. Mar. Biol. Assoc., 93:365–372.

Zorica, B., Sinovcic, G., and Kec, V. C. (2011). The reproductive cycle, size at maturity and fecundity of garfish (Belone belone, L. 1761) in the eastern Adriatic Sea. Helgol Mar Res, 65:435–444.

Zouiten, D., Khemis, I. B., Besbes, R., and Cahu, C. (2008). Ontogeny of the digestive tract of thick lipped grey mullet (Chelon labrosus) larvae reared in "mesocosms". Aquaculture, 279.

Zuliani, M. S., Ambrósio, A. M., Francisco, T. M., Balbi, T. J., Okada, E. K., and Gomes, L. C. (2016). Age and growth parameters of the dourado Salminus brasiliensis (Cuvier, 1816) from the river Cuiabá, Mato Grosso State, Brazil. Acta Scientiarum. Biological Sciences, 38:89–97.

Zymonas, N. D. and Propst, D. L. (2007). Ecology of blue sucker and gray redhorse in the lower pecos river, new mexico 2000-2006. Technical report, Conservation Services Division New Mexico Department of Game and Fish Santa Fe, NM.
